# Photoexcited Palladium Complex-Catalyzed Isocyanide Insertion into Inactivated Alkyl Iodides

**DOI:** 10.3390/molecules30122584

**Published:** 2025-06-13

**Authors:** Andrea Messina, Filippo Monticelli, Tiziano Miroglio, Anna Gagliardi, Igor Viviani, Luca Banfi, Renata Riva, Lisa Moni, Andrea Basso, Chiara Lambruschini

**Affiliations:** Department of Chemistry and Industrial Chemistry, University of Genova, Via Dodecaneso 31, 16146 Genova, Italy; andrea.messina@edu.unige.it (A.M.); filippo.monitcelli@gmail.com (F.M.); t.miroglio@gmail.com (T.M.); anna.gagliardi@outlook.it (A.G.); igor.viviani99@gmail.com (I.V.); luca.banfi@unige.it (L.B.); renata.riva@unige.it (R.R.); lisa.moni@unige.it (L.M.); andrea.basso@unige.it (A.B.)

**Keywords:** isocyanide insertion, radical addition, palladium catalysis, photocatalysis, multicomponent reactions, amide synthesis

## Abstract

Isocyanides insertions represent an important transformation in the palladium-catalyzed reactions landscape. However, one of their most significant limitations is in the use of inactivated alkyl electrophiles. Palladium photocatalysis has been proven as a solid tool for the generation of alkyl radicals from alkyl halides, which may engage in subsequent transformations with a variety of reaction partners, closing the catalytic cycle. Herein, we report the mild three-component isocyanide insertions into inactivated alkyl iodides mediated by the catalytic activity of a photoexcited palladium complex. We investigated the scope of the reaction obtaining differently substituted secondary amides in good to high yields. We also investigated the mechanism, hypothesizing a key role of 4-(*N*,*N*-dimethylamino)pyridine in the outcome of the reaction.

## 1. Introduction

**Scheme 1:**
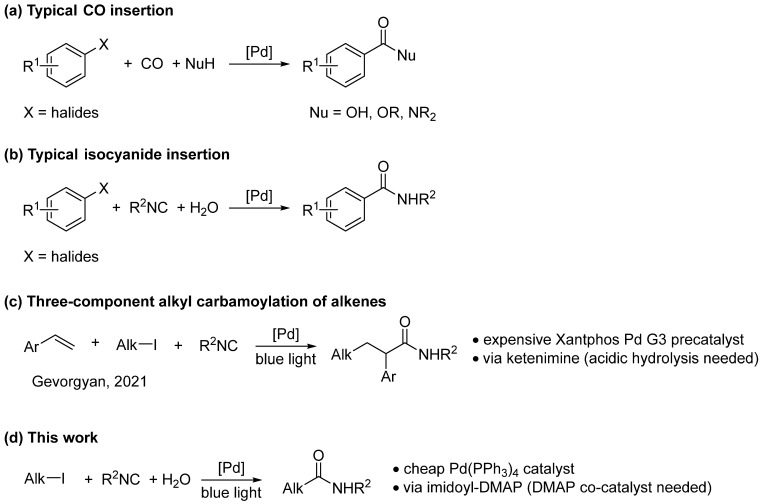
Typical CO (**a**) and isocyanides (**b**) insertion employing aryl halides as electrophiles; (**c**) three-component alkyl carbamoylation of alkenes; (**d**) multicomponent isocyanide insertion employing inactivated alkyl iodides as electrophiles catalyzed by photoexcited Pd species.

The discovery of palladium-catalyzed reactions represented a milestone in organic chemistry history, allowing transformation considered impossible until then. In addition to C-C, C-O, and C-N coupling reactions, the insertion of carbon monoxide (CO) in the presence of a nucleophile has been widely studied, allowing the installation of the important carbonyl moiety onto a wide variety of substrates in a multicomponent fashion (Scheme 1a) [1,2,3]. Isocyanides are recognized as highly versatile reagents that have been widely used in organic synthesis [4,5,6], especially in isocyanide-based multicomponent reactions (MCR), such as Passerini and Ugi [7,8]. Their unique feature is the presence of a carbon atom that can act as a nucleophile and an electrophile. Isocyanides are isoelectronic with CO; in fact, they behave similarly towards palladium, and there are many examples reported in the literature of imidoylative cross-coupling reactions (Scheme 1b), albeit this transformation is less studied than the carbonylative counterpart [9,10,11,12,13]. The use of isocyanides has many advantages over CO, which is a very toxic gas typically used under pressure and in high excess; on the other hand, isocyanides are easy to handle (liquids or solids), usually not toxic [14] (even if some are smelly), and used in stoichiometric amount without the need for special equipment. Moreover, the presence of an alkyl/aryl chain in isocyanide molecules gives the opportunity to install a diversity point in the final compounds, which is extremely important for the exploration of the chemical space. The main issue of imidoylative cross-coupling reactions is the propensity of isocyanides to undergo multiple insertions, which are difficult to suppress [15].

Despite the significant progress achieved through palladium-mediated insertion reactions, this chemistry still has some limitations, the most notable being its restriction to alkenyl and aryl halides. Alkyl electrophiles usually have high energy barriers towards the oxidative addition (OA) and, even if this first step of the mechanism takes place, the resulting intermediate will undergo a fast β-hydride (β-H) elimination, when a hydrogen atom is present. Visible light-induced transition metal (TM) catalysis is a recent and relatively unexplored area of photocatalysis [16,17,18]. Unlike the classical visible light photocatalysis or dual catalysis, here the TM plays a double role: absorbs photons and catalyzes the bond-forming/breaking events. Recently, the use of Pd complexes in combination with blue light was reported for the generation of alkyl radicals through a single electron transfer (SET) barrierless OA [19] and the resulting hybrid Pd(I)/radical species can be engaged in a plethora of transformations [20,21,22]. It has been demonstrated that the use of visible light allows us to overcome the dogma of the two-electron redox mechanism of Pd, suppresses the unproductive β-H elimination, and allows the normally sluggish OA to the electron-rich C(sp^3^)-halogen bond.

Starting from our experience with the chemistry of isocyanides [23] and the use of visible light in multicomponent reactions [], we were interested in the development of the multicomponent isocyanide insertion into inactivated alkyl halides (Scheme 1d). When we started our investigation, this type of transformation was unprecedented in the literature; however, shortly after, Gevorgyan et al. published the Pd-catalyzed 1,2-alkyl carbamoylation of alkenes, using tertiary isocyanides and visible light (Scheme 1c). In the article, the authors demonstrated the insertion of *t*-butyl isocyanide into three alkyl iodides as a proof of concept but did not provide a detailed analysis of the reaction conditions and scope [24]. This prompted us to explore in depth the transformation, and we report our results here.

## 2. Results and Discussion

We started our investigation by studying a model reaction between (3-iodobutyl)benzene **1** and *t*-butyl isocyanide and thoroughly screened the reaction parameters (see Table S1 in the SI for further details). The optimal conditions include the presence of water, Pd(PPh_3_)_4_, DMAP (4-(*N*,*N*-dimethylamino)pyridine), triethylamine, and blue light, and under these conditions, the desired insertion product **2** was isolated in 71% yield (Table 1, entry 1). We also demonstrated that each component of the reaction was essential for its success (Table 1, entries 2–6). It is important to highlight the crucial role of DMAP, which is not immediately obvious. We chose to add catalytic DMAP due to its well-known ability to accelerate acylations (see the proposed mechanism below) [25]. Toluene proved to be more efficient than other solvents that are more miscible with water (Table 1, entries 8–11). Therefore, when dealing with a biphasic system, vigorous agitation is essential (Table 1, entry 7). Concentration plays an important role, and the optimal value ranges from 0.3 M to 0.6 M (Table 1, entries 12–15).

**Figure 1:**
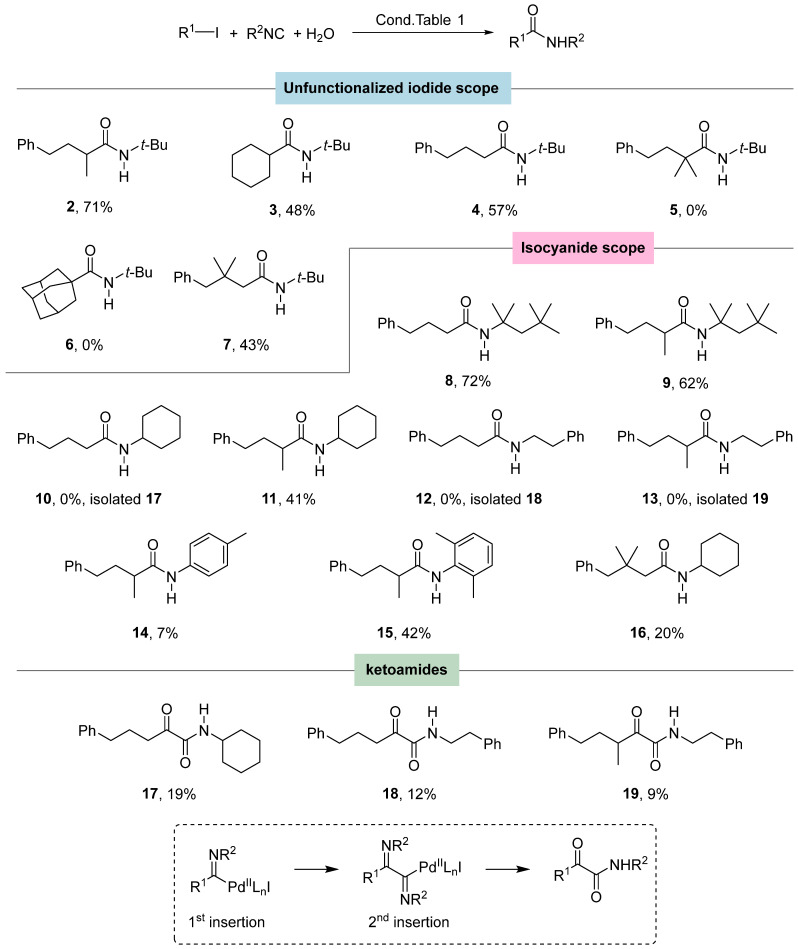
Scope of the reaction; conditions reported in Table 1; 100 mg scale of iodide; isolated yields.

With the optimal conditions in hand, we moved to the reaction scope, starting the investigation on the alkyl iodides (Figure 1). Secondary iodides (**1** and cyclohexyl iodide) and primary (3-iodopropyl)benzene afford the amides in good to high yields (**2**–**4**); on the other hand, tertiary iodides were not reactive under our conditions (amides **5** and **6**).

These results led us to hypothesize that steric hindrance plays a more important role than radical stability. We also tested (3-iodo-2,2-dimethylpropyl)benzene, a primary iodide with a bulky group in β position, and the desired product **7** was obtained in a comparable yield, confirming that, by reducing steric hindrance at the reaction site, the reaction can proceed even with less stable radicals.

Then, we explored the isocyanide scope changing its degree of hindrance (Figure 1). We confirmed that tertiary isocyanides were reactive toward the primary and secondary iodides affording products **8** and **9**, respectively, in high yield. Secondary cyclohexyl isocyanide gives the amide **11** in moderate yield, but when the primary iodide was employed, the ketoamide **17** was isolated in 19% yield, instead of the expected amide **10**. Primary phenethyl isocyanide showed the same behavior with both primary and secondary iodides and the ketoamides **18** and **19** were isolated in low yields, instead of the expected amides **12** and **13**. We explained the formation of the ketoamide by a double insertion of the isocyanide followed by hydrolysis (see box of Figure 1). This finding confirms that a certain degree of hindrance may suppress the multiple insertion of the isocyanide [15]. Methyl isocyanide and benzyl isocyanide were tested with iodide **1**, but there were no traces of the desired products. A steric trend was also observed with aromatic isocyanides with an increase in the yield as the hindrance increased (**14** vs. **15**). When we tested 1-iodoadamantane with *n*-butyl isocyanide or cyclohexyl isocyanide, we did not detect any traces of the desired products, therefore we can conclude that tertiary iodides are not reactive under the conditions reported in Table 1. (3-Iodo-2,2-dimethylpropyl)benzene was also tested with cyclohexyl isocyanide and afforded the amide **16**, albeit in low yield. The observed yields can be explained in terms of steric hindrance: when steric hindrance is too low, multiple insertions may take over; conversely, if it is too high, the reaction partners cannot interact efficiently, leading to an interruption of the catalytic cycle.

These first sets of experiments show that the best combination is a secondary alkyl iodide and a tertiary or secondary isocyanide. Then, we tested the functional groups’ tolerance to the reaction, employing *t*-BuNC or cyclohexyl isocyanide and functionalized secondary iodides. Surprisingly, when we employed 2-(3-iodobutyl)thiophene **20** together with cyclohexyl isocyanide, the desired amide **21** was obtained in a very low yield (12%, Table 2, entry 1). Our initial hypothesis was a poisoning of the metal catalyst by the sulfur atom and therefore we decided to carry out a second optimization campaign (Table 2; see Table S2 in the SI for further details) studying this reaction as a model.

When we applied the previously optimized conditions (Table 2, entry 1), a considerable amount of unreacted iodide **20** was present after 48 h. Starting with this clue, we increased the reaction time, and a doubling of the yield was observed (entry 2), albeit the absolute value was not satisfying. We hypothesized that the reagent, or the catalyst, may lose its effectiveness over time, therefore the addition of palladium complex (entry 3) and isocyanide (entry 4) was performed after 48 h, and the mixture was let react for a further 24 h. We were happy to observe a boost in the yield, so we tried to increase the equivalents of isocyanide from the beginning and an even higher yield was obtained (entry 5). It is worth mentioning that blue LEDs generate a significant amount of heat; therefore, a cooling system is needed. We normally use circulating tap water to cool down the rack without any fine control of the temperature; however, the value was always below room temperature (15–18 °C). So, we tested the effect of the temperature using a thermostatic system and we were pleased to see a further increase in the yield at 25 °C, obtaining amide **21** in 64% yield (entry 6). Between conditions of entry 3 and entry 6, we selected the latter as optimal due to the shorter time and the lower loading of the expensive catalyst.

**Figure 2:**
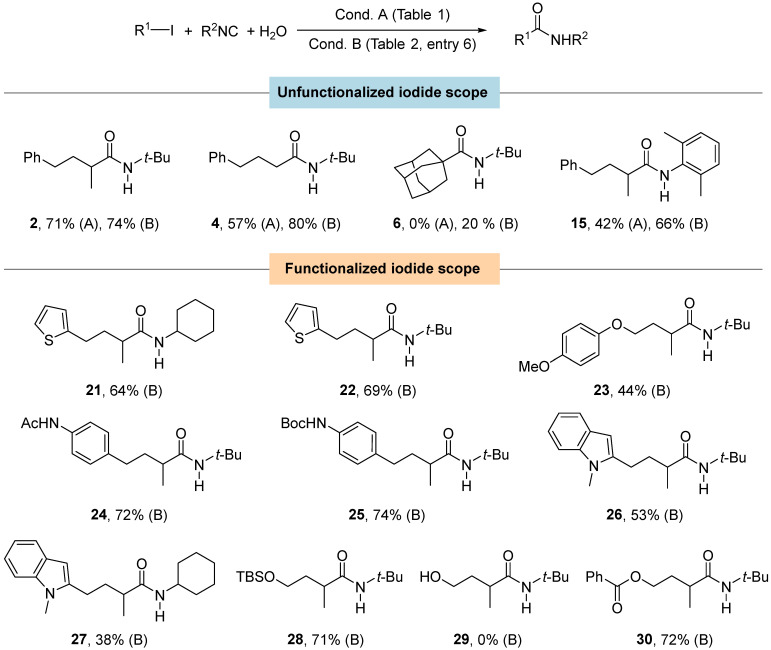
Scope of the reaction. Comparison of the yields under conditions A (Table 1) and conditions B (Table 2, entry 6); 100 mg scale of iodide; isolated yields.

Satisfied by these results, we decided to employ the new conditions to selected alkyl iodides already used in Figure 1, and we were pleased to observe a remarkable increase in the yields of amides **4**, **6**, and **15**; on the other hand, amide **2** was isolated with almost the same yield (Figure 2). These findings prove that partial degradation of the palladium catalyst and/or the isocyanide may happen regardless of the presence of the sulfur atom.

Finally, we tested the functional group tolerance of the reaction (Figure 2). The reaction is compatible with the presence of heteroaromatic residues (**21**, **22**, **26**, and **27**), amides (**24**), esters (**30**), the Boc-protecting group (**25**), and silyl ethers (**28**). The presence of a methoxy group lowers the yield (**23**), while the free alcohol is not compatible with the studied transformation (**29**). Although the excess of isocyanide may increase the probability of multiple insertion events, we did not isolate the corresponding ketoamide during the purification of the product reported in Figure 2.

**Figure 3:**
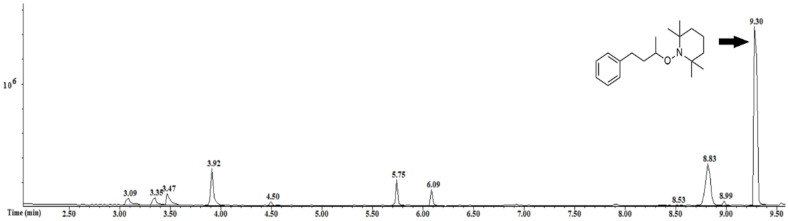
Gas Chromatography–Mass Spectrometry (GC–MS) of the radical-trapping experiment. The main peak is attributed to the adduct between TEMPO and the alkyl radical coming from **1** (*t*_R_ = 9.30 min). The amide **2** has a *t*_R_ = 8.665 min under the thermal method employed, but its peak is absent from the chromatogram.

Next, a set of experiments was carried out to investigate the mechanism of the transformation. The formation of the alkyl radical was undoubtedly proven by a radical trapping experiment. We added TEMPO (2,2,6,6-tetramethyl-1-piperidinyloxy), a known radical trap, to the reaction between iodide **1** and *t*-BuNC, following the conditions reported in Entry 1 of Table 1, and we analyzed the mixture by GC-MS (Figure 3). The chromatogram showed the absence of amide **2** and a main peak with *m*/*z* = 289, compatible with the alkyl radical trapped by TEMPO, proving the hypothesis.

**Figure 4:**
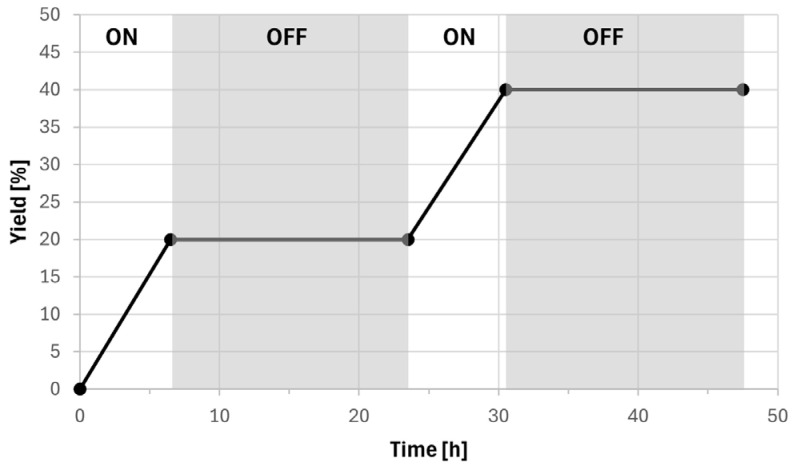
On/off experiment. The yields of the amide **2** were determined by ^1^H NMR using 4-iodoanisole as an internal standard.

Then, we carried out an on/off experiment to rule out the presence of a radical chain mechanism (see SI for details). The reaction between iodide **1** and *t*-BuNC following the conditions reported in Entry 1 of Table 1 was analyzed. As shown in Figure 4, we observed an increase in the yield only during irradiation times, whereas the yield was stable over time if the vial was kept in the dark. These data confirm the hypothesis of the radical catalytic cycle and prove that our product is stable in the reaction mixture.

**Scheme 2:**
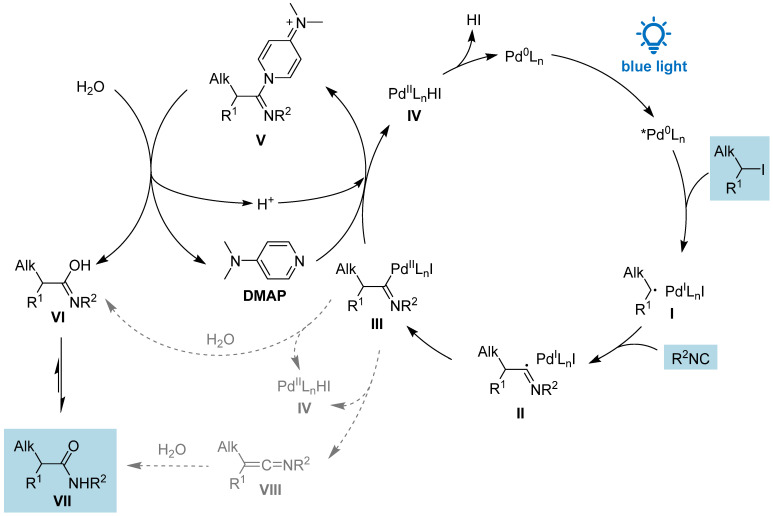
Proposed mechanism. Alternative pathways unlikely to happen are shown in grey.

According to the results described above and the control experiments in Table 1, and considering previous reports, we propose the following mechanisms (Scheme 2). The photoexcitation of Pd(PPh_3_)_4_ is the initial step and, according to the literature, the active catalytic species is the triplet state of *Pd(0), which interacts with the alkyl iodide generating the hybrid alkyl Pd(I)-radical species **I** via a smooth SET OA [19]. Then, species **I** is intercepted by the isocyanide to give the imidoyl radical **II,** which pairs with Pd(I) collapsing into the imidoyl-Pd(II) **III**. We can envisage several possible pathways starting from this intermediate. The most straightforward is the nucleophilic attack of H_2_O leading directly to **VI**, which tautomerizes to product **VII**. However, the presence of water alone is not enough to afford the product, as proven by the control experiment (Table 1, entry 4), therefore direct S_N_Ac is unlikely to happen. It is worth noting that our conditions require the presence of both water and DMAP, and this prompted us to propose an alternative mechanism, given the known ability of DMAP to accelerate the acylation process. The nitrogen of DMAP attacks the intermediate **III** forming the imidoyl-DMAP **V**, which is highly activated towards the nucleophilic substitution by water, with the concomitant formation of the tautomer of the amide **VI** and DMAP, which can be employed in catalytic amounts. We believe that the role of DMAP is to ease the breakage of the Pd-C bond and activate the imidoyl moiety toward the attack by a poor nucleophile, such as water. This sequence of steps also leads to the Pd(II) species **IV**, which undergoes a reductive elimination, releasing HI (quenched by Et_3_N) and regenerating the Pd(0) catalyst. We cannot exclude that the nucleophilic attack of DMAP on intermediate **III** promotes the release of iodide and the simultaneous reduction to Pd(0). The iodide may then serve as a counterion for species **V**, which undergoes hydrolysis by water, with Et_3_N scavenging the resulting proton (see Scheme S13 in the SI). Starting from **III**, another reasonable pathway is a β-H elimination affording the ketenimine **VIII** followed by its hydrolysis to furnish the amide. In the literature, there are examples of the formation of ketenimine from imidoyl-Ni [26,27] and imidoyl-Pd [24], although in the latter case, the β-H elimination occurs in a benzylic position and the extended conjugation of the ketenimine might be the driving force. To investigate this possibility, we submitted the iodide **1** and *t*-BuNC to the standard conditions of Table 1 except for the absence of water and DMAP, and after 48 h, we added 1 M HCl stirring for 1 h. The ^1^H NMR of the reaction showed an iodide/product ratio of 9:1, suggesting that the ketenimine is not playing a crucial role under our conditions.

## 3. Materials and Methods

**General remarks.** All non-aqueous reactions were performed under an inert atmosphere of argon or nitrogen. Inert gases were passed over a U-tube of silica and activated 3 Å molecular sieves. Photochemical reactions were carried out in a dedicated apparatus consisting of an aluminum cooling plate (LWH 160 × 100 × 25 mm) with 6 OSRAM^®^ (Munich, Germany) Oslon SSL 80 LDCQ7P (nominal 450 nm, royal blue) LEDs mounted in series powered by a MeanWell^®^ (New Taipei City, Taiwan) LPC-20-700 constant current power supply (700 mA) and a water-cooled aluminum vessel holder. The vessel holder (LWH 170 × 110 × 38 mm) holds the sealed vials 10 mm over the LEDs in a fixed position while cooling both the LED plate and the vial, keeping the temperature of the latter below 20 °C. Both the LED plate and the water-cooled vessel were custom-built, while the vials were purchased from Wicom International Co. (Heppenheim, Germany) (WIC43005, 5 mL crimp top vial 38.5 × 22.0 mm; WIC44510 20 mm crimp caps with 3.0 mm PTFE septum). For reactions performed under a controlled temperature, the vessel holder was connected to a thermostat Lab. Companion (Daejeon, Republic of Korea) RW-0525G and the temperature was 25 °C or 35 °C. Reactions were stirred magnetically and monitored by thin-layer chromatography (TLC). Anhydrous solvents were purchased by Merck (Darmstadt, Germany). The solvents used for the isocyanide insertion were further degassed with Ar for 20 min. Analytical TLC was performed using MERCK Silica Gel 60 F254 0.25 mm TLC glass plates and visualized by ultraviolet light (UV, 254 nm). TLC plates were also stained by dipping and heating with cerium ammonium molybdate (CAM, Hanessian’s stain, 1 g Ce(SO_4_)_2_·4H_2_O in 31 mL of H_2_SO_4_ and 469 mL of H_2_O), KMnO_4_ (1.5 g KMnO_4_, 10 g K_2_CO_3_, 1 mL of 10% aq. NaOH in 200 mL of H_2_O), and 20% H_2_SO_4_ in ethanol (spraying and heating). R_f_ values were measured after an elution of 7–9 cm. After extractions, the aqueous phases were always re-extracted three times with the appropriate organic solvent, and the organic extracts were always dried over Na_2_SO_4_ and filtered before evaporation to dryness. Concentration under reduced pressure was performed by rotavapor at 40 °C. Chromatographic purification was performed as flash chromatography (FC) on MERCK Geduran^®^ Si 60 (40–63 µm) under positive pressure, and the crude mixtures were loaded as solid absorbed on silica or as a solution in the eluent. Purified compounds were dried further under high vacuum (~10^−2^ torr). Petroleum ether (PE) is 40–60 °C. Nuclear Magnetic Resonance (NMR) spectra were recorded on VARIAN MERCURY (Palo Alto, CA, USA) (300 MHz ^1^H and 75 MHz ^13^C) or on Jeol JNM-ECZ400R (Akishima, Tokyo, Japan) (400 MHz ^1^H and 100 MHz ^13^C). Chemical shifts (δ) are reported in parts per million (ppm) using tetramethylsilane (TMS, 0.00 ppm) or the residual solvent signal (CDCl_3_ at 77.16 ppm for ^13^C) as internal standards. The data are reported as follows: chemical shift, integration, multiplicity (indicated as s, singlet; d, doublet; t, triplet; q, quartet; m, multiplet; and combinations thereof), coupling constants (*J*) in Hertz (Hz), and assignation (when possible). Peak assignments were performed with the aid of gCOSY (gradient correlation spectroscopy), gHSQC (gradient heteronuclear single-quantum correlation spectroscopy), and gHMBC (gradient heteronuclear multiple-bond correlation spectroscopy). All NMR spectra were analyzed using the software MestReNova (Santiago de Compostela, Spain) (Mestrelab Research^®^ v. 14.2). Fourier transform infrared (FTIR) spectra were recorded on a Perkin Elmer Spectrum 65 (Perkin Elmer, Waltham, MA, USA) instrument, equipped with a universal attenuated total reflectance (ATR) sampling accessory. IR peaks are reported as decreasing wavenumber (ῡ, cm^−1^). All spectra were elaborated using the software Spectrum (PerkinElmer^®^ v.10.03) and processed with a 10% smooth algorithm. Melting points were determined with an electrothermal apparatus Büchi (Flawil, Switzerland) B-535. The thermal program used is 1 °C/min. High-Performance Liquid Chromatography (HPLC) analysis was performed on Agilent (Palo Alto, CA, USA) HP 1100 equipped with a DAD detector (220 nm) and column C6 PHENYLIC RP column (150 × 3 mm, 3 μ) at 25 °C with flow = 0.34 mL/min and gradient H_2_O/CH_3_CN, (A = H_2_O, B = CH_3_CN), 0 min A = 55%, 30 min B = 40%. Gas Chromatography–Mass Spectrometry (GC-MS) analysis was performed on Shimadzu (Kyoto, Japan) GC−MS-QP2010 SE with an AOC-20i Plus auto-injector mounting an Avantor (Radnor, PA, USA) Hichrom HI-5 MS column (internal diameter 0.25 mm, film thickness 0.25 mm, length 30 m). A Mass Analyzer Metal quadrupole mass filter with pre-rods was used and the ionization was obtained through Electronic Impact (EI) operating with an ionization potential of 70 eV. He was used as carrier gas with the following details: flow = 1.0 mL/min, V_inj_ = 1 µL, and a split ratio of 1:10. Samples were prepared as EtOAc solutions at 100 ppm: solvent delay = 2.5 min, mass range 35–400, T_inj_ = 250 °C, T detector = 250 °C. Three thermal methods were employed: Method A: T_in_ = 100 °C, t_in_ = 3 min, gradient = 25 °C/min, T_final_ = 300 °C; Method B: T_in_ = 70 °C, t_in_ = 3 min, gradient = 25 °C/min, T_final_ = 300 °C; and Method C: T_in_ = 120 °C, t_in_ = 3 min, gradient = 25 °C/min, T_final_ = 300 °C. The analysis was processed using the software GCMS LabSolution Shimadzu (v. 4.52) and the data are reported as follows: retention time (*t*_R_, min), *m*/*z* (threshold 3%), and relative abundance. High-Resolution Mass Spectrometer (HRMS) analyses were carried out on a Waters (Milford, MA, USA) Synapt G2 QToF mass spectrometer. MS signals were acquired in ESI positive ionization mode.

**Isocyanide insertion general procedure, condition A.** In a photochemical vial, the iodide (1 equiv) and DMAP (6 mol%) were added and then Ar was fluxed through a septum. Then, degassed anhydrous toluene (0.3 M), Et_3_N (1.7 equiv), isocyanide (1.1 equiv), Pd(PPh_3_)_4_ (6 mol%), and bidistilled water (40 equiv) were added. The septum was replaced by the cap and crimped. The vial was placed in the photochemical plate and stirred for 48 h. The crude mixture was diluted with s.s. (saturated solution) NH_4_Cl, extracted with Et_2_O, washed with brine, dried (Na_2_SO_4_), and evaporated to dryness. The crude product was purified by FC on silica.

**Isocyanide insertion general procedure, condition B.** In a photochemical vial, the iodide (1 equiv) and DMAP (6 mol%) were added and then Ar was fluxed through a septum. Then, degassed anhydrous toluene (0.3 M), Et_3_N (1.7 equiv), isocyanide (2.2 equiv), Pd(PPh_3_)_4_ (6 mol%), and bidistilled water (40 equiv) were added. The septum was replaced by the cap and crimped. The vial was placed in the photochemical plate and stirred for 48 h at 25 °C. The crude mixture was diluted with s.s. NH_4_Cl, extracted with Et_2_O, washed with brine, dried (Na_2_SO_4_), and evaporated to dryness. The crude product was purified by FC on silica.

***N*-(*tert*-Butyl)-2-methyl-4-phenylbutanamide (2).** Following the general procedure (condition B), the mixture of (3-iodobutyl)benzene **1** (100 mg, 0.384 mmol), DMAP (2.8 mg, 0.0231 mmol), Pd(PPh_3_)_4_ (26.7 mg, 0.0231 mmol), Et_3_N (91 µL, 0.653 mmol), *t*-butyl isocyanide (96 µL, 0.845 mmol), and bidistilled H_2_O (277 µL, 15.36 mmol) in dry toluene (1.3 mL) was reacted. After extraction, the crude product was purified by silica FC (PE/Et_2_O 8:2), delivering **2** as a pale-yellow solid (66 mg, 74% yield). **R_f_** 0.29 (PE/Et_2_O 8:2, UV (weak), CAM (weak)). **^1^H NMR** (400 MHz, CDCl_3_, 27 °C) δ 7.31–7.27 (m, 2H, 2×CH_arom_), 7.24–7.11 (m, 3H, 3×CH_arom_), 5.18 (s, 1H, NH), 2.66 (ddd, *J* = 14.3, 9.2, 5.3 Hz, 1H, PhC*H*H), 2.61–2.51 (m, 1H, PhCH*H*), 2.08–1.91 (m, 2H, PhCH_2_C*H*H + CH), 1.71–1.64 (m, 1H, PhCH_2_CH*H*), 1.36 (s, 9H, C(CH_3_)_3_), 1.12 (d, *J* = 6.6 Hz, 3H, CH*_3_*). **^13^C NMR** (101 MHz, CDCl_3_, 27 °C) δ 175.6 (C=O), 142.0 (C_q_ Ar), 128.6 (2×CH_arom_), 128.5 (2×CH_arom_), 126.0 (*p*-CH_arom_), 51.2 (*C*(CH_3_)_3_), 41.6 (CH), 35.9 (PhCH_2_*C*H_2_), 33.7 (Ph*C*H_2_), 29.0 (C(*C*H_3_)_3_), 18.2 (CH_3_). **GC-MS**: *t*_R_ = 8.665 min (Method A); *m*/*z* (%): 39 (7), 41 (24), 42 (9), 44 (5), 51 (3), 55 (6), 56 (6), 57 (37), 58 (29), 65 (13), 69 (4), 72 (8), 73 (100), 74 (20), 77 (4), 86 (3), 91 (67), 92 (7), 100 (5), 104 (3), 114 (3), 115 (3), 129 (96), 130 (8). **IR** ῡ: 3309, 3068, 3029, 2969, 2926, 1642, 1544, 1496, 1481, 1453, 1389, 1360, 1286, 1259, 1225, 1094, 1068, 1033, 948, 928, 888, 790, 762, 742, 719, 695, 672. **HRMS** (ESI+): calcd. for C_15_H_24_NO [M+H]^+^ 234.1852, found 234.1857.

***N*-(*tert*-Butyl)cyclohexanecarboxamide (3).** Following the general procedure (condition A), the mixture of cyclohexyl iodide (101 mg, 0.48 mmol), DMAP (3.5 mg, 0.0288 mmol), Pd(PPh_3_)_4_ (33.3 mg, 0.0288 mmol), Et_3_N (114 µL, 0.816 mmol), *t*-butyl isocyanide (60 µL, 0.528 mmol), and bidistilled H_2_O (277 µL, 15.36 mmol) in dry toluene (1.6 mL) was reacted. After extraction, the crude product was purified by silica FC (PE/AcOEt 9:1), delivering **3** as pale-yellow oil (42 mg, 48% yield). **R_f_** 0.24 (PE/AcOEt 9:1, 20% H_2_SO_4_ in EtOH). **^1^H NMR** (300 MHz, CDCl_3_, 27 °C) δ 5.21 (s, 1H, NH), 1.94 (tt, *J* = 11.6, 3.4 Hz, 1H, CH), 1.86–1.72 (m, 2H, CH_2_), 1.70–1.63 (m, 2H, CH_2_), 1.49–1.36 (m, 2H, CH_2_), 1.33 (s, 9H, C(CH_3_)_3_), 1.30–1.15 (m, 4H, 2×CH_2_). **^13^C NMR** (75 MHz, CDCl_3_, 27 °C) δ 175.7 (C=O), 50.9 (*C*(CH_3_)_3_), 46.4 (CH), 30.0 (CH_2_), 29.9 (CH_2_), 29.0 (C(*C*H_3_)_3_), 25.9 (3×CH_2_). **GC-MS:** *t*_R_ = 6.644 min (Method A); *m*/*z* (%): 39 (15), 40 (3), 41 (47), 42 (14), 43 (4), 44 (3), 53 (5), 54 (5), 55 (45), 56 (18), 57 (52), 58 (100), 59 (12), 72 (15), 81 (3), 82 (3), 83 (36), 84 (3), 93 (3), 98 (5), 111 (9), 112 (6), 115 (5), 128 (57), 129 (5), 142 (5), 168 (4), 183 (16) [M]^+^. **HRMS** (ESI+): calcd. for C_11_H_22_NO [M+H]^+^ 184.1696, found 184.1704.

***N*-(*tert*-butyl)-4-phenylbutanamide (4).** Following the general procedure (condition B), the mixture of (3-iodopropyl)benzene (**S3**) (100 mg, 0.406 mmol), DMAP (3.0 mg, 0.0244 mmol), Pd(PPh_3_)_4_ (28.2 mg, 0.0244 mmol), Et_3_N (96 µL, 0.691 mmol), *t*-butyl isocyanide (101 µL, 0.894 mmol), and bidistilled H_2_O (293 µL, 16.3 mmol) in dry toluene (1.4 mL) was reacted. After extraction, the crude product was purified by silica FC (PE/Et_2_O 7:3), delivering **4** as a pale-yellow solid (71 mg, 80% yield). **R_f_** 0.20 (PE/Et_2_O 7:3, UV, CAM). **M.p.** 68.8–70.2 °C. **^1^H NMR** (400 MHz, CDCl_3_, 27 °C) δ 7.32–7.24 (m, 2H, 2×CH_arom_), 7.22–7.16 (m, 3H, 3×CH_arom_), 5.20 (bs, 1H, NH), 2.64 (t, *J* = 7.4 Hz, 2H, PhCH_2_), 2.08 (t, *J* = 7.3 Hz, 2H, CH_2_CO), 1.94 (p, *J* = 7.4 Hz, 2H, CH_2_C*H*_2_CH_2_), 1.34 (s, 9H, C(CH_3_)_3_). **^13^C NMR** (101 MHz, CDCl_3_, 27 °C) δ 172.1 (C=O), 141.8 (C_q_ Ar), 128.7 (2×CH_arom_), 128.5 (2×CH_arom_), 126.0 (*p*-CH_arom_), 51.2 (*C*(CH_3_)_3_), 36.9 (*C*H_2_CO), 35.2 (Ph*C*H_2_), 29.0 (C(*C*H_3_)_3_), 27.2 (CH_2_*C*H_2_CH_2_). **GC-MS**: *t*_R_ = 8.705 min (Method A); *m*/*z* (%): 39 (9), 41 (23), 42 (11), 43 (4), 44 (4), 51 (4), 55 (12), 56 (6), 57 (26), 58 (51), 59 (100), 60 (40), 65 (12), 72 (5), 77 (5), 78 (3), 91 (43), 92 (4), 100 (10), 104 (3), 105 (3), 115 (77), 116 (6), 117 (4), 129 (3), 147 (5), 219 (7) [M]^+^. **IR** ῡ: 3308, 3070, 3030, 2966, 2931, 2864, 1665, 1639, 1546, 1497, 1453, 1421, 1390, 1360, 1281, 1227, 1158, 1133, 1081, 1033, 965, 923, 907, 843, 759, 738, 698, 667, 622. **HRMS** (ESI+): calcd. for C_14_H_22_NO [M+H]^+^ 220.1696, found 220.1699.

**(*3r*,*5r*,*7r*)-*N*-(*tert*-Butyl)adamantane-1-carboxamide (6).** Following the general procedure (condition B), the mixture of 1-iodoadamantane (100 mg, 0.381 mmol), DMAP (2.8 mg, 0.0229 mmol), Pd(PPh_3_)_4_ (26.5 mg, 0.0229 mmol), Et_3_N (90 µL, 0.648 mmol), *t*-butyl isocyanide (95 µL, 0.839 mmol), and bidistilled H_2_O (275 µL, 15.3 mmol) in dry toluene (1.3 mL) was reacted. After extraction, the crude product was purified by silica FC (PE/Et_2_O 8:2), delivering **6** as a pale-yellow solid (18 mg, 20% yield). **^1^H NMR** (400 MHz, CDCl_3_, 27 °C) δ 5.37 (bs, 1H, NH), 2.03 (bs, 3H, 3×CH), 1.83–1.78 (m, 6H, 3×2-CH_2_), 1.76–1.65 (m, 6H, 3×4-CH_2_), 1.33 (s, 9H, C(CH_3_)_3_). **^13^C NMR** (101 MHz, CDCl_3_, 27 °C) δ 177.5 (C=O), 50.7 (*C*(CH_3_)_3_), 41.0 (*C*-CO), 39.5 (3×2-CH_2_), 36.7 (3×4-CH_2_), 29.0 (C(*C*H_3_)_3_), 28.3 (3×CH). **GC-MS**: *t*_R_ = 8.980 min (Method A); *m*/*z* (%): 39 (5), 41 (17), 42 (6), 53 (4), 55 (6), 56 (4), 57 (12), 58 (4), 65 (3), 67 (8), 77 (9), 79 (21), 80 (3), 81 (5), 91 (9), 92 (3), 93 (19), 94 (3), 107 (10), 135 (100), 136 (15), 163 (8), 180 (11), 193 (3), 220 (13), 235 (18) [M]^+^. **IR** ῡ: 3327, 2974, 2958, 2900, 2850, 1636, 1533, 1470, 1445, 1387, 1359, 1343, 1328, 1287, 1267, 1228, 1178, 1106, 1090, 1045, 981, 944, 925, 913, 816, 746, 673, 635. **HRMS** (ESI+): calcd. for C_15_H_26_NO [M+H]^+^ 236.2009, found 236.2017.

***N*-(*tert*-Butyl)-3,3-dimethyl-4-phenylbutanamide (7).** Following the general procedure (condition A), the mixture of (3-iodo-2,2-dimethylpropyl)benzene (**S8**) (100 mg, 0.365 mmol), DMAP (2.7 mg, 0.0219 mmol), Pd(PPh_3_)_4_ (25.3 mg, 0.0219 mmol), Et_3_N (85 µL, 0.620 mmol), *t*-butyl isocyanide (45 µL, 0.402 mmol), and bidistilled H_2_O (263 µL, 14.6 mmol) in dry toluene (1.2 mL) was reacted. After extraction, the crude product was purified by silica FC (PE/Et_2_O 8:2), delivering **7** as a pale-yellow solid (39 mg, 43% yield). **R_f_** 0.24 (PE/Et_2_O 7:3, UV, CAM). **M.p.** 72.2–73.7 °C. **^1^H NMR** (400 MHz, CDCl_3_, 27 °C) δ 7.30–7.24 (m, 2H, 2×CH_arom_), 7.23–7.16 (m, 3H, 3×CH_arom_), 5.20 (bs, 1H, NH), 2.66 (s, 2H, PhCH_2_), 1.94 (s, 2H, CH_2_CO), 1.35 (s, 9H, C(CH_3_)_3_), 1.01 (s, 6H, C(CH_3_)_2_). **^13^C NMR** (101 MHz, CDCl_3_, 27 °C) δ 171.2 (C=O), 138.9 (C_q_ Ar), 131.0 (2×CH_arom_), 127.8 (2×CH_arom_), 126.1 (*p*-CH_arom_), 51.3 (*C*(CH_3_)_3_)), 49.0 (*C*H_2_CO), 48.5 (PhCH_2_), 34.6 (*C*(CH_3_)_2_), 28.9 (C(*C*H_3_)_3_), 27.4 (C(*C*H_3_)_2_). **GC-MS**: *t*_R_ = 8.980 min (Method A); *m*/*z* (%): 39 (8), 41 (28), 42 (9), 43 (3), 44 (4), 55 (10), 56 (10), 57 (88), 58 (38), 59 (74), 60 (36), 65 (11), 72 (3), 83 (3), 91 (50), 92 (8), 100 (17), 105 (4), 115 (100), 116 (8), 117 (5), 132 (3), 133 (4), 247 (3) [M]^+^. **IR** ῡ: 3311, 3064, 3030, 2966, 2925, 1637, 1607, 1543, 1492,1452, 1389, 1354, 1329, 1317, 1290, 1265, 1225, 1182, 1145, 1114, 1073, 1031, 991, 934, 897, 791, 754, 713, 702, 685, 613. **HRMS** (ESI+): calcd. for C_16_H_26_NO [M+H]^+^ 248.2009, found 248.2003.

**4-Phenyl-*N*-(2,4,4-trimethylpentan-2-yl)butanamide (8).** Following the general procedure (condition A), the mixture of (3-iodopropyl)benzene (**S3**) (100 mg, 0.406 mmol), DMAP (3.0 mg, 0.0244 mmol), Pd(PPh_3_)_4_ (28.2 mg, 0.0244 mmol), Et_3_N (96 µL, 0.691 mmol), 1,1,3,3-tetramethylbutyl isocyanide (78 µL, 0.447 mmol), and bidistilled H_2_O (293 µL, 16.3 mmol) in dry toluene (1.4 mL) was reacted. After extraction, the crude product was purified by silica FC (PE/Et_2_O 7:3), delivering **8** as a pale-yellow solid (80 mg, 72% yield). **R_f_** 0.31 (PE/Et_2_O 7:3, UV (weak), CAM (weak)). **^1^H NMR** (400 MHz, CDCl_3_, 27 °C) δ 7.33–7.24 (m, 2H, 2×CH_arom_), 7.23–7.15 (m, 3H, 3×CH_arom_), 5.17 (bs, 1H, NH), 2.65 (t, *J* = 7.5 Hz, 2H, PhCH_2_), 2.08 (t, *J* = 7.6 Hz, 2H, CH_2_CO), 2.00–1.88 (m, 2H, CH_2_C*H*_2_CH_2_), 1.74 (s, 2H, C*H*_2_C(CH_3_)_3_), 1.39 (s, 6H, C(C*H*_3_)_2_), 0.99 (s, 9H, C(C*H*_3_)_3_). **^13^C NMR** (101 MHz, CDCl_3_, 27 °C) δ 171.8 (C=O), 141.8 (C_q_ Ar), 128.6 (2×CH_arom_), 128.5 (2×CH_arom_), 126.0 (*p*-CH_arom_), 55.2 (*C*(CH_3_)_2_), 51.6 (*C*H_2_C(CH_3_)_3_), 37.2 (*C*H_2_CO), 35.3 (Ph*C*H_2_), 31.8 (*C*(CH_3_)_3_), 31.6 (C(*C*H_3_)_3_), 29.4 (C(*C*H_3_)_2_), 27.1 (CH_2_*C*H_2_CH_2_). **GC-MS**: *t*_R_ = 10.110 min (Method A); *m*/*z* (%): 39 (5), 41 (19), 42 (7), 43 (7), 55 (13), 56 (4), 57 (29), 58 (100), 59 (48), 60 (4), 65 (6), 72 (6), 77 (3), 91 (32), 92 (3), 97 (13), 100 (3), 112 (3), 114 (12), 115 (4), 129 (3), 147 (11), 164 (8), 171 (9), 204 (9), 218 (4), 275 (2) [M]^+^. **IR** ῡ: 3295, 3083, 3028, 2949, 2866, 1639, 1545, 1477, 1453, 1387, 1361, 1348, 1315, 1279, 1260, 1229, 1159, 1142, 1080, 1053, 1030, 969, 929, 908, 857, 809, 743. **HRMS** (ESI+): calcd. for C_18_H_30_NO [M+H]^+^ 276.2322, found 276.2327.

**2-Methyl-4-phenyl-*N*-(2,4,4-trimethylpentan-2-yl)butanamide (9).** Following the general procedure (condition A), the mixture of (3-iodobutyl)benzene **1** (100 mg, 0.384 mmol), DMAP (2.8 mg, 0.0231 mmol), Pd(PPh_3_)_4_ (26.7 mg, 0.0231 mmol), Et_3_N (91 µL, 0.653 mmol), 1,1,3,3-tetramethylbutyl isocyanide (74 µL, 0.422 mmol), and bidistilled H_2_O (277 µL, 15.36 mmol) in dry toluene (1.3 mL) was reacted. After extraction, the crude product was purified by silica FC (PE/Et_2_O 8:2), delivering **9** as a pale-yellow solid (69 mg, 62% yield). **R_f_** 0.27 (PE/Et_2_O 9:1, UV (weak), CAM (weak)). **^1^H NMR** (400 MHz, CDCl_3_, 27 °C) δ 7.32–7.23 (m, 2H, 2×CH_arom_), 7.22–7.13 (m, 3H, 3×CH_arom_), 5.18 (s, 1H, NH), 2.71–2.50 (m, 2H, PhCH_2_), 2.09–1.90 (m, 2H, CH + PhCH_2_C*H*H), 1.81 (d, *J* = 14.9 Hz, 1H, C*H*HC(CH_3_)_3_), 1.70–1.58 (m, 2H, CH*H*C(CH_3_)_3_ + PhCH_2_CH*H*), 1.42 (s, 3H, C*H*_3_CCH_3_), 1.39 (s, 3H, CH_3_CC*H*_3_), 1.11 (d, *J* = 6.7 Hz, 3H, CHC*H*_3_), 0.99 (s, 9H, C(CH_3_)_3_). **^13^C NMR** (101 MHz, CDCl_3_, 27 °C) δ 175.2 (C=O), 142.1 (C_q_ Ar), 128.6 (2×CH_arom_), 128.5 (2×CH_arom_), 126.0 (*p*-CH_arom_), 55.3 (CH_3_*C*CH_3_), 52.2 (*C*H_2_C(CH_3_)_3_), 41.8 (CH), 35.9 (PhCH_2_*C*H_2_), 33.7 (Ph*C*H_2_), 31.8 (*C*(CH_3_)_3_), 31.7 (C(*C*H_3_)_3_), 29.33 (*C*H_3_C*C*H_3_), 29.26 (CH_3_CCH_3_), 18.0 (CH*C*H_3_). **GC-MS**: *t*_R_ = 10.085 min (Method A), *m*/*z* (%): 39 (4), 41 (20), 42 (7), 43 (8), 44 (3), 55 (12), 56 (5), 57 (40), 58 (100), 59 (4), 65 (9), 69 (7), 72 (8), 73 (77), 74 (9), 77 (3), 91 (88), 92 (8), 97 (15), 112 (4), 114 (14), 115 (3), 133 (8), 161 (13), 178 (9), 185 (28), 186 (3), 218 (8), 232 (5). **IR** ῡ: 3306, 3071, 2948, 2870, 1639, 1546, 1496, 1475, 1453, 1387, 1363, 1287, 1260, 1228, 1178, 1155, 1093, 1055, 1033, 956, 928, 903, 883, 861, 807, 785, 766, 742, 723, 693, 679. **HRMS** (ESI+): calcd. for C_19_H_32_NO [M+H]^+^ 290.2478, found 290.2470.

***N*-Cyclohexyl-2-methyl-4-phenylbutanamide (11).** Following the general procedure (condition A), the mixture of (3-iodobutyl)benzene **1** (100 mg, 0.384 mmol), DMAP (2.8 mg, 0.0231 mmol), Pd(PPh_3_)_4_ (26.7 mg, 0.0231 mmol), Et_3_N (91 µL, 0.653 mmol), *c*-Hex isocyanide (53 µL, 0.422 mmol), and bidistilled H_2_O (277 µL, 15.36 mmol) in dry toluene (1.3 mL) was reacted. After extraction, the crude product was purified by silica FC (PE/Et_2_O 6:4), delivering **11** as a pale-yellow solid (41 mg, 41% yield). **R_f_** 0.28 (PE/Et_2_O 6:4, UV (weak), CAM (weak)). **^1^H NMR** (400 MHz, CDCl_3_, 27 °C) δ 7.31–7.27 (m, 2H, 2×CH_arom_), 7.22–7.15 (m, 3H, 3×CH_arom_), 5.23 (bd, *J* = 7.5 Hz, 1H, NH), 3.86–3.74 (m, 1H, CH *c*-Hex), 2.66 (ddd, *J* = 14.7, 9.4, 5.6 Hz, 1H, PhC*H*H), 2.56 (ddd, *J* = 13.8, 8.9, 7.0 Hz, 1H, PhCH*H*), 2.15–2.05 (m, 1H, C*H*CH_3_), 2.04–1.86 (m, 3H, PhCH_2_C*H*H + 2×C*H*H *c*-Hex), 1.76–1.58 (m, 4H, PhCH_2_CH*H* + 2×C*H*H *c*-Hex + C*H*H *c*-Hex), 1.45–1.30 (m, 2H, 2×CH*H c*-Hex), 1.22–1.04 (m, 3H, 2×CH*H c*-Hex + CH*H c*-Hex), 1.15 (d, *J* = 6.8 Hz, 3H, CH_3_). **^13^C NMR** (101 MHz, CDCl_3_, 27 °C) δ 175.2 (C=O), 141.9 (C_q._), 128.5 (2×CH_arom_), 128.5 (2×CH_arom_), 126.0 (*p*-CH_arom_), 48.0 (CH *c*-Hex), 41.0 (*C*HCH_3_), 35.9 (PhCH_2_*C*H_2_), 33.6 (Ph*C*H_2_), 33.5 (2-CH_2_ *c*-Hex), 33.3 (6-CH_2_ *c*-Hex), 25.6 (CH_2_ *c*-Hex), 25.0 (2×CH_2_ *c*-Hex), 18.2 (CH_3_). **GC-MS**: *t*_R_ = 10.590 min (Method A); *m*/*z* (%): 39 (6), 41 (16), 42 (3), 43 (6), 44 (5), 54 (4), 55 (21), 56 (13), 57 (4), 65 (10), 67 (6), 69 (4), 72 (4), 73 (11), 74 (100), 75 (4), 77 (3), 79 (3), 82 (5), 83 (6), 91 (70), 92 (6), 98 (5), 104 (3), 126 (11), 155 (64), 156 (7). **IR** ῡ (cm^−1^): 3295, 3032, 2931, 2853, 2221, 1634, 1542, 1494, 1446, 1389, 1366, 1348, 1300, 1269, 1247, 1233, 1171, 1152, 1122, 1101, 1076, 1048, 1031, 973, 944, 891, 865, 846, 786, 749, 724, 697, 648. **HRMS** (ESI+): calcd. for C_17_H_26_NO [M+H]^+^ 260.2009, found 260.2004.

**2-Methyl-4-phenyl-*N*-(*p*-tolyl)butanamide (14).** Following the general procedure (condition A), the mixture of (3-iodobutyl)benzene **1** (100 mg, 0.384 mmol), DMAP (2.8 mg, 0.0231 mmol), Pd(PPh_3_)_4_ (26.7 mg, 0.0231 mmol), Et_3_N (91 µL, 0.653 mmol), 1-isocyano-4-methylbenzene (49.5 mg, 0.422 mmol), and bidistilled H_2_O (277 µL, 15.36 mmol) in dry toluene (1.3 mL) was reacted. After extraction, the crude product was purified by silica FC (PE/Et_2_O 7:3), delivering **14** as a pale-yellow oil (7 mg, 7% yield). **R_f_** 0.22 (PE/Et_2_O 7:3, UV, CAM). **^1^H NMR** (400 MHz, CDCl_3_, 27 °C) δ 7.43–7.38 (m, 2H, *para*-syst. 2×CH_arom_), 7.33–7.27 (m, 2H, 2×CH_arom_), 7.23–7.17 (m, 3H, 3×CH_arom_), 7.15–7.11 (m, 2H, *para*-syst. 2×CH_arom_), 7.00 (bs, 1H, NH), 2.73 (ddd, *J* = 14.5, 9.0, 5.8 Hz, 1H, PhC*H*H), 2.64 (dt, *J* = 14.2, 7.5 Hz, 1H, PhCH*H*), 2.38–2.23 (m, 4H, CH + C*H*_3_-C_6_H_4_), 2.16–2.05 (m, 1H, PhCH_2_C*H*H), 1.85–1.73 (m, 1H, PhCH_2_CH*H*), 1.26 (d, *J* = 6.8 Hz, 3H, CHC*H*_3_). **^13^C NMR** (100 MHz, CDCl3, 27 °C) δ 174.4 (C=O), 141.7 (C_q._), 135.5 (C_q._), 134.0 (C_q._), 129.6 (*para*-syst. 2×CH_arom_), 128.6 (2×CH_arom_), 128.6 (2×CH_arom_), 126.2 (CH_arom_), 120.0 (*para*-syst. 2×CH_arom_), 41.8 (CH), 35.8 (PhCH_2_*C*H_2_), 33.6 (Ph*C*H_2_), 21.0 (*C*H_3_-C_6_H_4_), 18.2 (CH*C*H_3_). **HRMS** (ESI+): calcd. for C_18_H_22_NO [M+H]^+^ 268.1696, found 268.1704.

***N*-(2,6-Dimethylphenyl)-2-methyl-4-phenylbutanamide (15).** Following the general procedure (condition B), the mixture of (3-iodobutyl)benzene **1** (100 mg, 0.384 mmol), DMAP (2.8 mg, 0.0231 mmol), Pd(PPh_3_)_4_ (26.7 mg, 0.0231 mmol), Et_3_N (91 µL, 0.653 mmol), 2,6-dimethylphenyl isocyanide (111 mg, 0.845 mmol), and bidistilled H_2_O (277 µL, 15.36 mmol) in dry toluene (1.3 mL) was reacted. After extraction, the crude product was purified by silica FC (PE/Et_2_O 7:3), delivering **15** as a pale-yellow solid (71 mg, 66% yield). **R_f_** 0.28 (PE/Et_2_O 7:3, UV (weak), CAM (weak)). **^1^H NMR** (400 MHz, CDCl_3_, 27 °C) δ 7.33–7.27 (m, 2H, 2×CH_arom_ of Ph), 7.24–7.18 (m, 3H, 3×CH_arom_ of Ph), 7.12–7.06 (m, 3H, 3×CH_arom_ of dimethylphenyl), 6.68 (s, 1H, NH amide), 2.84–2.64 (m, 2H, PhC*H*_2_), 2.54–2.40 (m, 1H, CH), 2.24 (s, 6H, 2×CH_3_ of dimethylphenyl), 2.23–2.09 (m, 1H, PhCH_2_C*H*H), 1.86–1.73 (m, 1H, PhCH_2_CH*H*), 1.33 (d, *J* = 6.9 Hz, 3H, CHC*H*_3_). **^13^C NMR** (101 MHz, CDCl_3_, 27 °C) δ 174.5 (C=O), 141.9 (C_q._ of Ph), 135.5 (2×*C_q._*-CH_3_), 133.9 (*C_q._*-NH), 128.6 (2×CH_arom_ of Ph), 128.6 (2×CH_arom_ of Ph), 128.4 (2×CH_arom_ of dimethylphenyl), 127.4 (CH_arom_ of dimethylphenyl), 126.1 (CH_arom_ of Ph), 41.3 (CH), 36.0 (PhCH_2_*C*H_2_), 33.9 (Ph*C*H_2_CH_2_), 18.7 (2×CH_3_ of dimethylphenyl), 18.7 (CH*C*H_3_). **GC-MS**: *t*_R_ = 11.415 min (Method A); *m*/*z* (%): 39 (4), 41 (4), 51 (3), 55 (3), 65 (11), 77 (10), 78 (3), 79 (5), 91 (100), 92 (9), 103 (4), 104 (3), 105 (5), 106 (6), 120 (27), 121 (35), 122 (4), 133 (3), 148 (34), 149 (4), 177 (38), 178 (5), 281 (1) [M]^+^. **IR** ῡ (cm^−1^): 3226, 3027, 2967, 2926, 2850, 1669, 1648, 1602, 1526, 1496, 1474, 1453, 1430, 1367, 1265, 1234, 1206, 1162, 1122, 1090, 1052, 1033, 945, 844, 774, 765, 742, 729, 697, 648. **HRMS** (ESI+): calcd. for C_19_H_24_NO [M+H]^+^ 282.1852, found 282.1849.

***N*-Cyclohexyl-3,3-dimethyl-4-phenylbutanamide (16).** Following the general procedure (condition A), the mixture of (3-iodo-2,2-dimethylpropyl)benzene **S8** (91.8 mg, 0.335 mmol), DMAP (2.4 mg, 0.0201 mmol), Pd(PPh_3_)_4_ (23.2 mg, 0.0201 mmol), Et_3_N (19 µL, 0.569 mmol), *c*-Hex isocyanide (46 µL, 0.368 mmol), and bidistilled H_2_O (241 µL, 13.4 mmol) in dry toluene (1.2 mL) was reacted. After extraction, the crude product was purified by silica FC (PE/Et_2_O 7:3), delivering **16** as a pale-yellow solid (18 mg, 20% yield). **R_f_** 0.23 (PE/Et_2_O 7:3, UV, CAM). **^1^H NMR** (400 MHz, CDCl_3_, 27 °C) δ 7.26 (t, *J* = 3.6 Hz, 2H, 2×CH_arom_), 7.24–7.18 (m, 3H, 2×CH_arom_), 5.22 (bd, *J* = 7.3 Hz, 1H, NH), 3.79 (tdt, *J* = 12.1, 8.1, 3.9 Hz, 1H, CH *c*-Hex), 2.67 (s, 2H, PhC*H*_2_), 1.99 (s, 2H, CH_2_CO), 1.97–1.88 (m, 2H, 2×C*H*H *c*-Hex), 1.70 (dt, *J* = 13.2, 3.4 Hz, 2H, 2×C*H*H *c*-Hex), 1.61 (dt, *J* = 12.7, 3.6 Hz, 1H, C*H*H *c*-Hex), 1.41–1.34 (m, 2H, 2×CH*H c*-Hex), 1.16–1.07 (m, 3H, CH*H c*-Hex + 2×CH*H c*-Hex), 1.01 (s, 6H, 2×CH_3_). **^13^C NMR** (101 MHz, CDCl_3_, 27 °C) δ 170.8 (C=O), 138.9 (C_q._), 131.0 (2×CH_arom_), 127.9 (2×CH_arom_), 126.1 (CH_arom_), 48.5 (PhCH_2_), 48.3 (*C*H_2_CO), 48.2 (CH *c*-Hex), 34.6 (*C*_q._-(CH_3_)_2_), 33.5 (2×CH_2_ *c*-Hex), 27.4 (2×CH_3_), 25.7 (CH_2_ *c*-Hex), 25.0 (2×CH_2_ *c*-Hex). **GC-MS**: *t*_R_ = 13.960 min (Method A); *m*/*z* (%): 41 (15), 43 (6), 55 (26), 56 (14), 57 (20), 59 (5), 60 (100), 65 (5), 67 (5), 82 (7), 83 (36), 91 (36), 92 (5), 98 (8), 126 (8), 141 (97), 142 (9). **IR** ῡ (cm^−1^): 3267, 3076, 3026, 2929, 2853, 1632, 1552, 1489, 1465, 1448, 1389, 1368, 1346, 1259, 1229, 1181, 1152, 1143, 1124, 1071, 1028, 994, 892, 846, 798, 763, 720, 701, 630. **HRMS** (ESI+): calcd. for C_18_H_28_NO [M+H]^+^ 274.2165, found 274.2171.

***N*-Cyclohexyl-2-methyl-4-(thiophen-2-yl)butanamide (21).** Following the general procedure (condition B), the mixture of 2-(3-iodobutyl)thiophene **20** (100 mg, 0.376 mmol), DMAP (2.8 mg, 0.0226 mmol), Pd(PPh_3_)_4_ (26.4 mg, 0.0226 mmol), Et_3_N (89 µL, 0.639 mmol), *t*-butyl isocyanide (103 µL, 0.827 mmol), and bidistilled H_2_O (271 µL, 15.04 mmol) in dry toluene (1.3 mL) was reacted. After extraction, the crude product was purified by silica FC (PE/Et_2_O 7:3), delivering **21** as a pale-yellow solid (64 mg, 64% yield). **R_f_** 0.28 (PE/Et_2_O 7:3, UV, CAM). **^1^H NMR** (400 MHz, CDCl_3_, 27 °C) δ 7.12 (dd, *J* = 5.1, 1.1 Hz, 1H, 5-CH thiophene), 6.92 (dd, *J* = 5.1, 3.4 Hz, 1H, 4-CH thiophene), 6.82–6.75 (m, 1H, 3-CH thiophene), 5.30 (s, 1H, NH), 3.80 (dddd, *J* = 14.9, 10.8, 8.3, 3.9 Hz, 1H, CH *c*-Hex), 2.93–2.73 (m, 2H, C*H*_2_CH_2_CH), 2.21–2.10 (m, 1H CH_2_C*H*), 2.05 (dtd, *J* = 14.4, 8.2, 5.7 Hz, 1H, C*H*HCH), 1.98–1.86 (m, 2H, 2×C*H*H *c*-Hex), 1.79–1.57 (m, 4H, CH*H*CH + 2×C*H*H *c*-Hex + C*H*H *c*-Hex), 1.44–1.30 (m, 2H, 2×CH*H c*-Hex), 1.23–1.03 (m, 3H, 2×CH*H c*-Hex + CH*H c*-Hex), 1.15 (d, *J* = 6.7 Hz, 3H, CH_3_). **^13^C NMR** (101 MHz, CDCl_3_, 27 °C) δ 175.0 (C=O), 144.8 (C_q._), 126.9 (4-CH thiophene), 124.5 (3-CH thiophene), 123.2 (5-CH thiophene), 48.1 (CH *c*-Hex), 40.6 (CH), 36.1 (*C*H_2_CH), 33.5 (2-CH_2_ *c*-Hex), 33.3 (6-CH_2_ *c*-Hex), 27.7 (*C*H_2_CH_2_CH), 25.7 (CH_2_ *c*-Hex), 25.0 (2×CH_2_ *c*-Hex), 18.1 (CH_3_). **GC-MS**: *t*_R_ = 10.620 min (Method A); *m*/*z* (%): 39 (5), 41 (15), 42 (3), 43 (4), 44 (6), 45 (7), 53 (7), 54 (3), 55 (18), 56 (8), 57 (5), 67 (6), 69 (5), 72 (4), 73 (10), 74 (100), 75 (4), 82 (5), 83 (3), 97 (36), 98 (6), 110 (3), 126 (10), 155 (45), 156 (4), 265 (1) [M]^+^. **IR** ῡ (cm^−1^): 3282, 3099, 2931, 2853, 2221, 1636, 1544, 1440, 1387, 1372, 1346, 1303, 1271, 1245, 1234, 1225, 1181, 1151, 1099, 1075, 1030, 976, 945, 892, 854, 823, 796, 698, 614. **HRMS** (ESI+): calcd. for C_15_H_24_NOS [M+H]^+^ 266.1573, found 266.1576.

***N*-(*tert*-Butyl)-2-methyl-4-(thiophen-2-yl)butanamide (22).** Following the general procedure (condition B), the mixture of 2-(3-iodobutyl)thiophene **20** (100 mg, 0.376 mmol), DMAP (2.8 mg, 0.0226 mmol), Pd(PPh_3_)_4_ (26.4 mg, 0.0226 mmol), Et_3_N (89 µL, 0.639 mmol), *t*-butyl isocyanide (93 µL, 0.827 mmol), and bidistilled H_2_O (271 µL, 15.04 mmol) in dry toluene (1.3 mL) was reacted. After extraction, the crude product was purified by silica FC (PE/Et_2_O 7:3)m, delivering **22** as a pale-yellow solid (76 mg, 69% yield). **R_f_** 0.38 (PE/Et_2_O 7:3, UV, CAM). **^1^H NMR** (400 MHz, CDCl_3_, 27 °C) δ 7.11 (dd, *J* = 5.1, 1.1 Hz, 1H, 5-CH thiophene), 6.92 (dd, *J* = 5.1, 3.4 Hz, 1H, 4-CH thiophene), 6.82–6.75 (m, 1H, 3-CH thiophene), 5.26 (bs, 1H, NH), 2.96–2.69 (m, 2H, C*H*_2_CH_2_CH), 2.15–1.96 (m, 2H, C*H*HCH + CH), 1.70 (dtd, *J* = 13.3, 7.8, 5.0 Hz, 1H, CH*H*CH), 1.36 (s, 9H, C(CH_3_)_3_), 1.12 (d, *J* = 6.6 Hz, 3H, CH_3_). **^13^C NMR** (101 MHz, CDCl_3_, 27 °C) δ 175.4 (C=O), 144.8 (C_q._), 126.9 (4-CH thiophene), 124.5 (3-CH thiophene), 123.2 (5-CH thiophene), 51.2 (*C*(CH_3_)_3_), 41.1 (CH), 36.2 (*C*H_2_CH), 29.0 (C(*C*H_3_)_3_), 27.7 (*C*H_2_CH_2_CH), 18.1 (CH_3_). **GC-MS**: *t*_R_ = 8.720 min (Method A); *m*/*z* (%): 39 (3), 41 (11), 42 (3), 44 (3), 45 (6), 53 (5), 55 (3), 56 (3), 57 (17), 58 (10), 69 (3), 72 (8), 73 (100), 74 (18), 97 (30), 98 (3), 100 (5), 110 (3), 114 (3), 129 (82), 130 (7), 239 (4) [M]^+^. **IR** ῡ (cm^−1^): 3302, 3072, 2964, 2925, 2870, 1641, 1543, 1479, 1450, 1440, 1389, 1359, 1310, 1294, 1262, 1229, 1185, 1140, 1082, 1065, 1038, 933, 890, 852, 829, 781, 762, 748, 691, 679, 670, 649, 615. **HRMS** (ESI+): calcd. for C_13_H_22_NOS [M+H]^+^ 240.1417, found 240.1427.

***N*-(*tert*-Butyl)-4-(4-methoxyphenoxy)-2-methylbutanamide (23).** Following the general procedure (condition B), the mixture of 1-(3-Iodobutoxy)-4-methoxybenzene **S12** (89.7 mg, 0.293 mmol), DMAP (2.1 mg, 0.0176 mmol), Pd(PPh_3_)_4_ (20.3 mg, 0.0176 mmol), Et_3_N (69 µL, 0.498 mmol), *t*-butyl isocyanide (36 µL, 0.322 mmol), and bidistilled H_2_O (211 µL, 11.7 mmol) in dry toluene (1.1 mL) was reacted. After extraction, the crude product was purified by silica FC (PE/Et_2_O 7:3), delivering **23** as a pale-yellow solid (36 mg, 44% yield). **R_f_** 0.20 (PE/Et_2_O 7:3, UV, CAM). **^1^H NMR** (400 MHz, CDCl_3_, 27 °C) δ 6.83 (s, 4H, 4×CH_arom_), 5.37 (s, 1H, NH), 3.96 (dt, *J* = 10.1, 5.2 Hz, 1H, OC*H*H), 3.88 (td, *J* = 9.2, 4.5 Hz, 1H, OCH*H*), 3.77 (s, 3H, OCH_3_), 2.50–2.37 (m, 1H, CH), 2.00 (ddt, *J* = 14.1, 9.4, 4.7 Hz, 1H, C*H*HCH), 1.84 (ddt, *J* = 14.2, 8.9, 5.4 Hz, 1H, CH*H*CH), 1.27 (s, 9H, C(CH_3_)_3_), 1.16 (d, *J* = 6.9 Hz, 3H, CHC*H*_3_). **^13^C NMR** (101 MHz, CDCl_3_, 27 °C) δ 175.3 (C=O), 154.0 (C_q._), 153.1 (C_q._), 115.4 (2 2×CH_arom_), 114.8 (2×CH_arom_), 66.3 (OCH_2_), 55.8 (OCH_3_), 51.1 (*C*(CH_3_)_3_), 38.5 (CH), 34.0 (*C*H_2_CH), 28.9 (C(*C*H_3_)_3_), 18.0 (CH*C*H_3_). **GC-MS**: *t*_R_ = 10.125 min (Method A); *m*/*z* (%): 39 (4), 41 (17), 42 (3), 44 (6), 55 (9), 56 (5), 57 (35), 58 (8), 73 (3), 77 (4), 81 (3), 83 (3), 100 (100), 101 (6), 109 (9), 124 (7), 137 (3), 156 (29), 157 (3). **IR** ῡ (cm^−1^): 3347, 2962, 2926, 2215, 1645, 1538, 1508, 1454, 1361, 1287, 1229, 1184, 1109, 1076, 1030, 991, 970, 912, 882, 819, 727, 694, 627. **HRMS** (ESI+): calcd. for C_16_H_26_NO_3_ [M+H]^+^ 280.1907, found 280.1910.

**4-(4-Acetamidophenyl)-*N*-(*tert*-butyl)-2-methylbutanamide (24).** Following the general procedure (condition B), the mixture of *N*-(4-(3-iodobutyl)phenyl)acetamide **S17** (100 mg, 0.315 mmol), DMAP (2.3 mg, 0.0189mmol), Pd(PPh_3_)_4_ (21.9 mg, 0.0189 mmol), Et_3_N (75 µL, 0.536 mmol), *t*-butyl isocyanide (78 µL, 0.654 mmol), and bidistilled H_2_O (227 µL, 12.6 mmol) in dry toluene (1.1 mL) was reacted. After extraction, the crude product was purified by silica FC (PE/AcOEt 2:8), delivering **24** as a pale-yellow solid (67 mg, 72% yield). **R_f_** 0.38 (PE/AcOEt 2:8, UV, CAM). **^1^H NMR** (400 MHz, CDCl_3_, 27 °C) δ 7.42–7.34 (m, 2H, 2×3-CH_arom_), 7.19–7.00 (m, 3H, 2×2-CH_arom_ + AcNH), 5.16 (bs, 1H, NH), 2.65–2.56 (m, 1H, C*H*HCH_2_CH), 2.55–2.47 (m, 1H, CH*H*CH_2_CH), 2.16 (s, 3H, CH_3_CO), 2.04–1.87 (m, 2H, CH + C*H*HCH), 1.68–1.55 (m, 1H, CH*H*CH), 1.34 (s, 9H, C(CH_3_)_3_), 1.10 (d, *J* = 6.6 Hz, 3H, CHC*H*_3_). **^13^C NMR** (101 MHz, CDCl_3_, 27 °C) δ 175.5 (CH*C*=O), 168.2 (CH_3_*C*=O), 138.1 (C-C_q._), 135.8 (N-C_q._), 129.0 (2×2-CH_arom_), 120.2 (2×3-CH_arom_), 51.1 (*C*(CH_3_)_3_), 41.4 (CH), 35.8 (*C*H_2_CH), 33.0 (*C*H_2_CH_2_CH), 29.0 (C(*C*H_3_)_3_), 24.7 (*C*H_3_CO), 18.2 (CH*C*H_3_). **GC-MS**: *t*_R_ = 11.845 min (Method A); *m*/*z* (%), 39 (3), 41 (12), 42 (4), 43 (16), 44 (6), 55 (3), 56 (5), 57 (17), 58 (10), 72 (6), 73 (90), 74 (18), 77 (5), 78 (6), 79 (3), 91 (3), 100 (4), 105 (4), 106 (34), 107 (4), 114 (3), 119 (6), 120 (4), 129 (100), 130 (8), 132 (3), 146 (3), 148 (3), 162 (6), 207 (3), 290 (3) [M]^+^. **IR** ῡ (cm^−1^): 3321, 3196, 3129, 3072, 2967, 2922, 2864, 1677, 1644, 1607, 1541, 1514, 1458, 1411, 1390, 1372, 1362, 1317, 1267, 1229, 1121, 1062, 1022, 962, 914, 882, 822, 763, 720, 672, 650, 609. **HRMS** (ESI+): calcd. for C_17_H_27_N_2_O_2_ [M+H]^+^ 291.2067, found 291.2069.

***tert*-Butyl (4-(4-(*tert*-butylamino)-3-methyl-4-oxobutyl)phenyl) carbamate (25).** Following the general procedure (condition B), the mixture of *tert*-butyl (4-(3-iodobutyl)phenyl)carbamate **S20** (100 mg, 0.266 mmol), DMAP (2 mg, 0.0160 mmol), Pd(PPh_3_)_4_ (18.5 mg, 0.0160 mmol), Et_3_N (63 µL, 0.453 mmol), *t*-butyl isocyanide (66 µL, 0.586 mmol), and bidistilled H_2_O (192 µL, 10.7mmol) in dry toluene (1.1 mL) was reacted. After extraction, the crude product was purified by silica FC (PE/Et_2_O 7:3), delivering **25** as a pale-yellow solid (66 mg, 74% yield). **R_f_** 0.31 (PE/Et_2_O 7:3, UV, CAM). **^1^H NMR** (400 MHz, CDCl_3_, 27 °C) δ 7.29–7.24 (m, 2H, 2×CH_arom_), 7.09 (d, *J* = 8.1 Hz, 2H, 2×CH_arom_), 6.47 (bs, 1H, OCON*H*), 5.18 (bs, 1H, CHCON*H*), 2.69–2.41 (m, 2H, C*H*_2_CH_2_CH), 2.05–1.86 (m, 2H, C*H*HCH + CH), 1.70–1.55 (m, 1H, CH*H*CH), 1.51 (s, 9H, OC(CH_3_)_3_), 1.35 (s, 9H, NC(CH_3_)_3_), 1.10 (d, *J* = 6.6 Hz, 3H, CHC*H*_3_). **^13^C NMR** (101 MHz, CDCl_3_, 27 °C) δ 175.6 (CH*C*=O), 153.0 (OC=ONH), 136.7 (C-*C*_q_), 136.3 (N-C_q_), 129.0 (2×CH_arom_), 118.9 (2×CH_arom_), 80.5 (O*C*(CH_3_)_3_), 51.2 (N*C*(CH_3_)_3_), 41.3 (CH), 35.9 (*C*H_2_CH), 32.9 (*C*H_2_CH_2_CH), 29.0 (NC(*C*H_3_)_3_), 28.5 (OC(*C*H_3_)_3_), 18.2 (CH*C*H_3_). **GC-MS**: the compound degrades during the analysis. A big and broad peak (*t*_R_ = 11.485 min) is obtained in the chromatograph alongside the product without the Boc- protecting group. *t*_R_ = 11.485 min (Method C); *m*/*z* (%): 39 (13), 40 (5), 41 (25), 42 (8), 43 (8), 44 (51), 45 (5), 51 (3), 53 (3), 55 (6), 56 (11), 57 (13), 58 (9), 59 (3), 65 (3), 69 (3), 72 (5), 73 (100), 74 (17), 75 (4), 77 (7), 78 (5), 79 (4), 91 (4), 96 (4), 100 (4), 105 (3), 106 (33), 107 (5), 118 (3), 119 (11), 120 (4), 129 (52), 130 (5), 132 (3), 133 (4), 135 (8), 146 (4), 147 (8), 191 (4), 193 (9), 194 (3), 207 (39), 208 (6), 209 (5), 221 (5), 248 (10), 249 (4), 267 (3), 281 (11), 282 (4), 283 (3). **IR** ῡ (cm^−1^): 3348, 3272, 2965, 2931, 1699, 1651, 1603, 1536, 1478, 1451, 1413, 1391, 1363, 1318, 1246, 1229, 1158, 1089, 1054, 1021, 947, 904, 833, 805, 735, 719, 665, 635, 607. **HRMS** (ESI+): calcd. for C_20_H_33_N_2_O_3_ [M+H]^+^ 349.2486, found 349.2480.

***N*-(*tert*-Butyl)-2-methyl-4-(1-methyl-1*H*-indol-3-yl)butanamide (26).** Following the general procedure (condition B), the mixture of 3-(3-iodobutyl)-1-methyl-1*H*-indole **S22** (100 mg, 0.319 mmol), DMAP (2.3 mg, 0.0190 mmol), Pd(PPh_3_)_4_ (22.0 mg, 0.0190 mmol), Et_3_N (78 µL, 0.542 mmol), *t*-butyl isocyanide (79 µL, 0.703 mmol), and bidistilled H_2_O (230 µL, 12.8 mmol) in dry toluene (1.1 mL) was reacted. After extraction, the crude product was purified by silica FC (PE/Et_2_O 6:4), delivering **26** as a pale-yellow solid (48 mg, 53% yield). **R_f_** 0.35 (PE/Et_2_O 6:4, UV, CAM). **^1^H NMR** (400 MHz, CDCl_3_, 27 °C) δ 7.59 (d, *J* = 7.9 Hz, 1H, 4-CH indole), 7.28 (d, *J* = 8.2 Hz, 1H, 7-CH indole), 7.21 (t, *J* = 7.2 Hz, 1H, 6-CH indole), 7.09 (t, *J* = 7.4 Hz, 1H, 5-CH indole), 6.84 (s, 1H, 2-CH indole), 5.17 (s, 1H, NH), 3.74 (s, 3H, N-CH_3_), 2.82–2.67 (m, 2H, C*H*_2_CH_2_CH), 2.16–1.98 (m, 2H, C*H*HCH + CH), 1.80–1.69 (m, 1H, CH*H*CH), 1.34 (s, 9H, C(CH_3_)_3_), 1.14 (d, *J* = 6.6 Hz, 3H, CHC*H*_3_). **^13^C NMR** (101 MHz, CDCl_3_, 27 °C) δ 175.9 (C=O), 137.2 (8-C indole), 128.0 (9-C indole), 126.3 (2-CH indole), 121.7 (6-CH indole), 119.2 (4-CH indole), 118.7 (5-CH indole), 114.7 (3-C indole), 109.3 (7-CH indole), 51.1 (*C*(CH_3_)_3_), 41.7 (*C*HCH_3_), 35.1 (*C*H_2_CH), 32.7 (NCH_3_), 29.0 (C(*C*H_3_)_3_), 22.9 (*C*H_2_CH_2_CH), 18.2 (CH*C*H_3_). **GC-MS**: *t*_R_ = 11.090 min (Method A); *m*/*z* (%): 39 (5), 41 (20), 42 (14), 56 (8), 57 (23), 58 (13), 72 (7), 73 (100), 74 (22), 77 (11), 100 (6), 102 (7), 103 (6), 115 (12), 128 (10), 129 (89), 130 (11), 131 (6), 143 (14), 144 (79), 145 (12), 157 (41), 158 (28), 214 (5), 286 (32), 287 (6). **IR** ῡ (cm^−1^): 3279, 3082, 2969, 2932, 1642, 1616, 1554, 1470, 1451, 1425, 1361, 1325, 1292, 1265, 1234, 1208, 1177, 1156, 1119, 1090, 1075, 1058, 1045, 1026, 1011, 947, 932, 920, 893, 836, 811, 765, 734, 699, 651, 608. **HRMS** (ESI+): calcd. for C_18_H_27_N_2_O [M+H]^+^ 287.2118, found 287.2114.

***N*-Cyclohexyl-2-methyl-4-(1-methyl-1*H*-indol-3-yl)butanamide (27).** Following the general procedure (condition B), the mixture of 3-(3-iodobutyl)-1-methyl-1*H*-indole **S22** (100 mg, 0.319 mmol), DMAP (2.3 mg, 0.0190 mmol), Pd(PPh_3_)_4_ (22.0 mg, 0.0190 mmol), Et_3_N (78 µL, 0.542 mmol), *c*-Hex isocyanide (87 µL, 0.703 mmol), and bidistilled H_2_O (230 µL, 12.8 mmol) in dry toluene (1.1 mL) was reacted. After extraction, the crude product was purified by silica FC (PE/Et_2_O 6:4), delivering **27** as a pale-yellow solid (38 mg, 38% yield). **R_f_** 0.28 (PE/Et_2_O 6:4, UV, CAM). **^1^H NMR** (400 MHz, CDCl_3_, 27 °C) δ 7.58 (d, *J* = 7.9 Hz, 1H, 4-CH indole), 7.28 (d, *J* = 8.2 Hz, 1H, 7-CH indole), 7.21 (td, *J* = 7.0, 0.8 Hz, 1H, 6-CH indole), 7.09 (td, *J* = 7.4, 1.0 Hz, 1H, 5-CH indole), 6.82 (s, 1H, 2-CH indole), 5.25 (d, *J* = 7.7 Hz, 1H, NH), 3.85–3.75 (m, 1H, CH *c*-Hex), 3.73 (s, 3H, NCH_3_), 2.83–2.67 (m, 2H, C*H*_2_CH_2_CH), 2.24–2.12 (m, 1H, C*H*CH_3_), 2.12–2.00 (m, 1H, C*H*HCH), 1.97–1.85 (m, 2H, 2×C*H*H *c*-Hex), 1.82–1.74 (m, 1H, CH*H*CH), 1.74–1.65 (m, 2H, 2×C*H*H *c*-Hex), 1.64–1.54 (m, 1H, C*H*H *c*-Hex), 1.45–1.29 (m, 2H, 2×CH*H c*-Hex), 1.16 (d, *J* = 6.8 Hz, 3H, CHC*H*_3_), 1.14–1.02 (m, 3H, CH*H c*-Hex + 2×CH*H c*-Hex). **^13^C NMR** (101 MHz, CDCl_3_, 27 °C) δ 175.5 (C=O), 137.2 (8-C indole), 128.0 (9-C indole), 126.3 (2-CH indole), 121.6 (6-CH indole), 119.1 (4-CH indole), 118.7 (5-CH indole), 114.6 (3-C indole), 109.3 (7-CH indole), 48.0 (CH *c*-Hex), 41.2 (C*H*CH_3_), 35.0 (C*H*_2_CH), 33.5 (2-CH_2_ *c*-Hex), 33.3 (6-CH_2_ *c*-Hex), 32.7 (NCH_3_), 25.7 (CH_2_ *c*-Hex), 25.0 (2×CH_2_ *c*-Hex), 22.9 (C*H*_2_CH_2_CH), 18.2 (CHC*H*_3_). **GC-MS**: *t*_R_ = 13,240 min (Method A); *m*/*z* (%): 39 (4), 41 (16), 42 (9), 43 (7), 44 (10), 54 (5), 55 (18), 56 (11), 57 (4), 67 (8), 72 (3), 73 (7), 74 (100), 75 (4), 77 (8), 82 (6), 83 (4), 98 (3), 102 (4), 103 (4), 115 (7), 117 (3), 126 (10), 127 (3), 128 (6), 129 (3), 130 (3), 131 (4), 143 (9), 144 (50), 145 (7), 155 (38), 156 (6), 157 (21), 158 (19), 159 (3), 312 (13) [M]^+^. **IR** ῡ (cm^−1^): 3677, 3289, 3055, 2927, 2852, 1635, 1543, 1471, 1449, 1424, 1375, 1325, 1249, 1232, 1153, 1130, 1015, 942, 891, 736, 669. **HRMS** (ESI+): calcd. for C_20_H_29_N_2_O [M+H]^+^ 313.2274, found 313.2270.

***N*-(*tert*-Butyl)-4-((*tert*-butyldimethylsilyl)oxy)-2-methylbutanamide (28).** Following the general procedure (condition B), the mixture of *tert*-butyl(3-iodobutoxy)dimethylsilane **S26** (100 mg, 0.318 mmol), DMAP (2.3 mg, 0.0191 mmol), Pd(PPh_3_)_4_ (22 mg, 0.0191 mmol), Et_3_N (75 µL, 0.541 mmol), *t*-butyl isocyanide (79 µL, 0.700 mmol), and bidistilled H_2_O (229 µL, 12.7 mmol) in dry toluene (1.1 mL) was reacted. After extraction, the crude product was purified by silica FC (PE/Et_2_O 7:3), delivering **28** as a pale-yellow oil (65 mg, 71% yield). **R_f_** 0.52 (PE/Et_2_O 7:3, basic KMnO_4_). **^1^H NMR** (400 MHz, CDCl_3_, 27 °C) δ 5.43 (bs, 1H, NH), 3.69–3.56 (m, 2H, OCH_2_), 2.40–2.26 (m, H, CH), 1.76 (ddt, *J* = 13.8, 9.3, 4.8 Hz, 1H, C*H*HCH), 1.60–1.49 (m, 1H, CH*H*CH), 1.34 (s, 9H, NH-C(C*H*_3_)_3_), 1.10 (d, *J* = 6.9 Hz, 3H, CHC*H*_3_), 0.90 (s, 9H, Si-C(CH_3_)_3_), 0.05 (s, 6H Si(CH_3_)_2_). **^13^C NMR** (101 MHz, CDCl_3_, 27 °C) δ 175.7 (C=O), 60.8 (OCH_2_), 51.1 (N-*C*(CH_3_)_3_), 38.0 (CH), 37.4 (*C*H_2_CH), 29.0 (N-C(*C*H_3_)_3_), 26.1 (Si-C(*C*H_3_)_3_), 18.4 (Si-*C*(CH_3_)_3_), 17.8 (CH*C*H_3_), -5.1 (Si-CH_3_), -5.2 (Si-CH_3_). **GC-MS**: R_t_ 8,035 min (Method A), *m*/*z* (%): 39 (6), 41 (27), 42 (5), 43 (4), 44 (4), 45 (6), 47 (5) 55 (10), 56 (11), 57 (33), 58 (16), 59 (12), 61 (4), 72 (3), 73 (52), 74 (89), 75 (42), 76 (6), 77 (3), 84 (3), 85 (4), 88 (4), 89 (5), 99 (3), 100 (12), 101 (6), 114 (7), 115 (9), 116 (4), 129 (19), 130 (3), 156 (5), 157 (4), 174 (100), 175 (13), 176 (4), 215 (9), 216 (5), 230 (33), 231 (7). **IR** ῡ (cm^−1^): 3300, 2961, 2930, 2858, 1643, 1552, 1462, 1389, 1361, 1256, 1227, 1089, 1024, 1005, 984, 938, 894, 834, 771, 684, 665. **HRMS** (ESI+): calcd. for C_15_H_34_NO_2_Si [M+H]^+^ 288.2353, found 288.2348.

**4-(*tert*-Butylamino)-3-methyl-4-oxobutyl benzoate (30).** Following the general procedure (condition B), the mixture of 3-iodobutyl benzoate **S28** (100 mg, 0.329 mmol), DMAP (2.4 mg, 0.0197 mmol), Pd(PPh_3_)_4_ (23 mg, 0.0197 mmol), Et_3_N (78 µL, 0.559 mmol), *t*-butyl isocyanide (82 µL, 0.723 mmol), and bidistilled H_2_O (237 µL, 13.2 mmol) in dry toluene (1.2 mL) was reacted. After extraction, the crude product was purified by silica FC (PE/Et_2_O 6:4), delivering **30** as a pale-yellow solid (67 mg, 72% yield). **R_f_** 0.27 (PE/Et_2_O 6:4, UV, CAM). **^1^H NMR** (400 MHz, CDCl_3_, 27 °C) δ 8.04 (dd, *J* = 8.3, 1.2 Hz, 2H, 2× *o*-CH_arom_), 7.57 (tt, *J* = 7.4, 1.3 Hz, 1H, *p*-CH_arom_), 7.45 (t, *J* = 7.8 Hz, 2H, 2× *m*-CH_arom_), 5.46 (s, 1H, NH), 4.46–4.25 (m, 2H, OCH_2_), 2.34–2.21 (m, 1H, C*H*CH_3_), 2.10 (ddt, *J* = 14.4, 8.9, 5.6 Hz, 1H, C*H*HCH), 1.83 (ddt, *J* = 11.6, 8.0, 5.8 Hz, 1H, CH*H*CH), 1.34 (s, 9H, 3×CH_3_), 1.18 (d, *J* = 6.8 Hz, 3H, CHCH_3_). **^13^C NMR** (101 MHz, CDCl_3_, 27 °C) δ 174.9 (OC=O), 166.8 (NC=O), 133.1 (*p*-CH_arom_), 129.7 (2×*o*-CH_arom_), 128.5 (2×*m*-CH_arom_), 63.2 (*C*(CH_3_)_3_), 51.3 (O*C*H_2_), 38.9 (*C*HCH_3_), 33.4 (*C*H_2_CH_2_CH), 28.9 (C(*C*H_3_)_3_), 18.3 (CH*C*H_3_). **GC-MS**: *t*_R_ = 9.940 min (Method A); *m*/*z* (%): 39 (4), 41 (21), 42 (7), 43 (3), 44 (4), 51 (8), 55 (9), 56 (31), 57 (37), 58 (39), 72 (4), 73 (13), 74 (3), 77 (33), 78 (3), 79 (4), 99 (4), 100 (43), 101 (3), 105 (100), 106 (8), 122 (3), 123 (22), 129 (25), 140 (7), 155 (3), 178 (3), 262 (5). **IR** ῡ (cm^−1^): 3383, 3276, 2971, 2924, 1699, 1665, 1599, 1584, 1518, 1451, 1389, 1362, 1318, 1280, 1224, 1202, 1178, 1141, 1116, 1102, 1087, 1072, 1032, 998, 978, 947, 909, 857, 812, 801, 714, 688, 675. **HRMS** (ESI+): calcd. for C_16_H_24_NO_3_ [M+H]^+^ 278.1751, found 278.1746.

## 4. Conclusions

Herein, we present a comprehensive study on the visible light-promoted three-component insertion of isocyanides into inactivated alkyl iodides. Experimental investigations were carried out to determine the optimal reaction conditions and elucidate the mechanism, revealing the formation of an alkyl radical intermediate through a photoinduced palladium-catalyzed step. Our protocol notably requires catalytic DMAP, which we propose plays a crucial role in accelerating and promoting the acylation step. The reaction scope was explored, leading to the synthesis of a diverse range of amides in good to high yields. Our data highlight the significant influence of steric hindrance in the reaction partners, with the most effective combination being secondary iodides and tertiary isocyanides. This mild, visible light-driven multicomponent reaction represents a novel approach in the field of palladium-catalyzed insertions, and we anticipate its broad applicability in organic synthesis.

## Figures and Tables

**Table 1:** First optimization of the insertion reaction ^1^.

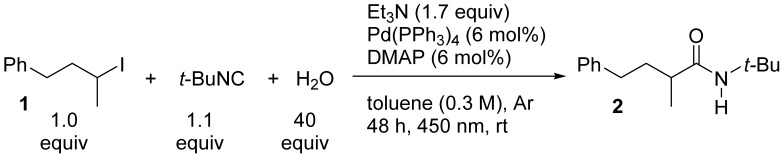		
Entry	Variation from Standard Conditions	Yield ^2^
1	none	72% (71%) ^3^
2	dark	0%
3	without Pd(PPh_3_)_4_	0%
4	without DMAP	2%
5	without Et_3_N	10%
6	without H_2_O	11%
7	without stirring	5%
8	DMSO	58%
9	DMF	50%
10	dioxane	0%
11	CH_3_CN	61%
12	0.05 M	0%
13	0.1 M	62%
14	0.6 M	72% ^3^
15	1 M	30% ^3^

^1^ 100 mg scale of iodide **1**. ^2^ The yields were determined by HPLC-UV using diphenyl ether as internal standard. ^3^ Isolated yields.

**Table 2:** Second optimization of the insertion reaction ^1^.

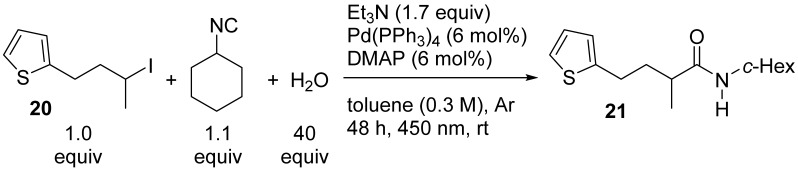			
Entry	Other Changes from Conditions of Table 1	Time	Yield ^2^
1	none	48 h	12%
2	none	72 h	28%
3	addition of Pd(PPh_3_)_4_ (6 mol%) after 48 h	72 h	60%
4	addition of *c*-HexNC (1.1 equiv) after 48 h	72 h	50%
5	*c*-HexNC (2.2 equiv)	48 h	58%
6	*c*-HexNC (2.2 equiv), 25 °C	48 h	64%

^1^ The optimal conditions of Table 1 are reported in the scheme above the table. 100 mg scale of iodide **20**. ^2^ Isolated yields.

## Data Availability

Data are contained within the article and Supplementary Materials.

## Supplementary Materials

The following supporting information can be downloaded at: https://www.mdpi.com/article/10.3390/molecules30122584/s1; additional data on the optimization (Tables S1 and S2, Figures S1 and S2), on-off experiments (page S5), the synthesis of non-commercial iodides and isocyanides (pages S5–S19), description of the vessel holder for the photochemical reaction (page S20), alternative mechanism pathway (Scheme S13), and the NMR copies (pages S21–S56).
