# Supplementary material for: Photoexcited Palladium Complex-Catalyzed Isocyanide Insertion into Inactivated Alkyl Iodides
Source: Molecules. 2025 Jun 13;30(12):2584. doi: 10.3390/molecules30122584 (PMC12196398; doi:10.3390/molecules30122584)

Photoexcited Palladium Complex-Catalyzed Isocyanide Insertion into Inactivated Alkyl Iodides

Andrea Messina, Filippo Monticelli, Tiziano Miroglio, Anna Gagliardi, Igor Viviani, Luca Banfi, Renata Riva, Lisa Moni, Andrea Basso and Chiara Lambruschini *

Department of Chemistry and Industrial Chemistry, University of Genova, Via Dodecaneso, 31, 16146 Genova, Italy.
* Correspondence: chiara.lambruschini@unige.it

SUPPORTING INFORMATIONS

Index

1 Full optimization data S2
2 On-off experiments S5
3 Procedures for iodides synthesis S5
3.1 General procedure for Appel reaction (iodide preparation) S5
3.2 Synthesis of (3-iodobutyl)benzene (1) S5
2.3 Synthesis of (3-iodopropyl)benzene (S3) S6
2.4 Synthesis of (3s,5s,7s)-1-iodoadamantane (S4) S6
2.5 Synthesis of (3-iodo-2,2-dimethylpropyl)benzene (S8) S7
2.6 Synthesis of 2-(3-iodobutyl)thiophene (20) S8
2.7 Synthesis of 1-(3-iodobutoxy)-4-methoxybenzene (S12) S9
2.8 Synthesis of *N*-(4-(3-iodobutyl)phenyl)acetamide (S17) S10
2.9 Synthesis of *tert*-butyl (4-(3-iodobutyl)phenyl)carbamate (S20) S12
2.10 Synthesis of 3-(3-iodobutyl)-1-methyl-1*H*-indole (S22) S14
2.11 Synthesis of *tert*-butyl(3-iodobutoxy)dimethylsilane (S26), 3-iodobutan-1-ol (S27) and 3-iodobutyl benzoate (S28) S15
2.12 Synthesis of (3-iodo-3-methylbutyl)benzene (S30) S17
3 Procedures for isocyanide synthesis S18
3.1 Synthesis of 1-isocyano-4-methylbenzene (S32) S18
4 Side products: ketoamides S18
5 Photochemical vessel holder S20
6 Mechanism: alternative pathway from III S20
7 NMR copies S21

1 Full optimization data

Table S1. First optimization of the insertion reaction. [1]

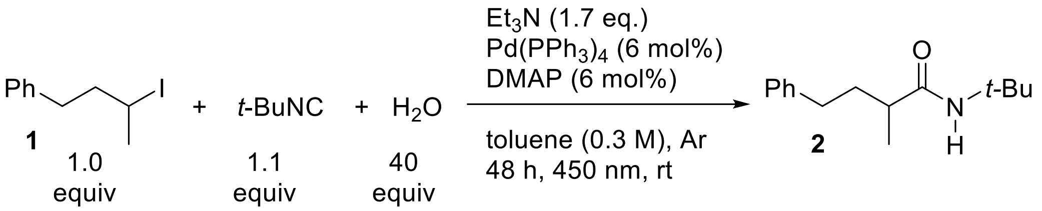

Entry	Variation from standard conditions	Yield [2]
1	0.1 M, 24 h	19%
2	0.1 M, 48 h	62%
3	0.1 M, 96 h	57%
4	0.1 M, DIPEA instead of Et_3N	52%
5	0.1 M, KOAc instead of Et_3N	18%
6	0.1 M, K_3PO_4 instead of Et_3N	11%
7	0.1 M, K_2CO_3 instead of Et_3N	15%
8	0.1 M, K_2HPO_4 instead of Et_3N	48%
9	0.1 M, Cs_2CO_3 instead of Et_3N	10%
10	0.1 M, Pyridine instead of Et_3N	25%
11	DMAP (30 mol%)	63%
12	$Pd(PPh_3)_2Cl_2$ instead of $Pd(PPh_3)_4$	
13	$Pd(OAc)_2$ (6 mol%) and dppf (12 mol%) instead of $Pd(PPh_3)_4$	-
14	$Pd(OAc)_2$ (6 mol%) and BippyPhos instead of $Pd(PPh_3)_4$	18%
15	$Pd(OAc)_2$ (6 mol%) and dppb instead of $Pd(PPh_3)_4$	-
16	$Pd(OAc)_2$ (6 mol%) and Xanthphos instead of $Pd(PPh_3)_4$	10%
17	$Pd(OAc)_2$ (6 mol%) and dppe instead of $Pd(PPh_3)_4$	-
18	$Pd(OAc)_2$ (6 mol%) and P(*n*-Bu)$_3$ instead of $Pd(PPh_3)_4$	-
19	$Pd(OAc)_2$ (6 mol%) and PPh_3 (30 mol%) instead of $Pd(PPh_3)_4$	45%
20	350 nm	28%
21	528 nm	14%
22	DCM	48%
23	Et_2O	28%

[1] 100 mg scale of iodide **1**. [2] The yields were determined by HPLC-UV using diphenyl ether as internal standard. [3] Isolated yields.

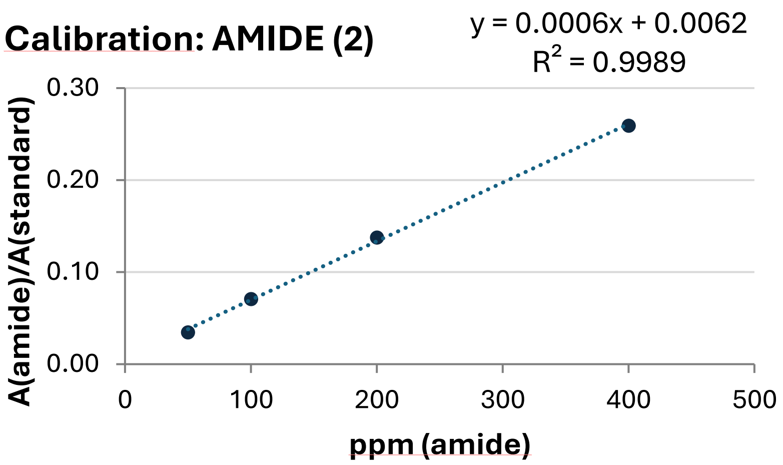

Figure S1. HPLC calibration curve of the amide **2** to determine the yield reported in table 1 and S1. A(amide) is the area of the amide determined by integration of the peak at t_R = 12.012 min and A(standard) is the area of the diphenyl ether determined by integration of the peak at t_R = 17.920 min. The chromatogram is reported in Figure S2.

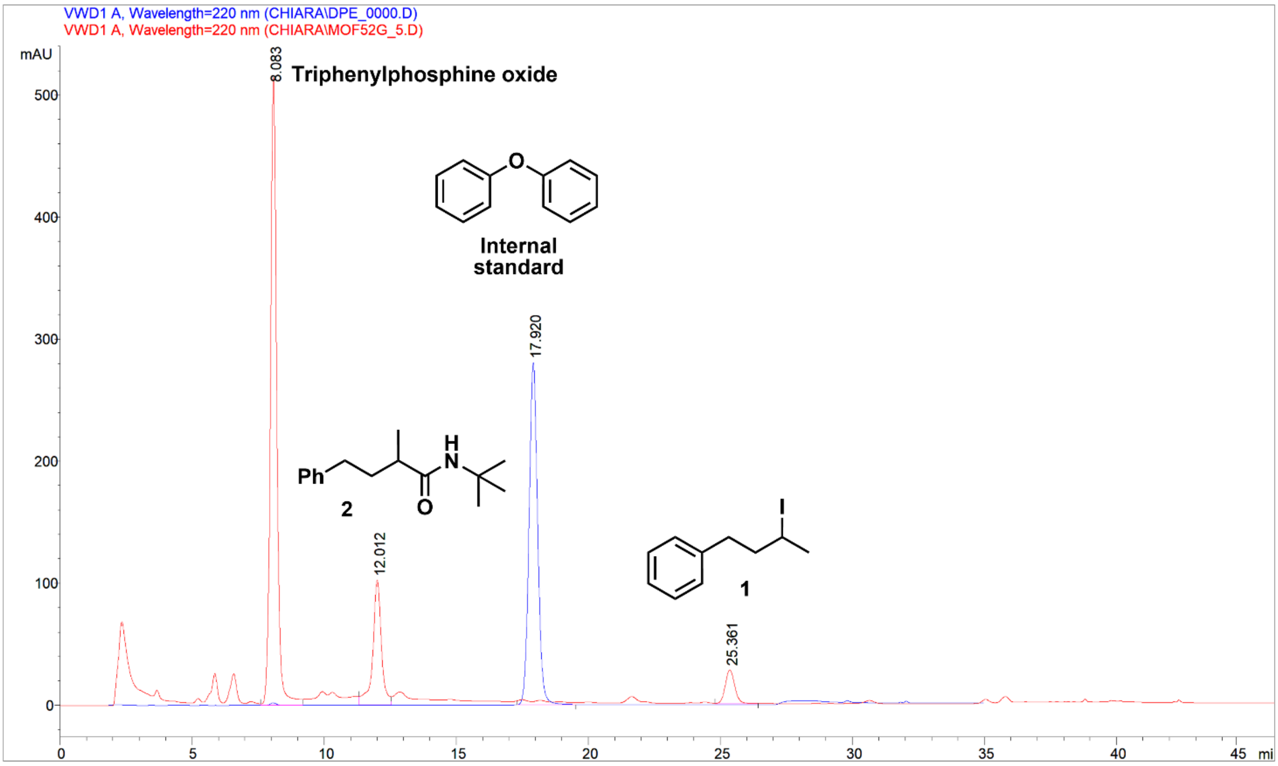

Figure S2. HPLC-UV of reaction crude (red) and of the internal standard diphenyl ether (blue).

Table S2. Second optimization of the insertion reaction. [1]

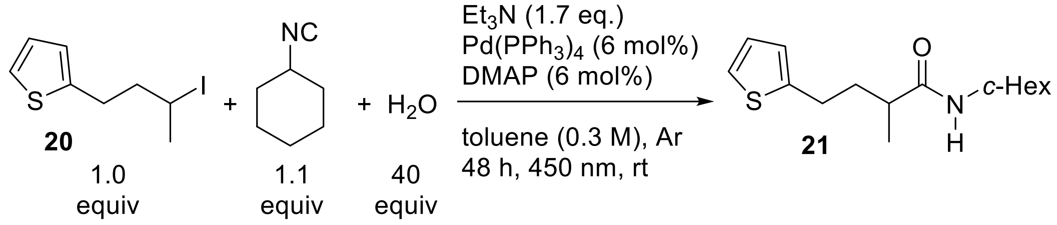

Entry	Others variation from conditions of table 1	Time	Yield [2]
1	25 °C	48 h	45%
2	35 °C	48 h	43%
3	addition of $Pd(PPh_3)_4$ (6 mol%), DMAP (6 mol%) and *c*-HexNC (1.1 equiv) after 48 h	72 h	62%
4	addition of $Pd(PPh_3)_4$ (6 mol%), DMAP (6 mol%) and *c*-HexNC (1.1 equiv) after 48 h	96 h	59%
5	addition of $Pd(PPh_3)_4$ (6 mol%), DMAP (6 mol%) and *c*-HexNC (1.1 equiv) after 48 h, 25 °C	72 h	52%
6	*c*-HexNC (2.2 equiv), 35 °C	48 h	62%
7	addition of PPh_3 (0.3 equiv) after 48 h. 25 °C	72 h	50 %
8	$Pd(OAc)_2$ (6 mol%) instead of $Pd(PPh_3)_4$, *c*-HexNC (2.2 equiv), 25 °C	48 h	45%
9	$Pd(PPh_3)_4$ (12 mol%), DMAP (12 mol%), 25 °C	48 h	18%
10	addition of *c*-HexNC (1.1 equiv) after 24 h, 25 °C	48 h	54%
11	$Pd(PPh_3)_4$ (3 mol%), 25 °C	72 h	45%

[1] The optimal conditions of table 1 are reported on the arrow. 100 mg scale of iodide **20**. [2] Isolated yields.

2 On-off experiments

A set of four reactions between iodide **1** (scale of 100 mg) and *t*-BuNC under the conditions of Entry 1 of Table 1 were set up in parallel following the general procedure (conditions A) reported in the manuscript. The vials were irradiated for 7 h and then one vial was worked up as described in the general procedure apart from the addition of 4-iodoanisole (1 equiv, internal standard) before the extraction. The reaction crude was checked by ^1H NMR and the yield was calculated by integration of signals attributed to the product and the internal standard. The remaining three vials were kept in the dark for 17 h. Thereafter one vial was worked up and checked by ^1H NMR and the remaining two vials were subjected to another cycle.

3 Procedures for iodides synthesis

Details on standard experimental procedures and instruments specifications are reported in the "Materials and Methods" section of the manuscript.

3.1 General procedure for Appel reaction (iodide preparation)

PPh_3 (1.2 equiv) and imidazole (1.2 equiv) were solubilized in DCM (1 mL/mmol of alcohol) under N_2 atmosphere. After 10 min, iodine (1.2 equiv) was slowly added. Alcohol (1 equiv) was then added by syringe as a solution in anhydrous DCM (1 mL/mmol of alcohol). After stirring for 16 h at rt, Et_2O was added, and the mixture was filtered to remove the precipitated imidazolium salts and triphenylphosphine oxide. The mother liquor was evaporated to dryness and the crude product was purified by flash chromatography on silica gel to give the desired product.

3.2 Synthesis of (3-iodobutyl)benzene (1)

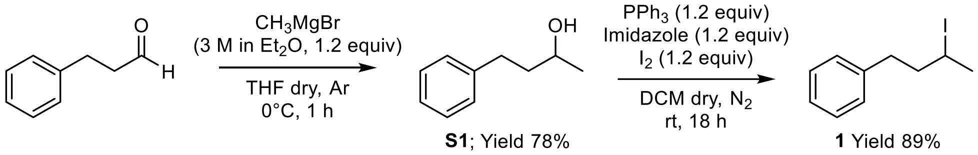

Scheme S1

4-Phenylbutan-2-ol (S1). In a two necked reaction flask the freshly distilled hydrocinnamaldehyde was added (1.00 g, 7.45 mmol, 0.98 mL). One neck was connected to a dropping funnel, the second neck was connected to the Ar line. Dry THF was added (15 mL) and the mixture was cooled to 0 °C. CH_3MgBr (3 M in Et_2O, 5 mL, 14.91 mmol) was slowly added in about 5 min. After 1 h at 0 °C, s.s. NH_4Cl was slowly added and the mixture was extracted with Et_2O, washed with brine, dried (Na_2SO_4), filtrated and evaporated to dryness. The crude product was purified by FC on silica (PE/Et_2O 6:4) to give the product as colourless oil (873 mg, 78% yield). **R_f** 0.28 (PE/Et_2O 65:35, UV and CAM). **^1H NMR** (400 MHz, $CDCl_3$, 27 °C) δ 7.31 – 7.26 (m, 2H, 2xCH_{arom}), 7.23 – 7.18 (m, 3H, 3xCH_{arom}), 3.83 (h, *J* = 6.2 Hz, 1H, CH), 2.82 – 2.61 (m, 2H, $PhCH_2$), 1.84 – 1.72 (m, 2H, CH_2CH), 1.23 (d, *J* = 6.2 Hz, 3H, CH_3). **^{13}C NMR** (101 MHz, $CDCl_3$, 27 °C) δ 142.2 (C_q), 128.5 (4xCH_{arom}), 126.0 (*p*-CH_{arom}), 67.7 (CH), 41.0 (CH_2CH), 32.3 ($PhCH_2$), 23.8 (CH_3). **GC-MS:** t_R = 4.92 min (Method A), *m/z* (%): 50 (5), 51 (14), 63 (6), 65 (19), 77 (18), 78 (31), 79 (11), 91 (100), 92 (48), 103 (6), 105 (9), 115 (7), 118 (8), 117 (84), 131 (6), 132 (30), 150 (6) $[M]^+$. Other data were in accordance with the literature.

(3-Iodobutyl)benzene (1). Following the general procedure for Appel reaction, the mixture of alcohol **S1** (3.50 g, 23.26 mmol), PPh_3 (7.32 g, 27.92 mmol), imidazole (1.90 g, 27.92 mmol) and I_2 (7.10 g, 27.92 mmol) in dry DCM (46 mL) was reacted. After workup, the crude product was purified by silica FC (PE/DCM 9:1) delivering the product as colourless oil (5.384 g, 89 % yield). **Rf** 0.75 (PE/DCM 9:1, UV (weak), CAM (weak) and 20 % H_2SO_4 in ethanol (weak)). **^1H NMR** (400 MHz, $CDCl_3$, 27 °C) δ 7.33 – 7.26 (m, 2H, 2xCH_{arom}), 7.23 – 7.17 (m, 3H, 3xCH_{arom}), 4.11 (dqd, *J* = 9.0, 6.8, 4.6 Hz, 1H, CH), 2.85 (ddd, *J* = 14.0, 9.0, 5.2 Hz, 1H, PhC*H*H), 2.69 (ddd, *J* = 13.8, 8.9, 7.0 Hz, 1H, PhCH*H*), 2.15 (dtd, *J* = 14.3, 9.0, 5.2 Hz, 1H, C*H*HCH), 1.95 (d, *J* = 6.8 Hz, 3H, CH_3), 1.88

(dddd, *J* = 14.5, 9.0, 7.0, 4.5 Hz, 1H, CH*H*CH). **^{13}C NMR** (101 MHz, $CDCl_3$, 27 °C) δ 140.9 ($C_{q.}$), 128.7 ($2xCH_{arom}$), 128.6 ($2xCH_{arom}$), 126.3 (*p*-CH_{arom}), 44.5 (CH_2CH), 36.0 ($PhCH_2$), 29.8 (CH), 29.1 (CH_3). **GC-MS:** t_R = 7.350 min (Method A); *m/z* (%): 39 (7), 41 (4), 51 (5), 63 (3), 65 (15), 77 (3), 89 (3), 91 (100), 92 (9), 115 (3), 117 (4), 133 (28), 134 (3). **IR** wavenumber (cm^{-1}): 3061, 3026, 2916, 2858, 1603, 1495, 1453, 1377, 1360, 1247, 1207, 1187, 1134, 1113, 1085, 1061, 1030, 994, 913, 893, 813, 745, 697, 606. Other data were in accordance with the literature.

2.3 Synthesis of (3-iodopropyl)benzene (S3)

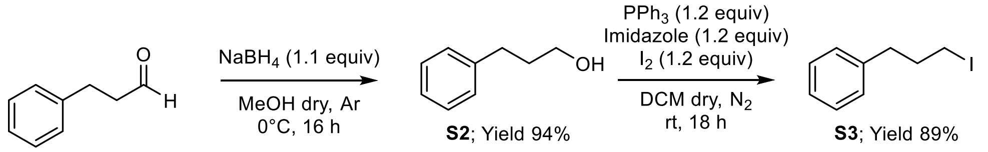

Scheme S2

3-Phenylpropan-1-ol (S2). Freshly distilled hydrocinnamaldehyde (1.010 g, 8.20 mmol) was solubilized in dry methanol (41 mL) under Ar atmosphere. The reaction was cooled to 0°C and $NaBH_4$ (341 mg, 9.02 mmol) was added portionwise. After 16 h at rt, 5% aq. solution of $(NH_4)H_2PO_4$ was added and the pH was adjusted to 3 by adding HCl 2N. The mixture was extracted with AcOEt, washed with brine, dried (Na_2SO_4), filtrated and evaporated to dryness. The crude product was purified by FC on silica (PE/Et_2O 6:4) to give the product as colourless oil (1.049 g, 94% yield). **Rf** 0.26 (PE/Et_2O 6:4, UV, CAM). **GC-MS** t_R = 5.745 min (Method A); *m/z* (Relative Intensity %): 39 (16), 41 (3), 50 (6), 51 (19), 52 (5), 63 (8), 65 (25), 77 (25), 78 (16), 79 (17), 89 (5), 91 (96), 92 (48), 93 (4), 103 (14), 104 (5), 105 (13), 115 (8), 117 (100), 118 (63), 119 (6), 136 (22) $[M]^+$. **IR** wavenumber (cm^{-1}): 3305, 3062, 3027, 2935, 2863, 1720, 1669, 1603, 1541, 1515, 1496, 1453, 1412, 1373, 1318, 1275, 1178, 1123, 1058, 1030, 919, 827, 744, 714, 699. Other data were in accordance with the literature.

(3-Iodopropyl)benzene (S3). Following the general procedure for Appel reaction, the mixture of alcohol **S2** (1.048 g, 7.70 mmol), PPh_3 (3.014 g, 9.24 mmol), imidazole (0.629 g, 9.24 mmol) and I_2 (2.345 g, 9.24 mmol) in dry DCM (15 mL) was reacted. After workup, the crude product was purified by silica FC (PE/DCM 9:1) delivering the product as colourless oil (1.686 g, 89 % yield). **Rf** 0.45 (PE/DCM 9:1, UV, CAM). **^{1}H NMR** (400 MHz, $CDCl_3$, 27 °C) δ 7.31 – 7.24 (m, 2H, $2xCH_{arom}$), 7.22 – 7.16 (m, 3H, $3xCH_{arom}$), 3.14 (t, *J* = 6.9 Hz, 2H, CH_2I), 2.71 (t, *J* = 7.3 Hz, 2H, $PhCH_2$), 2.10 (p, *J* = 6.6 Hz, 2H, $CH_2CH_2CH_2$). **^{13}C NMR** (101 MHz, $CDCl_3$, 27 °C) δ 140.5 ($C_{q.}$), 128.6 ($2xCH_{arom}$), 128.6 ($2xCH_{arom}$), 126.2 (*p*-CH_{arom}), 36.3 ($PhCH_2$), 34.9 ($CH_2CH_2CH_2$), 6.6 (CH_2I). **GC-MS** t_R = 7.090 min (Method A); *m/z* (Relative Intensity %): 39 (10), 41 (7), 50 (3), 51 (6), 63 (5), 65 (17), 77 (4), 89 (4), 91 (100), 92 (8), 115 (5), 117 (5), 119 (15), 127 (3), 246 (14) $[M]^+$. **IR** wavenumber (cm^{-1}): 3061, 3025, 2933, 2853, 1603, 1495, 1453, 1424, 1348, 1263, 1212, 1165, 1073, 1029, 955, 907, 851, 792, 739, 696, 614. Other data were in accordance with the literature.

2.4 Synthesis of (3s,5s,7s)-1-iodoadamantane (S4)

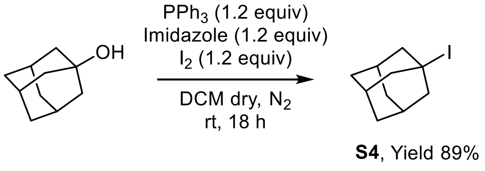

Scheme S3

Following the general procedure for Appel reaction, the mixture of 1-adamantol (1.92 g, 12.64 mmol), PPh_3 (3.98 g, 15.17 mmol), imidazole (1.03 g, 15.17 mmol) and I_2 (3.85 g, 15.17 mmol) in dry DCM (25 mL) was reacted. After workup, the crude product was purified by silica FC (PE) delivering the product as white solid (2.153 g, 65 % yield). Rf 0.37 (PE/DCM 9:1, 20 % H_2SO_4 in EtOH, CAM (weak)). **^{1}H NMR** (300 MHz, $CDCl_3$, 27 °C) δ 2.63 (d, *J* = 2.9 Hz, 6H, $3xCH_2$), 2.01 – 1.89 (m, 3H, 3xCH), 1.84 – 1.78 (m, 6H, $3xCH_2$). **^{13}C NMR** (101 MHz, $CDCl_3$, 27 °C) δ 52.6 ($3xCH_2$), 35.7 ($3xCH_2$), 33.2 (3xCH). **GC-MS:** t_R = 6.470 min (Method A); *m/z* (relative intensity %): 39 (9), 41 (9), 55 (6), 67 (11), 77 (10), 79 (30), 81 (7), 91 (9), 107 (7), 93 (19), 135 (100), 136 (10), 262 $[M]^+$. **IR** wavenumber (cm^{-1}): 2905, 2850, 1470, 1450, 1364, 1341, 1310, 1274, 1178, 1100, 1019, 979, 941, 887, 792, 760, 663. Other data were in accordance with the literature.

2.5 Synthesis of (3-iodo-2,2-dimethylpropyl)benzene (S8)

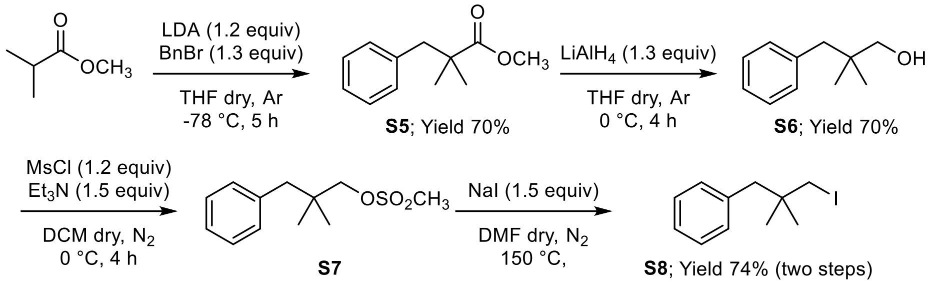

Scheme S4

Methyl 2,2-dimethyl-3-phenylpropanoate (S5). Methyl isobutyrate (1.303 g, 12.76 mmol) was solubilized in dry THF (8.5 mL) under Ar atmosphere. In another flask *i*Pr_2NH (2.70 mL, 19.13 mmol) was solubilized in dry THF (34 mL) under Ar atmosphere, the solution was cooled to -78 °C and *n*-BuLi (2.5 M in hexane, 17.86 mmol, 11.1 mL) was added dropwise. After 15 min, the ester solution was added to the LDA (lithium diisopropyl amide) solution dropwise. After 1 h BnBr (1.97 mL,16.58 mmol) was added dropwise to the reaction mixture. After 4 h at -78 °C, the mixture was warmed to 0 °C, s.s. NH_4Cl was slowly added and then it was warmed to rt. The mixture was extracted with Et_2O, washed with brine, dried (Na_2SO_4), filtrated and evaporated to dryness. The crude product was purified by FC on silica (PE/DCM 9:1) to give the product as yellowish liquid (1.717 g, 70% yield). **Rf** 0.52 (PE/DCM 9:1, UV, CAM). **^{1}H NMR** (300 MHz, $CDCl_3$, 27 °C) δ 7.30 – 7.17 (m, 3H, 3xCH_{arom}), 7.14 – 7.05 (m, 2H, 2xCH_{arom}), 3.66 (s, 3H, OCH_3), 2.85 (s, 2H, $PhCH_2$), 1.18 (s, 6H, 2xCH_3). **^{13}C NMR** (100 MHz, $CDCl_3$, 27 °C) δ 177.90 (C=O), 137.85 ($C_{q.,arom}$), 130.06 (2xCH_{arom}), 127.96 (2xCH_{arom}), 126.42 (*p*-CH_{arom}), 51.65 (*C*(CH_3)$_2$), 46.35 (OCH_3), 43.63 ($PhCH_2$), 24.91 (2xCH_3). **HRMS** (ESI+): calcd. for $C_{12}H_{17}O_2$ $[M+H]^+$ 193.1223, found 193.1219.

2,2-Dimethyl-3-phenylpropan-1-ol (S6). $LiAlH_4$ (340 mg, 8.95 mmol) was suspended in dry THF (60 mL) at 0 °C under Ar atmosphere. After, a solution of **S5** (1.721 g, 8.95 mmol) in dry THF (25 mL) was added dropwise. 5 mL of dry THF were used to wash the flask containing **S5** to transfer it quantitatively. After 4 h at 0 °C, a saturated aqueous solution of Rochelle salts was added slowly and the mixture was stirred for 1 h allowing the flask to warm up. The mixture was extracted with Et_2O, washed with brine, dried (Na_2SO_4), filtrated and evaporated to dryness. The crude product was purified by FC on silica (PE/AcOEt 9:1) to give the product as colourless oil (1.249 g, 85% yield). **Rf** 0.37 (PE/AcOEt 9:1, UV, CAM). **^{1}H NMR** (300 MHz, $CDCl_3$, 27 °C) δ 7.31 – 7.25 (m, 1H, *p*-CH_{arom}), 7.24 – 7.12 (m, 4H, 4xCH_{arom}), 3.35 – 3.28 (m, 2H, OCH_2), 2.58 (s, 2H, $PhCH_2$), 2.17 (s,1H, OH), 0.89 (s, 6H, 2xCH_3). **^{13}C NMR** (100 MHz, $CDCl_3$, 27 °C) δ 138.90 ($C_{q.\ arom}$), 130.65 (2xCH_{arom}), 128.02 (2xCH_{arom}), 126.15 (*p*-CH_{arom}), 71.3 (OCH_2), 44.90 (*C*(CH_3)$_2$), 36.6 ($PhCH_2$), 24.10 (2xCH_3). **GC-MS**: t_R = 6.540 min (Method A); *m/z* (Relative Intensity %): 39 (12), 41 (6), 43 (9), 45 (6), 51 (6), 55 (34), 57 (4), 63 (4), 65 (16), 72 (15), 73 (19), 77 (5), 78 (3), 89 (3), 91 (78), 92 (100), 93 (8), 105 (4), 115 (6), 117 (6), 131 (4), 133 (4), 164 (10) $[M]^{+\cdot}$. **IR** wavenumber (cm^{-1}): 3232, 3025, 2958, 2932, 2866, 1602, 1581, 1493, 1467, 1451, 1386, 1361, 1292, 1248, 1198, 1181, 1154, 1123, 1071, 1062, 1042, 1020, 994, 943, 922, 908, 897, 831, 776, 719, 700, 617. Other data were in accordance with the literature.

2,2-Dimethyl-3-phenylpropyl methanesulfonate (S7). The alcohol **S6** (1.120 g, 6.821 mmol) was solubilized in dry DCM (68 mL) under N_2 atmosphere. Et_3N (1.71 mL, 12.28 mmol) was added, and the reaction flask was cooled to 0 °C and after 5 min MsCl (0.634 mL, 8.186 mmol) was introduced in the reaction mixture. After 4 h at 0 °C, HCl 1 M was added and the mixture was extracted with Et_2O, washed with NaOH 0.1 M, brine, dried (Na_2SO_4), filtrated and evaporated to dryness. The crude methansulfonate (a yellowish oil) was directly used in the sequent reaction. **Rf** 0.16 (PE/Et_2O 7:3, UV, CAM). **^{1}H NMR** (400 MHz, $CDCl_3$, 27°C) δ 7.32 – 7.27 (m, 2H, 2xCH_{arom}), 7.26 – 7.21 (m, 1H, *p*-CH_{arom}), 7.17 – 7.12 (m, 2H, 2xCH_{arom}), 3.88 (s, 2H, OCH_2), 3.02 (s, 3H, SO_2CH_3), 2.62 (s, 2H, $PhCH_2$), 0.98 (s, 6H, 2xCH_3).

(3-Iodo-2,2-dimethylpropyl)benzene (S8). The methansulfonate **S7** (6.821 mmol) was solubilized in dry DMF (17 mL) under N_2 atmosphere. NaI (5.11g, 34.11 mmol) was added. After 18 h at 150 °C, a 5% LiCl aq. solution was added, and the mixture was extracted with Et_2O, washed with brine, dried (Na_2SO_4), filtrated and evaporated to dryness. The crude product was purified by FC on silica (PE/DCM 9:1) to give the product as

colourless oil (1.384 g, 74% yield over 2 steps). **Rf** 0.60 (PE/DCM 9:1, UV, CAM). **^{1}H NMR** (400 MHz, $CDCl_3$, 27 °C) δ 7.31 – 7.27 (m, 2H, 2xCH_{arom}), 7.25 – 7.17 (m, 3H, 3xCH_{arom}), 3.13 (s, 2H, CH_2I), 2.65 (s, 2H, $PhCH_2$), 1.04 (s, 6H, 2xCH_3). **^{13}C NMR** (100 MHz, $CDCl_3$, 27 °C) δ 138.32 (1C, $C_{q.\ arom}$), 130.41 (2xCH_{arom}), 128.10 (2xCH_{arom}), 126.45 (*p*-CH_{arom}), 46.42 ($PhCH_2$), 34.84 (*C*(CH_3)$_2$), 27.20 (2xCH_3), 24.28 (CH_2I). **GC-MS**: t_R = 7.760 min (Method A); *m/z* (Relative Intensity %): 39 (9), 41 (10), 51 (4), 55 (8), 56 (7), 63 (3), 65 (14), 89 (3), 91 (100), 92 (9), 105 (13), 115 (3), 117 (3), 147 (6), 218 (6), 274 (3) $[M]^{+}$. **IR** wavenumber (cm^{-1}): 3027, 2960, 2926, 1601, 1494, 1467, 1453, 1420, 1383, 1365, 1323, 1244, 1212, 1182, 1165, 1129, 1086, 1031, 909, 878, 813, 773, 721, 700, 605. **HRMS** (ESI+): calcd. for $C_{11}H_{16}I$ $[M+H]^{+}$ 275.0291, found 275.0295.

2.6 Synthesis of 2-(3-iodobutyl)thiophene (20)

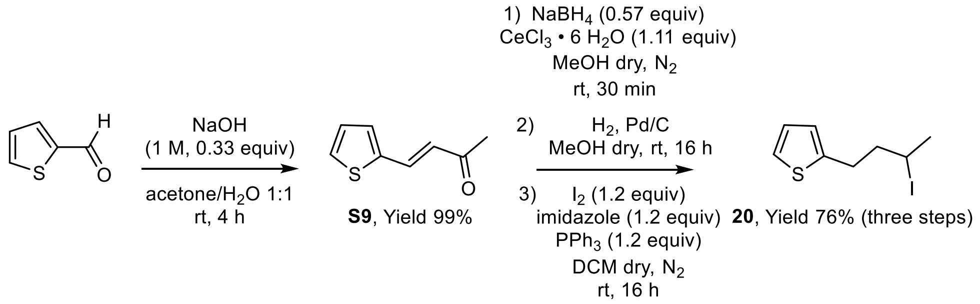

Scheme S5

(*E*)-4-(Thiophen-2-yl)but-3-en-2-one (S9). Thiophene-2-carbaldehyde (1.38 mL, 15.16 mmol) was solubilized in acetone/H_2O (1:1, 19 mL), then 1 M NaOH (5.0 mL, 5.00 mmol) was slowly added. After 4 h at rt, 1 M HCl was added until pH ~6: The mixture was extracted with AcOEt, washed with brine, dried (Na_2SO_4), filtrated and evaporated to dryness. The crude product was purified by FC on silica (PE/Et_2O 8:2) to give the product as a bright-yellow oil (753 mg, 99% yield). **Rf** 0.40 (PE/Et_2O 8:2, UV, CAM). **^{1}H NMR** (400 MHz, $CDCl_3$, 27 °C) δ 7.64 (d, *J* = 15.9 Hz, 1H, C*H*=CHCO), 7.43 – 7.39 (m, 1H, CH_{arom}), 7.31 – 7.29 (m, 1H, CH_{arom}), 7.08 (dd, *J* = 5.1, 3.6 Hz, 1H, 4-CH_{arom}), 6.54 (d, *J* = 15.9 Hz, 1H, CH=C*H*CO), 2.35 (s, 3H, CH_3). **^{13}C NMR** (101 MHz, $CDCl_3$, 27 °C) δ 197.9 (C=O), 139.9 ($C_{q.\ arom}$), 135.9 (*C*H=CHCO), 131.7 (CH_{arom}), 129.1 (CH_{arom}), 128.4 (4-CH_{arom}), 126.0 (CH=*C*HCO), 27.8 (CH_3). **GC-MS**: t_R = 6.815 min (Method A); *m/z* (Relative Intensity %): 38 (6), 39 (27), 43 (33), 45 (20), 50 (7), 51 (10), 58 (7), 63 (10), 65 (41), 69 (15), 108 (7), 109 (74), 110 (6), 137 (100), 138 (9), 139 (5), 151 (9), 152 (51) $[M]^{+}$, 153 (5). **IR** wavenumber (cm^{-1}): 3103, 3006, 1684, 1661, 1591, 1514, 1422, 1356, 1305, 1270, 1251, 1225, 1198, 1167, 1096, 1079, 1045, 1000, 961, 904, 854, 816, 702, 632. Other data were in accordance with the literature.

2-(3-Iodobutyl)thiophene (20). STEP 1. Ketone **S9** (1.616 g, 10.6 mmol) was solubilized in dry methanol (53 mL) under N_2 atmosphere. $CeCl_3$·6 H_2O (4.40 g, 11.8 mmol) was added, then the reaction flask was cooled to 0 °C and $NaBH_4$ (229 mg, 6.05 mmol) was added portion-wise. The bright-yellow mixture turned pale pink over 30 min. The solvent was removed by rotavapor, and the residue was extracted with H_2O and Et_2O. The organic phases were washed with brine, dried (Na_2SO_4), filtrated and evaporated to dryness. The crude *α*,β-unsaturated alcohol (1.531 g, pale yellow oil) was sufficiently pure to be used in the next step without further purification. **Rf** 0.35 (PE/Et_2O 7:3, UV, CAM). **^{1}H NMR** (400 MHz, $CDCl_3$, 27 °C) δ 7.20 – 7.12 (m, 1H, CH_{arom}), 6.99 – 6.92 (m, 2H, 2xCH_{arom}), 6.71 (d, *J* = 15.7 Hz, 1H, C*H*=CHCH), 6.11 (dd, *J* = 15.7, 6.3 Hz, 1H, CH=C*H*CH), 4.53 – 4.37 (m, 1H, CH=CHC*H*), 1.55 (bd, *J* = 3.9 Hz, 1H, OH), 1.36 (d, *J* = 6.4 Hz, 3H, CH_3). **GC-MS**: t_R = 6.525 min (Method A); *m/z* (Relative Intensity %): 38 (4), 39 (28), 41 (6), 43 (48), 44 (3), 45 (37), 50 (8), 51 (14), 52 (4), 53 (10), 55 (55), 57 (4), 58 (10), 59 (5), 62 (4), 63 (10), 65 (20), 66 (7), 67 (12), 69 (14), 70 (6), 71 (5), 74 (3), 77 (24), 78 (8), 79 (3), 82 (3), 84 (26), 85 (8), 91 (21), 95 (4), 97 (100), 98 (7), 99 (4), 103 (4), 108 (4), 109 (11), 110 (9), 111 (85), 112 (7), 113 (4), 121 (6), 134 (5), 135 (27), 136 (15), 137 (8), 139 (12), 154 (35) $[M]^{+}$, 155 (3). STEP 2. The crude *α*,β-unsaturated alcohol (1.531 g, 9.93 mmol) was solubilized in dry MeOH/AcOEt (2:1, 99 mL) under N_2 atmosphere, then 10 % wt. Pd/C (0.383 g, 25% wt. respect to crude alcohol) was added. The inert atmosphere was replaced by H_2, and after 18 h the mixture was filtered through celite using AcOEt for washing and evaporated to dryness. The crude alcohol (1.551 g, pale yellow oil) was sufficiently pure to be used in the next step without further purification. **Rf** 0.33 (PE/Et_2O 7:3, UV, CAM). **^{1}H NMR** (400 MHz, $CDCl_3$, 27 °C) δ 7.12

(dd, J = 5.1, 1.2 Hz, 1H, 5-CH_{arom}), 6.92 (dd, J = 5.1, 3.4 Hz, 1H, 4-CH), 6.83 – 6.80 (m, 1H, 3-**C**H_{arom}), 3.87 (dt, J = 11.1, 6.1 Hz, 1H, C*H*OH), 3.04 – 2.86 (m, 2H, C*H*$_2$$CH_2$CH), 1.88 – 1.78 (m, 2H, C*H*$_2$CH), 1.41 (s, 1H, OH), 1.24 (d, J = 6.2 Hz, 3H, CH_3). **GC-MS**: t_R = 6.050 min (Method A); *m/z* (Relative Intensity %): 39 (18), 41 (5), 43 (15), 45 (52), 50 (3), 51 (6), 53 (15), 57 (4), 58 (6), 59 (3), 63 (3), 65 (5), 66 (3), 67 (5), 69 (6), 71 (5), 77 (10), 78 (4), 79 (6), 84 (13), 85 (8), 97 (100), 98 (41), 99 (7), 105 (3), 109 (3), 110 (5), 111 (11), 123 (79), 124 (7), 125 (4), 137 (6), 138 (20), 139 (2), 156 (17) $[M]^+$. STEP 3. Following the general procedure for Appel reaction, the mixture of the crude alcohol (9.32 mmol), PPh_3 (2.935 g, 11.2 mmol), imidazole (762 mg, 11.2 mmol) and I_2 (110 mg, 11.2 mmol) in dry DCM (19 mL) was reacted. After workup, the crude product was purified by silica FC (PE/DCM 9:1) delivering the product as a pale-yellow oil (1.884 g, 76 % yield three steps). **Rf** 0.6 (PE/DCM 9:1, UV, CAM). **^{1}H NMR** (400 MHz, $CDCl_3$, 27 °C) δ 7.14 (dd, J = 5.1, 1.2 Hz, 1H, 5-CH_{arom}), 6.93 (dd, J = 5.1, 3.4 Hz, 1H, 4-CH_{arom}), 6.84 (dd, J = 3.4, 0.9 Hz, 1H, 3-CH_{arom}), 4.14 (dqd, J = 9.4, 6.8, 4.4 Hz, 1H, CHI), 3.06 (ddd, J = 14.2, 8.3, 5.2 Hz, 1H, C*H*HCH_2CH), 2.94 (dt, J = 15.3, 7.8 Hz, 1H, CH*H*CH_2CH), 2.24 – 2.13 (m, 1H, C*H*HCH), 1.99 – 1.86 (m, 4H, CH*H*CH + CH_3). **^{13}C NMR** (101 MHz, $CDCl_3$, 27 °C) δ 143.3 (2-$C_{q.\ arom}$), 127.0 (4-CH_{arom}), 124.9 (3-CH_{arom}), 123.5 (5-CH_{arom}), 44.5 (CH_2CH), 30.3 (CH_2CH_2CH), 29.1 (CHI), 29.1 (CH_3). **GC-MS**: t_R = 7.350 min (Method A); *m/z* (Relative Intensity): 39 (8), 41 (3), 45 (13), 53 (10), 69 (3), 97 (100), 98 (7), 99 (5), 123 (3), 139 (12), 266 (9) $[M]^+$. **IR** wavenumber (cm^{-1}): 2914, 1535, 1440, 1377, 1295, 1242, 1197, 1165, 1130, 1080, 1037, 995, 902, 848, 822, 800, 768, 744, 690, 609. **HRMS** (ESI+): calcd. for $C_8H_{12}IS$ $[M+H]^+$ 266.9699, found 266.9695.

2.7 Synthesis of 1-(3-iodobutoxy)-4-methoxybenzene (S12)

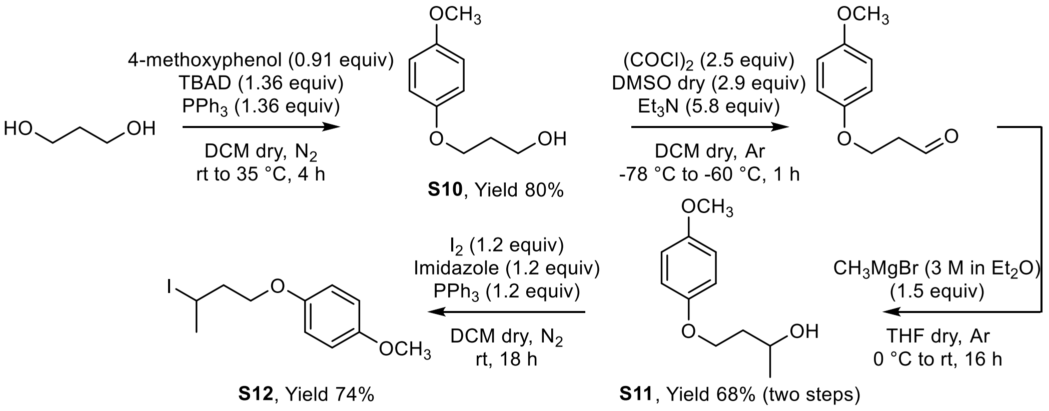

Scheme S6

3-(4-Methoxyphenoxy)propan-1-ol (S10). 4-Methoxyphenol (0.375 g, 3.02 mmol) was solubilized in dry DCM (30 mL) under N_2 atmosphere. Then 1,3-propanediol (0.241 mL, 3.33 mmol), PPh_3 (1.341 g, 4.11 mmol) and TBAD (946 mg, 4.11 mmol) were added to the reaction flask. After 4 h at 35 °C, the mixture was evaporated to dryness and the crude product was purified by silica FC (PE/AcOEt 7:3 to 1:1) delivering the product as a white solid (440 mg, 80% yield) **Rf** 0.35 (PE/AcOEt 7:3, UV, CAM). **M.P.** 55.0 – 57.5 °C. **^{1}H NMR** (400 MHz, $CDCl_3$, 27°C) δ 6.87 – 6.80 (m, 4H, 4xCH_{arom}), 4.08 (t, J = 5.9 Hz, 2H, OC*H*$_2$$CH_2$), 3.86 (bt, J = 5.4 Hz, 2H, C*H*$_2$OH), 3.77 (s, 3H, CH_3), 2.02 (p, J = 5.9 Hz, 2H, CH_2C*H*$_2$$CH_2$), 1.92 (s, 1H, OH). **^{13}C NMR** (101 MHz, $CDCl_3$, 27°C) δ 154.1 (*C*-OCH_3), 153.0 (*C*-OCH_2), 115.6 (2xCH_{arom}), 114.8 (2xCH_{arom}), 66.8 (OCH_2CH_2), 60.9 (CH_2OH), 55.9 (CH_3), 32.2 ($CH_2CH_2CH_2$). **GC-MS**: t_R = 8.125 min (Method A); *m/z* (Relative Intensity %): 39 (8), 41 (12), 52 (6), 53 (8), 63 (6), 64 (5), 65 (8), 81 (15), 95 (11), 109 (75), 110 (5), 123 (11), 124 (100), 125 (8), 182 (28) $[M]^+$. **IR** wavenumber (cm^{-1}): 3266, 3016, 2954, 2935, 2870, 2838, 2480, 2059, 1984, 1871, 1640, 1508, 1469, 1440, 1390, 1292, 1225, 1179, 1115, 1057, 1031, 946, 898, 825, 778, 720. Other data were in accordance with the literature.

4-(4-Methoxyphenoxy)butan-2-ol (S11). STEP 1. dry DMSO (499.0 μL, 7.03 mmol) was solubilized in dry DCM (16 mL) under inert Ar atmosphere in a 2-neck flask. The mixture was cooled to -78 °C and after 5 min $(COCl)_2$ (1.43 M in dry DCM, 4.24 mL, 6.06 mmol) was added. After 10 min a solution of the alcohol **S10** (441.9 mg, 2.43 mmol) in dry DCM (6 mL) was added dropwise. 3 mL of dry THF were used to wash the flask containing **S10** to transfer it quantitatively. After 15 min Et_3N (1.2 mL, 14.07 mmol) was added dropwise. After 1 h at -60 °C, a 5% aq. solution of $(NH_4)H_2PO_4$ was added while the reaction flask was let warm to rt. 2 M HCl was added until pH 5, then the mixture was extracted with Et_2O. The organic phases were washed with brine, dried (Na_2SO_4), filtrated and evaporated to dryness. The crude aldehyde (438 mg, yellowish oil) was sufficiently pure

to be used in the next step without further purification. **Rf** 0.71 (PE/AcOEt 1:1, UV, CAM). **^{1}H NMR** (400 MHz, $CDCl_3$, 27°C) δ 9.87 (t, *J* = 1.6 Hz, 1H, CHO), 6.90 – 6.80 (m, 4H, 4xCH_{arom}), 4.27 (t, *J* = 6.1 Hz, 2H, OCH_2), 3.77 (s, 3H, CH_3), 2.88 (td, *J* = 6.1, 1.6 Hz, 2H, C*H*$_2$CHO). **GC-MS**: t_R = 7.750 min (Method A), *m*/*z* (Relative Intensity %): 38 (7), 39 (20), 41 (21), 50 (6), 51 (10), 52 (18), 53 (25), 54 (12), 55 (10), 56 (5), 63 (14), 64 (12), 65 (17), 77 (9), 80 (7), 81 (35), 82 (5), 92 (6), 95 (35), 96 (5), 109 (100), 110 (7), 123 (56), 124 (85), 125 (7), 180 (46) $[M]^+$, 181 (6). STEP 2. The aldehyde (2.43 mmol) was solubilized in dry THF (12 mL) under Ar atmosphere. The reaction flask was cooled to 0 °C and CH_3MgBr (3 M in Et_2O, 1.21 mL, 3.64 mmol) was slowly added. After 16 h at rt, s.s. NH_4Cl was slowly added, and the mixture was extracted with Et_2O. The organic phases were washed with brine, dried (Na_2SO_4), filtrated and evaporated to dryness. The crude product was purified by silica FC (PE/AcOEt 7:3) to give the product as a white solid (324 mg, 68% yield two step). **Rf** 0.28 (PE/AcOEt 7:3, UV, CAM). **M.P.** 59.6 – 61.6 °C. **^{1}H NMR** (400 MHz, $CDCl_3$) δ 6.88 – 6.81 (m, 4H, 4xCH_{arom}), 4.18 – 4.01 (m, 3H, OCH_2 + CH), 3.77 (s, 3H, OCH_3), 2.21 – 2.15 (m, 1H, OH), 1.91 (q, *J* = 5.9 Hz, 2H, C*H*$_2$CH), 1.27 (d, *J* = 6.2 Hz, 3H, CHC*H*$_3$). **^{13}C NMR** (101 MHz, $CDCl_3$) δ 154.1 (CH_3O-$C_{q.\ arom}$), 152.9 (CH_2O-$C_{q.\ arom}$), 115.6 (2xCH_{arom}), 114.7 (2xCH_{arom}), 66.8 (OCH_2), 66.6 (CH), 55.8 (OCH_3), 38.2 (CH_2CH), 23.7 ($CHCH_3$). **GC-MS**: t_R = 8.250 min (Method A); *m*/*z* (Relative Intensity %): 39 (5), 41 (7), 43 (10), 45 (24), 53 (6), 55 (8), 65 (5), 81 (9), 95 (7), 109 (50), 123 (6), 124 (100), 125 (8), 196 (16) $[M]^+$. **IR** wavenumber (cm^{-1}): 3373, 2936, 1509, 1465, 1441, 1420, 1391, 1373, 1294, 1231, 1180, 1146, 1112, 1051, 1034, 997, 979, 945, 913, 828, 787, 727. **HRMS** (ESI+): calcd. for $C_{11}H_{17}O_3$ $[M+H]^+$ 197.1172, found 197.1179.

1-(3-Iodobutoxy)-4-methoxybenzene (S12). Following the general procedure for Appel reaction, the mixture of **S11** (230 mg, 1.17 mmol), PPh_3 (0.458 g, 1.40 mmol), imidazole (95 mg, 1.40 mmol) and I_2 (355 mg, 1.40 mmol) in dry DCM (2.4 mL) was reacted. After workup, the crude product was purified by silica FC (PE/DCM 1:1) delivering the product as a pale-yellow oil (272 mg, 74 % yield). **Rf** 0.56 (PE/DCM 1:1, UV, CAM). **^{1}H NMR** (400 MHz, $CDCl_3$, 27°C) δ 6.84 (m, *J* = 7.0 Hz, 4H, 4xCH_{arom}), 4.44 (dqd, *J* = 9.6, 6.6, 4.7 Hz, 1H, CH), 4.07 (dt, *J* = 10.3, 5.3 Hz, 1H, OC*H*H), 3.99 (td, *J* = 8.7, 5.1 Hz, 1H, OCH*H*), 3.77 (s, 3H, OCH_3), 2.22 (ddt, *J* = 14.6, 9.7, 5.0 Hz, 1H, C*H*HCH), 2.12 – 2.03 (m, 1H, CH*H*CH), 2.01 (d, *J* = 6.9 Hz, 3H, CHC*H*$_3$). **^{13}C NMR** (101 MHz, $CDCl_3$, 27°C) δ 154.1 (CH_3O-$C_{q.\ arom}$), 153.0 (CH_2O-$C_{q.\ arom}$), 115.7 (2xCH_{arom}), 114.8 (2xCH_{arom}), 68.4 (OCH_2), 55.9 (OCH_3), 42.3 (CH_2CH), 29.2 ($CHCH_3$), 25.8 (CH). **GC-MS**: t_R = 9.260 min (Method A); *m*/*z* (Relative Intensity %): 39 (20), 41 (29), 51 (6), 52 (9), 53 (12), 54 (8), 55 (39), 56 (8), 63 (9), 64 (11), 65 (9), 77 (17), 79 (5), 81 (10), 92 (10), 95 (17), 107 (13), 109 (54), 123 (28), 124 (100), 125 (10), 137 (27), 306 (19). **IR** wavenumber (cm^{-1}): 2913, 2832, 1592, 1505, 1465, 1441, 1378, 1290, 1225, 1162, 1127, 1105, 1035, 991, 910, 881, 822, 737, 710, 637. **HRMS** (ESI+): calcd. for $C_{11}H_{16}IO_3$ $[M+H]^+$ 307.0190, found 307.0187.

2.8 Synthesis of *N*-(4-(3-iodobutyl)phenyl)acetamide (S17)

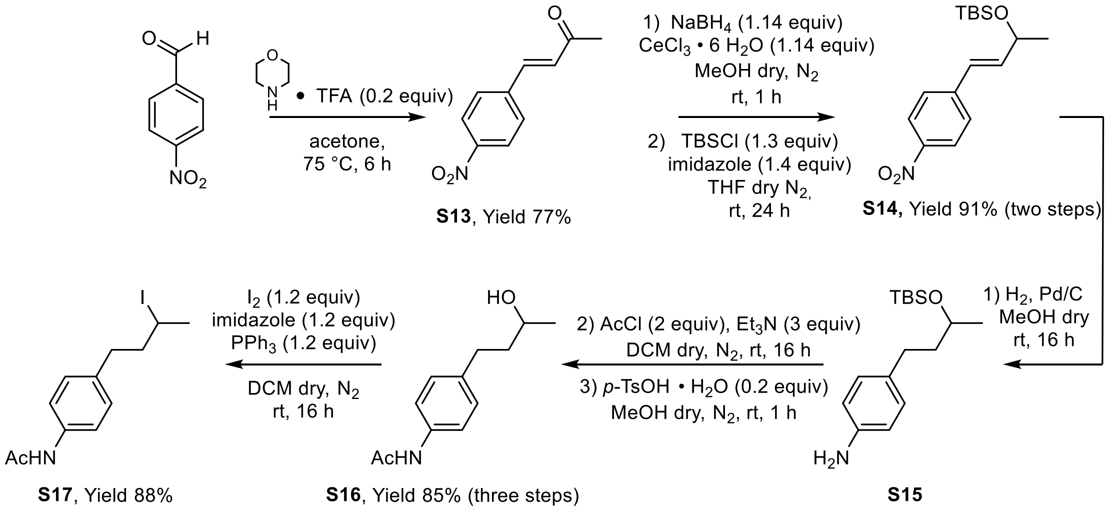

Scheme S7

(*E*)-4-(4-nitrophenyl)but-3-en-2-one (S13). The compound was prepared following the procedure published by K. Zumbansen *et al.* (K. Zumbansen, A. Döhring, B. List, *Adv. Synth. Catal.* **2010**, 352, 1135–1138). 4-Nitrobenzaldehyde (1.004 g, 6.64 mmol) was solubilized in acetone (16.6 mL). After morfolinium 2,2,2-

trifluoroacetate (0.2672 g, 1.33 mmol) was added and the reaction flask was sealed and heated at 75 °C for 6 h. Then s.s. $NaHCO_3$ was slowly poured into the flask, and the mixture was extracted with AcOEt. The organic phases were washed with brine, dried (Na_2SO_4), filtrated and evaporated to dryness. The crude product was purified by silica FC (PE/AcOEt 8:2) to give the product as a yellow solid (977 mg, 77% yield). **Rf** 0.26 (PE/AcOEt 8:2, UV, CAM). **^{1}H NMR** (400 MHz, $CDCl_3$, 27°C) δ 8.27 (d, *J* = 8.5 Hz, 2H, 2x*m*-CH_{arom}), 7.70 (d, *J* = 8.8 Hz, 2H, 2x*o*-CH_{arom}), 7.54 (d, *J* = 16.3 Hz, 1H, C*H*=CH(C=O)), 6.82 (d, *J* = 16.3 Hz, 1H, CH=C*H*(C=O)), 2.43 (s, 3H, CH_3). **^{13}C NMR** (101 MHz, $CDCl_3$, 27°C) δ 197.7 (C=O), 148.7 (C-NO_2), 140.8 (*C*-CH=CH), 140.2 (*C*H=CH(C=O)), 130.5 (CH=CH(C=O)), 129.0 (2x*o*-CH_{arom}), 124.4 (2x*m*-**C**H_{arom}), 28.2 (CH_3). **GC-MS**: t_R = 9.150 min (Method A); *m/z* (Relative Intensity %): 39 (7), 43 (100), 44 (12), 45 (7), 50 (5), 51 (10), 63 (9), 65 (5), 76 (7), 77 (17), 78 (9), 91 (9), 102 (7), 103 (14), 104 (10), 115 (6), 121 (5), 128 (6), 131 (13), 132 (12), 133 (7), 134 (6), 151 (12), 178 (7). **IR** wavenumber (cm^{-1}): 3112, 2838, 1687, 1666, 1626, 1614, 1595, 1512, 1414, 1342, 1296, 1257, 1207, 1188, 1177, 1110, 1009, 975, 955, 910, 861, 827, 743, 707, 684, 669, 631. Other data were in accordance with the literature.

(*E*)-*tert*-Butyldimethyl((4-(4-nitrophenyl)but-3-en-2-yl)oxy)silane (S14). STEP 1. Ketone **S13** (956 mg, 5.0 mmol) was solubilized in dry methanol (25.0 mL) under N_2 atmosphere, then $CeCl_3{\cdot}6\,H_2O$ (2.021 g, 5.70 mmol) was added. The reaction flask was cooled to 0 °C and $NaBH_4$ (215 mg, 5.70 mmol) was added portion-wise. After 1 h s.s. NH_4Cl was slowly added, and the mixture was extracted with AcOEt. The organic phases were washed with brine, dried (Na_2SO_4), filtrated and evaporated to dryness. The crude secondary alcohol (966 mg, pale yellow solid) was used in the next reaction without further purification. **Rf** 0.21 (PE/AcOEt 7:3, UV, CAM). **^{1}H NMR** (400 MHz, $CDCl_3$, 27°C) δ 8.19 (d, *J* = 8.3 Hz, 2H, 2x*m*-CH_{arom}), 7.51 (d, *J* = 8.4 Hz, 2H, 2x*o*-CH_{arom}), 6.67 (d, *J* = 15.9 Hz, 1H, C*H*=CHCH), 6.46 (dd, *J* = 15.8, 5.7 Hz, 1H, CH=C*H*CH), 4.61 – 4.48 (m, 1H, OCH), 1.63 (d, *J* = 4.0 Hz, 1H, OH), 1.41 (d, *J* = 6.5 Hz, 3H, CH_3). STEP 2. The crude of the secondary alcohol (1.3456 g, 6.96 mmol) was solubilized in dry THF (14 mL) under Ar atmosphere. Imidazole (0.6631 g, 9.75 mmol) was added portion-wise, then *tert*-butyldimethylsilyl chloride (9.05 mmol, 1.3646 g) was added to the solution. After 7 h an addition of imidazole (4.88 mmol) and TBSCl (9.05 mmol) was made to reach completion. The solvent was removed using the rotavapor and the crude mixture was extracted with water and Et_2O. The organic phases were washed with brine, dried (Na_2SO_4), filtrated and evaporated to dryness. The crude product was purified by silica FC (PE/Et_2O 100:3) delivering the product as yellow oil (1.947 g, 91% yield two steps). **Rf** 0.25 (PE/Et2O 100:3, UV, CAM). **^{1}H NMR** (400 MHz, $CDCl_3$, 27°C) δ 8.20 – 8.11 (m, 2H, 2x*m*-CH_{arom}), 7.51 – 7.44 (m, 2H, 2x*o*-CH_{arom}), 6.60 (dd, *J* = 15.8, 1.4 Hz, 1H, C*H*=CHCH), 6.40 (dd, *J* = 15.8, 4.9 Hz, 1H, CH=C*H*CH), 4.50 (qdd, *J* = 6.4, 4.9, 1.4 Hz, 1H, OCH), 1.31 (d, *J* = 6.4 Hz, 3H, CHC*H*3), 0.92 (s, 9H, $SiC(CH_3)_3$), 0.09 (s, 3H, $SiCH_3$), 0.08 (s, 3H, $SiCH_3$). **^{13}C NMR** (101 MHz, $CDCl_3$, 27°C) δ 146.8 (C-NO_2), 143.9 (*C*-CH=CH), 139.7 (CH=CHCH), 127.0 (2x*o*-CH_{arom}), 126.0 (CH=CHCH), 124.1 (2x*m*-CH_{arom}), 68.9 (OCH), 26.0 ($SiC(CH_3)_3$), 24.4 ($CHCH_3$), 18.4 ($SiC(CH_3)_3$), -4.6 ($SiCH_3$), -4.6 ($SiCH_3$). **GC-MS**: t_R = 10,350 min (Method A); *m/z* (Relative Intensity %): 41 (5), 43 (3), 45 (4), 47 (5), 57 (5), 59 (3), 73 (17), 75 (100), 76 (8), 77 (5), 115 (9), 127 (3), 128 (7), 129 (9), 130 (13), 250 (21), 251 (4), 307 (2) $[M]^+$. **IR** wavenumber (cm^{-1}): 2959, 2929, 2888, 2857, 1597, 1519, 1343, 1252, 1148, 1089, 970, 834, 777. **HRMS** (ESI+): calcd. for $C_{16}H_{26}NO_3Si$ $[M+H]^+$ 308.1676, found 308.1680.

***N*-(4-(3-hydroxybutyl)phenyl)acetamide (S16).** STEP 1. The alcohol **S14** (1.934 g, 6.30 mmol) was solubilized in dry MeOH/AcOEt (2:1, 63 mL) under N_2 atmosphere, then 10 % wt. Pd/C (0.968 g, 50% wt. respect to **S14**) was added. The inert atmosphere was replaced by H_2, and after 18 h the mixture was filtered through celite using AcOEt for washing and evaporated to dryness. The crude aniline **S15** (1.761 g, pinkish oil) was sufficiently pure to be used in the next step without further purification. **Rf** 0.69 (PE/AcOEt 7:3, UV, CAM). **^{1}H NMR** (400 MHz, $CDCl_3$, 27°C) δ 7.01 – 6.94 (m, 2H, 2xCH_{arom}), 6.65 – 6.60 (m, 2H, 2xCH_{arom}), 3.82 (h, *J* = 6.1 Hz, 1H, OCH), 3.54 (bs, 2H, NH_2), 2.60 (ddd, *J* = 13.8, 10.6, 5.9 Hz, 1H, C*H*HCH_2CH), 2.47 (ddd, *J* = 13.8, 10.5, 5.9 Hz, 1H, CH*H*CH_2CH), 1.77 – 1.59 (m, 2H, C*H*2CH), 1.15 (d, *J* = 6.1 Hz, 3H, CHC*H*3), 0.90 (s, 9H, ($SiC(CH_3)_3$)), 0.06 (s, 3H, $Si(CH_3)$)), 0.05 (s, 3H, $Si(CH_3)$)). STEP 2. The crude aniline **S15** (6.30 mmol) was solubilized in dry DCM (31.5 mL) under N_2 atmosphere, then Et_3N (2.63 mL, 18.90 mmol) was added. The reaction flask was cooled to 0 °C and AcCl (8.95 mL, 12.60 mmol) was added dropwise. After 16 h at rt, the volume of solvent was reduced using a rotavapor, s.s. NH_4Cl was added, and the mixture was extracted with Et_2O. The organic phases were washed with brine, dried (Na_2SO_4), filtrated and evaporated to dryness. The crude acetamide (2.026 g, pale-yellow oil) was sufficiently pure to be used in the next step without further purification. **Rf** 0.10

(PE/AcOEt 8:2, UV, CAM). **^{1}H NMR** (400 MHz, $CDCl_3$, 27°C) δ 7.42 – 7.36 (m, 2H, 2xCH$_{arom}$), 7.16 – 7.10 (m, 2H, 2xCH$_{arom}$), 3.83 (h, *J* = 6.1 Hz, 1H, OCH), 2.67 (ddd, *J* = 15.5, 10.4, 5.8 Hz, 1H, C*H*HCH$_2$CH), 2.55 (ddd, *J* = 13.5, 10.3, 6.0 Hz, 1H, CH*H*CH$_2$CH), 2.17 (s, 3H, CH$_3$-C=O), 1.80 – 1.65 (m, 2H, C*H$_2$*CH), 1.16 (d, *J* = 6.1 Hz, 3H, CHC*H$_3$*), 0.90 (s, 9H, (SiC(CH$_3$)$_3$)), 0.06 (s, 3H, Si(CH$_3$)), 0.05 (s, 3H, Si(CH$_3$)). STEP 3. The crude acetamide (6.30 mmol) was solubilized in dry MeOH (31.5 mL) under N_2 atmosphere, then *p*TsOH·H_2O (240 mg, 1.26 mmol) was slowly added. After 1 h, the solvent was removed using the rotavapor and the crude mixture was extracted with s.s. $NaHCO_3$ and Et_2O. The organic phases were washed with brine, dried (Na_2SO_4), filtrated and evaporated to dryness. The crude product was purified by silica FC (PE/AcOEt 1:5, AcOEt/MeOH 9:1) delivering the product as a pale-yellow solid (1.110 g, 85% yield three steps). **Rf** 0.23 (PE/AcOEt 1:5, UV, CAM). **M.P.** 133.4 – 135.2 °C. **^{1}H NMR** (400 MHz, $CDCl_3$, 27 °C) δ 7.43 – 7.37 (m, 2H, 2xCH$_{arom}$), 7.29 (bs, 1H, NH), 7.16 – 7.12 (m, 2H, 2xCH$_{arom}$), 3.87 – 3.75 (m, 1H, OCH), 2.78 – 2.58 (m, 2H, C*H$_2$*CH$_2$CH[l]), 2.16 (s, 3H, CH$_3$-CO), 1.78 – 1.70 (m, 2H, C*H$_2$*CH), 1.68 (s, 1H, OH), 1.22 (d, *J* = 6.2 Hz, 3H, CHC*H$_3$*). **^{13}C NMR** (101 MHz, $CDCl_3$, 27 °C) δ 168.4 (C=O), 138.3 (C$_{q.\ arom}$), 135.8 (C$_{q.\ arom}$), 129.0 (2xCH$_{arom}$), 120.3 (2xCH$_{arom}$), 67.5 (OCH), 40.9 (*C*H$_2$CH), 31.6 (*C*H$_2$CH$_2$CH), 24.7 (*C*H$_3$-CO), 23.8 (CH*C*H$_3$). **GC-MS**: t_R = 9.945 min (Method A); *m/z* (Relative Intensity %): 39 (5), 41 (3), 42 (3), 43 (36), 44 (4), 45 (14), 51 (4), 52 (3), 65 (6), 77 (12), 78 (10), 79 (6), 91 (8), 92 (3), 93 (8), 94 (4), 103 (3), 104 (3), 105 (4), 106 (100), 107 (13), 108 (3), 117 (3), 118 (5), 119 (6), 120 (10), 132 (36), 133 (5), 136 (13), 146 (7), 147 (27), 148 (11), 149 (4), 162 (4), 165 (3), 174 (3), 189 (10), 207 (24) $[M]^+$. **IR** wavenumber (cm^{-1}): 3238, 3182, 3120, 3069, 2971, 2935, 2854, 1657, 1603, 1545, 1515, 1454, 1411, 1369, 1322, 1279, 1265, 1182, 1133, 1113, 1095, 1067, 1023, 961, 944, 864, 824, 792, 759, 720, 653, 632, 607. **HRMS** (ESI+): calcd. for $C_{12}H_{18}NO_2$ $[M+H]^+$ 208.1332, found 208.1336.

***N*-(4-(3-iodobutyl)phenyl)acetamide (S17).** Following the general procedure for Appel reaction, the mixture of **S15** (1.109 g, 5.35 mmol), PPh_3 (2.095 g, 6.42 mmol), imidazole (437 mg, 6.42 mmol) and I_2 (1.629 g, 6.42 mmol) in dry DCM (11 mL) was reacted. After workup, the crude product was purified by silica FC (PE/DCM 1:1) delivering the product as a pale-yellow solid (1.493 g, 88 % yield). **Rf** 0.67 (PE/AcOEt 1:5, UV, CAM). **M.P.** 138.2 – 139.8 °C. **^{1}H NMR** (400 MHz, $CDCl_3$, 27 °C) δ 7.43 – 7.38 (m, 2H, 2xCH$_{arom}$), 7.25 (s, 1H, NH), 7.19 – 7.11 (m, 2H, 2xCH$_{arom}$), 4.08 (dqd, *J* = 9.2, 6.8, 4.5 Hz, 1H, ICH), 2.81 (ddd, *J* = 13.9, 8.8, 5.1 Hz, 1H, C*H*HCH$_2$CH[l]), 2.66 (ddd, *J* = 13.9, 8.6, 7.3 Hz, 1H, CH*H*CH$_2$CH), 2.17 (s, 3H, CH$_3$-CO), 2.15 – 2.06 (m, 1H, C*H*HCH), 1.94 (d, *J* = 6.8 Hz, 3H, CHC*H$_3$*), 1.84 (dddd, *J* = 14.6, 8.8, 7.1, 4.4 Hz, 1H, CH*H*CH). **^{13}C NMR** (101 MHz, $CDCl_3$, 27°C) δ 168.4 (C=O), 136.9 (C$_{q.\ arom}$), 136.1 (C$_{q.\ ,arom}$), 129.2 (2xCH$_{arom}$), 120.3 (2xCH$_{arom}$), 44.4 (*C*H$_2$CH [2'l]), 35.4 (*C*H$_2$CH$_2$CH), 29.8 (ICH), 29.1(CH*C*H$_3$), 24.7 (*C*H$_3$-CO). **GC-MS**: t_R = 10.885 min (Method A); *m/z* (Relative Intensity %): 39 (5), 41 (5), 43 (25), 51 (3), 52 (4), 65 (3), 77 (10), 78 (13), 79 (5), 91 (4), 104 (3), 105 (6), 106 (100), 107 (10), 132 (6), 148 (45), 149 (5), 190 (8), 317 (13) $[M]^+$. **IR** wavenumber (cm^{-1}): 3247, 3185, 3118, 3061, 2958, 2918, 2857, 1907, 1658, 1602, 1548, 1511, 1441, 1409, 1369, 1318, 1266, 1250, 1203, 1186, 1136, 1117, 1071, 1042, 1014, 968, 944, 899, 835, 807, 763, 726, 652, 608. **HRMS** (ESI+): calcd. for $C_{12}H_{17}INO$ $[M+H]^+$ 318.0349, found 318.0342.

2.9 Synthesis of *tert*-butyl (4-(3-iodobutyl)phenyl)carbamate (S20)

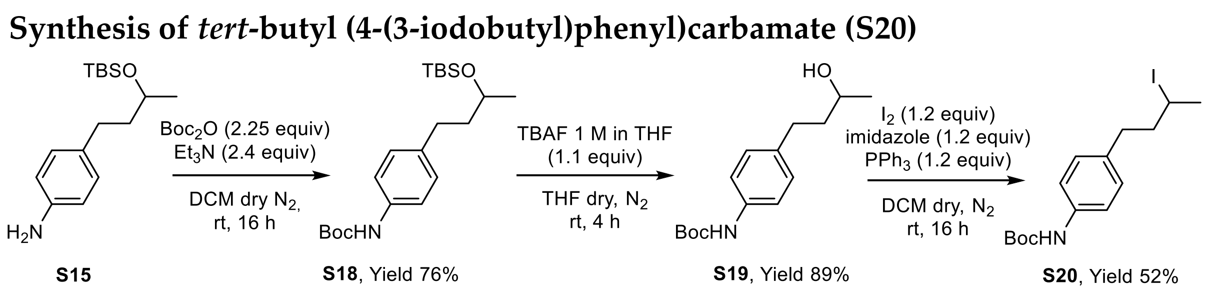

Scheme S8

***tert*-Butyl (4-(3-((tert-butyldimethylsilyl)oxy)butyl)phenyl)carbamate (S18).** The aniline **S15** (533 mg, 1.91 mmol) was solubilized in dry DCM (10 mL) under N_2 atmosphere, then Et_3N (639 μL, 4.58 mmol) was added. The reaction flask is cooled to 0 °C and Boc_2O (938 mg, 4.30 mmol) was added dropwise. After 16 h, the solvent was reduced using a rotavapor and the crude mixture was extracted with AcOEt and water. The organic phases were washed with brine, dried (Na_2SO_4), filtrated and evaporated to dryness. The crude product was purified by silica FC (PE/Et_2O 9:1 and PE/Et_2O 1:1) delivering the product as yellow oil (551 mg, 76 %). **Rf** 0.5 (PE/Et_2O

9:1, UV, CAM). **^{1}H NMR** (400 MHz, $CDCl_3$, 27°C) δ 7.25 – 7.23 (m, 2H, 2xCH_{arom} *ortho* to NH), 7.13 – 7.07 (m, 2H, 2xCH_{arom} *meta* to NH), 6.39 (s, 1H, NH), 3.82 (h, *J* = 6.3 Hz, 1H, OCH[l]), 2.65 (ddd, *J* = 13.9, 10.4, 5.7 Hz, 1H, C*H*HCH_2CH), 2.53 (ddd, *J* = 13.6, 10.4, 6.2 Hz, 1H, CH*H*CH_2CH), 1.81 – 1.59 (m, 2H, C*H*$_2$CH), 1.51 (s, 9H, (OC(CH_3)$_3$)), 1.16 (d, *J* = 6.1 Hz, 3H, CH_3), 0.90 (s, 9H, SiC(CH_3)$_3$), 0.05 (s, 3H, SiCH_3), 0.05 (s, 3H, SiCH_3). **^{13}C NMR** (101 MHz, $CDCl_3$, 27°C) δ 164.7 (C=O), 137.6 (C-$C_{q.\ arom}$), 136.1 (N-$C_{q.\ arom}$), 128.9 (2xCH_{arom} *meta* to NH), 118.8 (2xCH_{arom} *ortho* to NH),80.5 (OC(CH_3)$_3$), 68.3 (OCH), 41.7 (CH_2CH), 31.6 (CH_2CH_2CH), 28.5 (OC(CH_3)$_3$), 26.1 (SiC(CH_3)$_3$), 23.9 (CHCH_3), 18.3 (SiC(CH_3)$_3$), -4.1 (SiCH_3), -4.5 (SiCH_3). **GC-MS:** t_R = 10.995 min (very broad peak) (Method A); *m/z* (Relative Intensity %): 39 (17), 40 (6), 41 (30), 42 (3), 43 (4), 44 (50), 45 (9), 47 (7), 55 (5), 56 (14), 57 (6), 59 (8), 60 (3), 61 (3), 73 (22), 74 (3), 75 (46), 76 (5), 77 (9), 78 (4), 79 (3), 91 (3), 103 (100), 104 (11), 105 (5), 106 (47), 107 (7), 115 (3), 118 (3), 119 (4), 120 (10), 130 (3), 131 (3), 132 (23), 133 (3), 145 (3), 146 (5), 147 (13), 148 (3), 150 (3), 178 (15), 179 (3), 180 (3), 207 (12), 208 (3), 209 (3), 222 (40), 223 (8), 248 (4), 279 (6). **IR** wavenumber (cm^{-1}): 3334, 2956, 2929, 2857, 1729, 1702, 1595, 1521, 1472, 1462, 1411, 1392, 1367, 1314, 1248, 1158, 1134, 1073, 1052, 1028, 1017, 990, 939, 903, 832, 809, 772, 709, 661. **HRMS** (ESI+): calcd. For $C_{21}H_{38}NO_3Si$ $[M+H]^+$ 380.2615, found 380.2610.

***tert*-Butyl (4-(3-hydroxybutyl)phenyl)carbamate (S19).** The silyl ether **S18** (255 mg, 0.700 mmol) was solubilized in dry THF (1.4 mL) under N_2 atmosphere, then TBAF (1 M in THF, 1.54 mL, 1.54 mmol) was added. After 4 h, the crude mixture was diluted with water and extracted with AcOEt. The organic phases were washed with brine, dried (Na_2SO_4), filtrated and evaporated to dryness. The crude product was purified by silica FC (PE/AcOEt 7:3) delivering the product as pale-yellow oil (165 mg, 89% yield). **Rf** 0.29 (PE/AcOEt 7:3, UV, CAM). **^{1}H NMR** (400 MHz, $CDCl_3$, 27°C) δ 7.29 – 7.23 (m, 2H, 2xCH_{arom} *ortho* to NH), 7.15 – 7.09 (m, 2H, 2xCH_{arom} *meta* to NH), 6.42 (bs, 1H, NH), 3.88 – 3.74 (m, 1H, OCH), 2.76 – 2.55 (m, 2H, C*H*$_2CH_2$CH), 1.81 – 1.66 (m, 2H, C*H*$_2$CH), 1.51 (s, 9H, OC(CH_3)$_3$), 1.33 (bs, 1H, OH), 1.22 (d, *J* = 6.2 Hz, 3H, CHC*H*$_3$). **^{13}C NMR** (101 MHz, $CDCl_3$, 27°C) δ 153.0 (C=O), 136.9 (C-$C_{q.\ arom}$), 136.3 (N-$C_{q.\ arom}$), 129.0 (2xCH_{arom} *meta* to NH), 118.9 (2xCH_{arom} *ortho* to NH), 80.7 (OC(CH_3)$_3$), 67.6 (OCH), 41.0 (CH_2CH), 31.5 (CH_2CH_2CH), 28.5 (OC(CH_3)$_3$), 23.8 (CHCH_3). **GC-MS**: t_R = 10.325 min (very broad peak) (Method A); *m/z* (Relative Intensity %): 39 (13), 40 (3), 41 (42), 42 (3), 43 (7), 44 (9), 45 (14), 51 (3), 52 (3), 55 (5), 56 (8), 57 (100), 58 (5), 59 (14), 65 (4), 77 (8), 78 (8), 79 (3), 91 (7), 93 (4), 103 (3), 104 (3), 105 (4), 106 (60), 107 (8), 118 (3), 119 (3), 120 (4), 130 (4), 131 (3), 132 (25), 133 (3), 138 (5), 146 (5), 147 (8), 148 (3), 150 (7), 151 (3), 158 (5), 165 (8), 176 (10), 191 (20), 192 (3), 209 (27), 210 (4), 265 (7) $[M]^{+}$. **IR** wavenumber (cm^{-1}): 3317, 2964, 1697, 1597, 1521, 1453, 1412, 1392, 1367, 1314, 1258, 1157, 1052, 1012, 863, 792, 702. **HRMS** (ESI+): calcd. for $C_{15}H_{24}NO_3$ $[M+H]^+$ 266.1751, found 266.1755.

***tert*-Butyl (4-(3-iodobutyl)phenyl)carbamate (S20).** Following the general procedure for Appel reaction, the mixture of **S19** (191 mg, 0.72 mmol), PPh_3 (282 mg, 0.864 mmol), imidazole (588 mg, 0.864 mmol) and I_2 (219 mg, 0.864 mmol) in dry DCM (1.5 mL) was reacted. After workup, the crude product was purified by silica FC (PE/DCM 9:1) delivering the product as a pale-yellow solid (140 mg, 52 % yield). **Rf** 0.40 (PE/Et_2O 9:1, UV, CAM). **^{1}H NMR** (400 MHz, $CDCl_3$, 27°C) δ 7.31 – 7.21 (m, 2H, 2xCH_{arom} *ortho* to NH), 7.13 (d, *J* = 8.5 Hz, 2H, 2xCH_{arom} *meta* to NH), 6.41 (bs, 1H, NH), 4.08 (dqd, *J* = 9.0, 7.0, 4.5 Hz, 1H, ICH), 2.79 (ddd, *J* = 13.9, 8.7, 5.1 Hz, 1H, C*H*HCH_2CH), 2.69 – 2.58 (m, 1H, CH*H*CH_2CH), 2.11 (dtd, *J* = 14.3, 8.9, 5.2 Hz, 1H, C*H*HCH), 1.93 (d, *J* = 6.8 Hz, 3H, CHC*H*$_3$), 1.82 (dddd, *J* = 14.6, 8.8, 7.2, 4.5 Hz, 1H, CH*H*CH), 1.51 (s, 9H, OC(CH_3)$_3$). **^{13}C NMR** (101 MHz, $CDCl_3$, 27°C) δ 152.9 (C=O), 136.5 (N-$C_{q.\ arom}$), 135.5 (C-$C_{q.\ arom}$), 129.1 (2xCH_{arom} *meta* to NH), 118.9 (2xCH_{arom} *ortho* to NH), 80.7 (OC(CH_3)$_3$), 44.5 (CH_2CH), 35.2 (CH_2CH_2CH), 29.8 (ICH), 29.1 (CHCH_3), 28.4 (OC(CH_3)$_3$). **GC-MS**: t_R = 7.710 min (Method A); *m/z* (Relative Intensity %): 39 (13), 41 (12), 51 (4), 53 (6), 55 (6), 65 (6), 66 (3), 67 (12), 69 (3), 77 (17), 78 (5), 79 (37), 80 (3), 81 (9), 91 (16), 92 (6), 93 (27), 94 (3), 105 (3), 107 (14), 127 (3), 135 (100), 136 (11). **IR** wavenumber (cm^{-1}): 3309, 3013, 2985, 2969, 2932, 1686, 1594, 1525, 1479, 1444, 1411, 1394, 1379, 1368, 1312, 1240, 1216, 1152, 1119, 1095, 1083, 1054, 1028, 1016, 938, 906, 827, 800, 774, 763, 738, 682. **HRMS** (ESI+): calcd. for $C_{15}H_{23}INO_2$ $[M+H]^+$ 376.0768, found 376.0771.

2.10 Synthesis of 3-(3-iodobutyl)-1-methyl-1*H*-indole (S22)

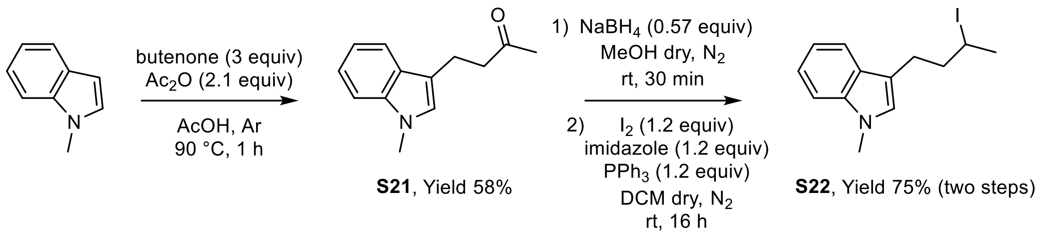

Scheme S9

4-(1-Methyl-1*H*-indol-3-yl)butan-2-one (S21). *N*-methylindole (0.7819 g, 5.96 mmol) was solubilized in acetic acid (3.6 mL) under Ar atmosphere, then acetic anhydride (1.2 mL,12.5 mmol) and butenone (1.47 mL, 17.9 mmol) were added. The reaction flask was sealed and heated to 90 °C. After 1 h, the flask was cooled down to rt and s.s. $NaHCO_3$ was slowly added until pH 9. The mixture was extracted with AcOEt and the organic phases were washed with brine, dried (Na_2SO_4), filtrated and evaporated to dryness. The crude product was purified by silica FC (PE/AcOEt 7:1) delivering the product as yellow oil (696 mg, 58% yield). **Rf** 0.38 (PE/AcOEt 7:1, UV, CAM). **^{1}H NMR** (400 MHz, $CDCl_3$, 27°C) δ 7.57 (d, *J* = 7.9 Hz, 1H, 4-CH_{arom}), 7.28 (d, *J* = 8.2 Hz, 1H, 7-CH_{arom}), 7.22 (td, *J* = 6.8, 1.2 Hz, 1H, 6-CH_{arom}), 7.10 (td, *J* = 7.4, 1.1 Hz, 1H, 5-CH_{arom}), 6.84 (s, 1H, 2-CH_{arom}), 3.73 (s, 3H, NCH_3), 3.04 (t, *J* = 7.4 Hz, 2H, CH_2CH_2CO), 2.83 (t, *J* = 7.4 Hz, 2H, CH_2CO), 2.14 (s, 3H, $COCH_3$). **^{13}C NMR** (101 MHz, $CDCl_3$, 27°C) δ 208.9 (C=O), 137.2 (7a-$C_{q.}$), 127.7 (3a-$C_{q.}$), 126.5 (2-CH_{arom}), 121.7 (6-CH_{arom}), 118.9 (4- or 5-CH_{arom}), 118.7 (4- or 5-CH_{arom}), 113.8 (3-$C_{q.}$), 109.4 (7-CH_{arom}), 44.5 (CH_2CO), 32.7 (NCH_3), 30.2 ($COCH_3$), 19.4 (CH_2CH_2CO). **GC-MS**: t_R = 9.295 min (Method A); *m/z* (Relative Intensity %): 39 (2), 42 (3), 43 (9), 51 (2), 63 (3), 77 (9), 89 (3), 91 (2), 102 (5), 103 (4), 115 (12), 116 (3), 117 (4), 127 (2), 128 (7), 129 (3), 130 (3), 131 (3), 142 (3), 143 (12), 144 (100), 145 (11), 156 (2), 157 (3), 158 (11), 201 (25) $[M]^+$, 202 (4). **IR** wavenumber (cm^{-1}): 3053, 2917, 1710, 1615, 1553, 1472, 1424, 1356, 1325, 1276, 1247, 1207, 1187, 1159, 1131, 1066, 1012, 924, 801, 736, 654. Other data were in accordance with the literature.

3-(3-Iodobutyl)-1-methyl-1*H*-indole (S22). STEP 1. The ketone **S21** (690 mg, 3.43 mmol) was solubilized in dry methanol (6.9 mL) under N_2 atmosphere. The reaction flask was cooled to 0 °C and $NaBH_4$ (76 mg, 1.96 mmol) was added portion-wise. After 1 h, the initial yellow solution turned pale pink. Then water was slowly added, and the pH was adjusted to 3 using 1 M HCl. The mixture was extracted with AcOEt and the organic phases were washed with brine, dried (Na_2SO_4), filtrated and evaporated to dryness. The crude alcohol (697 mg, brown viscous oil) was used in the next step without further purification. **Rf** 0.28 (PE/Et_2O 6:4, UV, CAM). **^{1}H NMR** (400 MHz, $CDCl_3$, 27°C) δ 7.60 (d, *J* = 7.9 Hz, 1H, 4-CH_{arom}), 7.28 (d, *J* = 8.2 Hz, 1H, 7-CH_{arom}), 7.22 (t, *J* = 7.4 Hz, 1H, 6-CH_{arom}), 7.10 (t, *J* = 7.4 Hz, 1H, 5-CH_{arom}), 6.85 (s, 1H, 2-CH_{arom}), 3.96 – 3.83 (m, 1H, OCH), 3.74 (s, 3H, NCH_3), 2.96 – 2.75 (m, 2H, CH_2CH_2CH), 1.91 – 1.81 (m, 2H, CH_2CH), 1.39 (bs, 1H, OH), 1.24 (d, *J* = 6.2 Hz, 3H, $CHCH_3$). **GC-MS**: t_R = 9.395 min (Method A); *m/z* (Relative Intensity %): 39 (3), 42 (5), 43 (4), 44 (3), 45 (5), 51 (3), 63 (2), 76 (2), 77 (10), 89 (3), 91 (3), 102 (5), 103 (5), 115 (11), 116 (3), 117 (5), 127 (3), 128 (8), 129 (4), 130 (4), 131 (5), 132 (14), 142 (3), 143 (12), 144 (100), 145 (28), 146 (3), 156 (2), 157 (3), 158 (6), 170 (3), 203 (21) $[M]^+$, 204 (3). STEP 2. Following the general procedure for Appel reaction, the mixture of the crude alcohol (0.6538 g, 3.23 mmol), PPh_3 (1.018 g, 3.88 mmol), imidazole (264 mg, 3.88 mmol) and I_2 (985 mg, 3.88 mmol) in dry DCM (6.5 mL) was reacted. After workup, the crude product was purified by silica FC (PE/Et_2O 10:0.2) delivering the product as a pale-yellow solid (0.759 g, 75 % yield two steps). **Rf** 0.28 (PE/Et_2O 10:0.2, UV, CAM). **^{1}H NMR** (400 MHz, $CDCl_3$, 27°C) δ 7.61 (d, *J* = 7.9 Hz, 1H, 4-CH_{arom}), 7.29 (d, *J* = 8.2 Hz, 1H, 7-CH_{arom}), 7.23 (t, *J* = 7.6 Hz, 1H, 6-CH_{arom}), 7.11 (t, *J* = 7.4 Hz, 1H, 5-CH_{arom}), 6.90 (s, 1H, 2-CH_{arom}), 4.17 (tq, *J* = 9.8, 7.0 Hz, 1H, ICH), 3.75 (s, 3H, NCH_3), 2.99 (ddd, *J* = 13.6, 8.1, 5.0 Hz, 1H, $CHHCH_2CH$), 2.83 (dt, *J* = 15.0, 7.8 Hz, 1H, $CHHCH_2CH$), 2.19 (ddt, *J* = 14.1, 8.9, 4.3 Hz, 1H, CHHCH), 2.02 – 1.86 (m, 4H, CHHCH + $CHCH_3$). **^{13}C NMR** (101 MHz, $CDCl_3$, 27°C) δ 137.2 (7a-$C_{q.\ arom}$), 127.8 (3a-$C_{q.\ arom}$), 126.6 (2-CH_{arom}), 121.7 (6-CH_{arom}), 119.1(4-CH_{arom}), 118.8 (5-CH_{arom}), 113.3 (3-$C_{q-\ arom}$), 109.4 (7-CH_{arom}), 43.4 (CH_2CH), 32.7 (NCH_3), 31.0 (ICH), 29.2 ($CHCH_3$), 25.5 (CH_2CH_2CH). **GC-MS**: t_R = 10.325 min (Method A); *m/z* (Relative Intensity %): 39 (6), 41 (6), 42 (9), 76 (3), 77 (11), 102 (8), 103 (5), 115 (9), 127 (4), 128 (8), 129 (5), 143 (9), 144 (100), 145 (12), 186 (4), 313 (11) $[M]^+$. **IR** wavenumber (cm^{-1}): 2916, 1615, 1506, 1467, 1441, 1377, 1327, 1290, 1228, 1180, 1161, 1127, 1105, 1036, 882, 822, 737. **HRMS** (ESI+): calcd. for $C_{13}H_{17}IN$ $[M+H]^+$ 314.0400, found 314.0405.

2.11 Synthesis of *tert*-butyl(3-iodobutoxy)dimethylsilane (S26), 3-iodobutan-1-ol (S27) and 3-iodobutyl benzoate (S28)

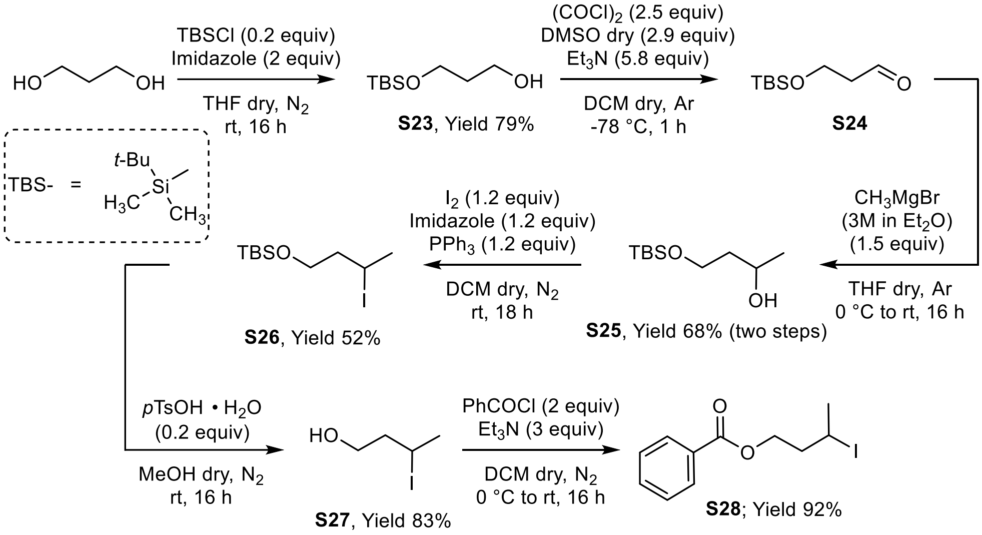

Scheme S10

3-((*tert*-Butyldimethylsilyl)oxy)propan-1-ol (S23). 1,3-Propanediol (4.7 mL, 65.7 mmol) was solubilized in dry THF (66 mL) under Ar atmosphere, then imidazole (8.945g, 131.4 mmol) was slowly added. When all the reagents were solubilized, *tert*-butyldimethylsilyl chloride (1.980 g, 13.14 mmol) was added. After 16 h, the solvent was removed using the rotavapor and the crude mixture was diluted with water and extracted with Et_2O. The organic phases were washed with brine, dried (Na_2SO_4), filtrated and evaporated to dryness. The crude product was purified by silica FC (PE/AcOEt 8:2) delivering the product as colourless oil (1.976 g, 79% yield). **Rf** 0.31 (PE/AcOEt 8:2, UV, CAM, basic $KMnO_4$). **^{1}H NMR** (400 MHz, $CDCl_3$, 27 °C) δ 3.87 – 3.82 (m, 2H, C*H*$_2$OH), 3.81 – 3.77 (m, 2H, CH_2OSi), 2.64 (s, 1H, OH), 1.78 (p, *J* = 5.6 Hz, 2H, CH_2C*H*$_2$$CH_2$), 0.90 (s, 9H, $C(CH_3)_3$), 0.08 (s, 6H, $Si(CH_3)_2$). **^{13}C NMR** (100 MHz, $CDCl_3$, 27 °C) δ 63.1 (CH_2OH), 62.6 (CH_2OSi), 34.4 ($CH_2CH_2CH_2$), 26.0 ($C(CH_3)_3$), 18.3 (*C*$(CH_3)_3$), -5.4 ppm ($Si(CH_3)_2$). **GC-MS**: t_R = 7.570 min (Method B); *m/z* (Relative Intensity %): 41 (7), 43 (3), 45 (9), 47 (6), 57 (3), 59 (6), 61 (3), 73 (8), 74 (3), 75 (100), 76 (7), 77 (5), 87 (3), 91 (3), 105 (35), 106 (3), 133 (13). **IR** wavenumber (cm^{-1}): 3350, 2953, 2929, 2857, 1472, 1388, 1361, 1254, 1084, 1006, 960, 939, 832, 773, 719, 661. Other data were in accordance with the literature.

3-((*tert*-Butyldimethylsilyl)oxy)propanal (S24). dry DMSO (1.165 mL, 16.40 mmol) was solubilized in dry DCM (30 mL) under inert Ar atmosphere in a 2-neck flask. The mixture was cooled to -78 °C and after 5 min $(COCl)_2$ (1.43 M in dry DCM, 9.9 mL, 14.16 mmol) was added. After 10 min a solution of the alcohol **S23** (1.0766 g, 5.66 mmol) in dry DCM (20 mL) was added dropwise. 5 mL of dry THF were used to wash the flask containing **S23** to transfer it quantitatively. After 15 min Et_3N (4.6 mL, 32.80 mmol) was added dropwise. After 1 h at -78 °C, a 5% aq. solution of $(NH_4)H_2PO_4$ was added while the reaction flask was let warm to rt. 2 M HCl was added until pH 5, then the mixture was extracted with Et_2O. The organic phases were washed with brine, dried (Na_2SO_4), filtrated and evaporated to dryness. The crude aldehyde (1.066 g, yellowish oil) was sufficiently pure to be used in the next step without further purification. **Rf** 0.69 (PE/AcOEt 8:2, UV, CAM). **^{1}H NMR** (400 MHz, $CDCl_3$, 27°C) δ 9.81 (s, 1H, CHO), 3.99 (t, *J* = 6.0 Hz, 2H, OCH_2), 2.60 (td, *J* = 6.2, 2.1 Hz, 2H, C*H*$_2$CHO), 0.88 (s, 9H, $C(CH_3)_3$), 0.07 (s, 6H, $Si(CH_3)_2$).

4-((*tert*-Butyldimethylsilyl)oxy)butan-2-ol (S25). The crude aldehyde **S24** (5.66 mmol) was solubilized in dry THF (28 mL) under Ar atmosphere. The flask was cooled to 0°C and a solution of CH_3MgBr (3 M in Et_2O, 2.83 mL, 8.49 mmol) was slowly added. The flask was let warm to rt and after 16 h s.s. NH_4Cl was slowly added. The mixture was extracted with Et_2O, and the organic phases were washed with brine, dried (Na_2SO_4), filtrated and evaporated to dryness. The crude product was purified by silica FC (PE:Et_2O 6:4) delivering the product as colourless oil (787 mg, 68% yield two steps). **Rf** 0.28 (PE/AcOEt 9:1, UV, CAM, basic $KMnO_4$). **^{1}H NMR** (400 MHz, $CDCl_3$, 27°C) δ 4.08 – 3.98 (m, 1H, C*H*OH), 3.93 – 3.86 (m, 1H, C*H*HCH), 3.85 – 3.78 (m, 1H, CH*H*CH), 3.41 (d, *J* = 2.3 Hz, 1H, OH), 1.74 – 1.60 (m, 2H, CH_2OSi), 1.20 (d, *J* = 6.2 Hz, 3H, CHC*H*$_3$), 0.90 (s, 9H, $SiC(CH_3)_3$),

0.09 – 0.07 (m, 6H, $Si(CH_3)_2$). **^{13}C NMR** (101 MHz, $CDCl_3$, 27°C) δ 68.5 (CHOH), 63.0 (*C*H_2CH), 40.0 (CH_2OSi), 26.0 ($SiC(CH_3)_3$), 23.5 ($CHCH_3$), 18.3 (Si*C*$(CH_3)_3$), -5.40 ($SiCH_3$), -5.45 ($SiCH_3$). **GC-MS**: t_R = 7.745 min (Method A); *m/z* (Relative Intensity %): 39 (3), 41 (6), 43 (6), 45 (14), 47 (6), 55 (12), 57 (5), 58 (3), 59 (7), 61 (3), 73 (12), 74 (3), 75 (100), 76 (7), 77 (5), 87 (3), 89 (4), 91 (4), 101 (4), 105 (66), 106 (5), 107 (3), 129 (3), 147 (4). **HRMS** (ESI+): calcd. for $C_{10}H_{25}O_2Si$ $[M+H]^+$ 205.1618, found 205.1614.

***tert*-Butyl(3-iodobutoxy)dimethylsilane (S26).** Following the general procedure for Appel reaction, the mixture of alcohol **S25** (0.791 g, 3.87 mmol), PPh_3 (1.514 g, 4.64 mmol), imidazole (316 mg, 4.64 mmol) and I_2 (1.178 g, 4.64 mmol) in dry DCM (7.7 mL) was reacted. After workup, the crude product was purified by silica FC (PE/DCM 8:2 to PE/DCM 1:1) delivering the product as colourless oil (632 mg, 52 % yield). **Rf** 0.49 (PE/DCM 8:2, UV, CAM, basic $KMnO_4$). **^{1}H NMR** (400 MHz, $CDCl_3$, 27°C) δ 4.35 (dqd, *J* = 9.5, 6.9, 4.5 Hz, 1H, CHI), 3.80 – 3.71 (m, 1H, C*H*HOSi), 3.65 (ddd, *J* = 10.3, 8.1, 4.8 Hz, 1H, CH*H*OSi), 2.07 – 1.92 (m, 4H, C*H*HCH + CHC*H*$_3$), 1.85 – 1.72 (m, 1H, CH*H*CH), 0.90 (s, 9H, $SiC(CH_3)_3$), 0.07 (d, *J* = 3.8 Hz, 6H, $Si(CH_3)_2$). **^{13}C NMR** (101 MHz, $CDCl_3$, 27°C) δ 62.8 (CH_2OSi), 45.5 (*C*H_2CH), 29.3 ($CHCH_3$), 27.0 (CHI), 26.1 ($SiC(CH_3)_3$), 18.4 (Si*C*$(CH_3)_3$), -5.1 ($SiCH_3$), -5.2 ($SiCH_3$). **GC-MS**: t_R = 6.625 min (Method A); *m/z* (Relative Intensity %): 39 (21), 40 (4), 41 (50), 42 (6), 43 (23), 44 (6), 45 (34), 47 (25), 53 (5), 55 (81), 56 (10), 57 (34), 58 (28), 59 (47), 60 (7), 61 (15), 71 (5), 73 (76), 74 (8), 75 (100), 76 (7), 77 (4), 85 (9), 87 (7), 88 (12), 89 (27), 90 (3), 99 (12), 101 (22), 102 (3), 113 (3), 115 (17), 127 (3), 129 (37), 130 (5), 131 (5), 145 (5), 185 (65), 186 (5), 187 (4), 201 (6), 215 (95), 216 (8), 217 (3), 257 (72), 258 (9), 259 (3). **IR** wavenumber (cm^{-1}): 2954, 2928, 2857, 1471, 1444, 1379, 1361, 1301, 1254, 1160, 1101, 1076, 1034, 1006, 991, 962, 939, 897, 831, 808, 774, 734, 662. **HRMS** (ESI+): calcd. for $C_{10}H_{24}IOSi$ $[M+H]^+$ 315.0636, found 315.0631.

3-Iodobutan-1-ol (S27). Iodide **S26** (501 mg, 1.59 mmol) was solubilized in dry MeOH (16 mL) under N_2 atmosphere, then *p*TsOH·H_2O (61 mg, 0.319 mmol) was slowly added. After 16 h, the solvent was removed using the rotavapor and the crude mixture was extracted with s.s. $NaHCO_3$ and Et_2O. The organic phases were washed with brine, dried (Na_2SO_4), filtrated and evaporated to dryness. The crude product was purified by silica FC (PE/AcOEt 7:3) delivering the product as colourless oil (264 mg, 83% yield). **Rf** 0.51 (PE/AcOEt 7:3, UV, CAM, basic $KMnO_4$). **^{1}H NMR** (400 MHz, $CDCl_3$, 27°C) δ 4.36 (dqd, *J* = 9.7, 6.9, 4.4 Hz, 1H, CHI), 3.83 (dt, *J* = 10.6, 5.3 Hz, 1H, C*H*HOH), 3.74 (dq, *J* = 10.9, 5.3 Hz, 1H, CH*H*OH), 2.09 – 1.96 (m, 4H, C*H*HCH + CH_3), 1.86 (dddd, *J* = 14.8, 8.1, 5.9, 4.4 Hz, 1H, CH*H*CH), 1.52 (bs, 1H, OH). **^{13}C NMR** (101 MHz, CDCl3, 27°C) δ 62.7 (CH_2OH), 44.9 (*C*H_2CH), 29.3 (CH_3), 26.2 (CHI). **GC-MS**: t_R = 6.520 min (Method A); *m/z* (Relative Intensity %): 38 (4), 39 (32), 40 (7), 41 (43), 42 (20), 43 (49), 44 (4), 45 (6), 53 (3), 55 (100), 56 (5), 57 (6), 73 (42), 127 (6), 200 (1) $[M]^{+}$. **IR** wavenumber (cm^{-1}): 3317, 2915, 2882, 1444, 1417, 1377, 1300, 1253, 1218, 1149, 1123, 1091, 1047, 1022, 985, 946, 904, 846, 814, 780. Other data were in accordance with the literature.

3-Iodobutyl benzoate (S28). Alcohol **S27** (250 mg, 1.25 mmol) was solubilized in dry DCM (13 mL) under N_2 atmosphere, then Et_3N (0.523 mL, 3.75 mmol) was added. The reaction flask was cooled to 0°C and PhCOCl (0.290 mL, 2.5 mmol) was dropwise added. After 16 h at rt, the solvent was reduced using a rotavapor and the crude mixture was extracted using Et_2O and a s.s. NH_4Cl. The organic phases were washed with brine, dried (Na_2SO_4), filtrated and evaporated to dryness. The crude product was purified by FC on silica (PE/ Et_2O 10:1) to give the product as colourless oil (350 mg, 92% yield). **Rf** 0.55 (PE/Et_2O 10:1, UV, CAM). **^{1}H NMR** (400 MHz, $CDCl_3$, 27°C) δ 8.06 – 8.00 (m, 2H, 2x*o*-CH_{arom}), 7.61 – 7.53 (m, 1H, *p*-CH_{arom}), 7.45 (t, *J* = 7.7 Hz, 2H, 2x*m*-CH_{arom}), 4.53 (dt, *J* = 11.2, 5.6 Hz, 1H, OC*H*H), 4.41 – 4.27 (m, 2H, OCH*H* + CHI), 2.27 (ddt, *J* = 14.6, 9.2, 5.4 Hz, 1H, C*H*HCH), 2.18 – 2.08 (m, 1H, CH*H*CH), 2.02 (d, *J* = 6.9 Hz, 3H CH_3). **^{13}C NMR** (101 MHz, $CDCl_3$, 27°C) δ 166.5 (C=O), 133.2 (*p*-CH_{arom}), 130.2 ($C_{q.\ arom}$), 129.7 (2x*o*-CH_{arom}), 128.6 (2x*m*-CH_{arom}), 64.8 (OCH_2), 41.6 (*C*H_2CH), 29.1 (CH_3), 24.3 (CHI). **GC-MS**: t_R = 9.040 min (Method A); *m/z* (Relative Intensity %): 39 (16), 41 (17), 42 (4), 43 (3), 50 (10), 51 (33), 53 (4), 54 (4), 55 (70), 56 (7), 76 (5), 77 (67), 78 (5), 79 (10), 105 (100), 106 (8), 123 (40), 124 (3), 177 (24), 178 (3). **IR** wavenumber (cm^{-1}): 2959, 2914, 1715, 1602, 1584, 1492, 1450, 1380, 1314, 1267, 1174, 1108, 1097, 1069, 1026, 968, 846, 806, 707, 686, 675, 617. **HRMS** (ESI+): calcd. for $C_{11}H_{14}IO_2$ $[M+H]^+$ 305.0033, found 305.0039.

2.12 Synthesis of (3-iodo-3-methylbutyl)benzene (S30)

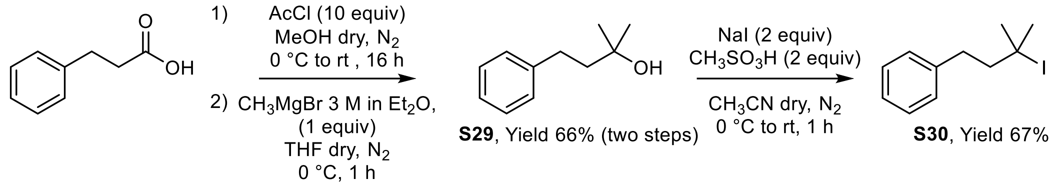

Scheme S11

2-Methyl-4-phenylbutan-2-ol (S29). STEP1. 3-Phenylpropanoic acid (1.0687 g, 7.12 mmol) was solubilized dry MeOH (24 mL) under N_2 atmosphere The flask was cooled to 0 °C and AcCl (5.06 mL, 71.2 mmol) was added dropwise. After 16 h, the solvent was reduced using a rotavapor and the crude mixture was extracted using s.s. $NaHCO_3$ and AcOEt. The organic phases were washed with brine, dried (Na_2SO_4), filtrated and evaporated to dryness. The crude methyl ester (1.169 g, colourless oil) was was sufficiently pure to be used in the next step without further purification. **Rf** 0.78 (PE/AcOEt 7:3, UV). STEP 2. The methyl ester (7.12 mmol) was solubilized in dry THF (14 mL) under Ar atmosphere. The reaction flask was cooled to 0 °C and CH_3MgBr (3 M in Et_2O, 2.4 mL, 7.12 mmol) was slowly added. After 1 h at rt, s.s. NH_4Cl was slowly added and 0.1 M HCl was added until pH 3. The mixture was extracted with Et_2O and the organic phases were washed with brine, dried (Na_2SO_4), filtrated and evaporated to dryness. The crude product was purified by silica FC (PE/Et_2O 7:3) to give the product as a white solid (772 mg, 66% yield two step). **Rf** 0.24 (PE/Et_2O 7:3, UV, CAM). **^{1}H NMR** (300 MHz, $CDCl_3$, 27 °C) δ 7.28 – 7.24 (m, 2H, 2xCH_{arom}), 7.19 – 7.15 (m, 3H, 3xCH_{arom}), 2.71 – 2.67 (m, 2H, $PhCH_2$), 1.80 – 1.76 (m, $PhCH_2CH_2$), 1.28 (d, 6H, $C(CH_3)_2$) **^{13}C NMR** (75 MHz, $CDCl_3$, 27 °C) 142.5 ($C_{q.arom}$), 128.4 (2xCH_{arom}), 128.3 (2xCH_{arom}), 125.7 (CH_{arom}), 70.9 ($C(CH_3)_2$), 30.7 ($PhCH_2CH_2$), 29.4 ($PhCH_2$), 29.3 ($C(CH_3)_2$). **GC-MS**: t_R = 6.280 min (Method A); *m/z* (Relative Intensity %): 39 (11), 41 (10), 43 (19), 51 (9), 58 (5), 59 (100), 60 (4), 63 (4), 65 (16), 77 (12), 78 (10), 79 (7), 89 (3), 91 (85), 92 (7), 103 (5), 104 (4), 105 (7), 106 (5), 129 (3), 131 (54), 132 (7), 146 (35), 147 (5), 149 (4). **IR** wavenumber (cm^{-1}): 3375, 3063, 3027, 2969, 2933, 1604, 1494, 1454, 1376, 1273, 1211, 1150, 1124, 1071, 1031, 980, 926, 914, 829, 767, 738, 697. Other data were in accordance with the literature.

(3-Iodo-3-methylbutyl)benzene (S30). The alcohol **S29** (118 mg, 0.699 mmol) was solubilized in dry CH_3CN (3.5 mL) under N_2 atmosphere. NaI (210 mg, 1.40 mmol) was added. The flask was cooled to 0 °C and CH_3SO_3Cl (91 μL, 1.40 mmol) was added. After 1 h at rt, the flask was cooled again to 0 °C and s.s. $NaHCO_3$ was slowly poured and then, the flask was let warm to rt. The crude mixture was extracted with Et_2O, and the organic phases were washed with brine, dried (Na_2SO_4), filtrated and evaporated to dryness. The crude product was purified by silica FC (PE/Et_2O 9:1) to give the product as a white solid (128 mg, 67% yield). **Rf** 0.75 (PE/Et_2O 9:1, UV, CAM). **^{1}H NMR** (400 MHz, $CDCl_3$, 27 °C) δ 7.32 – 7.26 (m, 2H, 2xCH_{arom}), 7.24 – 7.16 (m, 3H, 3xCH_{arom}), 2.87 – 2.80 (m, 2H, $PhCH_2$), 1.99 (s, 6H, 2xCH_3), 1.94 – 1.87 (m, 2H, $PhCH_2CH_2$). **^{13}C NMR** (101 MHz, $CDCl_3$, 27 °C) δ 141.6 ($C_{q.\ arom}$), 128.6 (2xCH_{arom}), 128.6 (2xCH_{arom}), 126.1 (*p*-CH_{arom}), 52.5 ($PhCH_2CH_2$), 51.6 ($C(CH_3)_2$), 38.2 (2xCH_3), 35.3 ($PhCH_2$). **GC-MS**: t_R = 7.695 min (high degradation was observed) (Method A); *m/z* (Relative Intensity %), 39 (9), 40 (1), 41 (10), 43 (3), 44 (2), 50 (2), 51 (5), 52 (1), 53 (2), 55 (1), 56 (6), 63 (3), 64 (1), 65 (14), 77 (4), 78 (2), 79 (1), 89 (3), 90 (1), 91 (100), 92 (9), 103 (2), 104 (2), 105 (11), 115 (2), 116 (1), 117 (1), 127 (2), 128 (2), 129 (1), 131 (5), 146 (4), 147 (19), 148 (3). **IR** wavenumber (cm^{-1}): 3062, 3026, 2958, 2923, 1603, 1495, 1453, 1385, 1368, 1274, 1225, 1187, 1147, 1099, 1070, 1030, 905, 846, 797, 744, 697, 608. **HRMS** (ESI+): calcd. for $C_{11}H_{16}I$ $[M+H]^+$ 275.0291, found 275.0287.

3 Procedures for isocyanide synthesis

3.1 Synthesis of 1-isocyano-4-methylbenzene (S32)

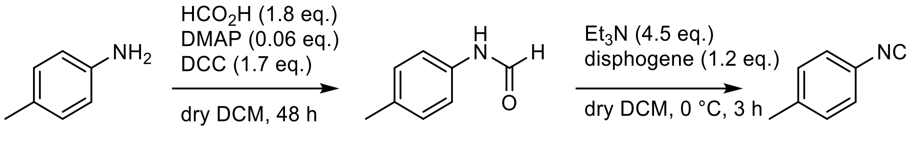

S31, Yield 75% **S32**, Yield 98%

Scheme S12

***N-p*-Tolylformamide (S31).** *p*-Toluidine (544.1 mg, 5.08 mmol) was solubilized in dry DCM (51 mL) under N_2 atmosphere. Then HCO_2H (230 µL, 6.09 mmol), DMAP (8.3 mg, 0.2 mmol) and DCC (36.9 mg, 5.59 mmol) were added. After 16 h, TLC showed the presence of unreacted aniline, and an addition of HCO_2H (3.05 mmol), DMAP (0.1 mmol) and DCC (2.80 mmol) was made. After 48 h, TLC showed the full conversion of the starting aniline. The solvent was removed by rotavapor and Et_2O was added. The resulting white solid was filtered off and the crude product was purified by silica FC (PE/Et_2O 7:3) to give the product as orange-brown solid (515 mg, 75% yield). **Rf** 0.22 (PE/AcOEt, UV, CAM). **m.p.** 50.5 – 51.5 °C. **^{1}H NMR** (400 MHz, $CDCl_3$, 27 °C) 2 rotamers (A e B) in ratio 1:1: δ 8.63 (d, J = 11.5 Hz, 1H, HC=O *A*), 8.54 – 8.40 (m, 1H, NH *A*), 8.34 (d, J = 1.8 Hz, 1H, HC=O *B*), 7.64 (s, 1H, NH *B*), 7.45 – 7.40 (m, 2H, 2xCH_{arom} *A*), 7.17 – 7.14 (m, 2H, 2xCH_{arom} *B*), 7.14 – 7.11 (m, 2H, 2xCH_{arom} *A*), 7.02 – 6.97 (m, 2H, 2xCH_{arom} *B*), 2.33 (s, 3H, CH_3 *A*), 2.31 (s, 3H, CH_3 *B*). **^{13}C NMR** (100 MHz, $CDCl_3$, 27 °C) 2 rotamers (A e B) in ratio 1:1: δ 163.0 (C=O *A*), 159.2 (C=O *B*), 135.2 ($C_{q.\ arom}$ *A*), 134.6 ($C_{q.\ arom}$ *B*), 134.4 ($C_{q.\ arom}$ *B*), 134.2 ($C_{q.\ arom}$ *A*), 130.3 (2xCH_{arom} *B*), 129.7 (2xCH_{arom} *A*), 120.1 (2xCH_{arom} *A*), 119.2 (2xCH_{arom} *B*), 21.00 (CH_3 *A*), 20.9 (CH_3 *B*). **GC-MS**: t_R = 7.165 min (Method A); *m/z* (Relative Intensity %): 39 (11), 41 (3), 50 (6), 51 (13), 52 (10), 53 (8), 63 (5), 65 (6), 77 (26), 78 (9), 79 (15), 80 (6), 91 (4), 104 (3), 106 (100), 107 (28), 108 (7), 135 (60) $[M]^+$, 136 (5). **IR** wavenumber (cm^{-1}): 3322, 3176, 3058, 2986, 2929, 2896, 2850, 1923, 1686, 1650, 1607, 1583, 1518, 1474, 1407, 1381, 1308, 1225, 1185, 1113, 1089, 1033, 889, 822, 782, 709, 642, 602. Other data were in accordance with the literature.

1-Isocyano-4-methylbenzene (S32). The formamide **S31** (137 mg, 1.00 mmol) was solubilized in dry DCM (3.3 mL) under Ar atmosphere, then Et_3N (419 µL, 3.00 mmol) was added. The flask was cooled to 0 °C and diphosgene (97 µL, 0.800 mmol) was added. After 1.5 h at 0 °C, TLC showed the presence of unreacted formamide, and an addition of Et_3N (1.50 mmol) e difosgene (0.400 mmol) was made. After 1.5 h, TLC showed the full conversion of the starting formamideand s.s. $NaHCO_3$ was slowly poured, and the flask was warmed to rt. The mixture was extracted with Et_2O, and the organic phases were washed with brine, dried (Na_2SO_4), filtrated and evaporated to dryness. The crude product was purified by silica FC (PE/Et_2O 96:4) to give the product as brown oil (115 mg, 98% yield). **Rf** 0.29 (PE/Et_2O 96:4, UV, $KMnO_4$). **^{1}H NMR** (300 MHz, $CDCl_3$, 27 °C) δ 7.28 – 7.22 (m, 2H, 2xCH_{arom}), 7.22 – 7.12 (m, 2H, 2xCH_{arom}), 2.37 (m, 3H, CH_3). **^{13}C NMR** (100 MHz, $CDCl_3$, 27 °C) δ 166.00 (NC), 144.20 ($C_{q.\ arom}$), 129.00 (2xCH_{arom}), 126.70 (2xCH_{arom}), 123.48 ($C_{q.\ arom}$), 21.44 (CH_3). **IR** wavenumber (cm^{-1}): 3262, 3032, 2922, 2858, 2123, 1684, 1605, 1515, 1450, 1406, 1314, 1253, 865, 816.

4 Side products: ketoamides

***N*-Cyclohexyl-2-oxo-5-phenylpentanamide (17).** The reaction was carried out following the general procedure for isocyanide insertion (condition B) on 100 mg scale of iodide **S3** and using *c*-hexyl isocyanide as isocyanide. After FC (PE/Et_2O 7:3) the main spot isolated was the ketoamide **17** (pale-yellow solid 19 mg, 19% yield). **Rf** 0.20 (PE/Et_2O 7:3, UV, CAM). **^{1}H NMR** (400 MHz, $CDCl_3$, 27 °C) δ 7.31 – 7.27 (m, 2H, 2xCH_{arom}), 7.22 – 7.15 (m, 3H, 3xCH_{arom}), 6.81 (bd, *J* = 7.1 Hz, 1H, NH), 3.71 (dddd, *J* = 14.5, 10.6, 8.3, 3.9 Hz, 1H, CH *c*-Hex), 2.95 (t, *J* = 7.3 Hz, 2H, $PhCH_2$), 2.66 (t, *J* = 7.3 Hz, 2H, CH_2CO), 2.00 – 1.84 (m, 4H, $PhCH_2CH_2$ + CH_2 *c*-Hex), 1.73 (dt, *J* = 13.3, 3.7 Hz, 2H, CH_2 *c*-Hex), 1.63 (dt, *J* = 12.8, 3.7 Hz, 1H, C*H*H *c*-Hex), 1.46 – 1.30 (m, 2H, CH_2 *c*-Hex), 1.28 – 1.13 (m, 3H, CH_2 *c*-Hex + CH*H* *c*-Hex). **^{13}C NMR** (101 MHz, $CDCl_3$, 27 °C) δ 199.5 (*C*=O(CONH)), 159.1 (CONH), 141.5 ($C_{q.\ arom}$), 128.6 (2xCH_{arom}), 128.5 (2xCH_{arom}), 126.1 (*p*-CH_{arom}), 48.4 (CH *c*-Hex), 36.2 (CH_2CO), 35.1 ($PhCH_2$), 32.8 (2xCH_2 *c*-Hex), 25.4 (CH_2 *c*-Hex), 25.0 ($PhCH_2CH_2$), 24.8 (2xCH_2 *c*-Hex). **GC-MS:** t_R = 14.095

min (Method A); *m/z* (Relative Intensity %): 39 (7), 41 (27), 43 (5), 55 (58), 56 (7), 65 (9), 83 (100), 84 (7), 88 (10), 91 (64), 92 (7), 98 (8), 104 (23), 105 (6), 120 (22), 126 (11), 129 (13), 147 (39), 169 (10), 217 (31), 218 (5), 245 (9) $[M]^+$. **IR** wavenumber (cm^{-1}): 3320, 3068, 3034, 2939, 2918, 2855, 1722, 1654, 1607, 1521, 1497, 1454, 1385, 1351, 1314, 1287, 1260, 1246, 1228, 1193, 1119, 1071, 1032, 965, 928, 903, 890, 842, 812, 761, 735, 695, 653. **HRMS** (ESI+): calcd. for $C_{17}H_{24}NO_2$ $[M+H]^+$ 274.1802, found 274.1807.

2-Oxo-*N*-phenethyl-5-phenylpentanamide (18). The reaction was carried out following the general procedure for isocyanide insertion (condition B) on 100 mg scale of iodide **S3** and using (2-isocyanoethyl)benzene as isocyanide. After FC (PE/Et_2O 7:3) the main spot isolated was the ketoamide **18** (pale-yellow solid, 14 mg, 12% yield). **Rf** 0.32 (PE/Et_2O 7:3, UV, CAM). **^{1}H NMR** (400 MHz, $CDCl_3$, 27 °C) δ 7.34 – 7.21 (m, 5H, 5xCH_{arom}), 7.21 – 7.15 (m, 5H, 5xCH_{arom}), 6.96 (bs, 1H, NH), 3.55 (q, *J* = 7.0 Hz, 2H, C*H*$_2$NH), 2.94 (t, *J* = 7.3 Hz, 2H, C*H*$_2$$CH_2CH_2$CO), 2.84 (t, *J* = 7.1 Hz, 2H, C*H*$_2$$CH_2$NH), 2.65 (t, *J* = 7.4 Hz, 2H, CH_2CO), 1.93 (p, *J* = 7.5 Hz, 2H, C*H*$_2$$CH_2$CO). **^{13}C NMR** (101 MHz, $CDCl_3$, 27 °C) δ 199.0 (*C*=O(CONH)), 160.1 (CONH), 141.5 (**C**$_{q.\ arom}$), 138.3 (**C**$_{q.\ arom}$), 128.9 (2xCH_{arom}), 128.8 (2xCH_{arom}), 128.63 (2xCH_{arom}), 128.57 (2xCH_{arom}), 126.9 (CH_{arom}), 126.2 (CH_{arom}), 40.6 (CH_2NH), 36.2 ($CH_2CH_2CH_2$CO), 35.6 (C*H*$_2$$CH_2$NH), 35.1 ($CH_2$CO [3]), 24.9 ($CH_2CH_2$CO). **GC-MS**: t_R = 11.805 min (Method A); *m/z* (Relative Intensity %): 39 (7), 41 (9), 44 (5), 51 (6), 55 (4), 63 (3), 65 (19), 73 (4), 77 (18), 78 (7), 79 (17), 89 (3), 91 (100), 92 (9), 100 (3), 103 (12), 104 (64), 105 (76), 106 (6), 115 (3), 117 (4), 119 (3), 120 (5), 129 (13), 147 (36), 148 (14), 191 (5), 207 (4), 239 (24), 240 (4), 267 (14), 268 (3), 28), 295 (2) $[M]^+$. **IR** wavenumber (cm^{-1}): 3343, 3062, 3028, 2928, 2852, 1721, 1659, 1603, 1533, 1495, 1455, 1396, 1376, 1285, 1263, 1233, 1192, 1120, 1078, 1027, 991, 907, 853, 805, 742, 722, 693, 651. Other data were in accordance with the literature.

3-Methyl-2-oxo-*N*-phenethyl-5-phenylpentanamide (19). The reaction was carried out following the general procedure for isocyanide insertion (condition B) on 100 mg scale of iodide **1** and using (2-isocyanoethyl)benzene as isocyanide. After FC (PE/Et_2O 8:2) the main spot isolated was the ketoamide **19** (pale-yellow oil, 11 mg, 9% yield). **Rf** 0.35 (PE/Et_2O 8:2, UV, CAM). **^{1}H NMR** (400 MHz, $CDCl_3$, 27 °C) δ 7.34 – 7.28 (m, 2H, 2xCH_{arom}), 7.28 – 7.22 (m, 3H, 3xCH_{arom}), 7.22 – 7.14 (m, 5H, 5xCH_{arom}), 6.95 (bs, 1H, NH), 3.65 – 3.50 (m, 3H, C*H*CH_3 + C*H*$_2$NH), 2.85 (t, *J* = 7.1 Hz, 2H, C*H*$_2$$CH_2$NH), 2.68 – 2.52 (m, 2H, C*H*$_2$$CH_2$CH), 2.05 (ddt, *J* = 13.3, 10.0, 6.5 Hz, 1H, C*H*HCH), 1.71 – 1.60 (m, 1H, CH*H*CH), 1.14 (d, *J* = 7.0 Hz, 3H, CH_3). **^{13}C NMR** (101 MHz, $CDCl_3$, 27 °C) δ 202.0 (CH(*C*=O)), 159.9 ((C=O)NH), 141.7 ($C_{q.arom}$-CH_2CH_2CH), 138.3 ($C_{q.arom}$-CH_2CH_2NH), 128.9 (2xCH_{arom}), 128.8 (2xCH_{arom}), 128.6 (2xCH_{arom}), 128.5 (2xCH_{arom}), 126.9 (*p*-CH_{arom}), 126.1 (*p*-CH_{arom}), 40.6 (CH_2NH), 38.9 (CH), 35.6 (CH_2CH_2NH), 34.2 (CH_2CH), 33.6 (CH_2CH_2CH), 16.0 (CH_3). **GC-MS**: t_R = 11.810 min (Method A); *m/z* (Relative Intensity %): 39 (4), 41 (3), 51 (3), 65 (12), 77 (8), 78 (3), 79 (8), 91 (100), 92 (9), 103 (5), 104 (22), 105 (34), 106 (3), 114 (6), 133 (8), 148 (3), 161 (8), 205 (10), 239 (20), 240 (3). **IR** wavenumber (cm^{-1}): 3362, 3064, 3027, 2929, 2861, 1719, 1659, 1604, 1532, 1496, 1454, 1370, 1285, 1240, 1193, 1128, 1100, 1044, 1029, 977, 905, 883, 846, 815, 739, 695. **HRMS** (ESI+): calcd. For $C_{20}H_{24}NO_2$ $[M+H]^+$ 310.1802, found 310.1807.

5 Photochemical vessel holder

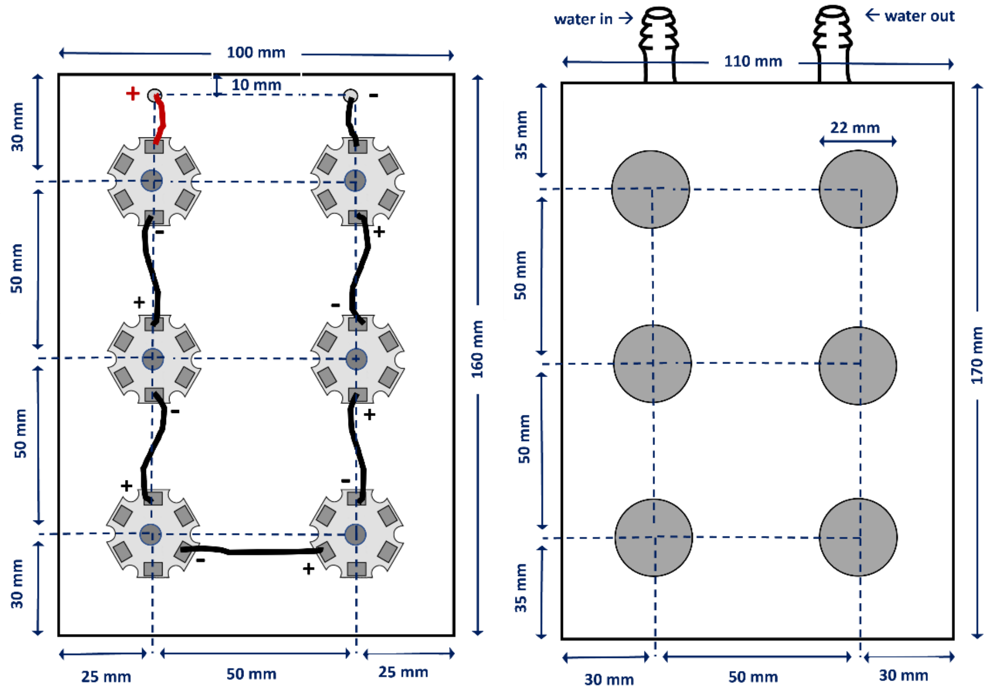

6 Mechanism: alternative pathway from III

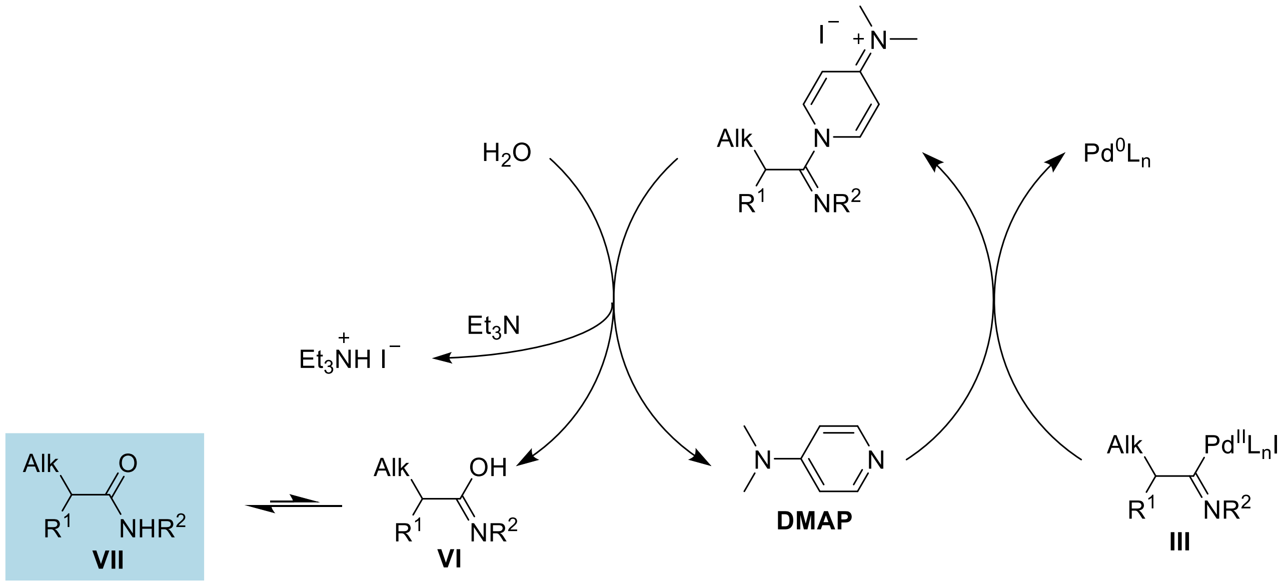

Scheme S13

7 NMR copies

N-(*tert*-Butyl)-2-methyl-4-phenylbutanamide (2).

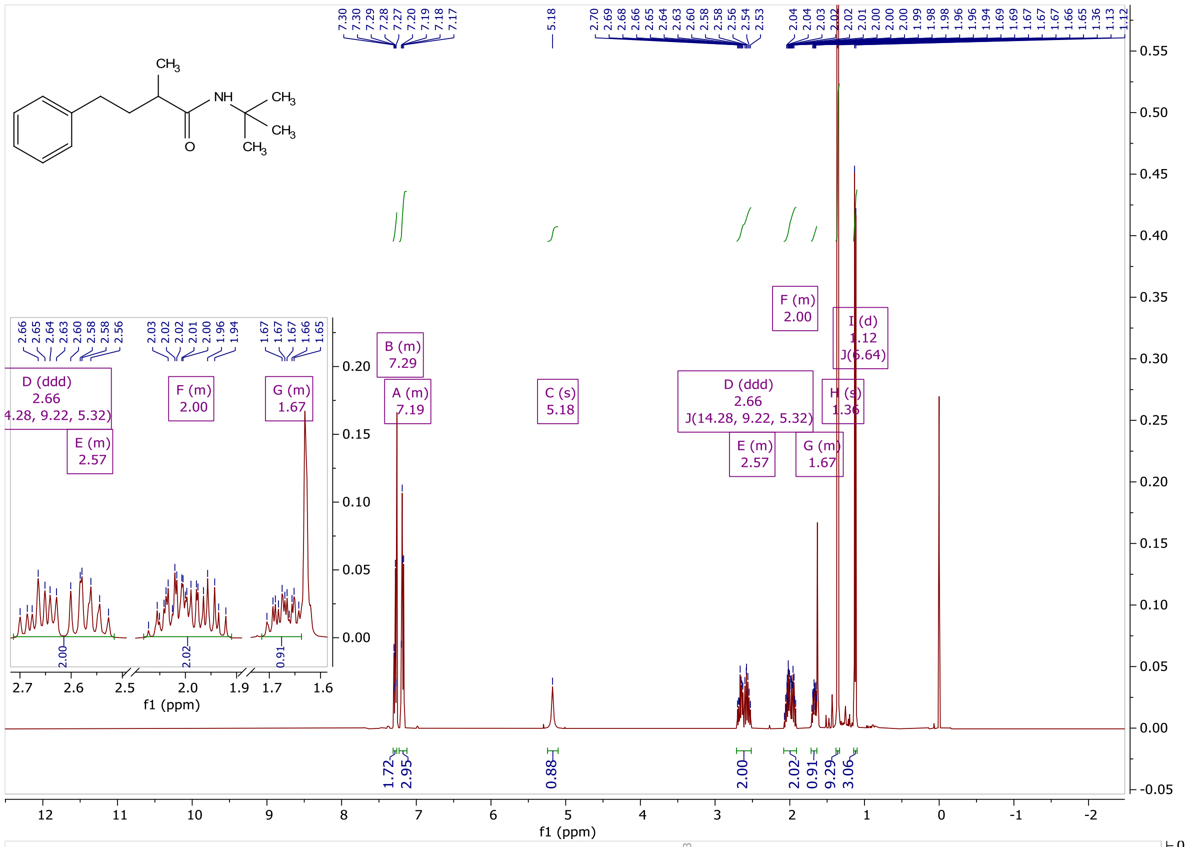

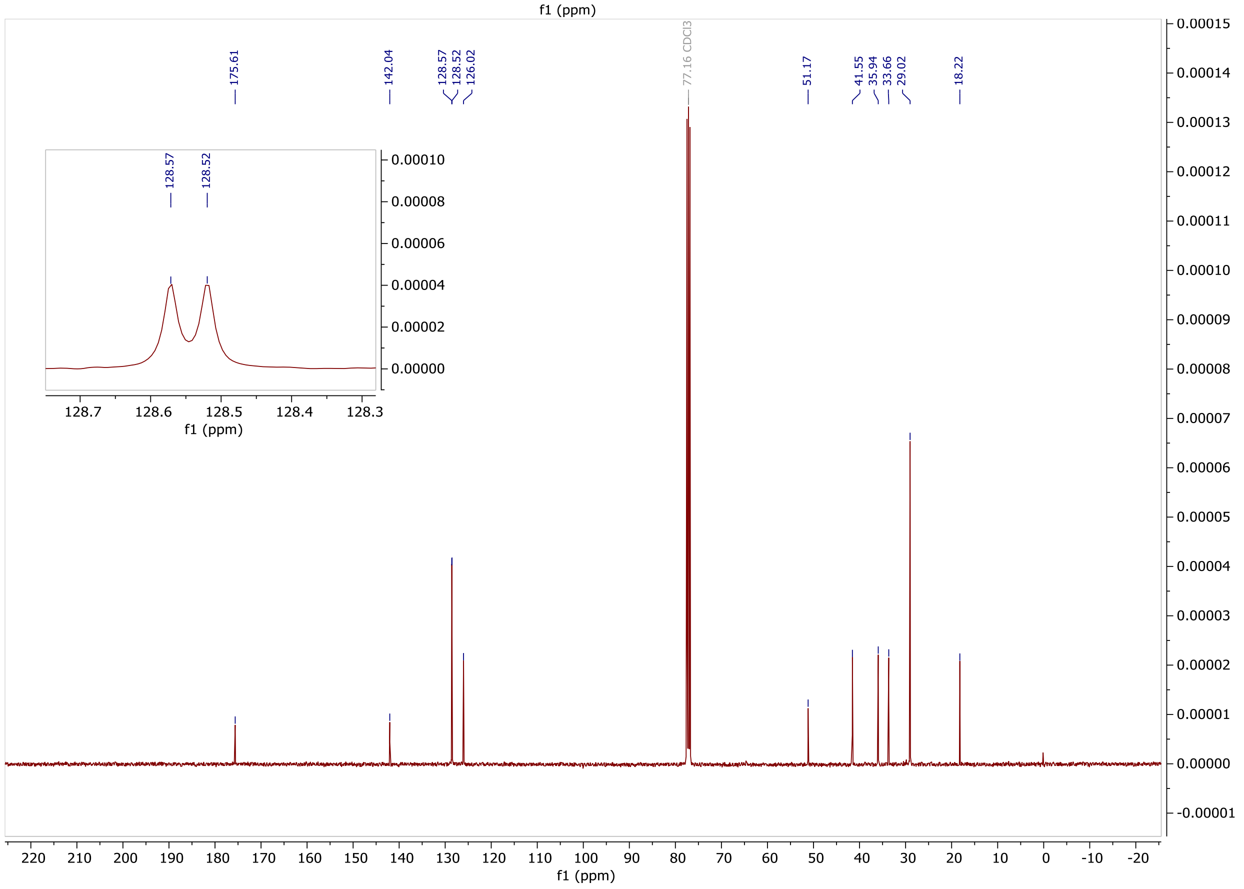

N-(tert-Butyl)cyclohexanecarboxamide (3).

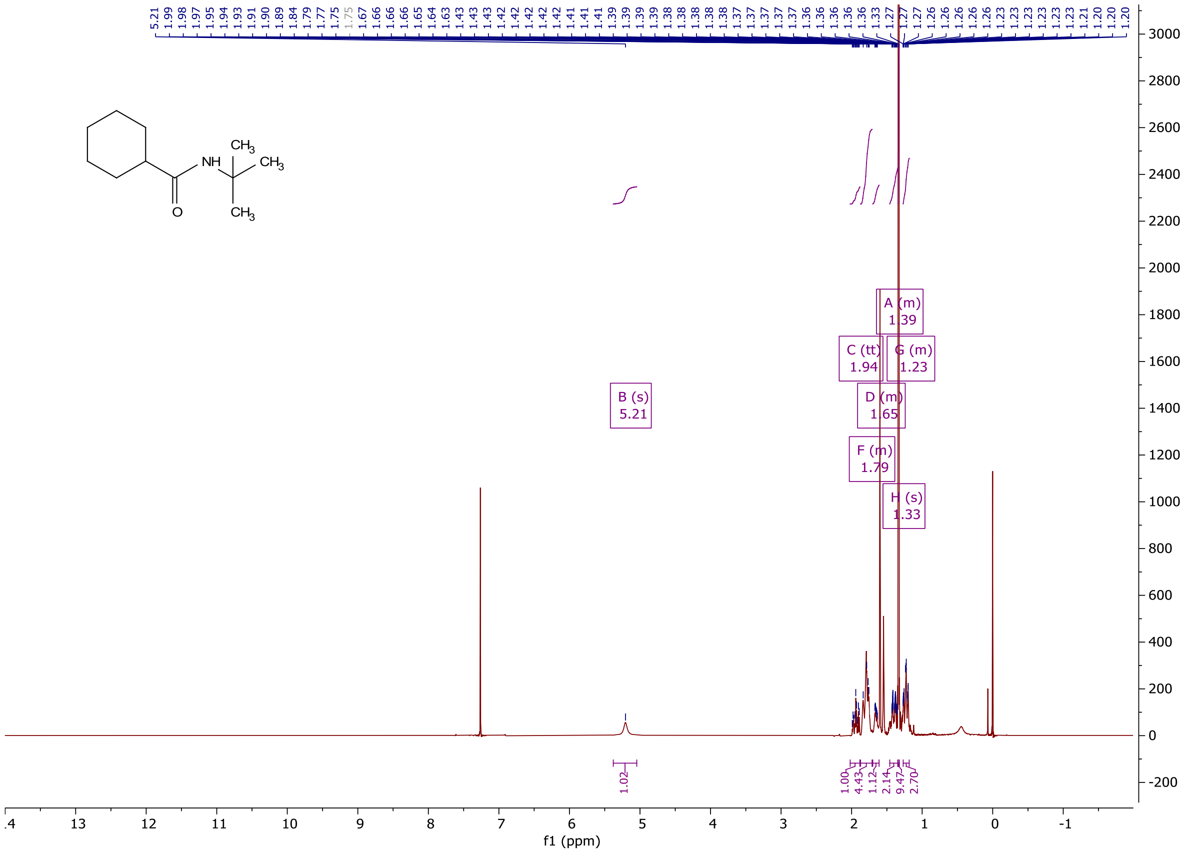

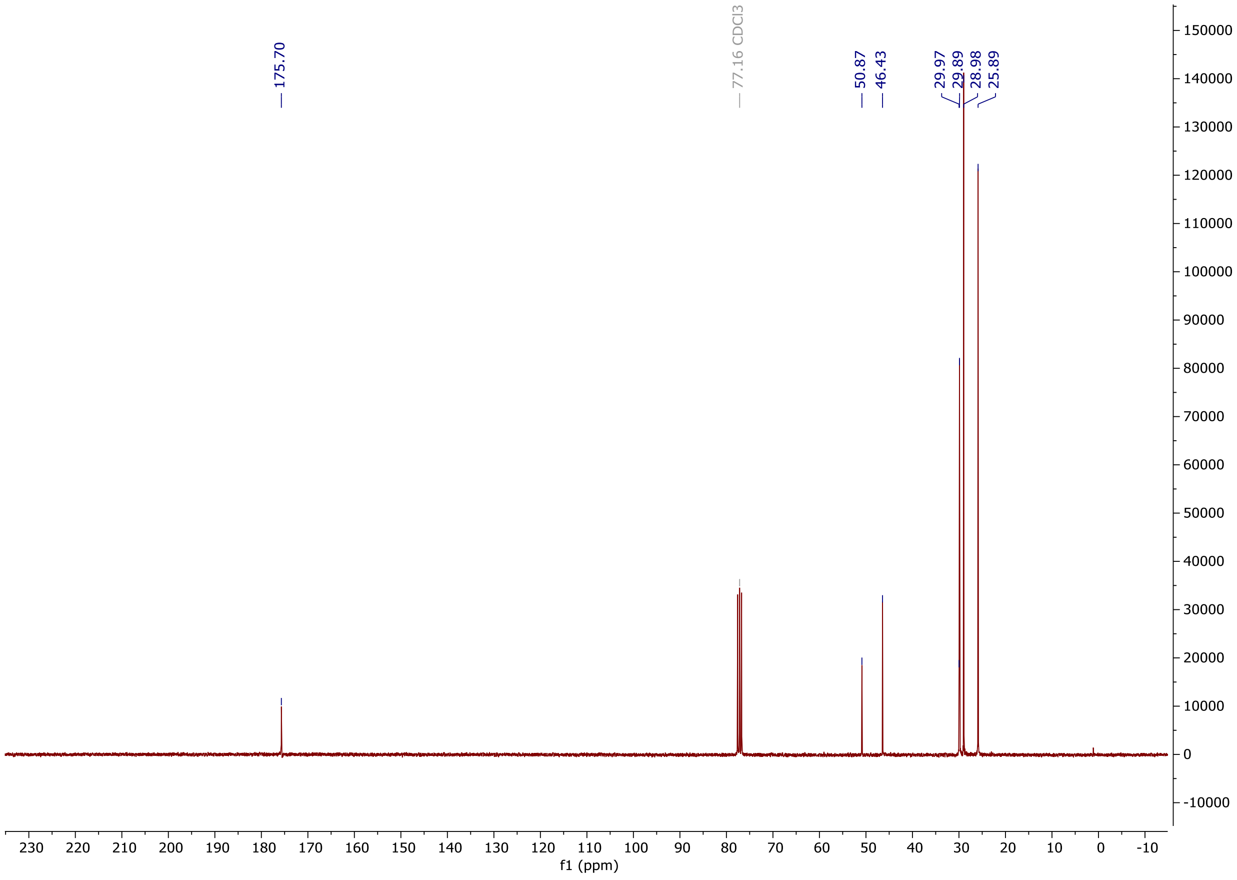

***N*-(*tert*-butyl)-4-phenylbutanamide (4).**

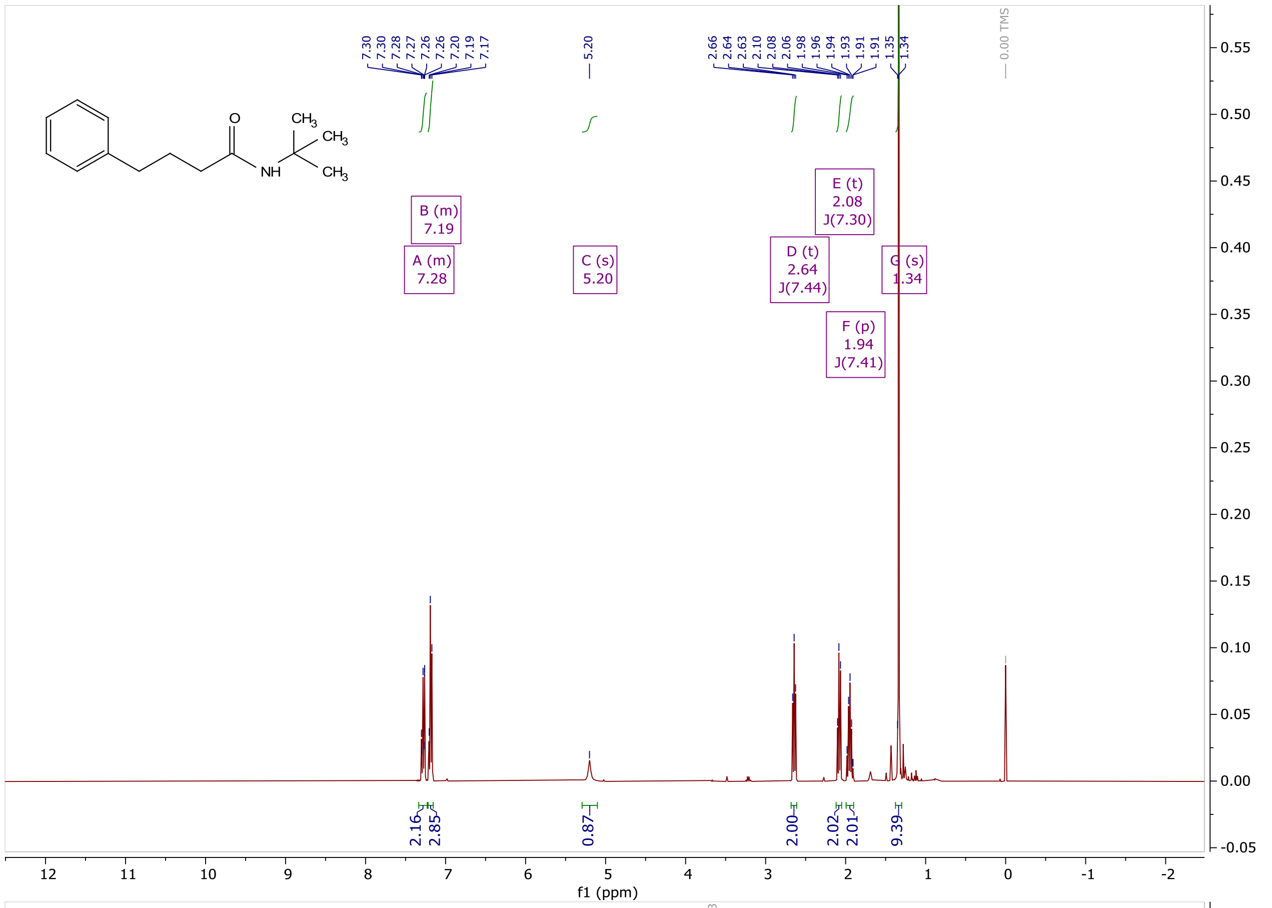

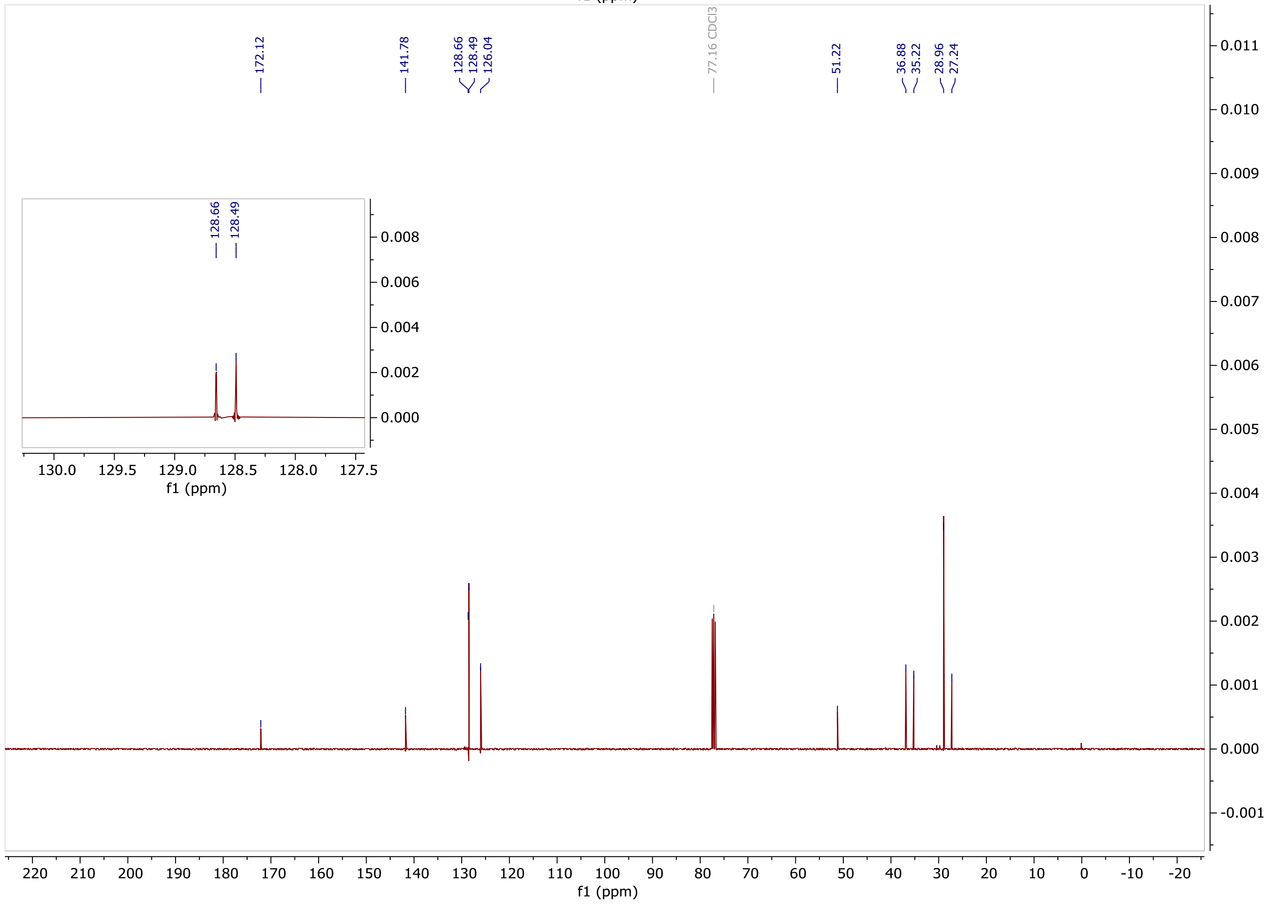

(3*r*,5*r*,7*r*)-*N*-(*tert*-Butyl)adamantane-1-carboxamide (6).

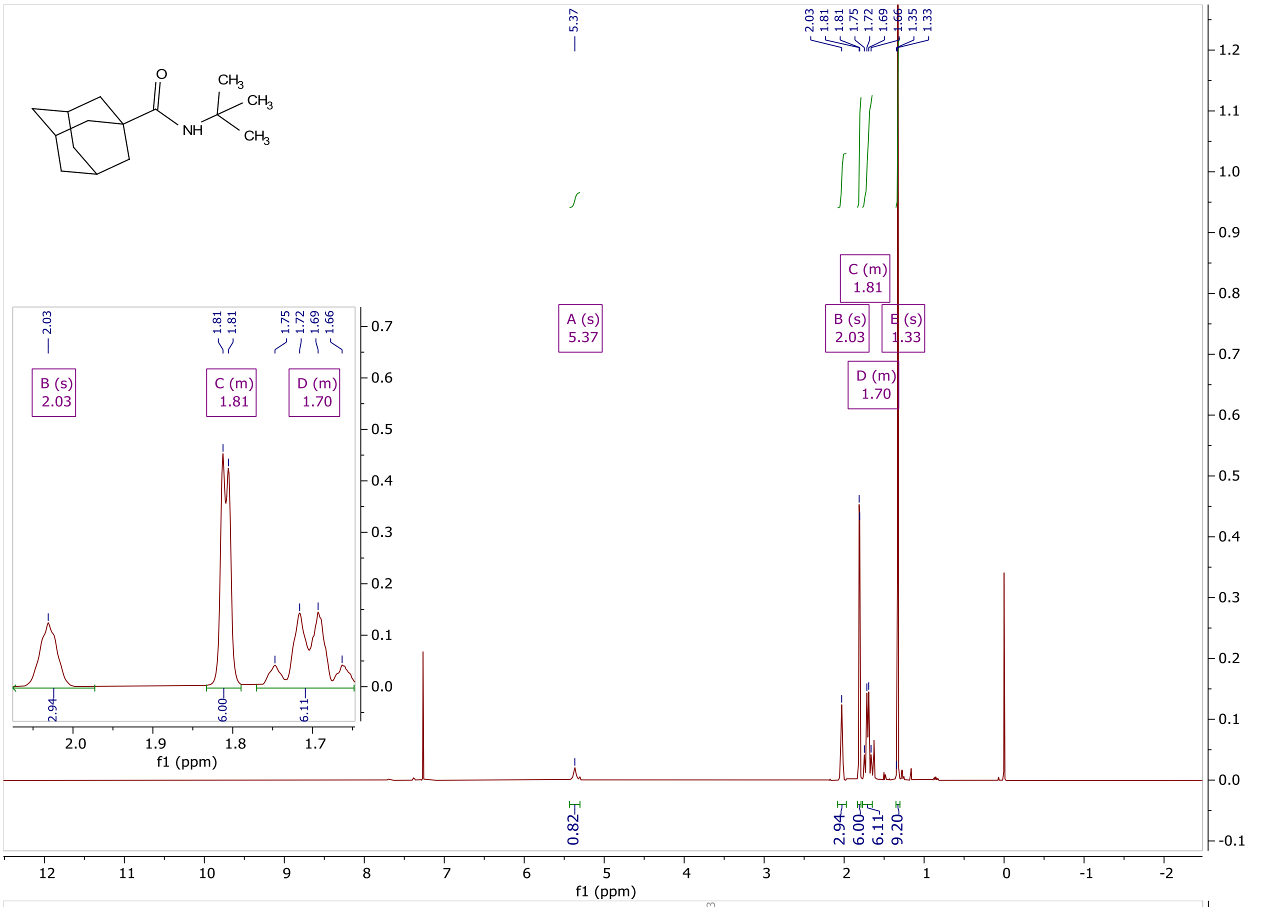

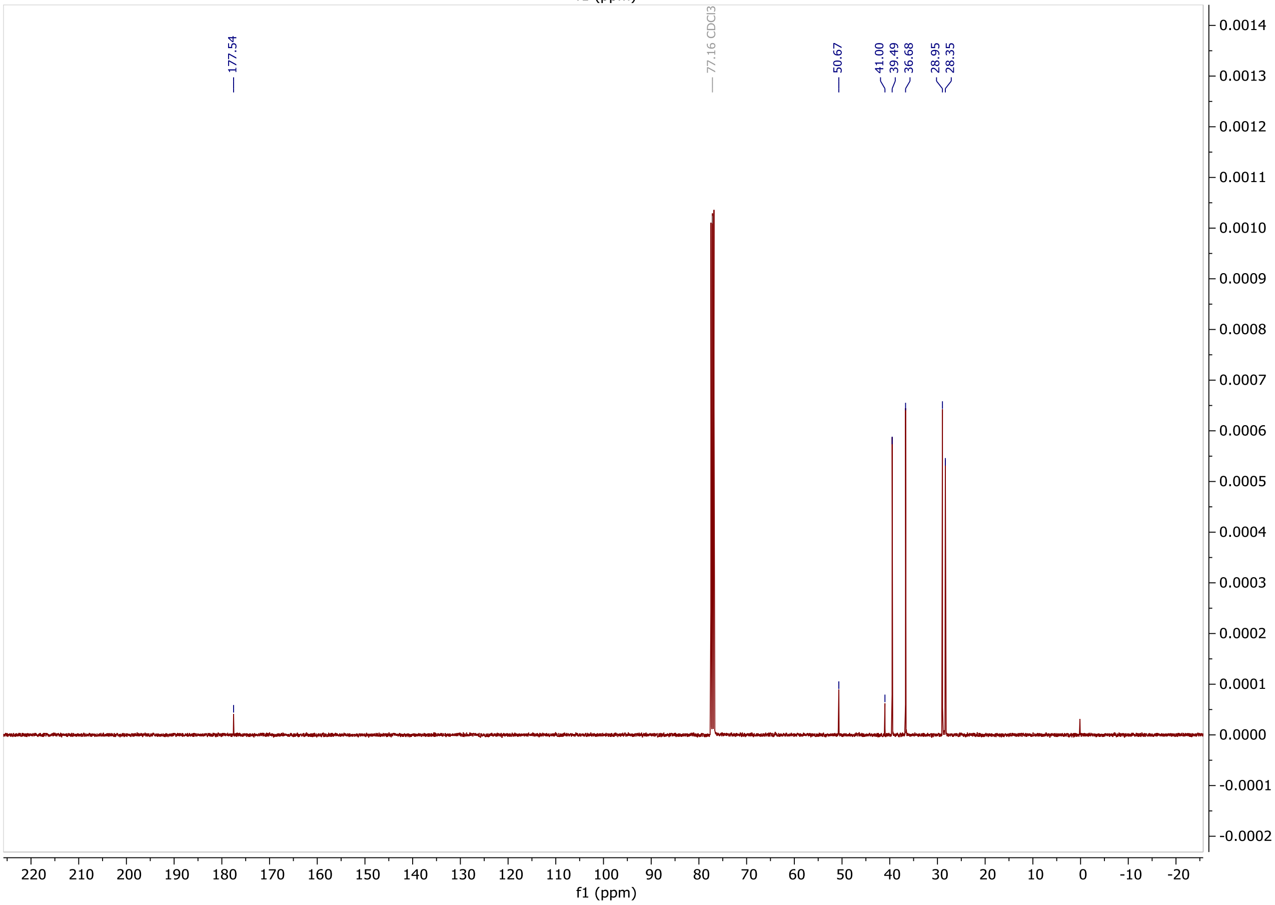

_N_-(_tert_-Butyl)-3,3-dimethyl-4-phenylbutanamide (7).

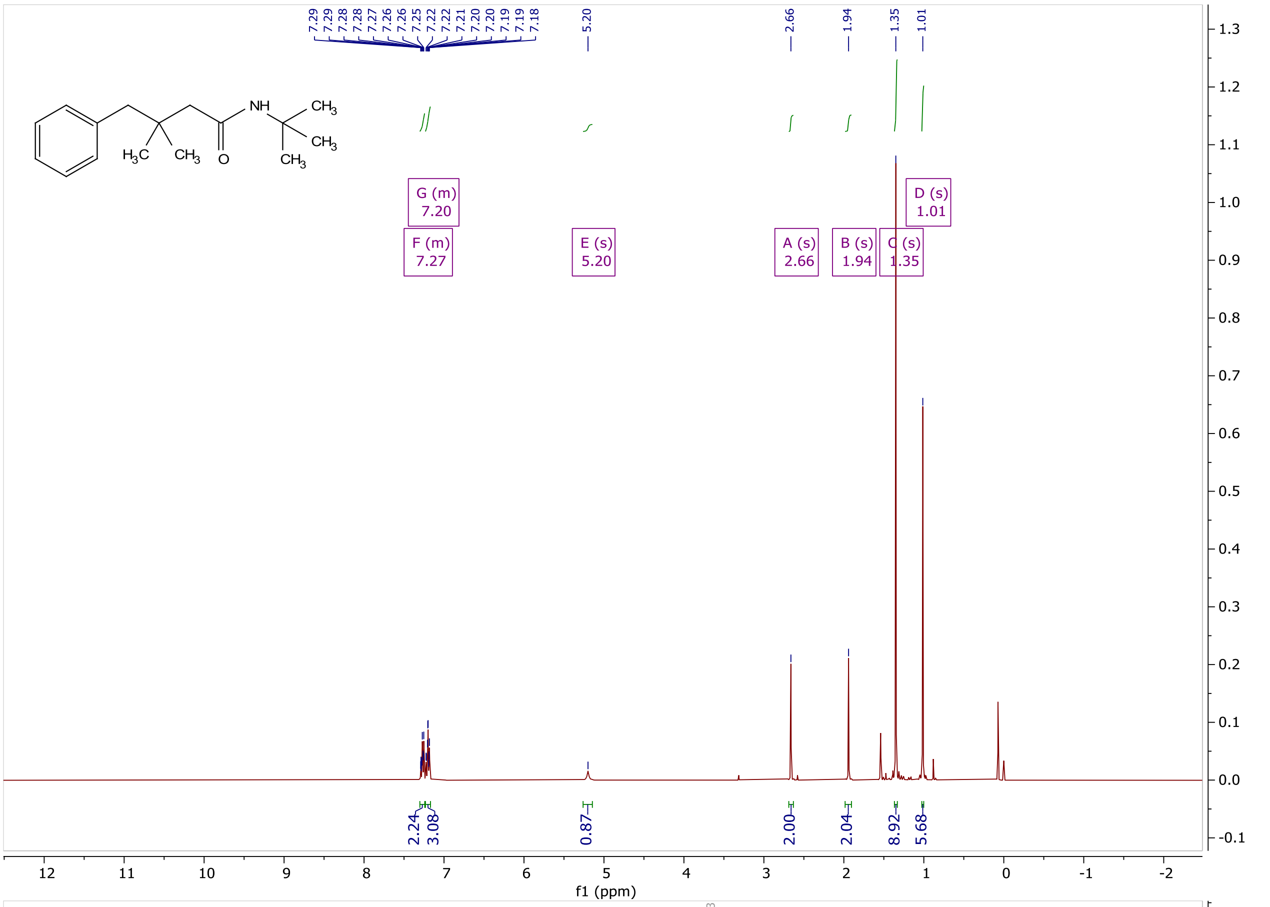

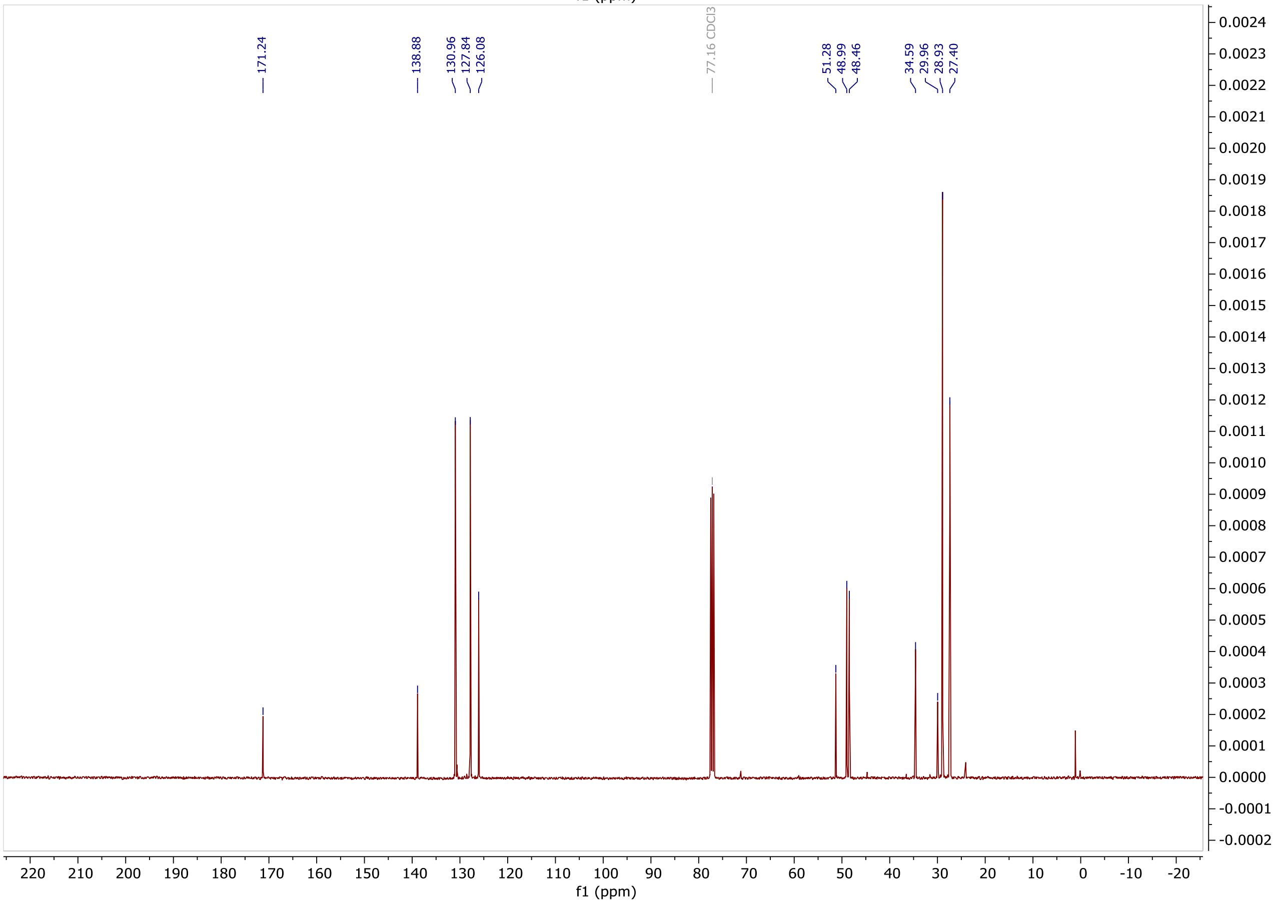

4-Phenyl-*N*-(2,4,4-trimethylpentan-2-yl)butanamide (8).

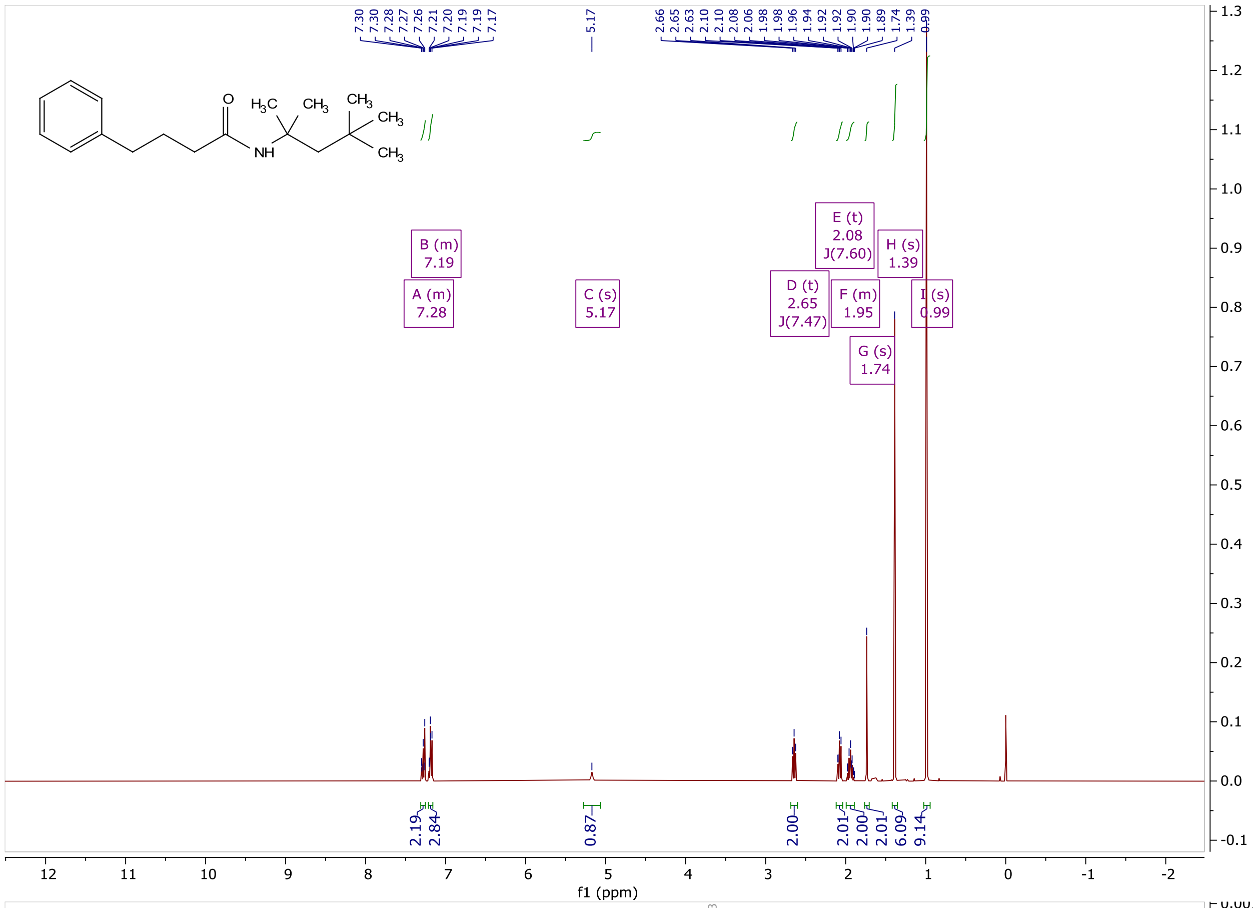

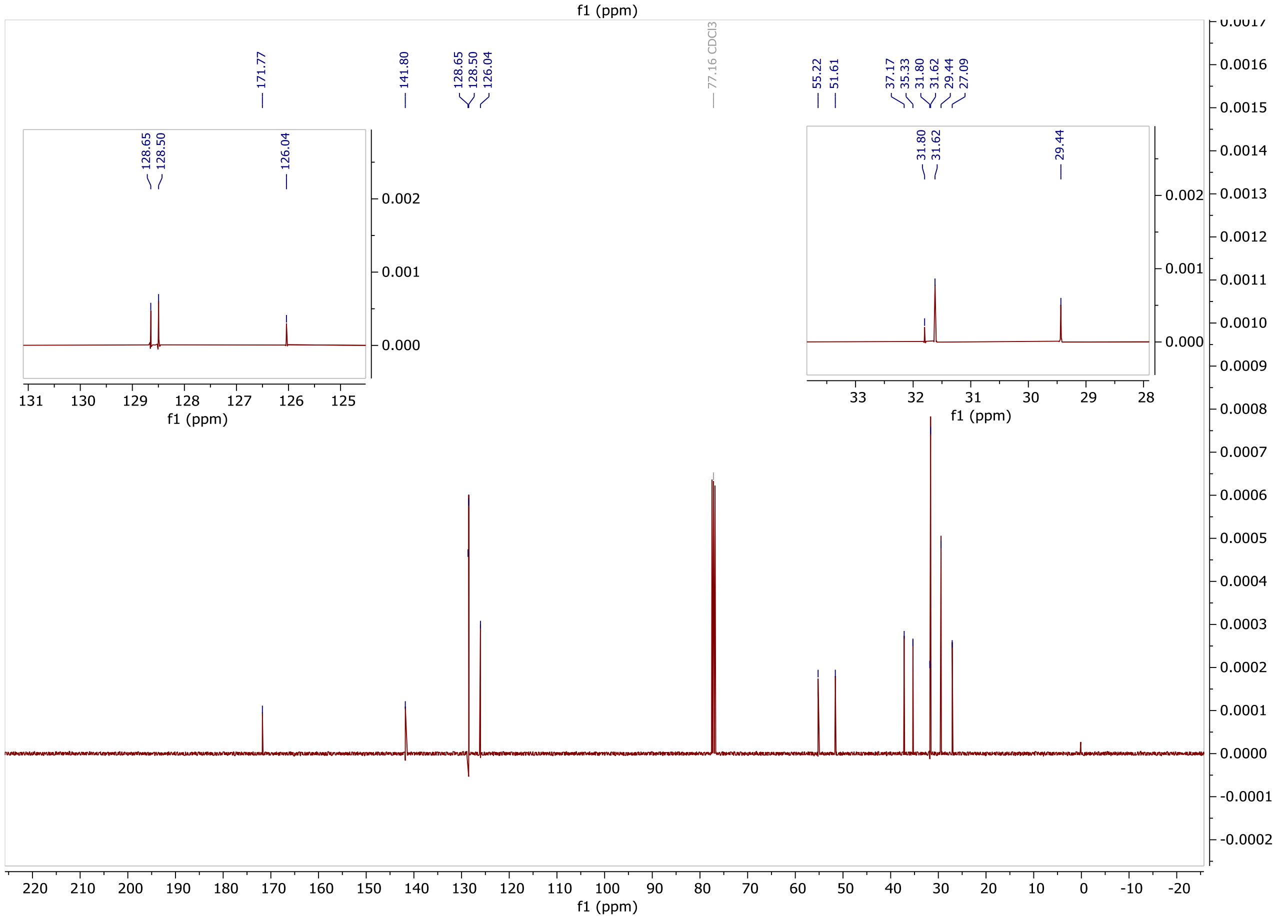

2-Methyl-4-phenyl-*N*-(2,4,4-trimethylpentan-2-yl)butanamide (9).

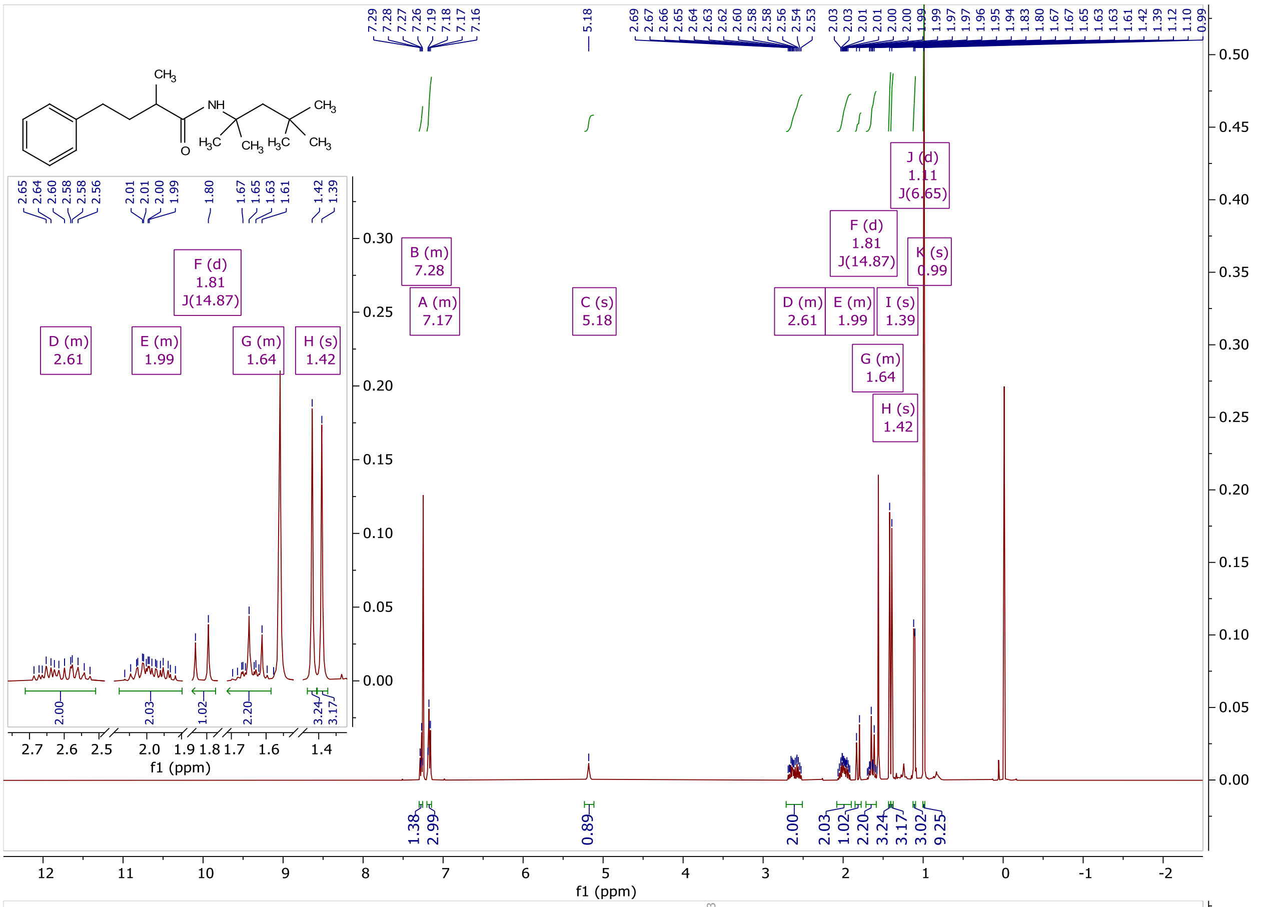

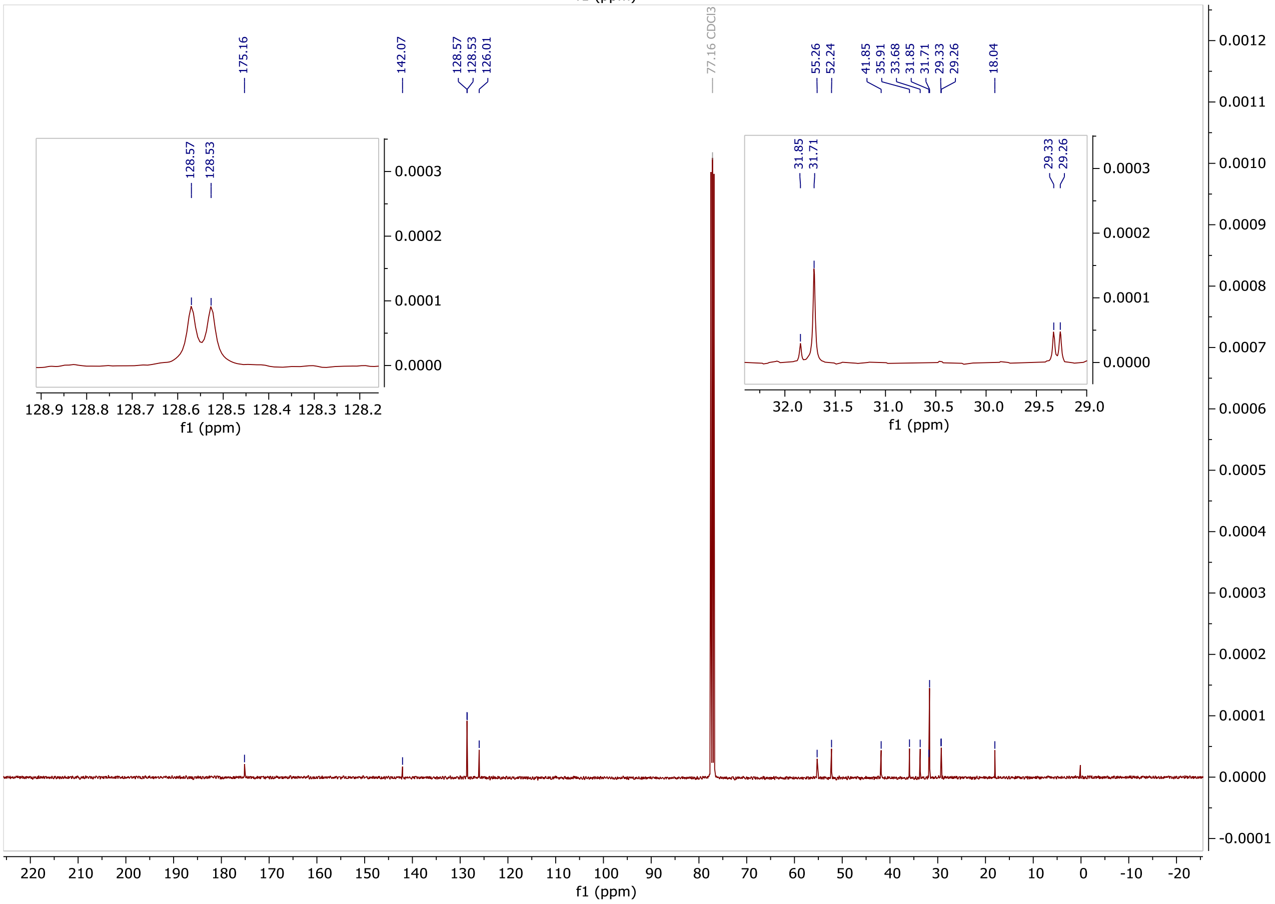

N-Cyclohexyl-2-methyl-4-phenylbutanamide (11).

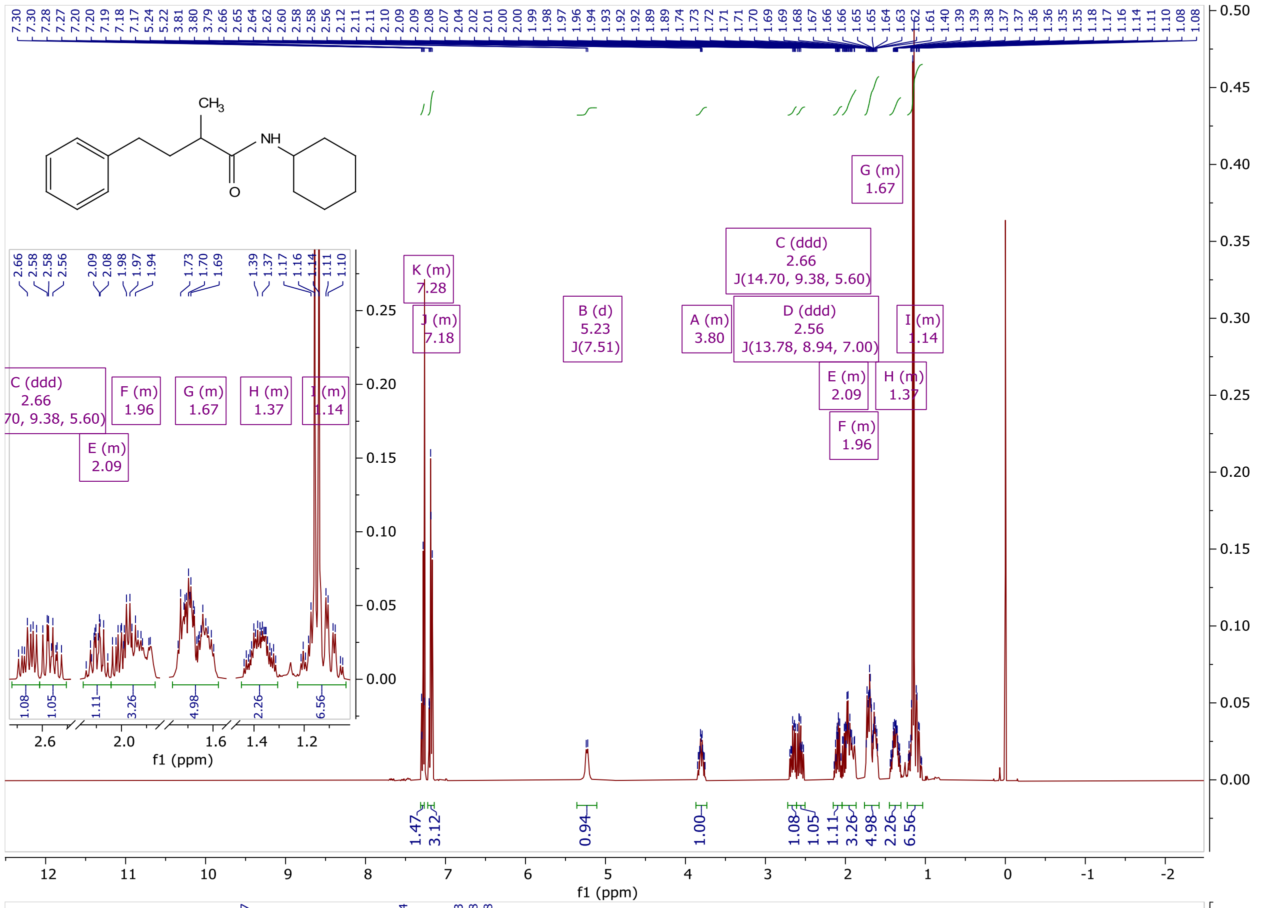

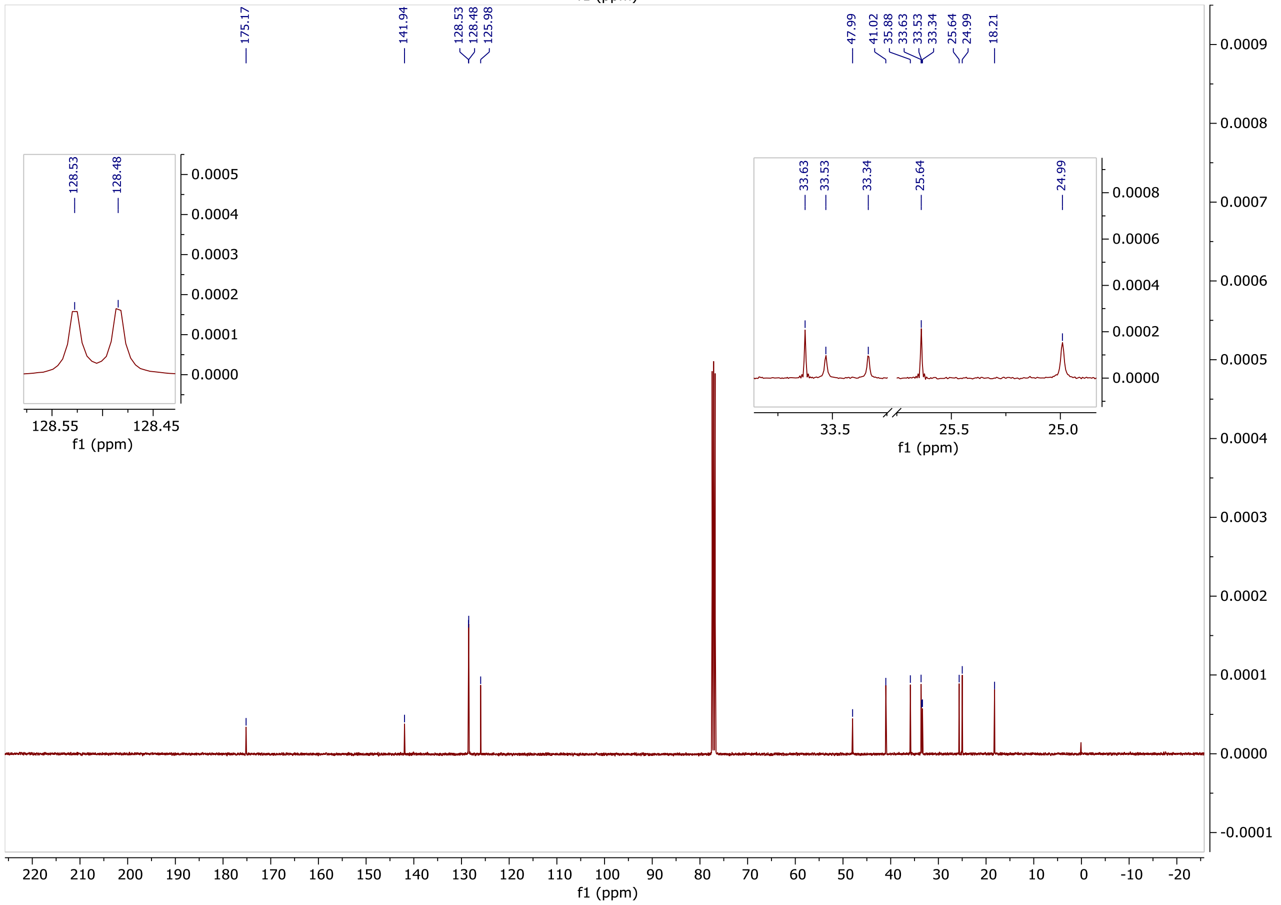

2-Methyl-4-phenyl-*N*-(*p*-tolyl)butanamide (14).

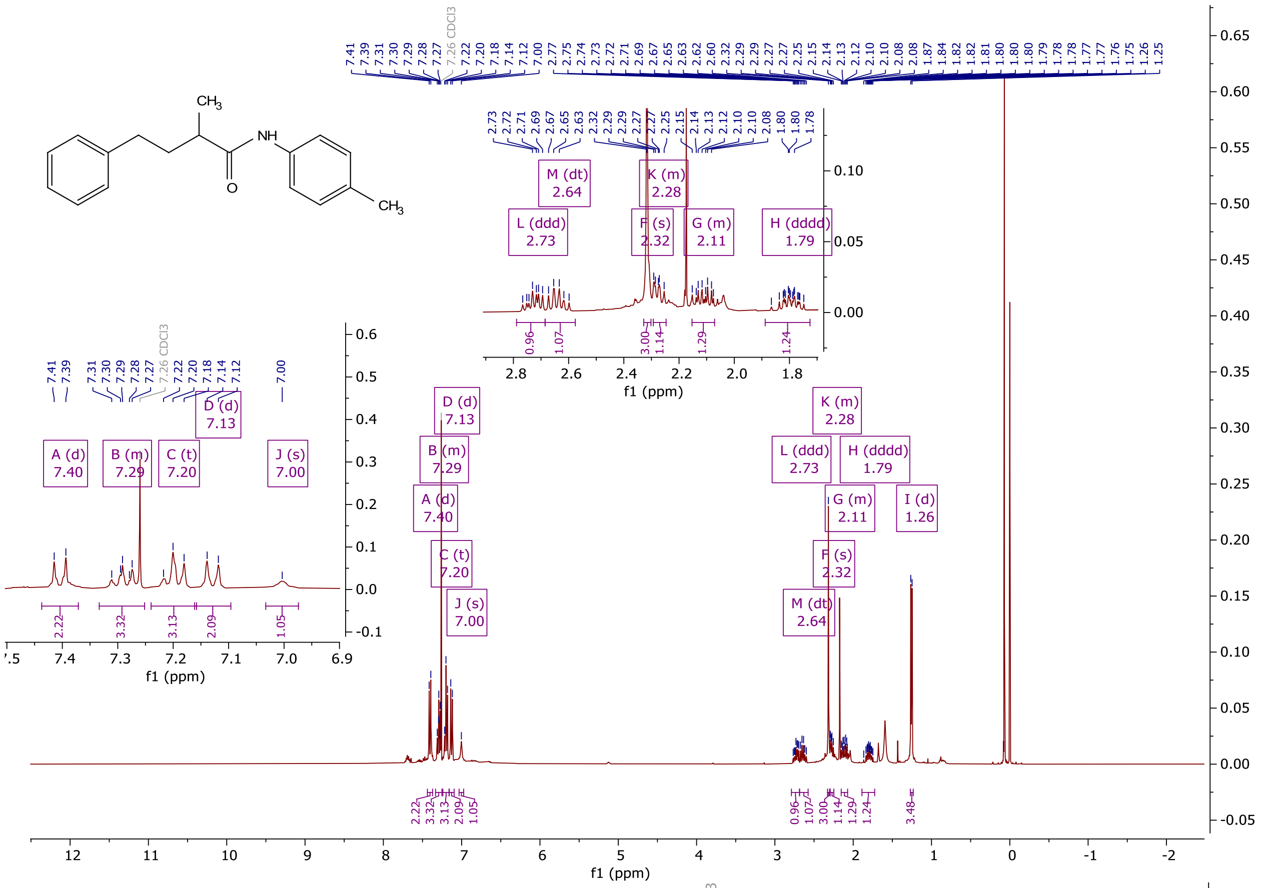

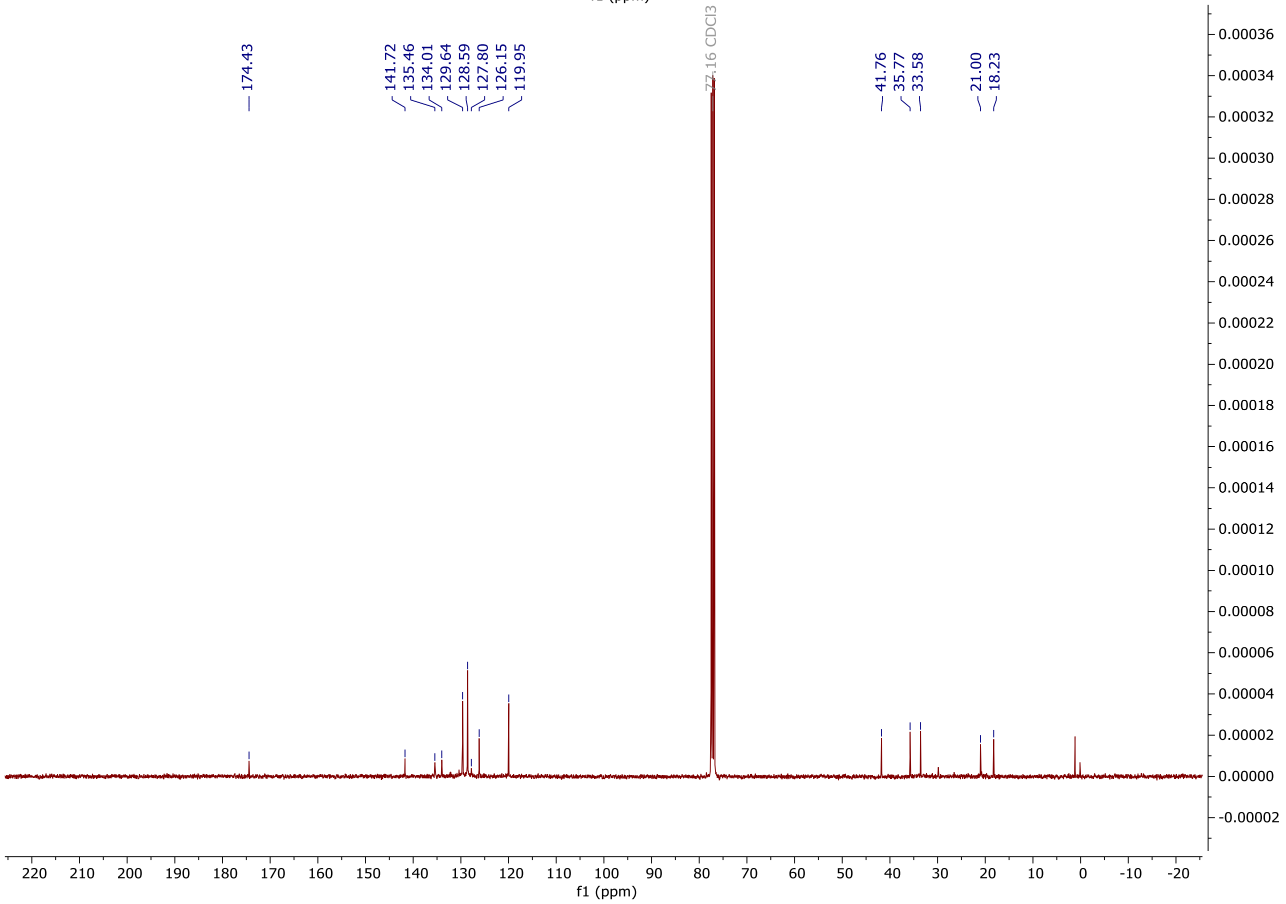

_N_-(2,6-Dimethylphenyl)-2-methyl-4-phenylbutanamide (15).

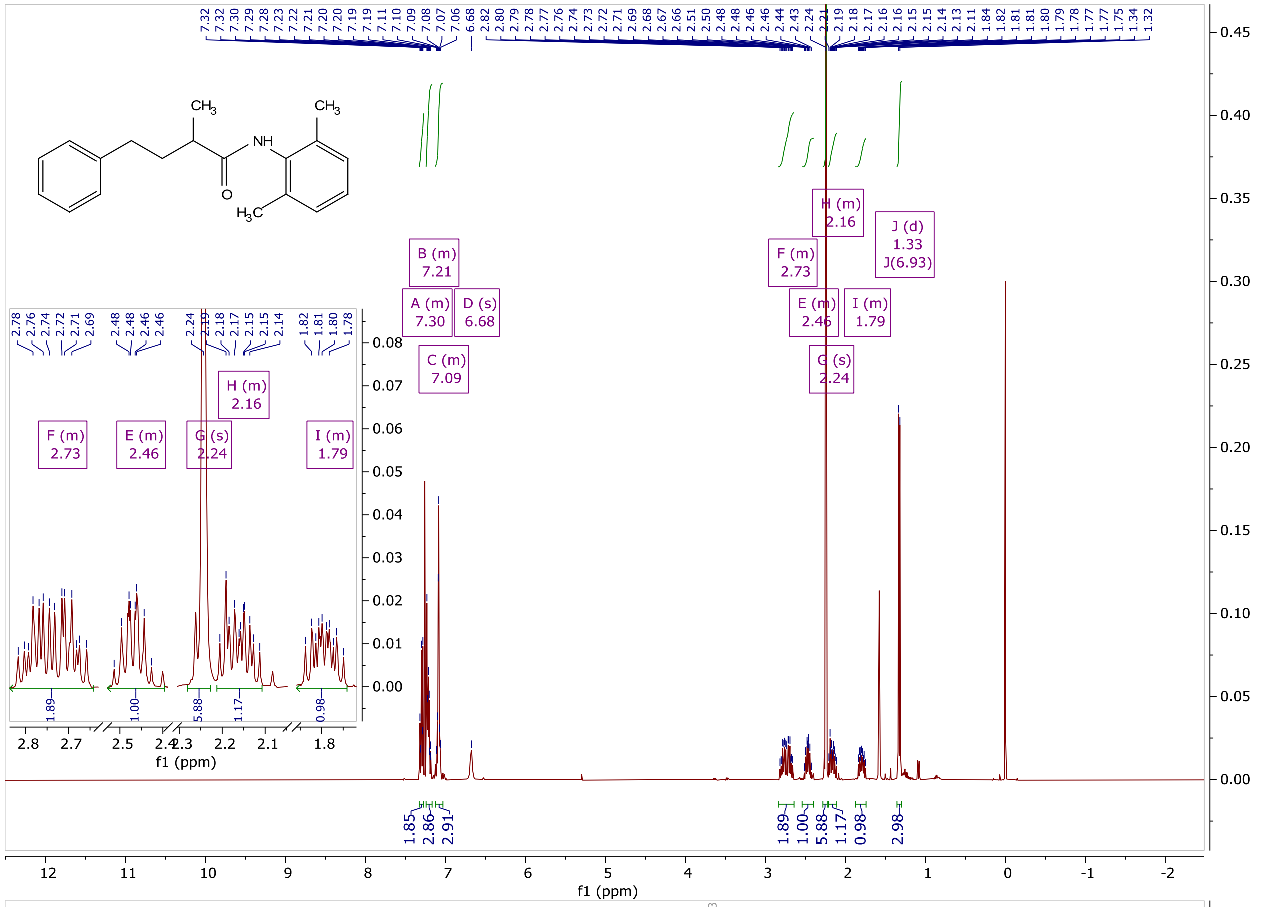

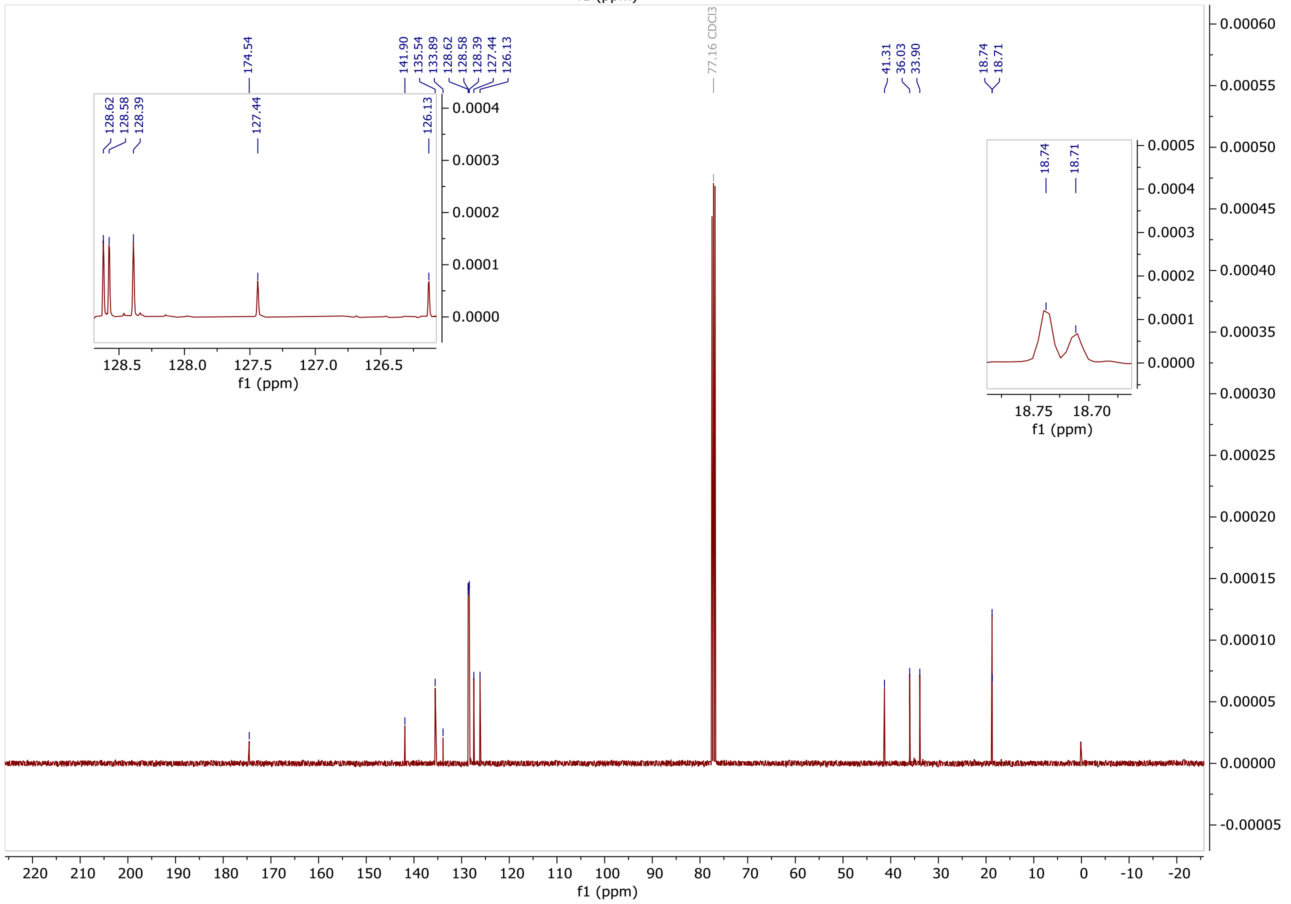

N-Cyclohexyl-3,3-dimethyl-4-phenylbutanamide (16).

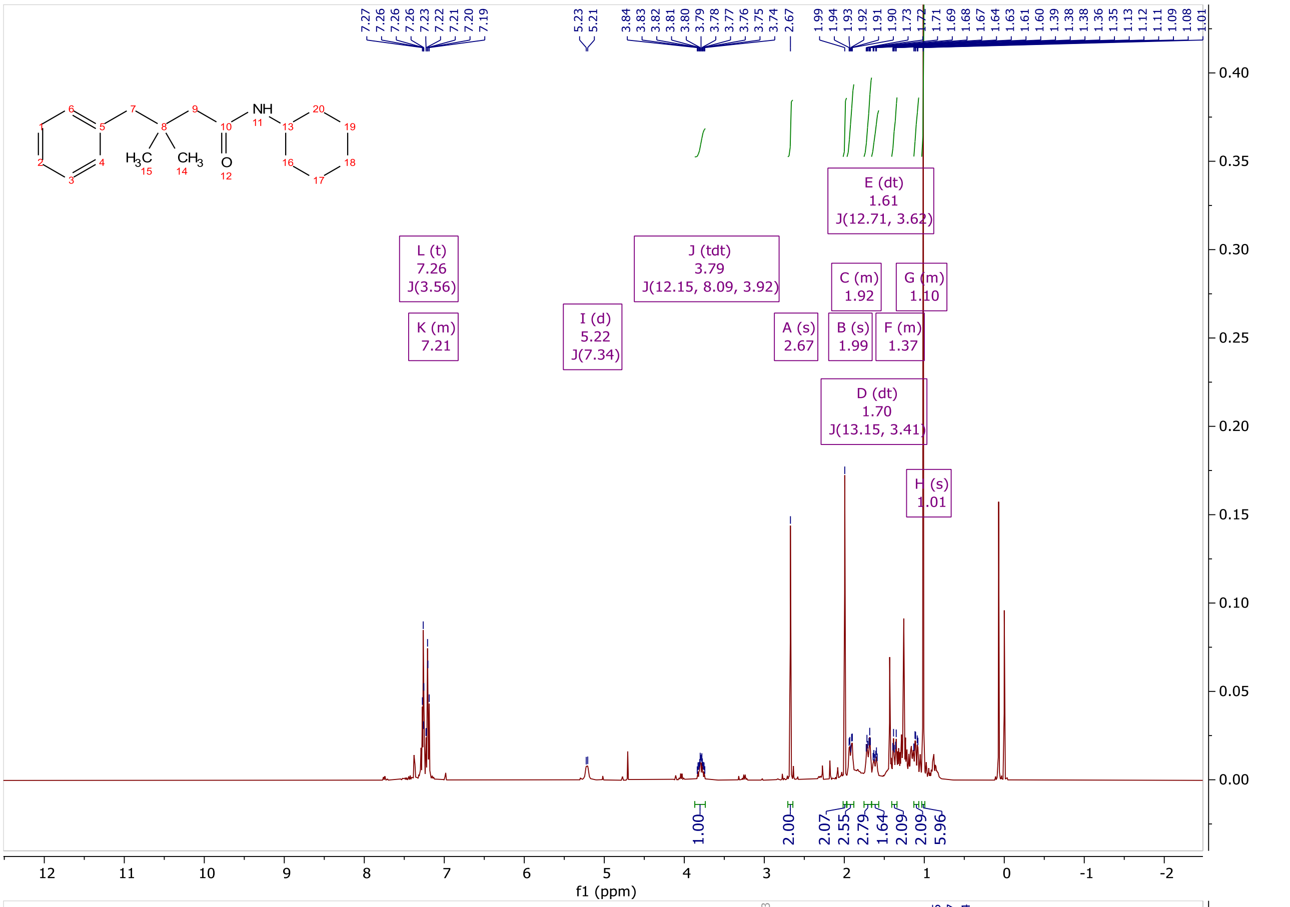

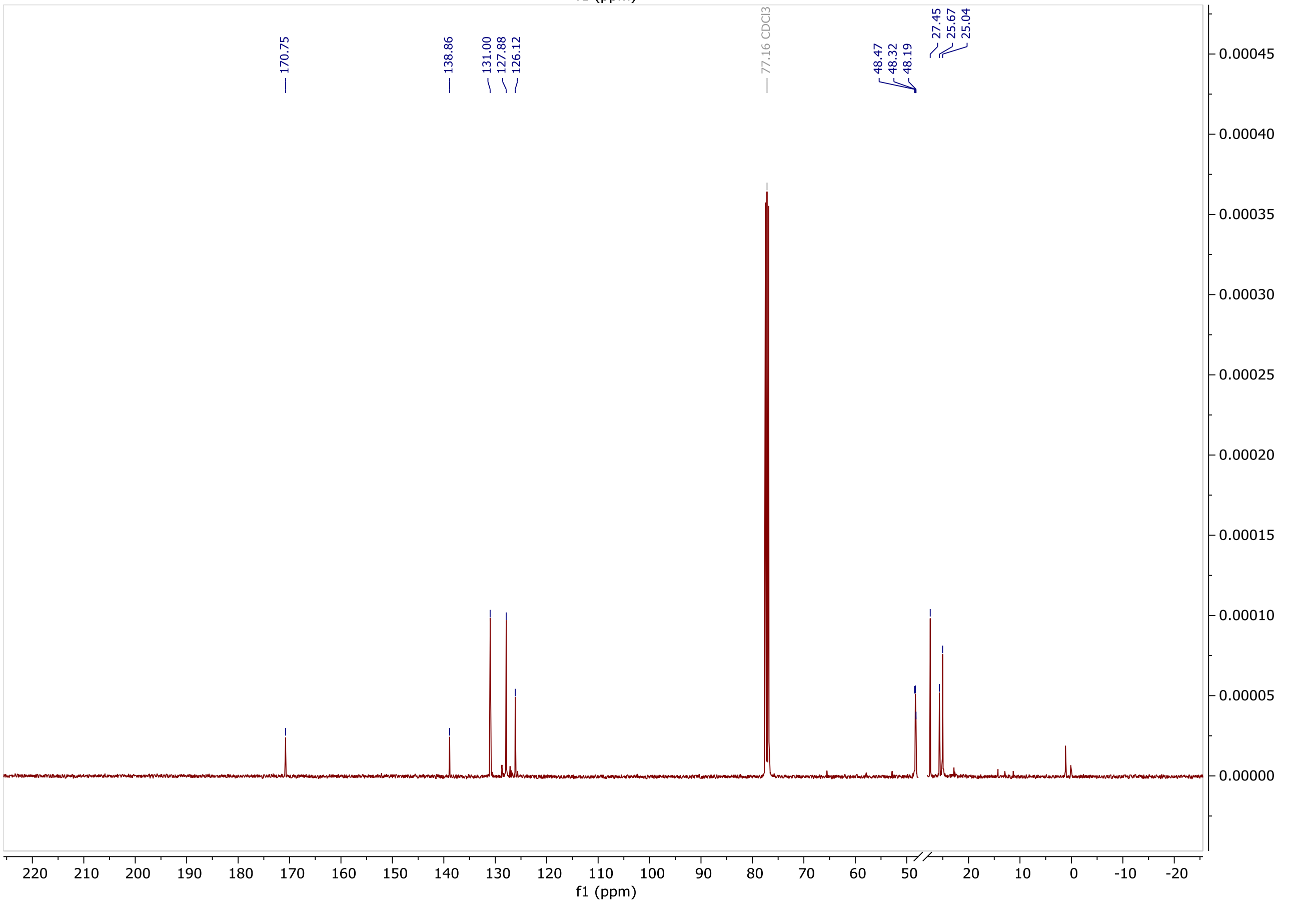

***N*-Cyclohexyl-2-oxo-5-phenylpentanamide (17).**

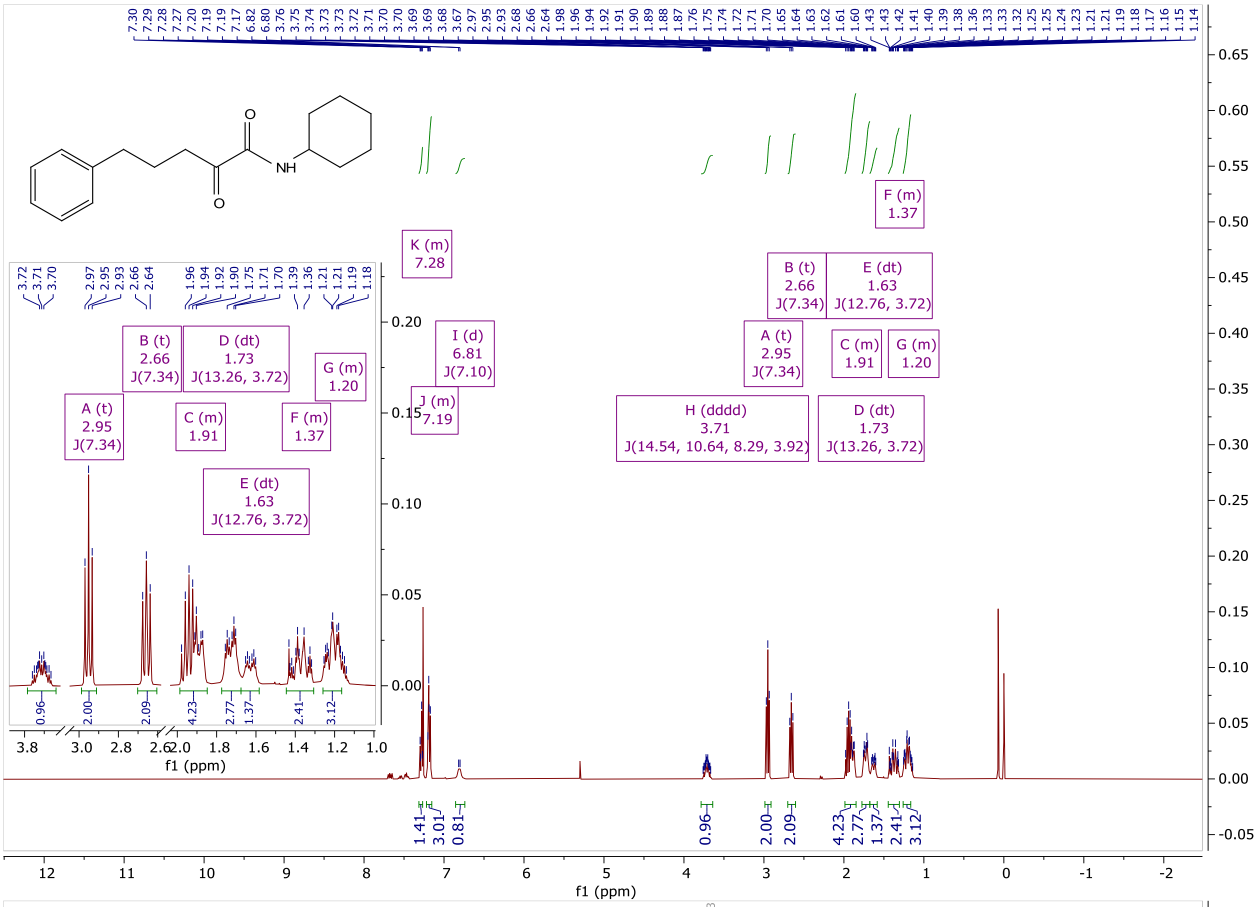

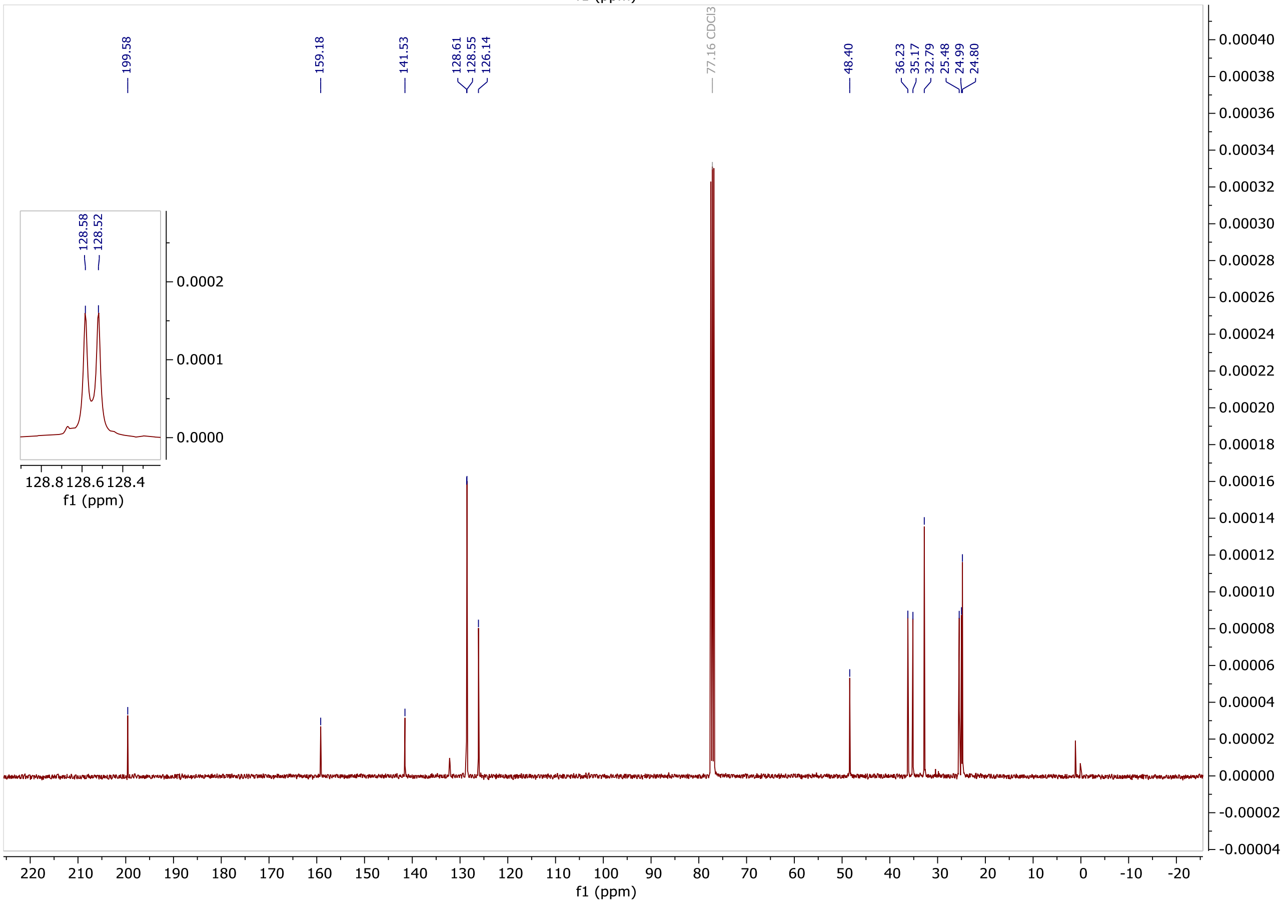

2-Oxo-*N*-phenethyl-5-phenylpentanamide (18).

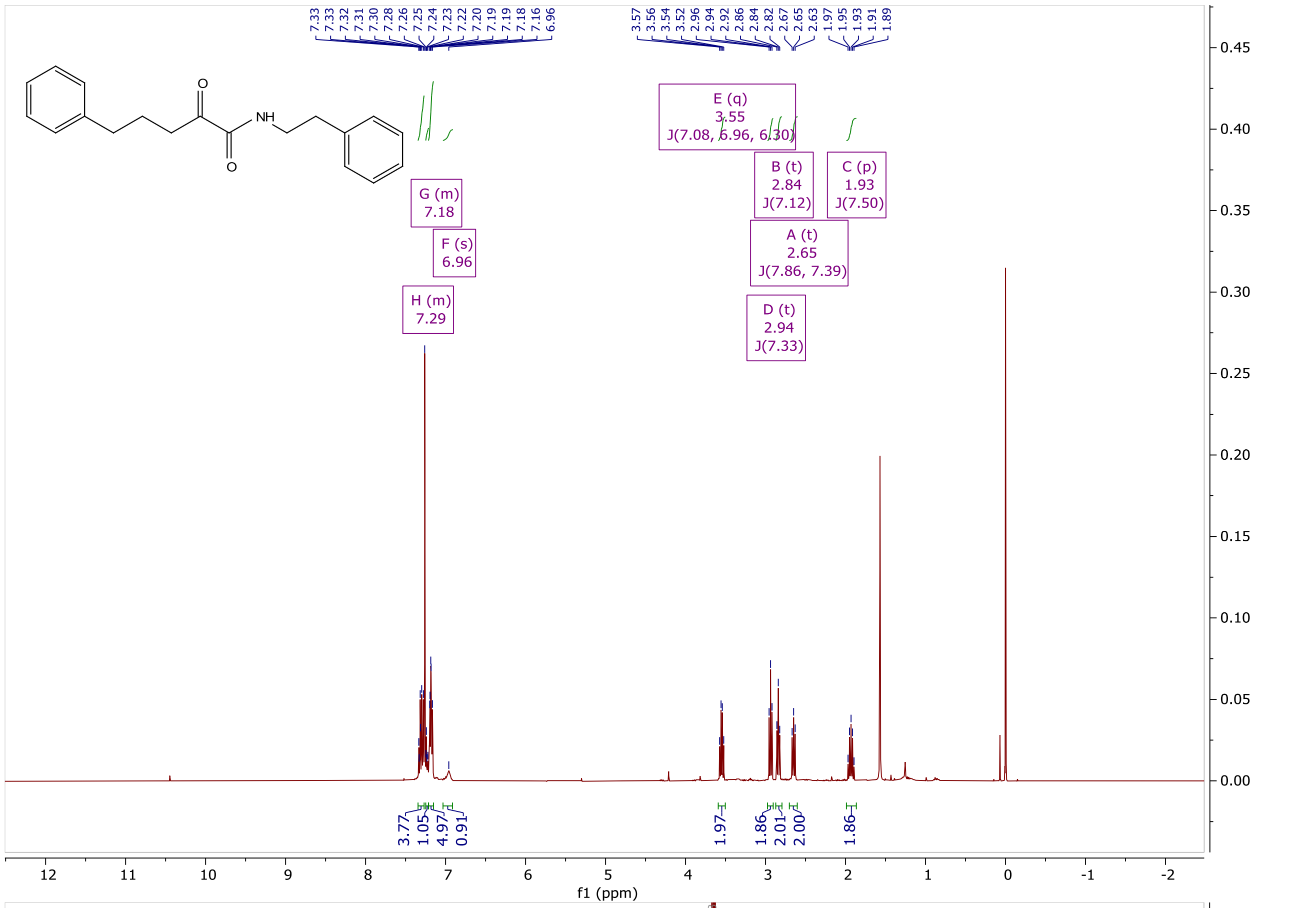

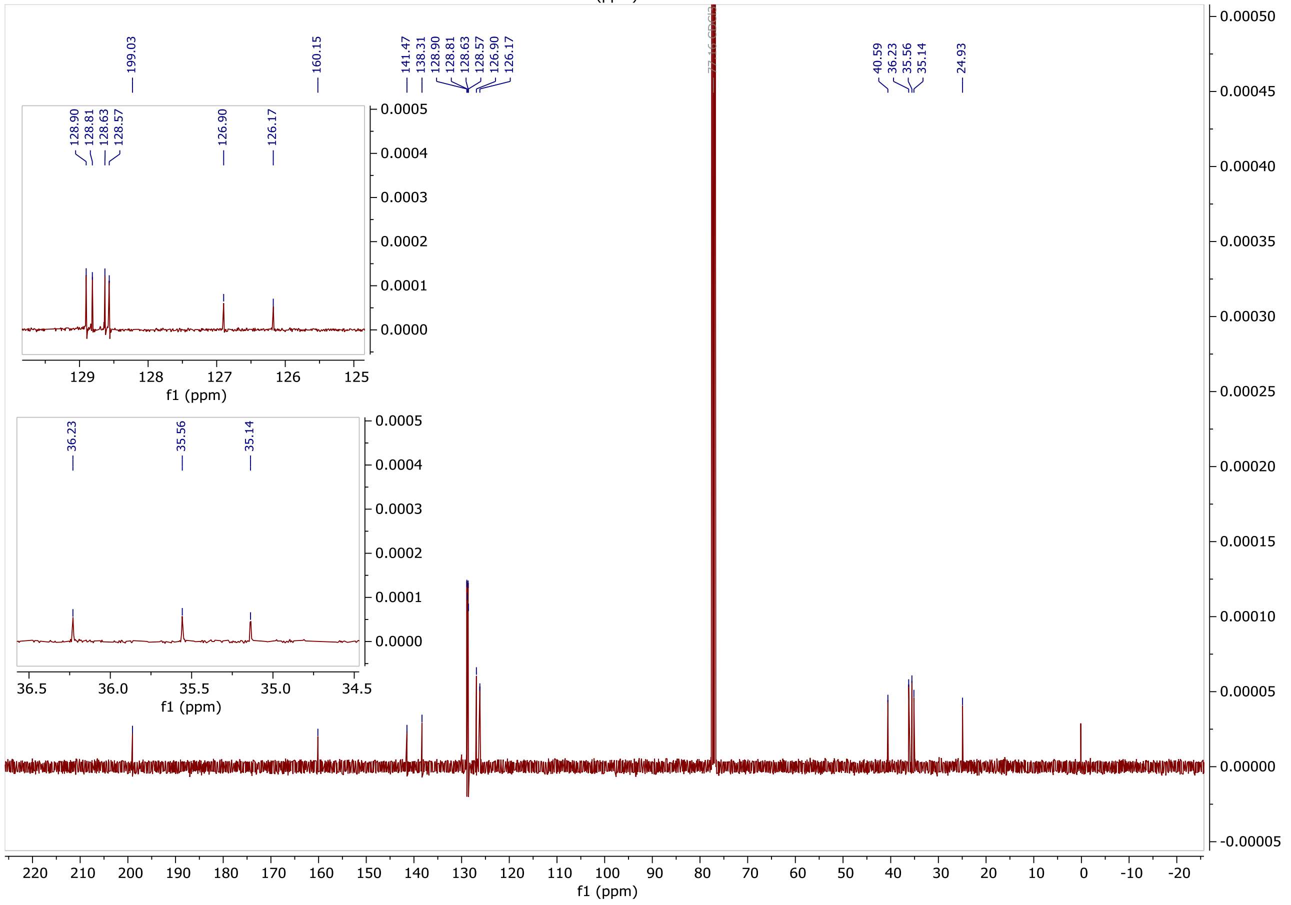

3-Methyl-2-oxo-*N*-phenethyl-5-phenylpentanamide (19).

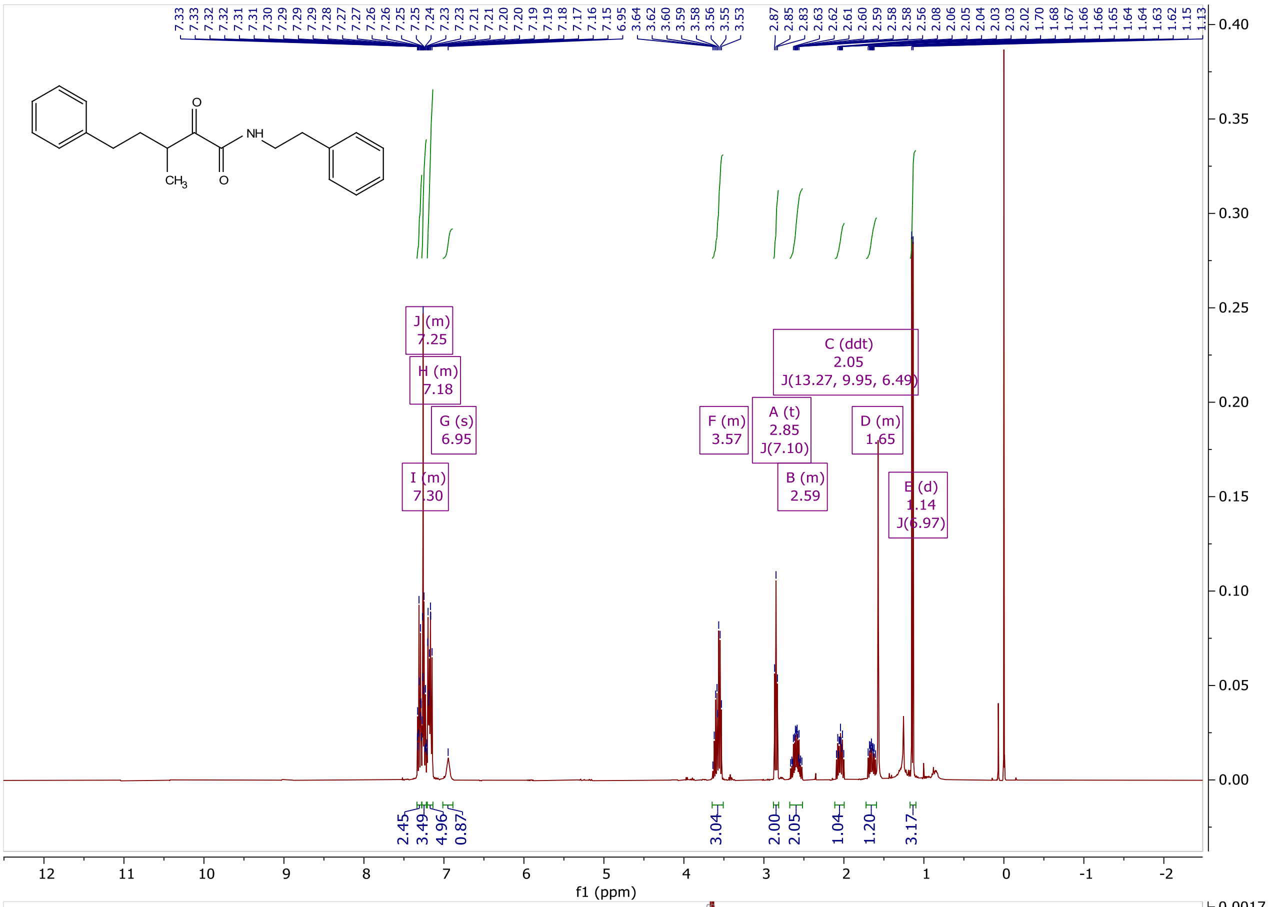

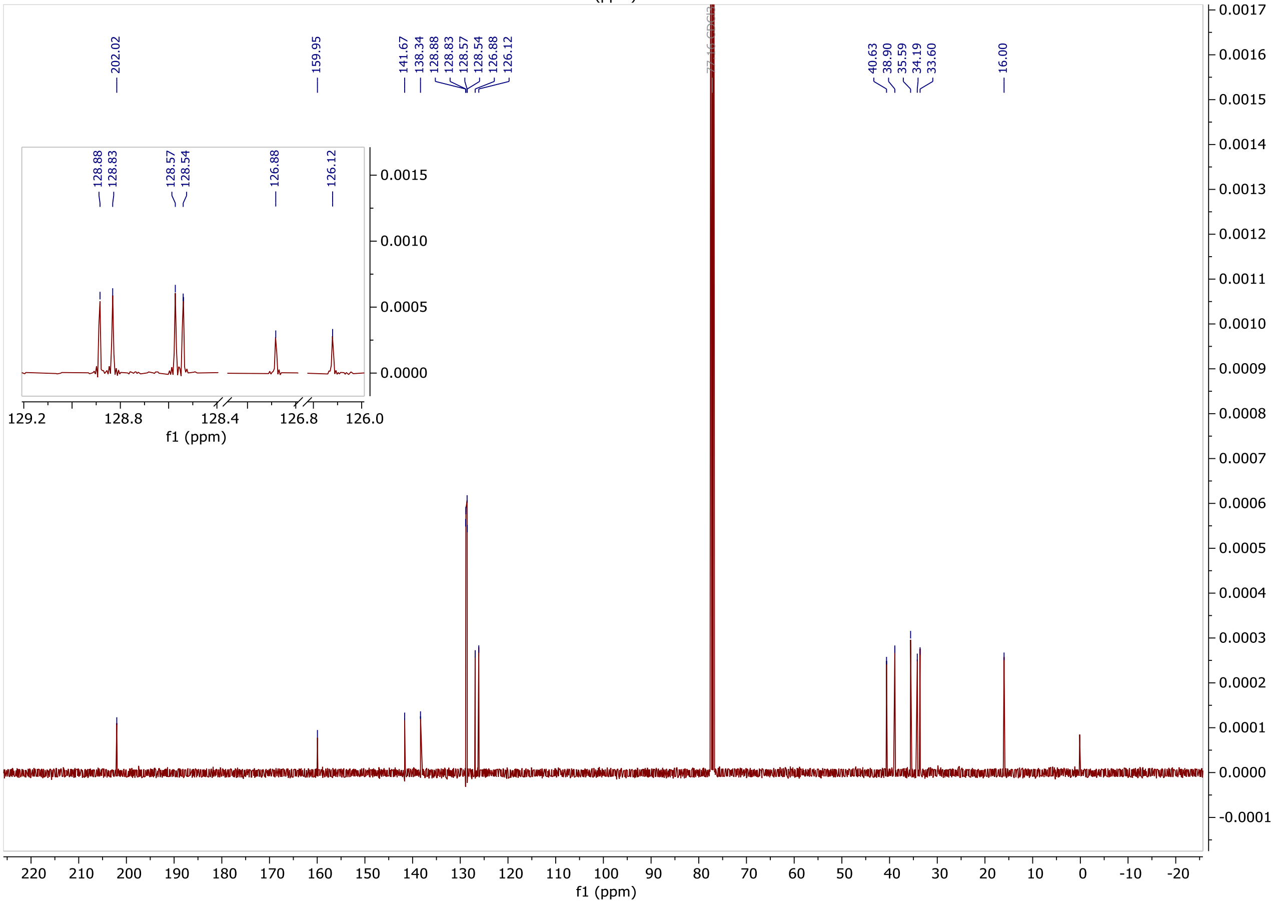

N-Cyclohexyl-2-methyl-4-(thiophen-2-yl)butanamide (21).

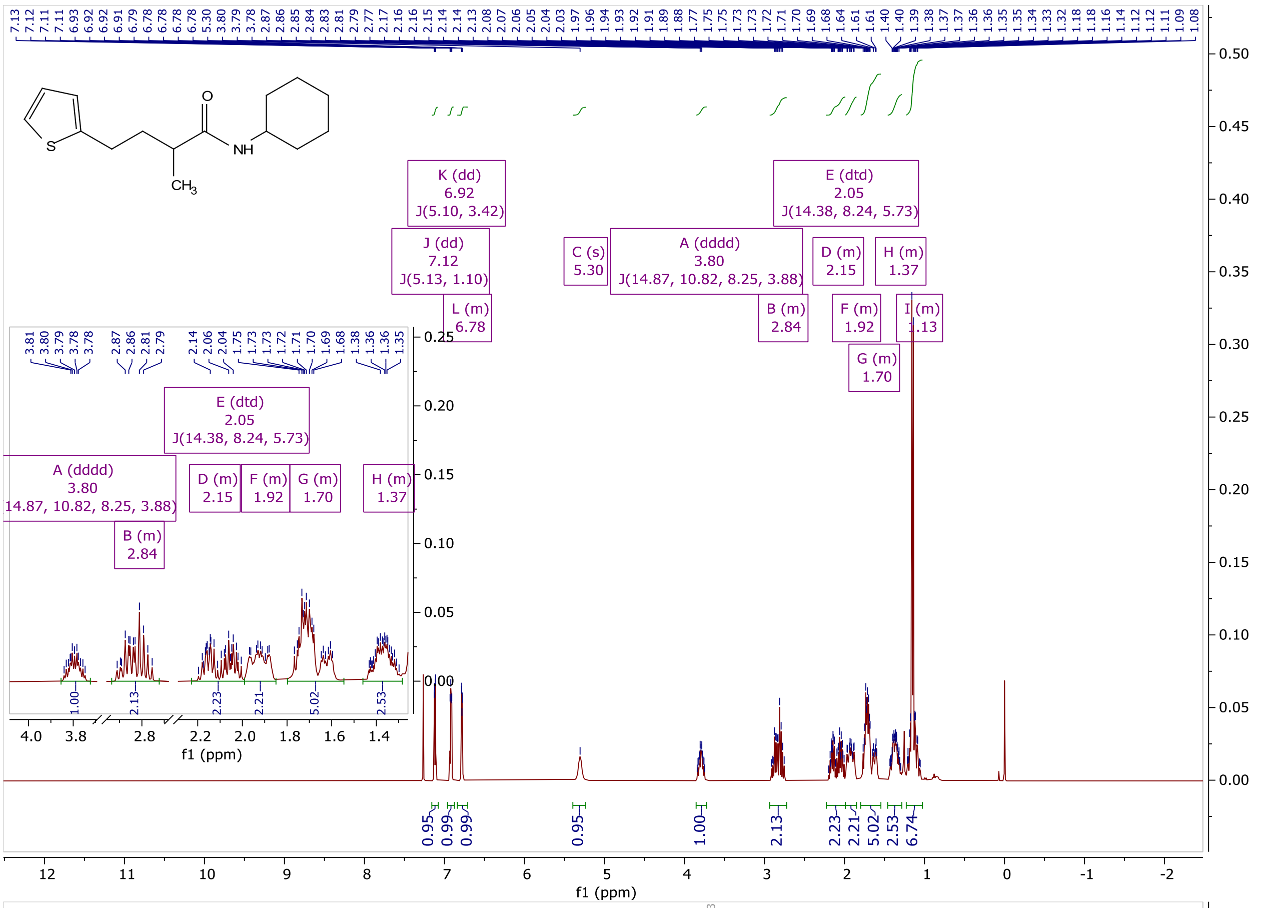

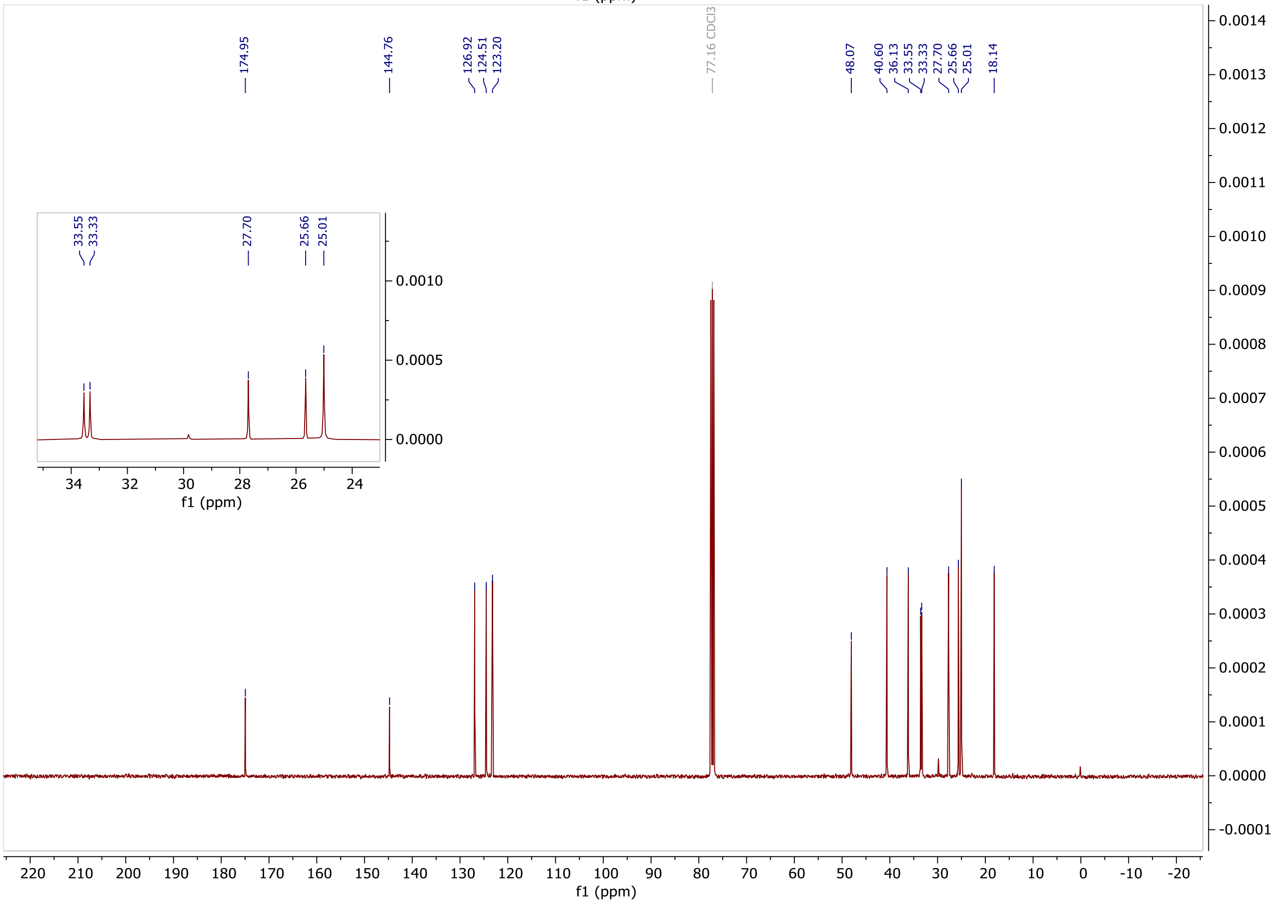

N-(tert-Butyl)-2-methyl-4-(thiophen-2-yl)butanamide (22).

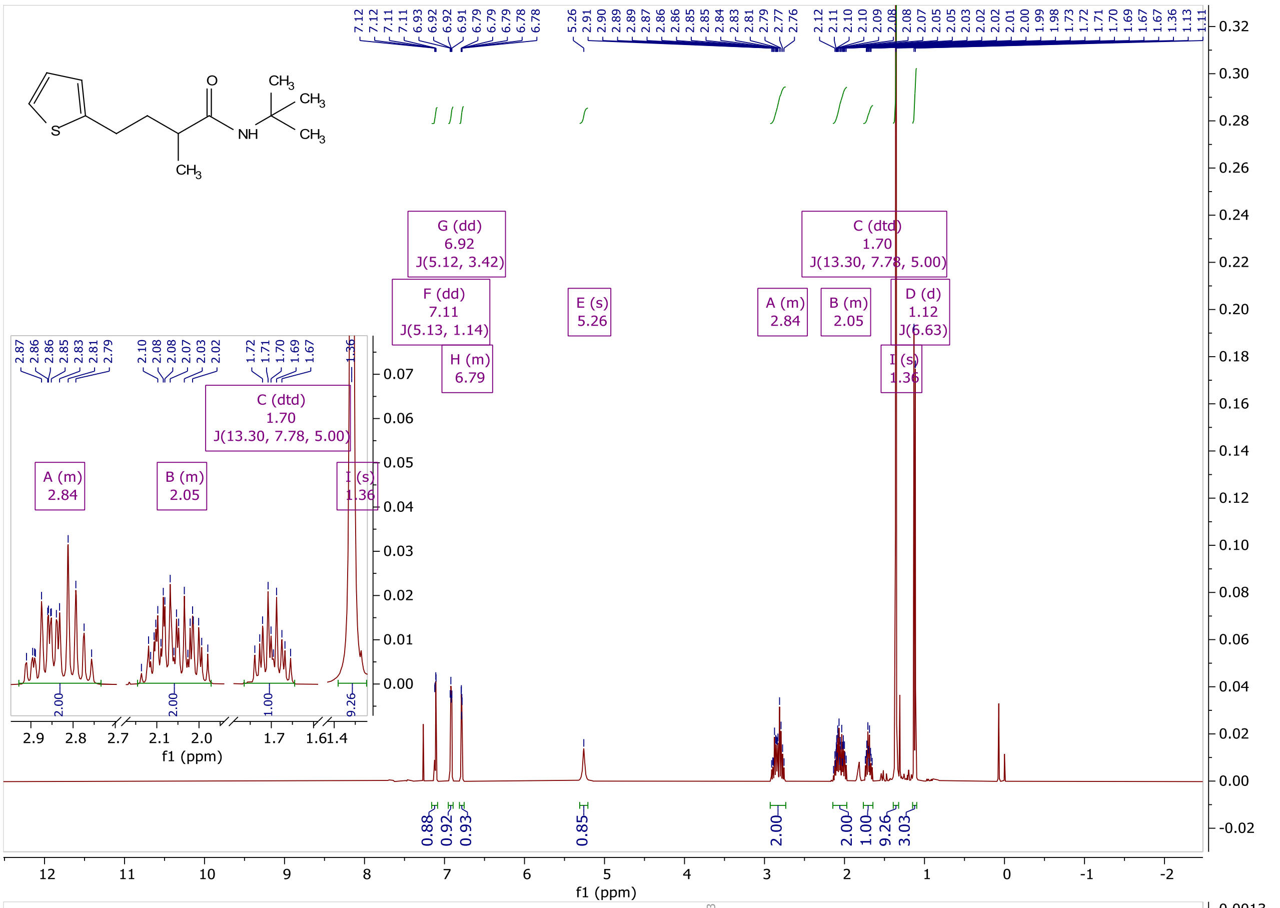

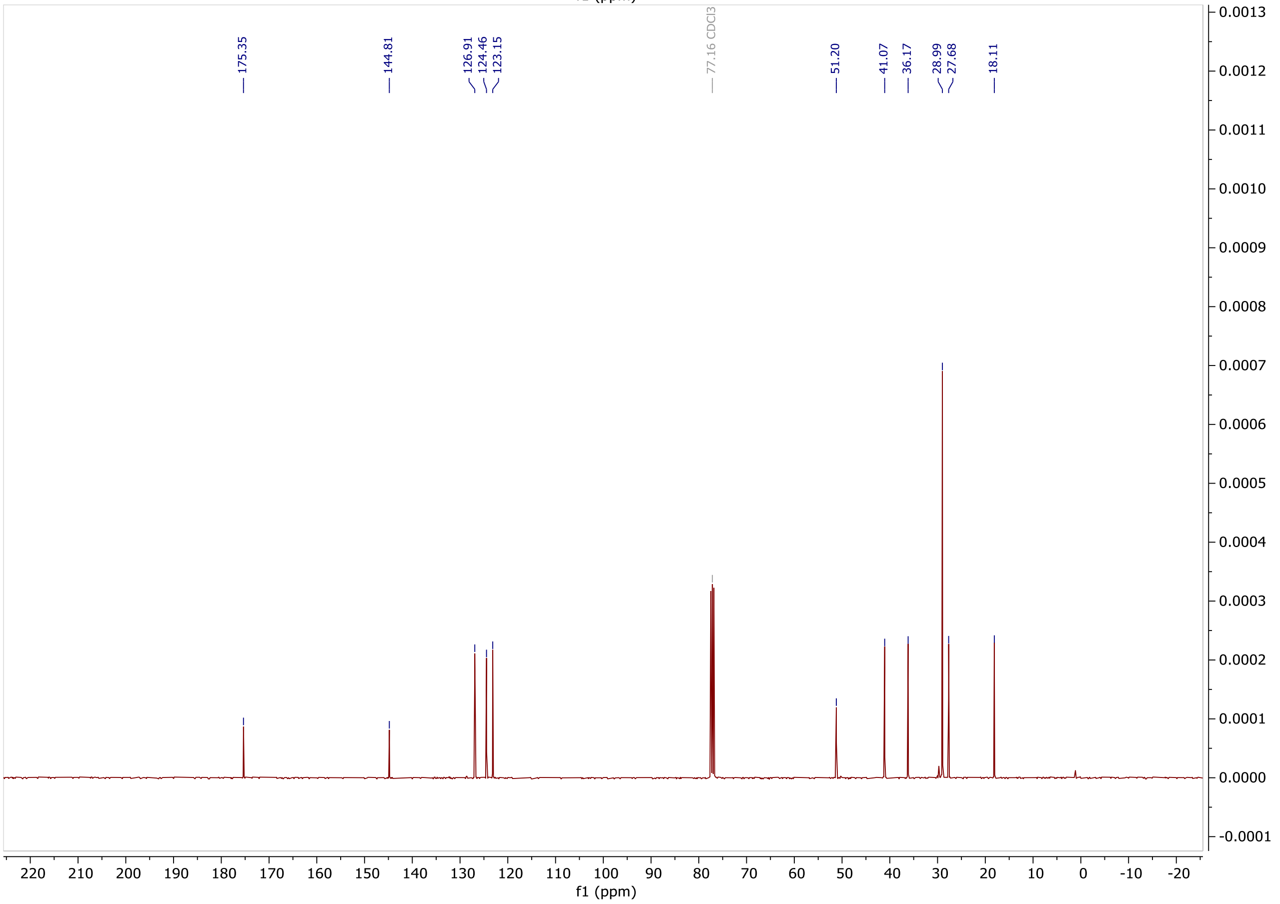

_N_-(_tert_-Butyl)-4-(4-methoxyphenoxy)-2-methylbutanamide (23).

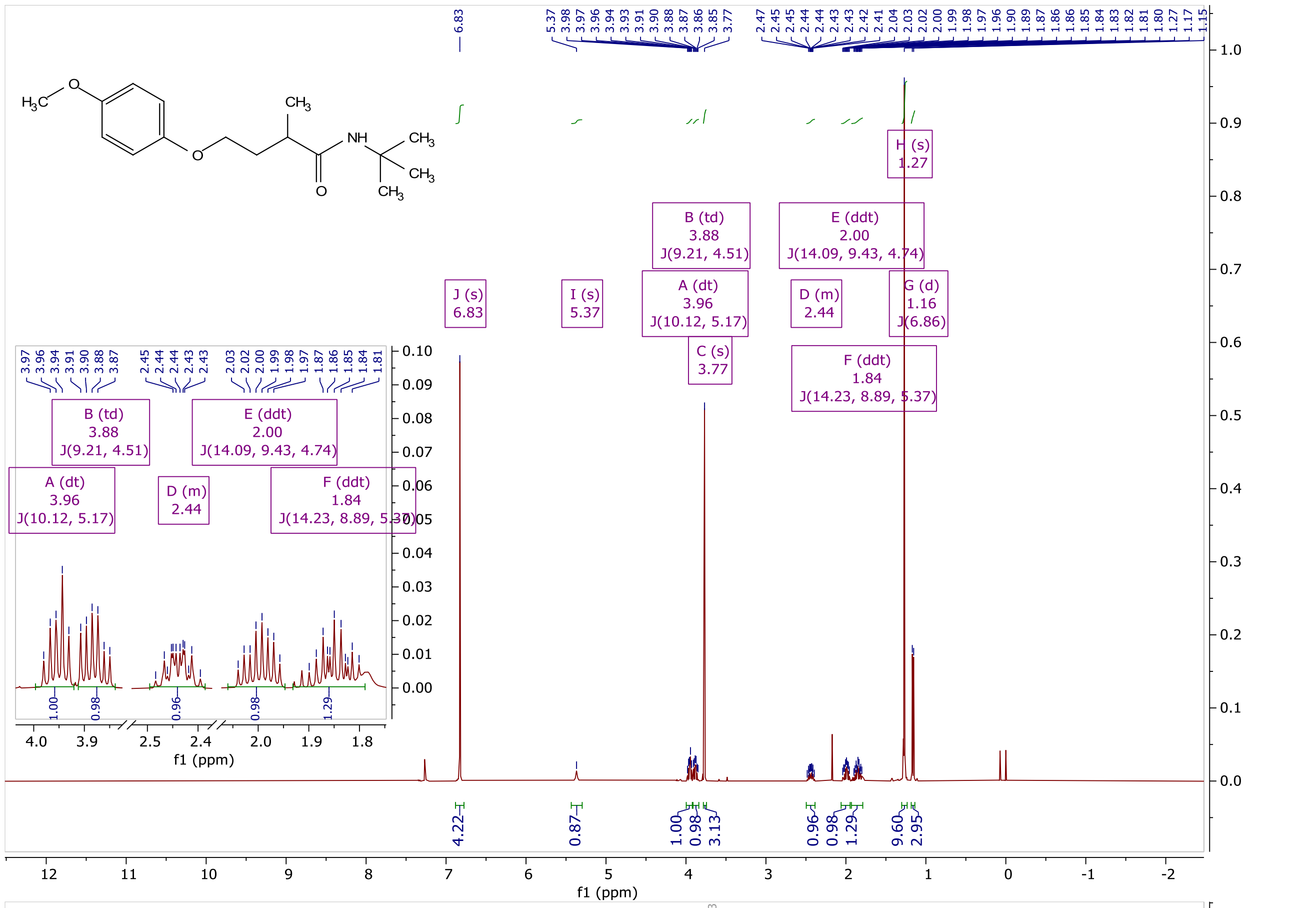

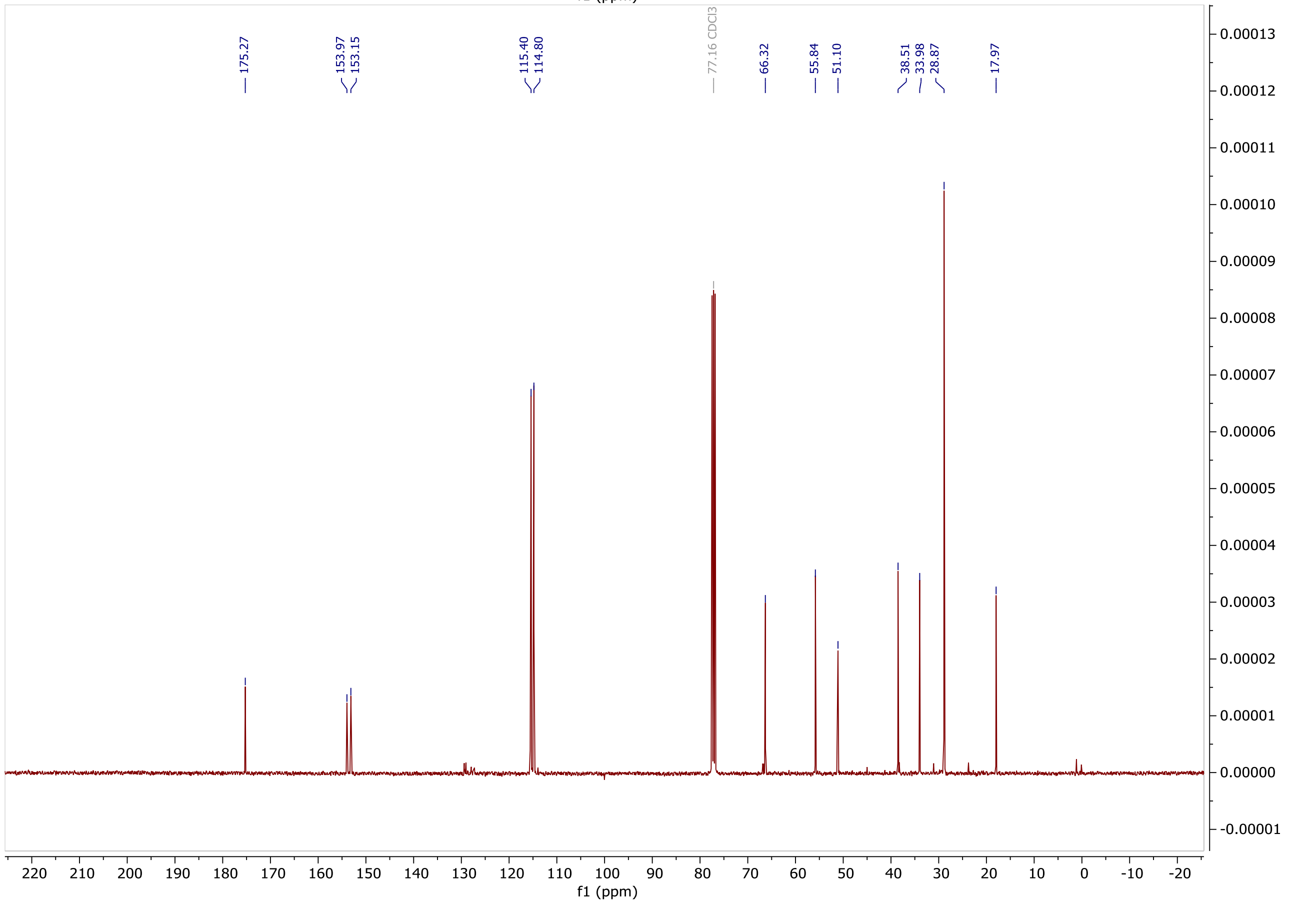

4-(4-Acetamidophenyl)-*N*-(*tert*-butyl)-2-methylbutanamide (24).

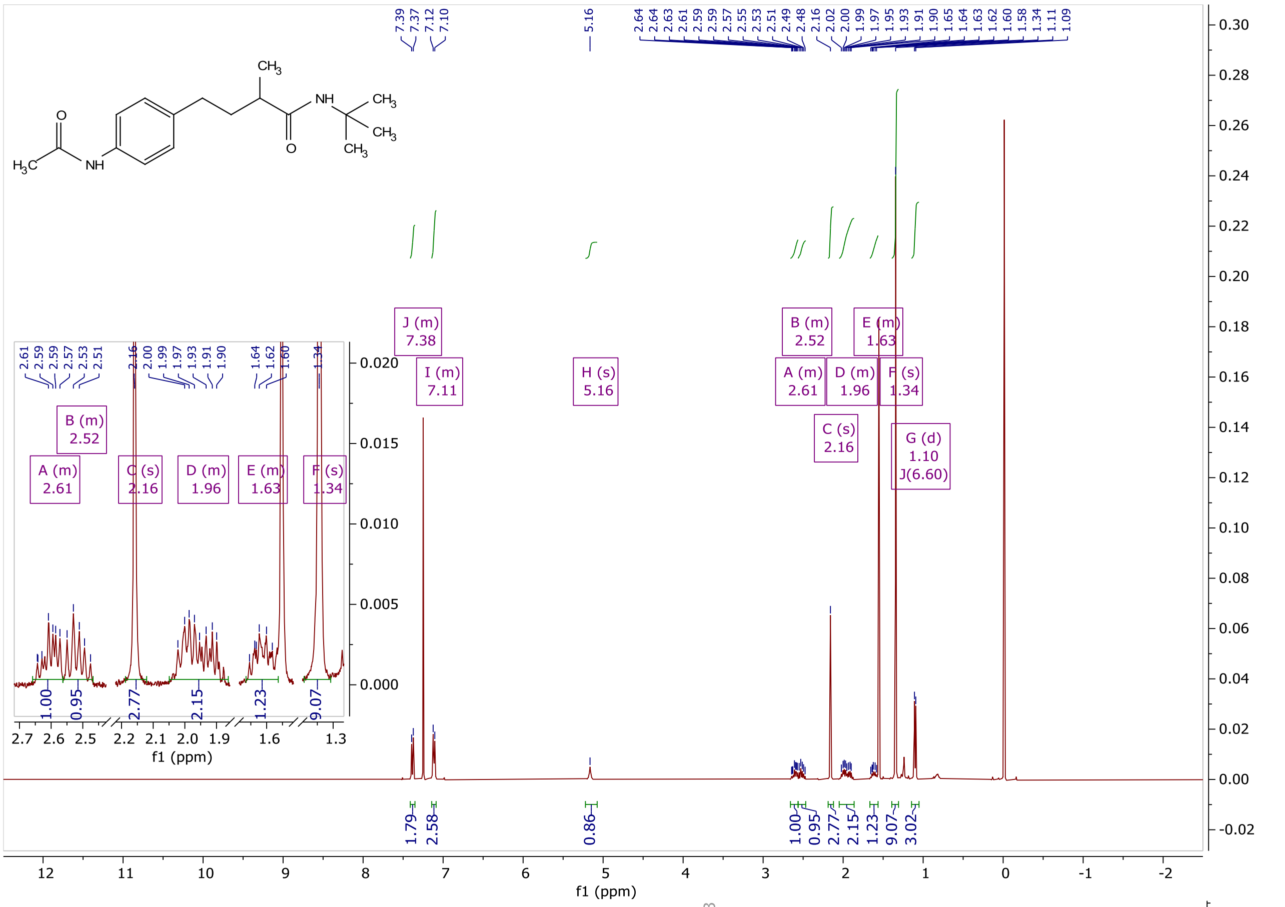

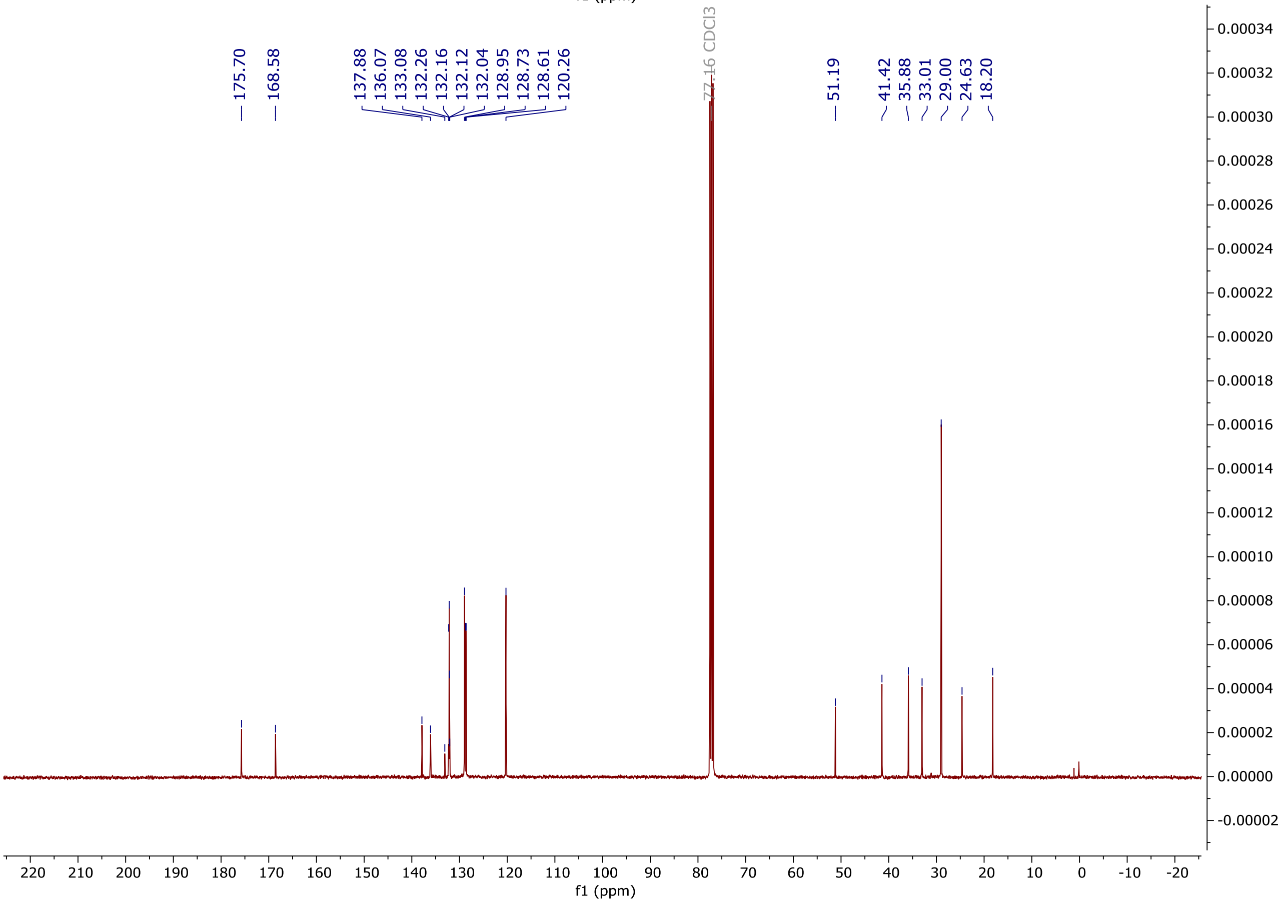

tert-Butyl (4-(4-(_tert_-butylamino)-3-methyl-4-oxobutyl)phenyl) carbamate (25).

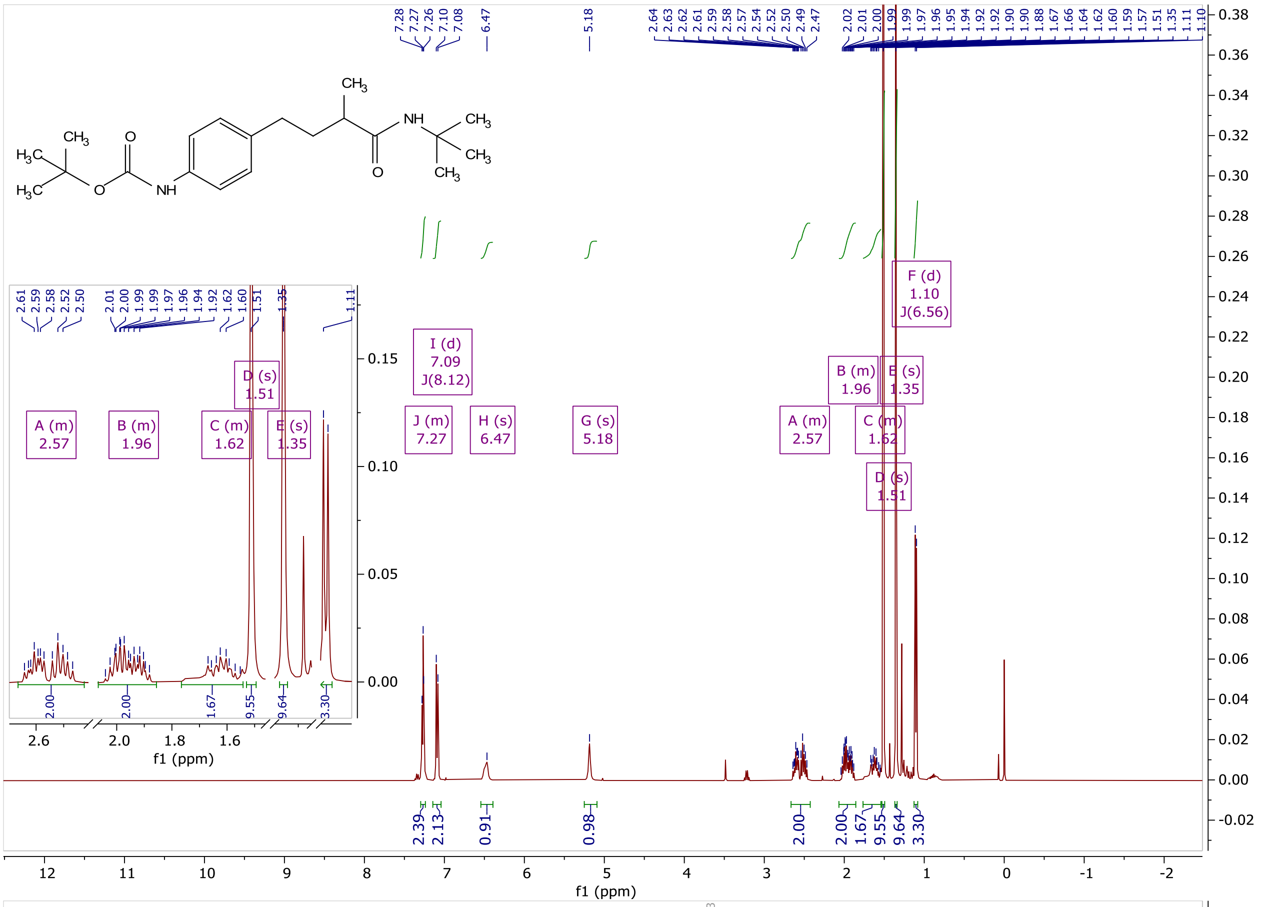

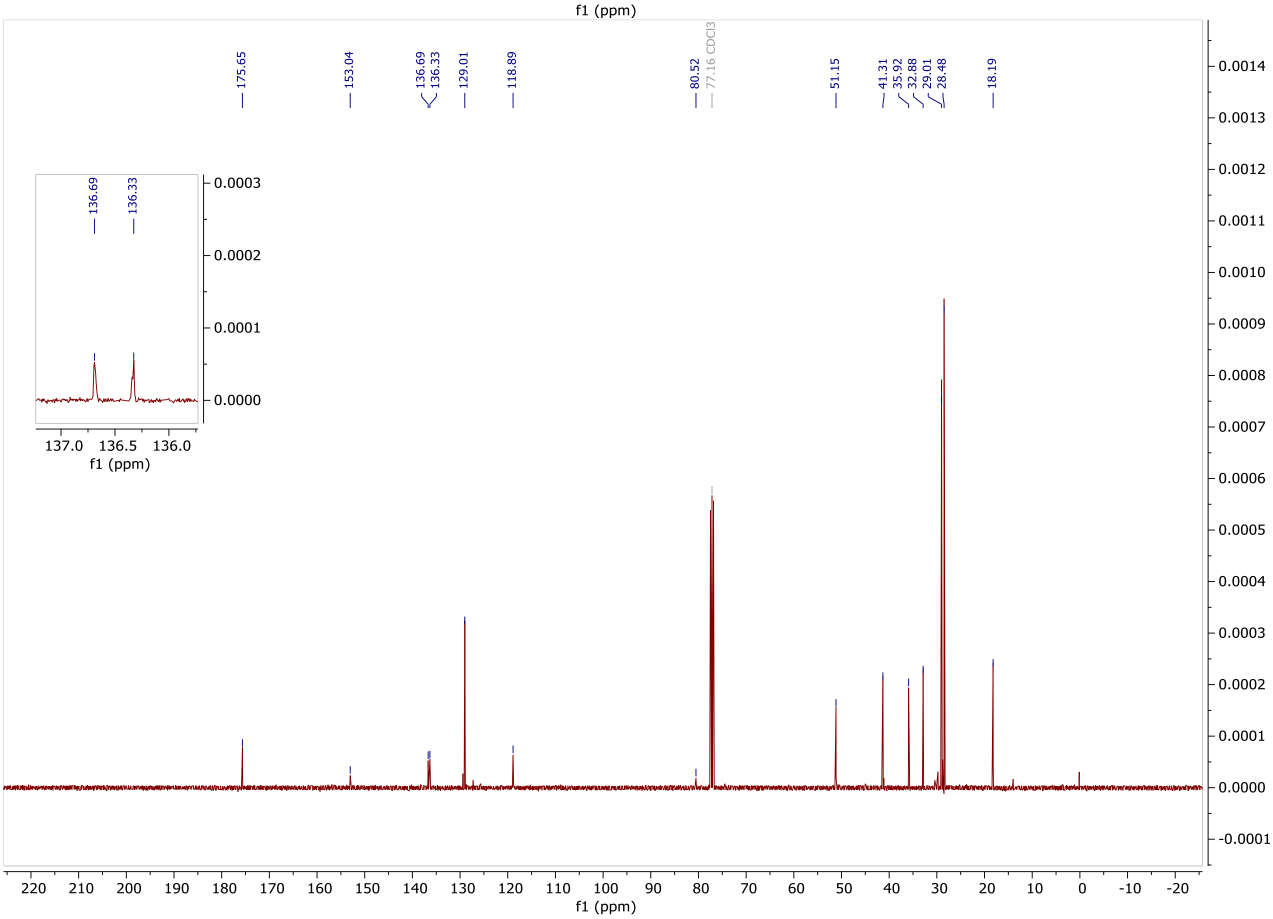

N-(tert-Butyl)-2-methyl-4-(1-methyl-1H-indol-3-yl)butanamide (26).

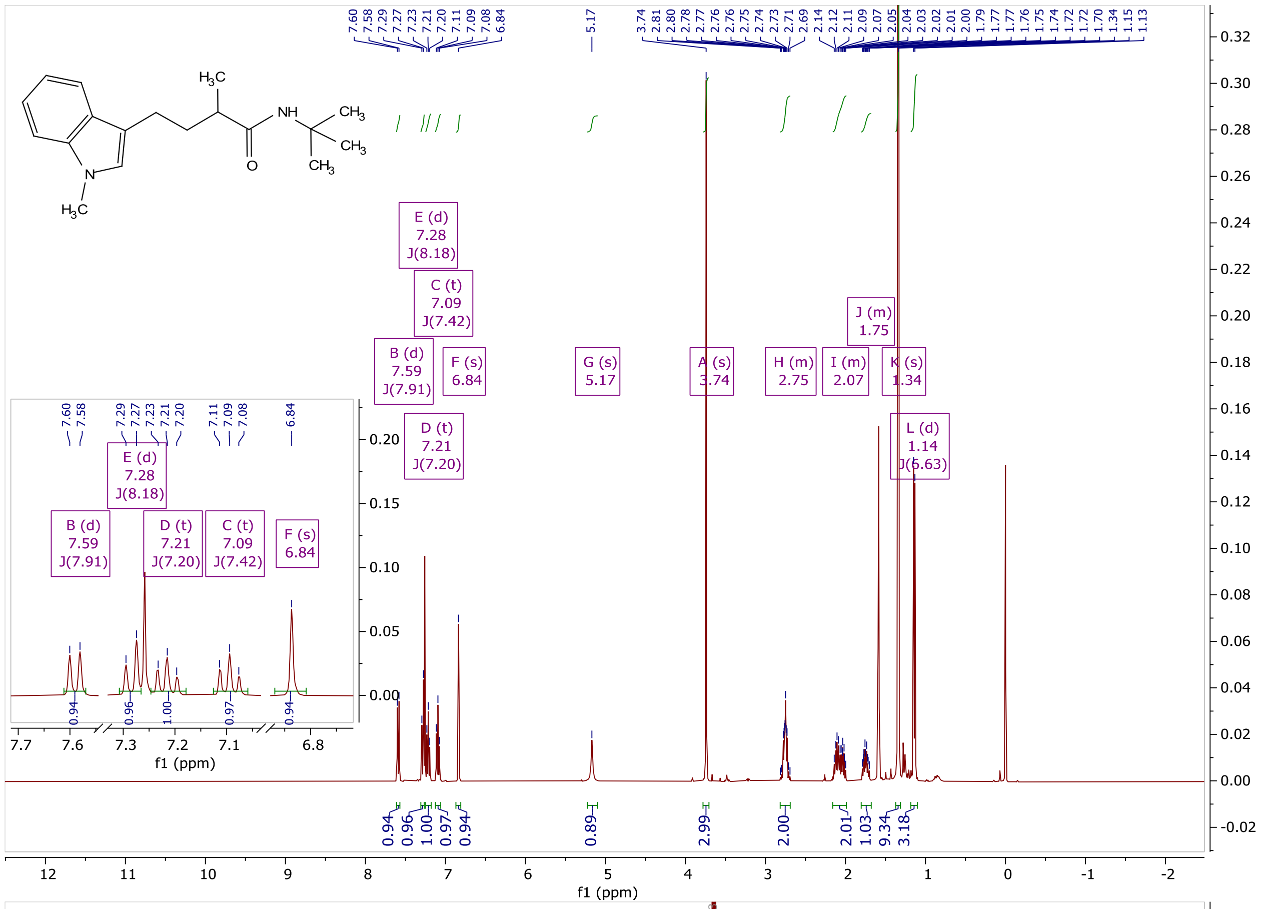

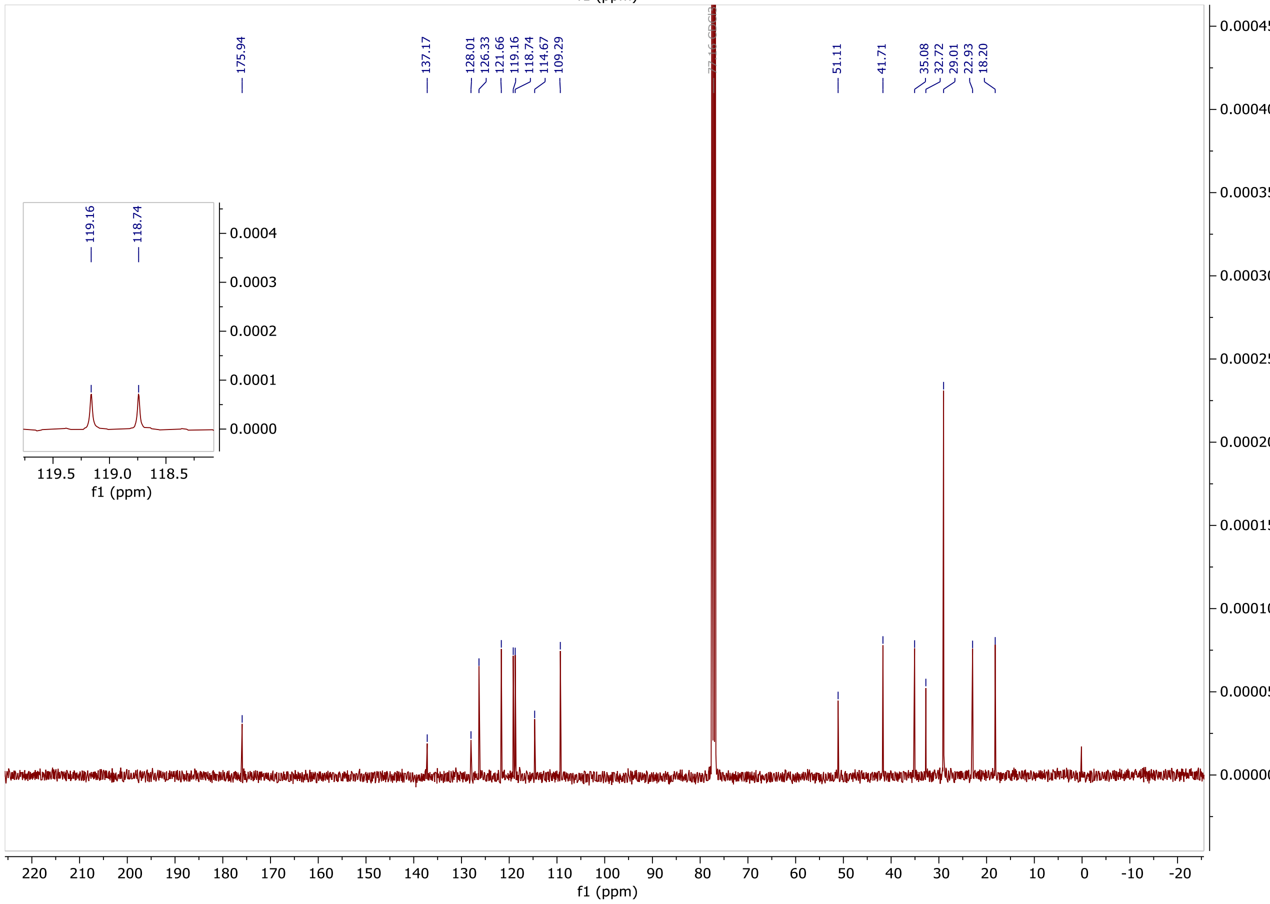

N-Cyclohexyl-2-methyl-4-(1-methyl-1H-indol-3-yl)butanamide (27).

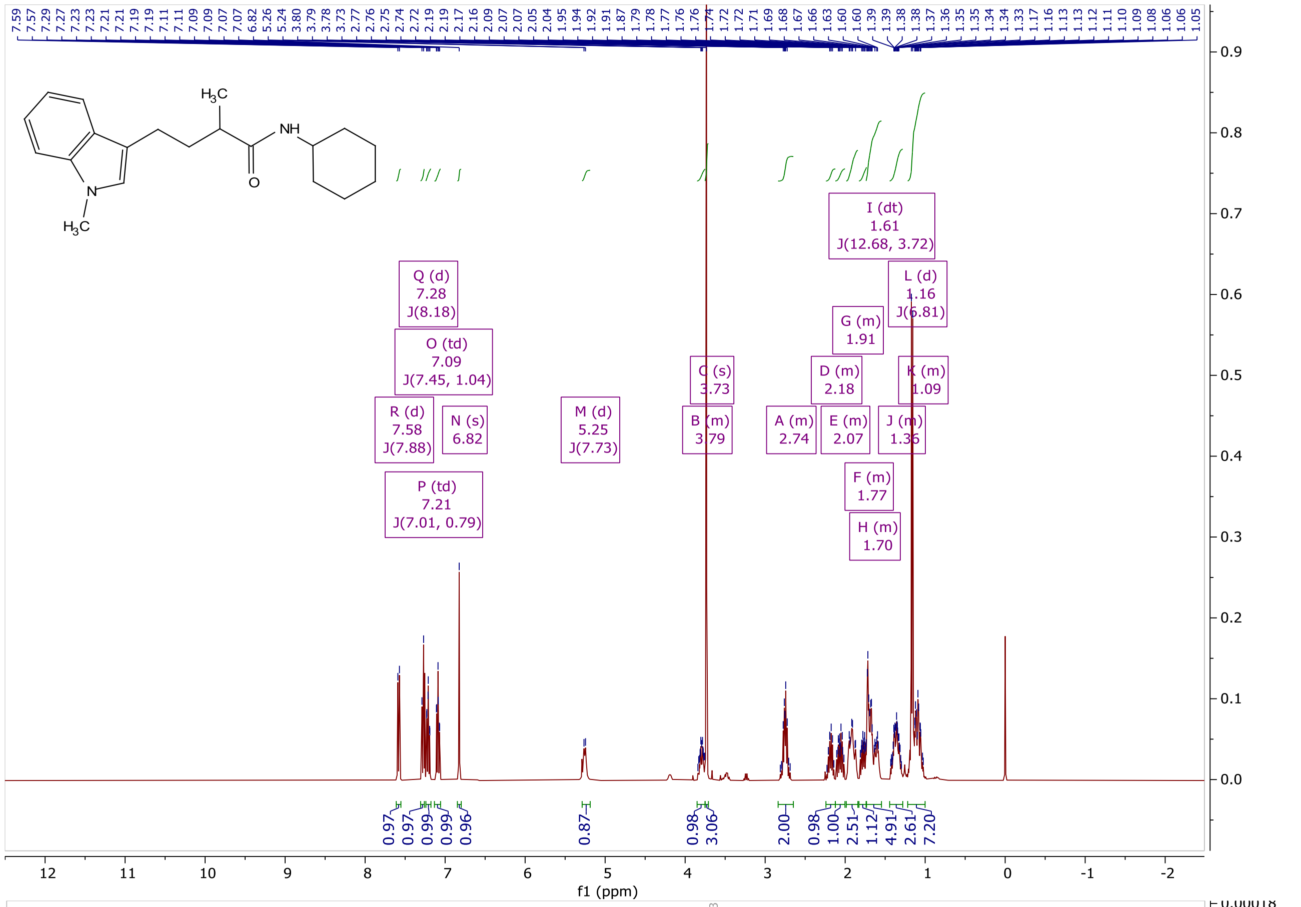

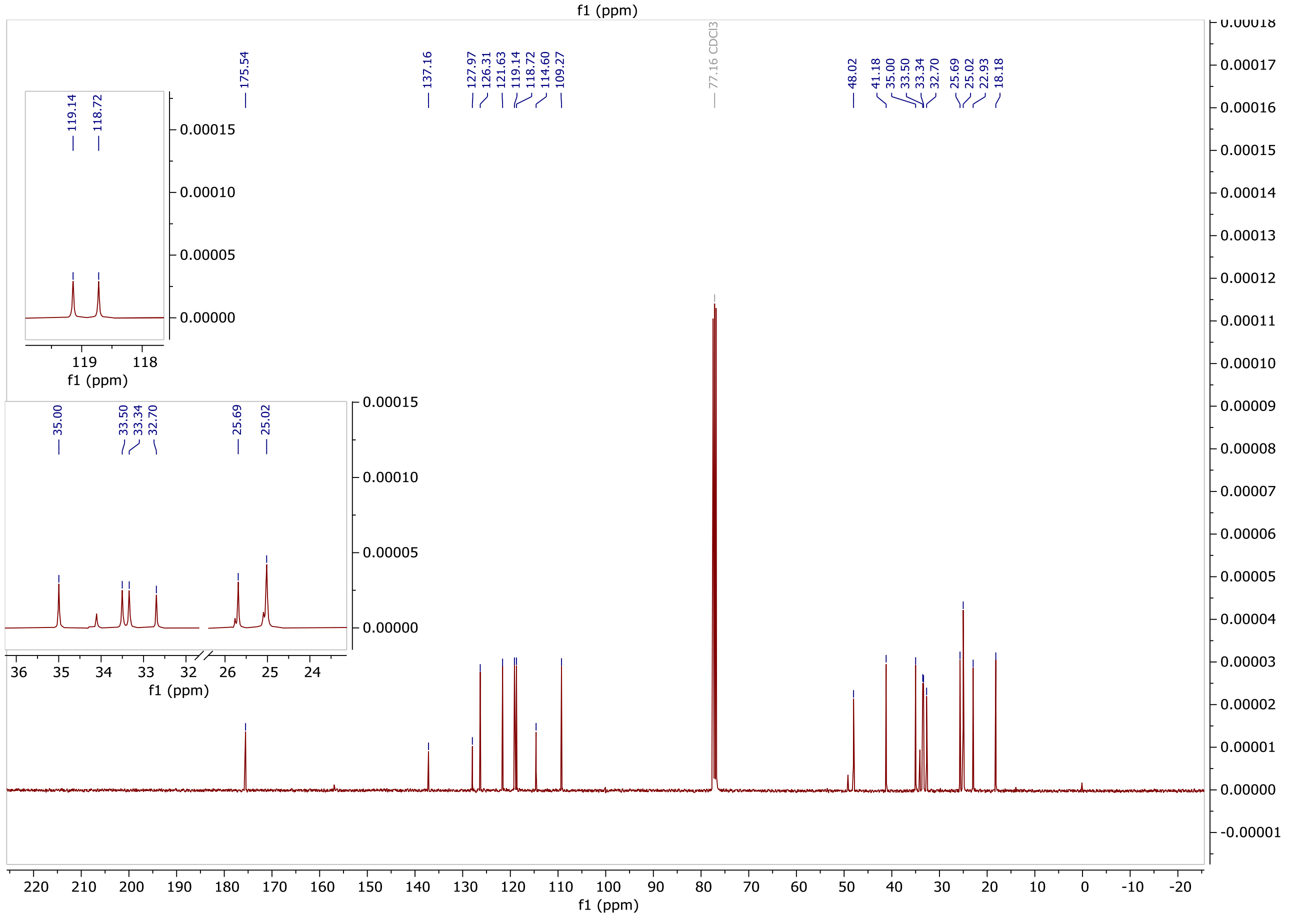

N-(*tert*-Butyl)-4-((*tert*-butyldimethylsilyl)oxy)-2-methylbutanamide (28).

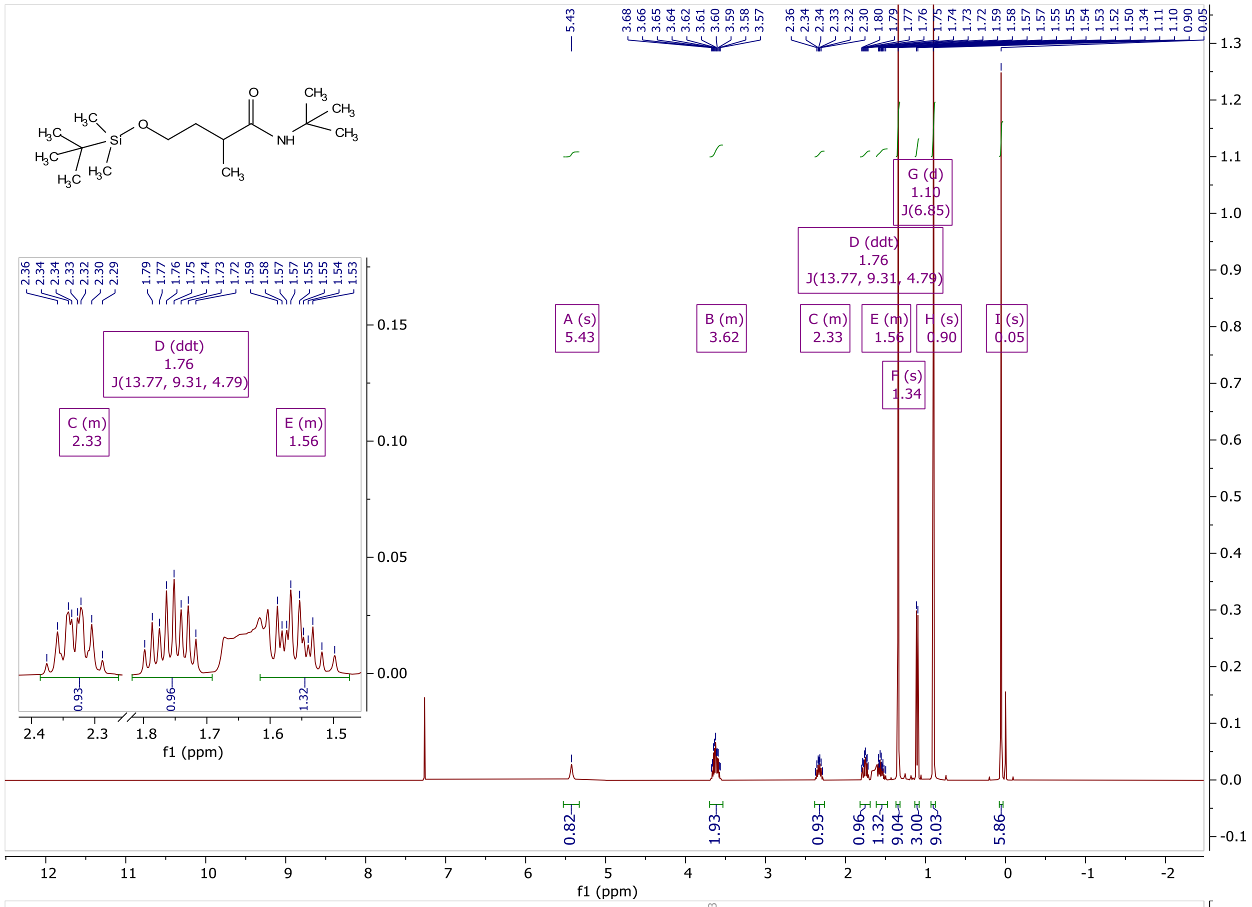

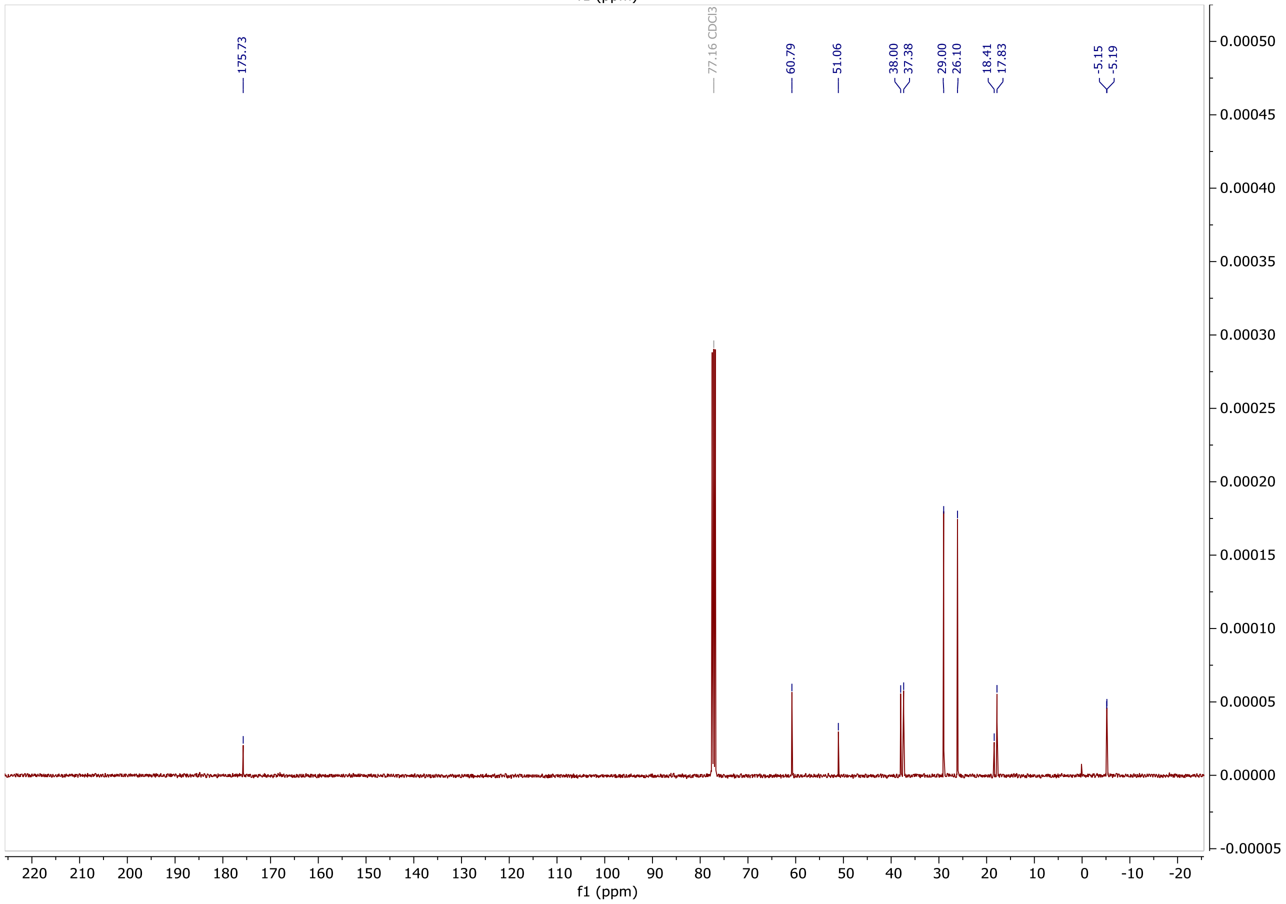

4-(*tert*-Butylamino)-3-methyl-4-oxobutyl benzoate (30).

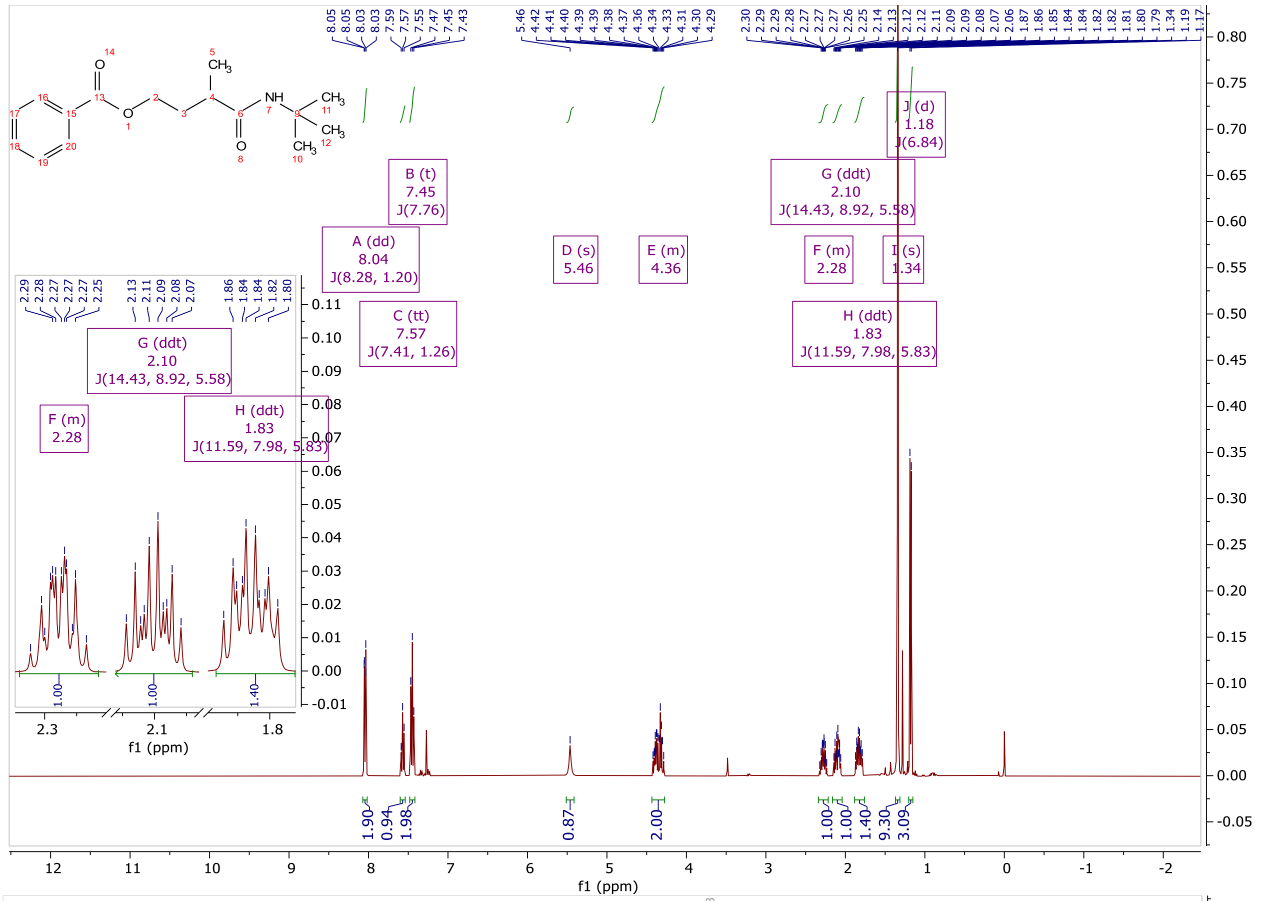

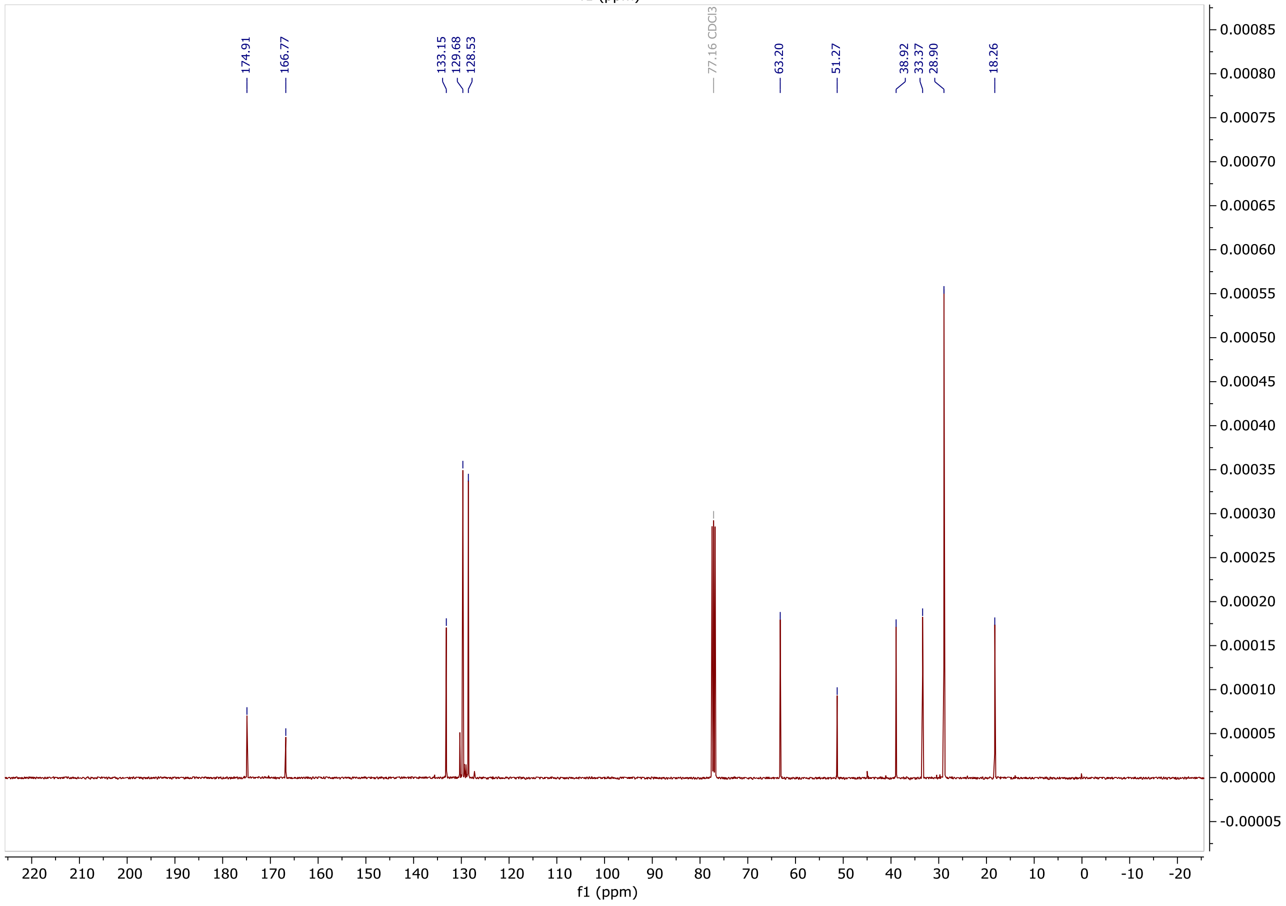

(3-Iodobutyl)benzene (1).

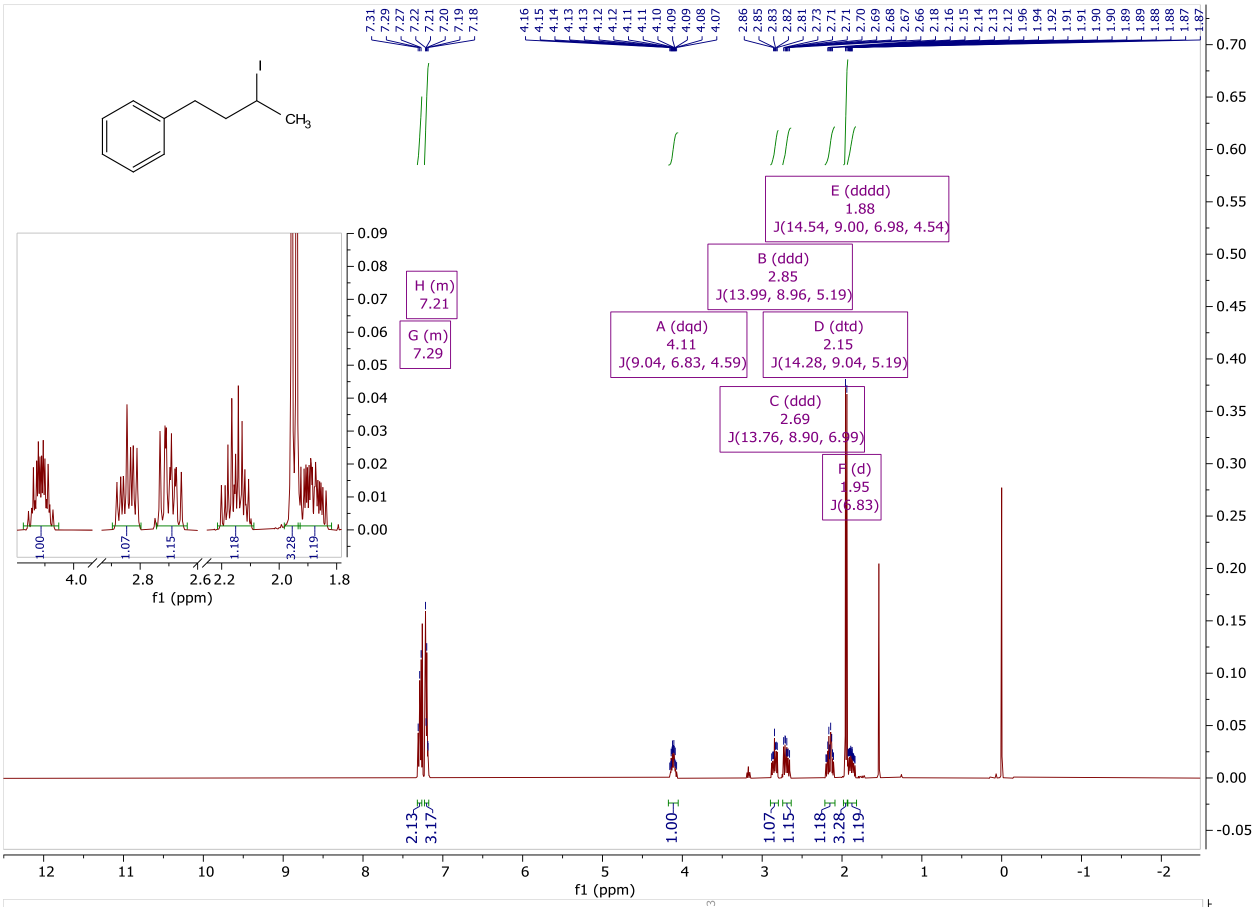

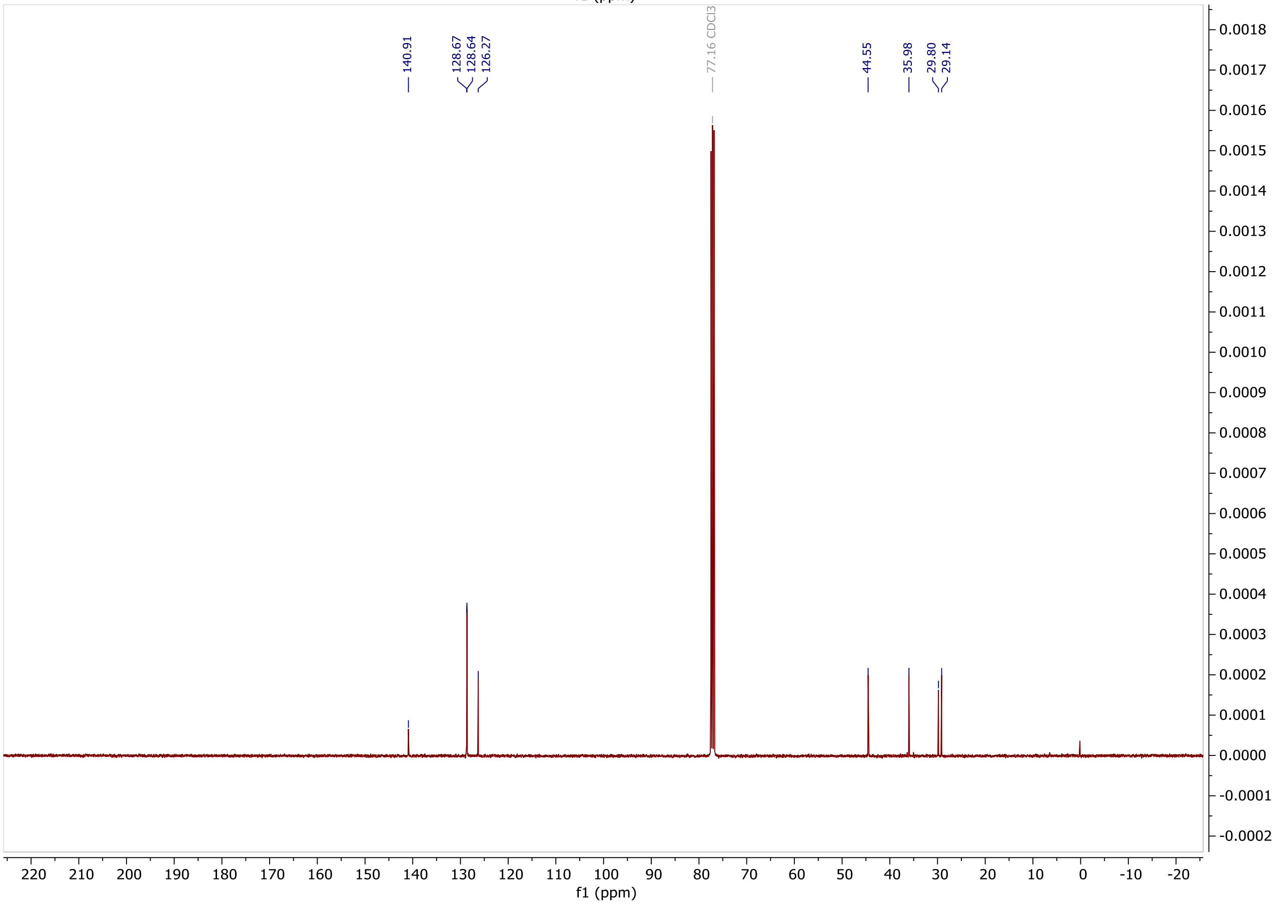

(3-Iodopropyl)benzene (S3).

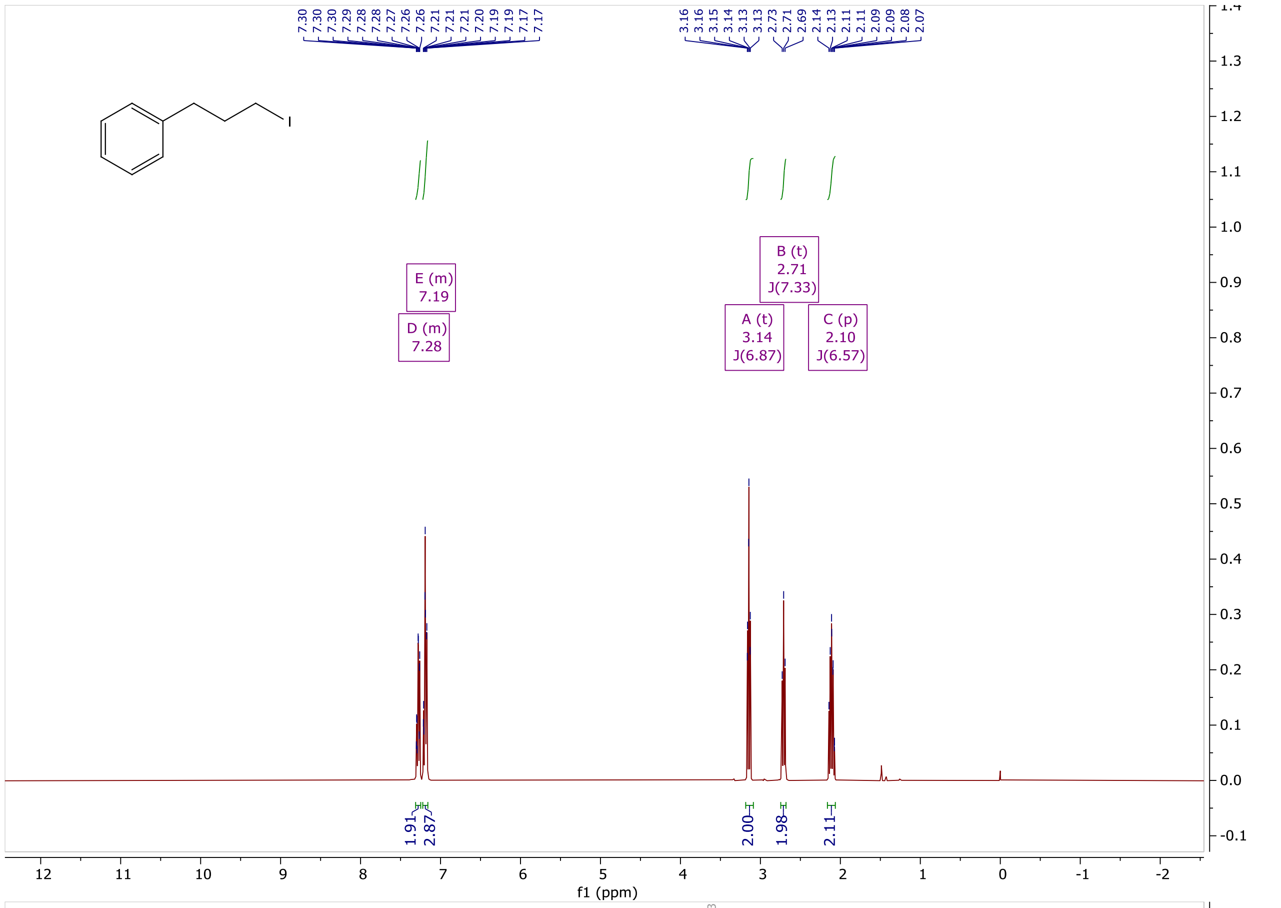

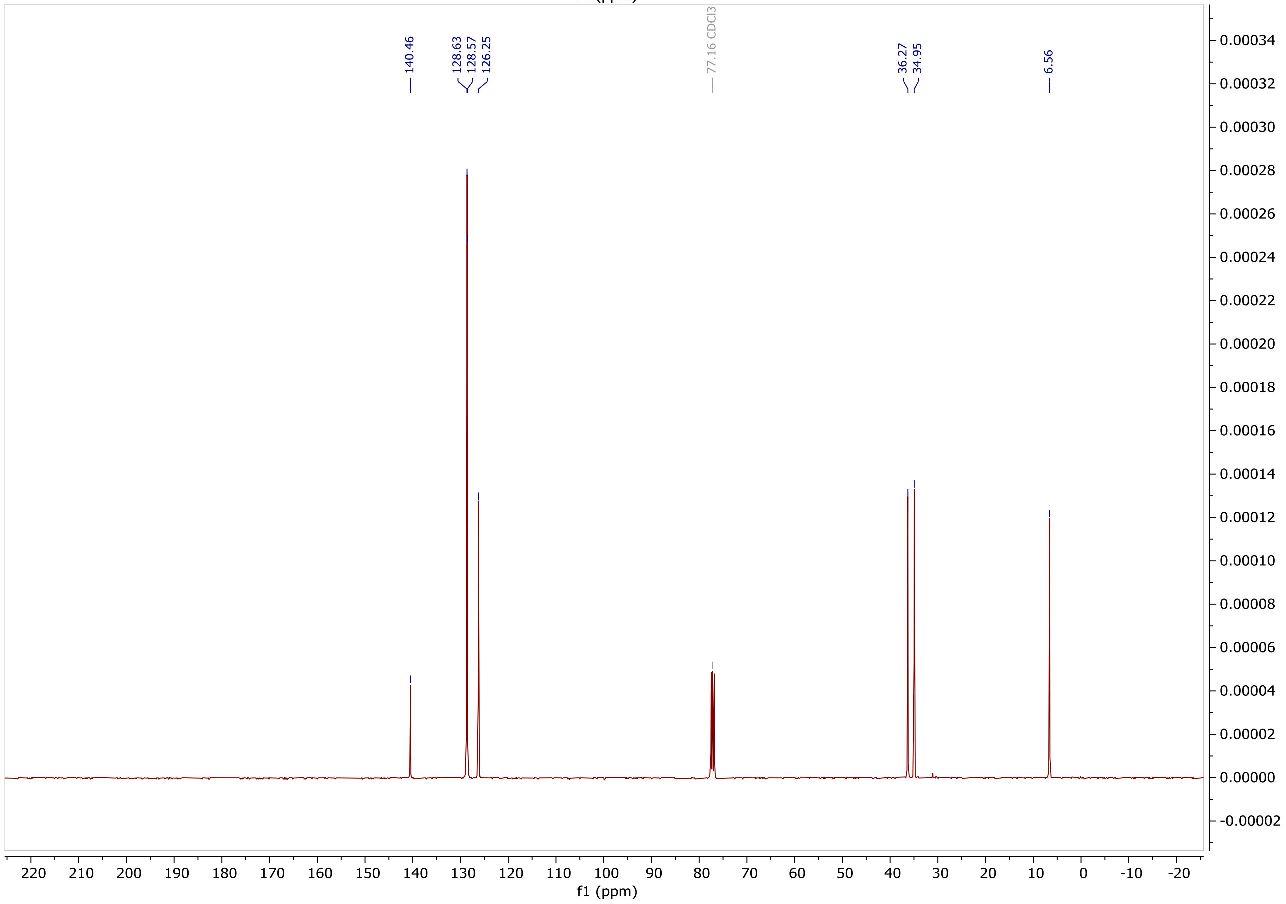

(3s,5s,7s)-1-Iodoadamantane (S4).

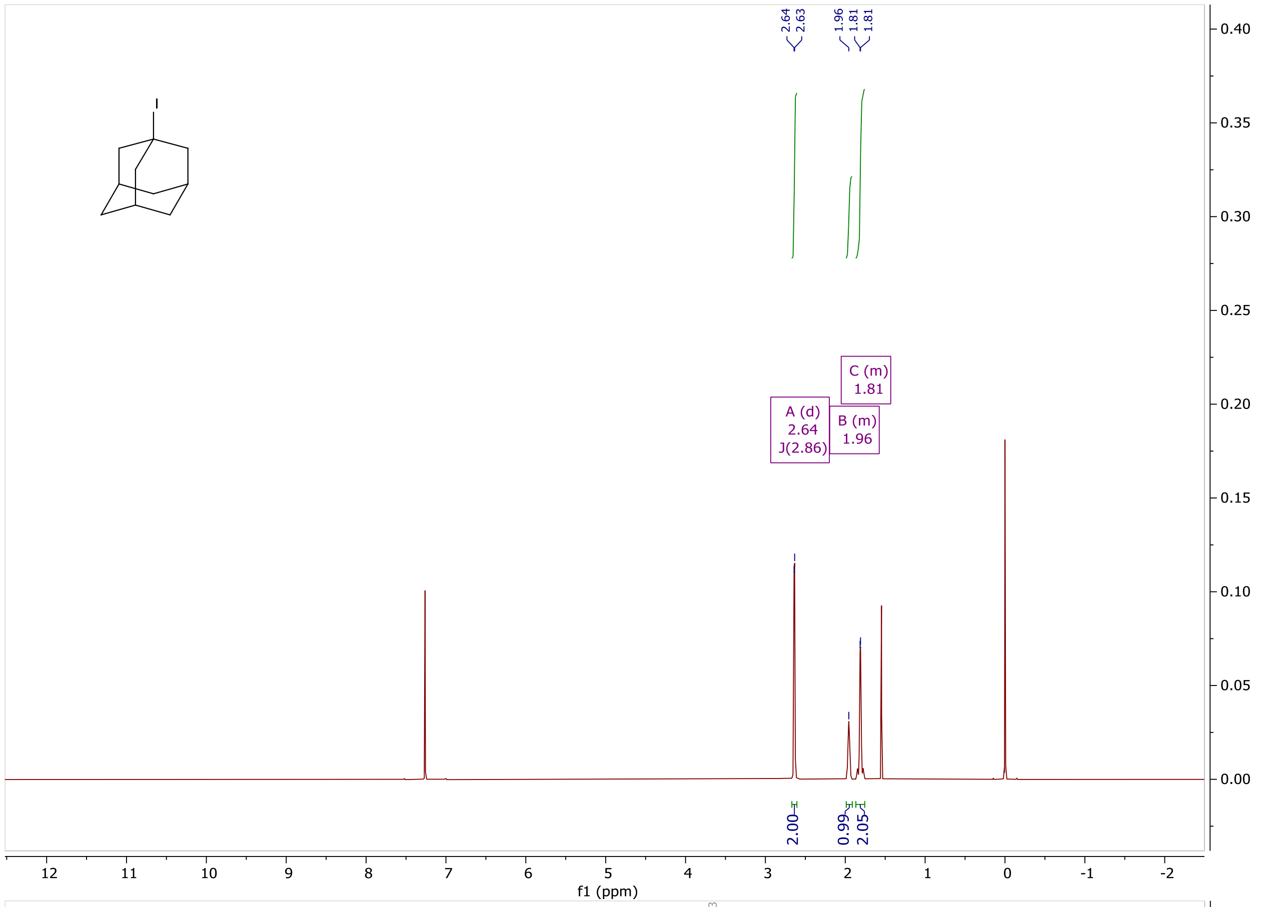

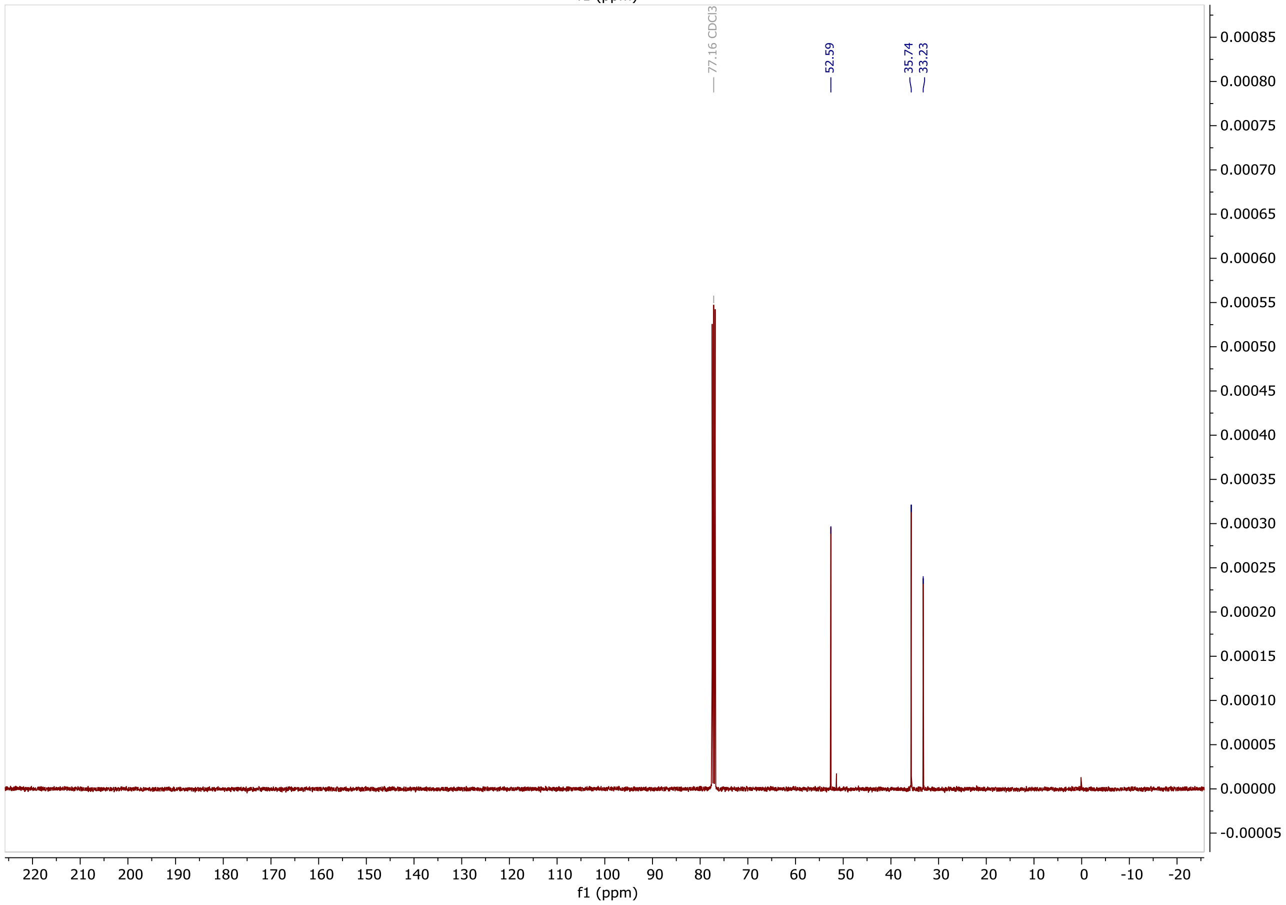

(3-Iodo-2,2-dimethylpropyl)benzene (S8).

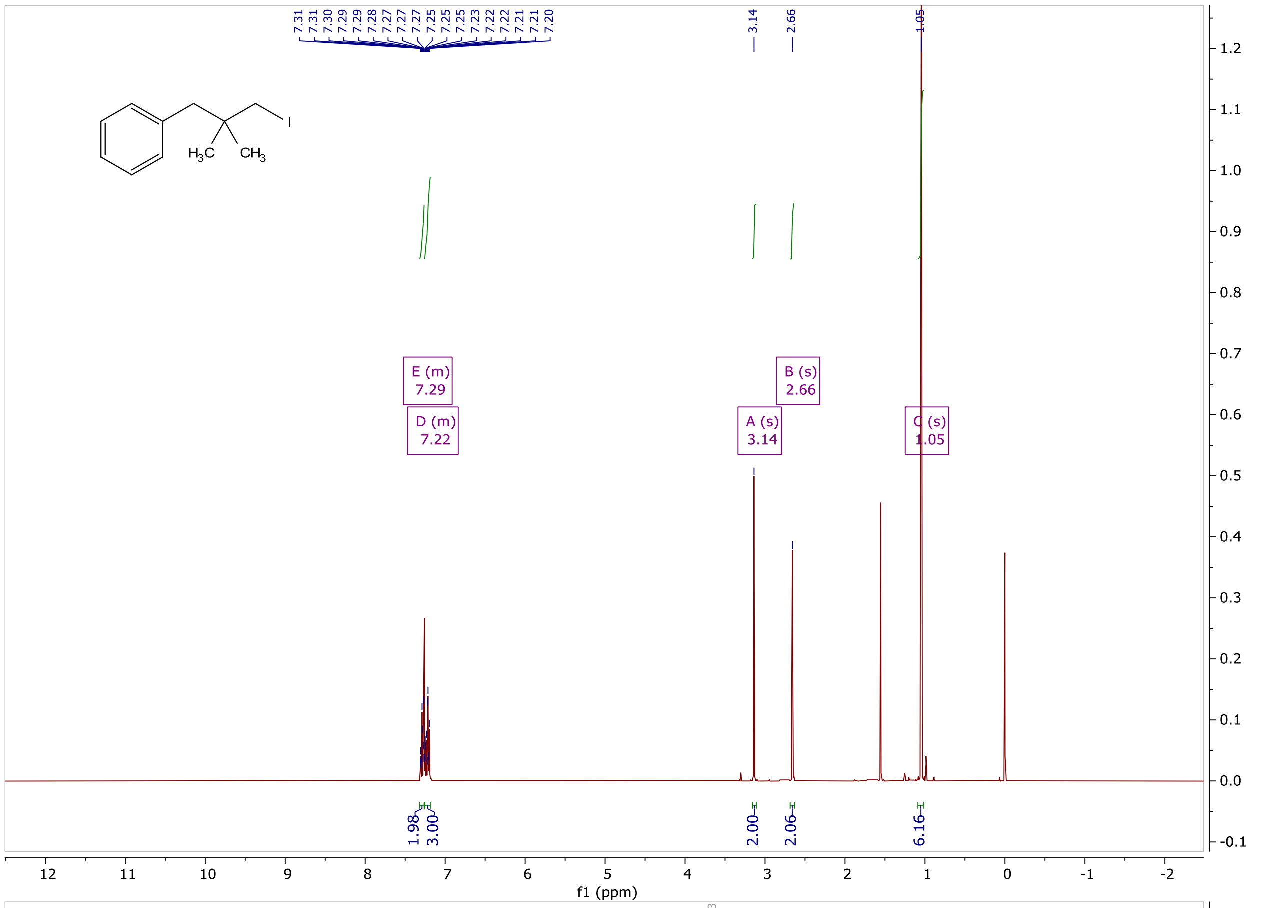

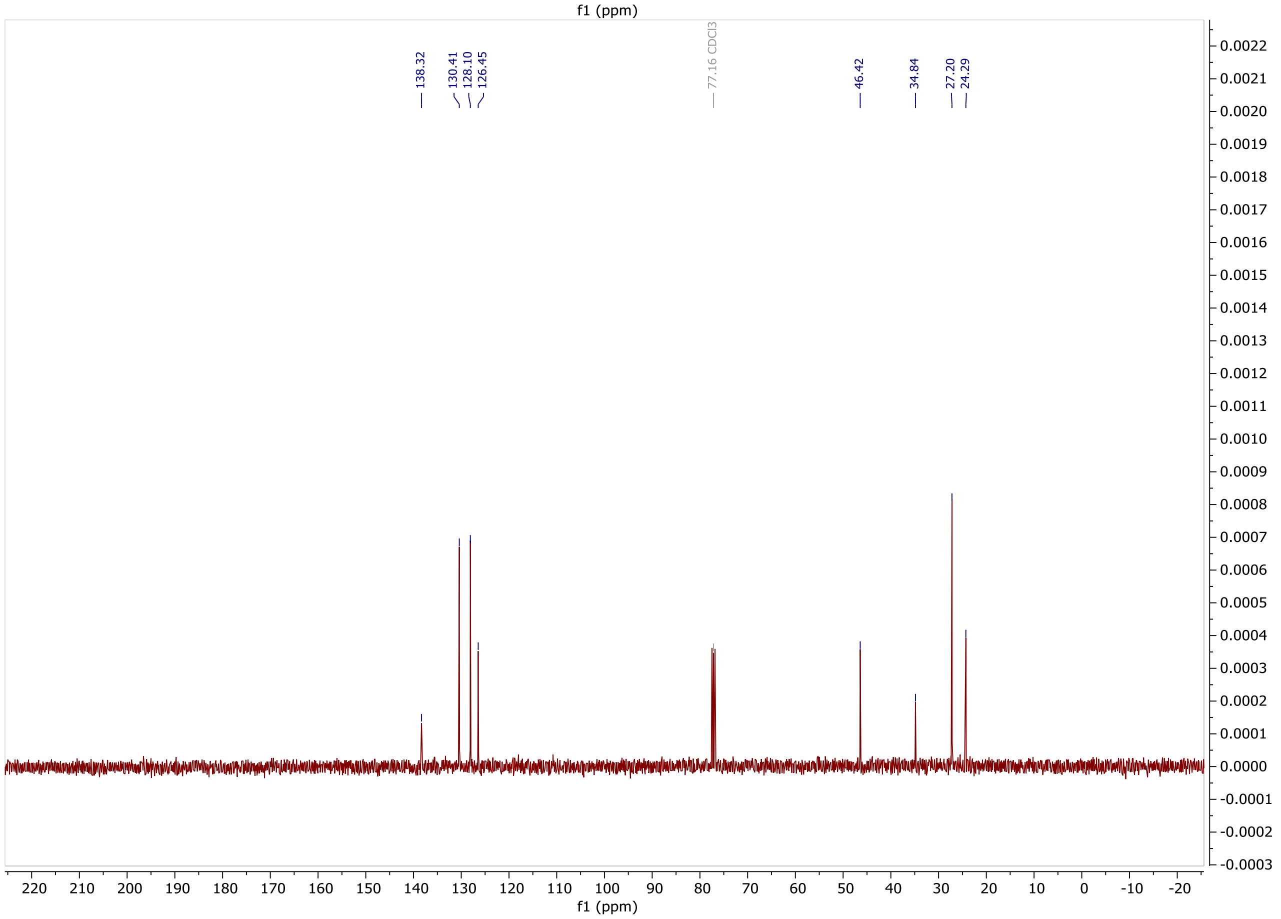

2-(3-Iodobutyl)thiophene (20).

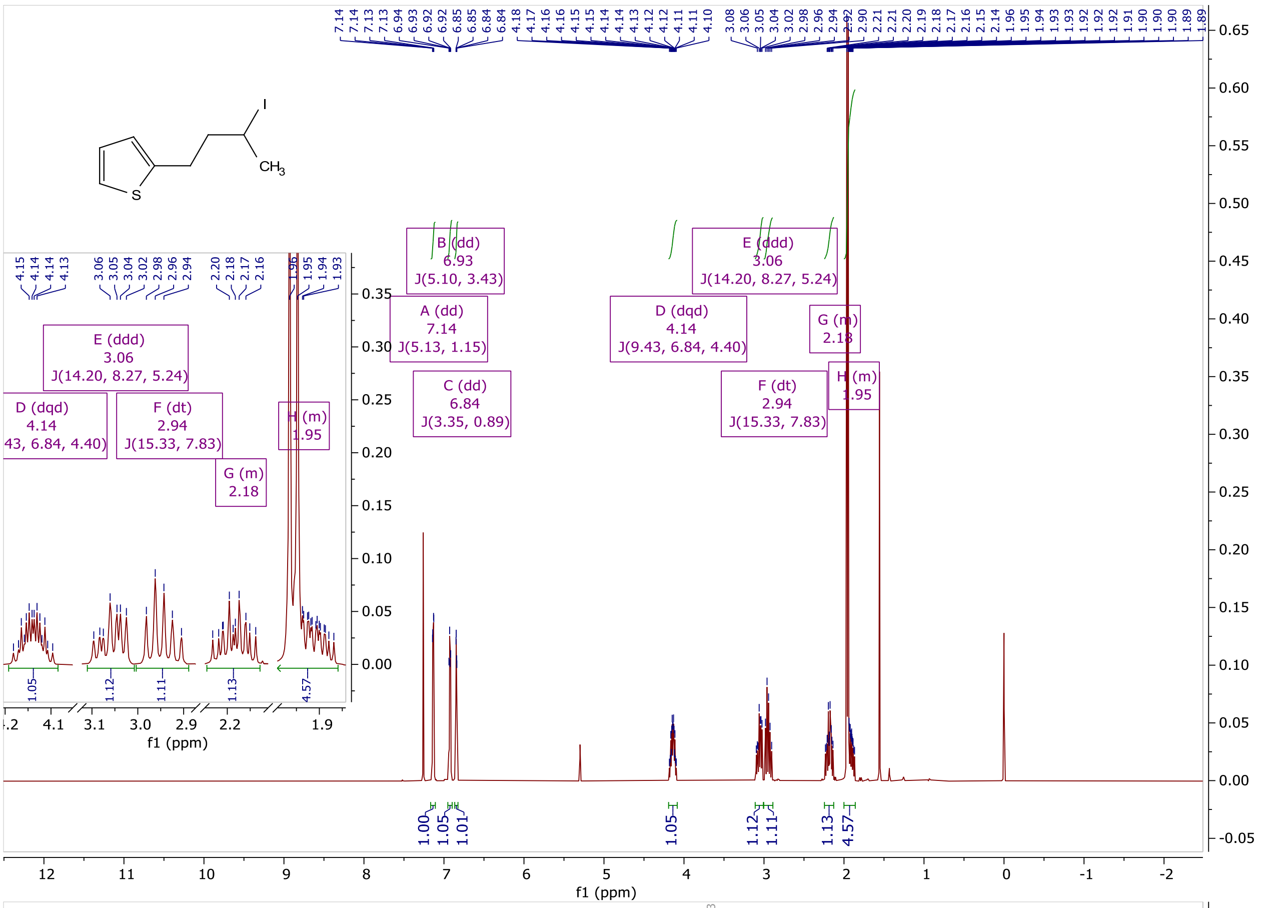

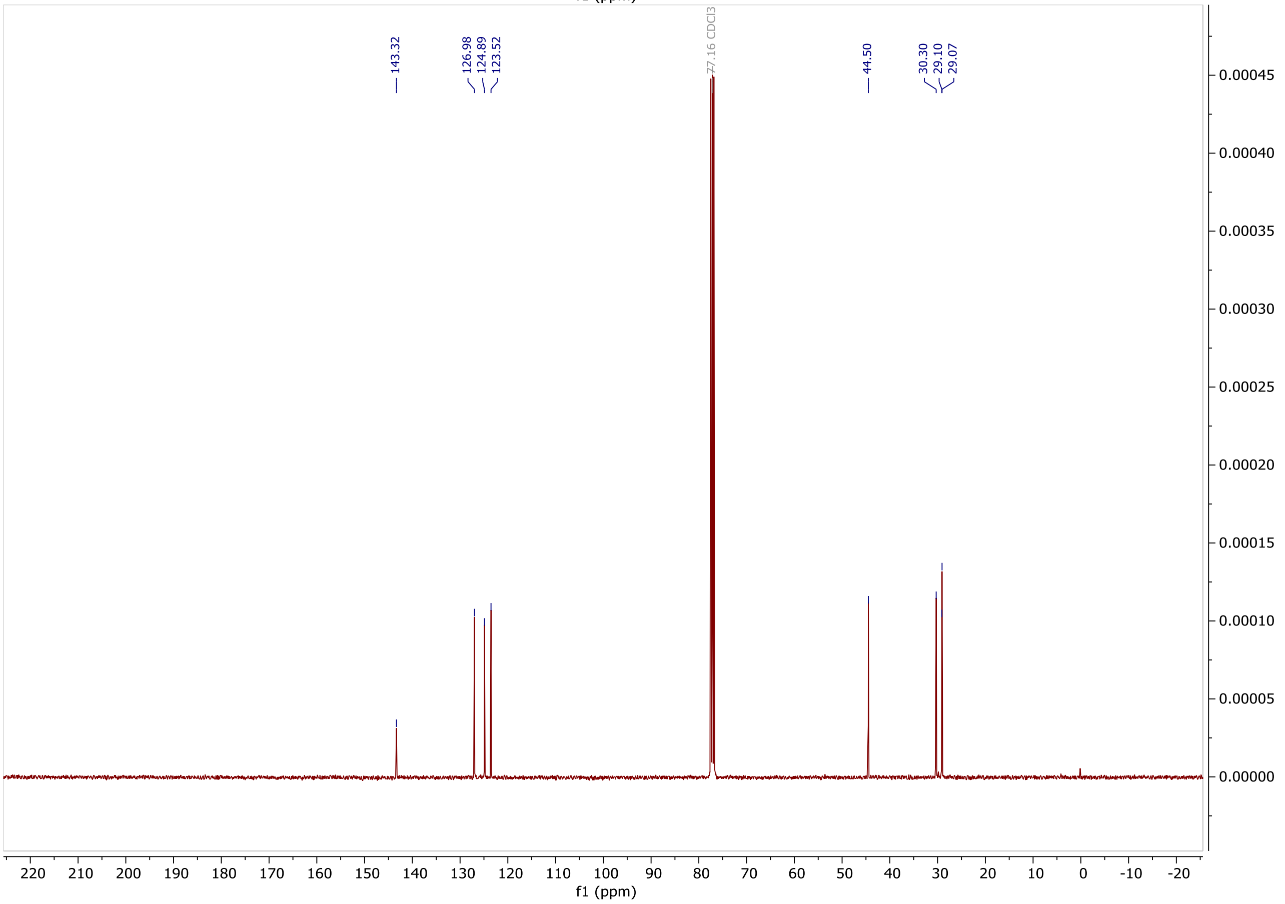

1-(3-Iodobutoxy)-4-methoxybenzene (S12).

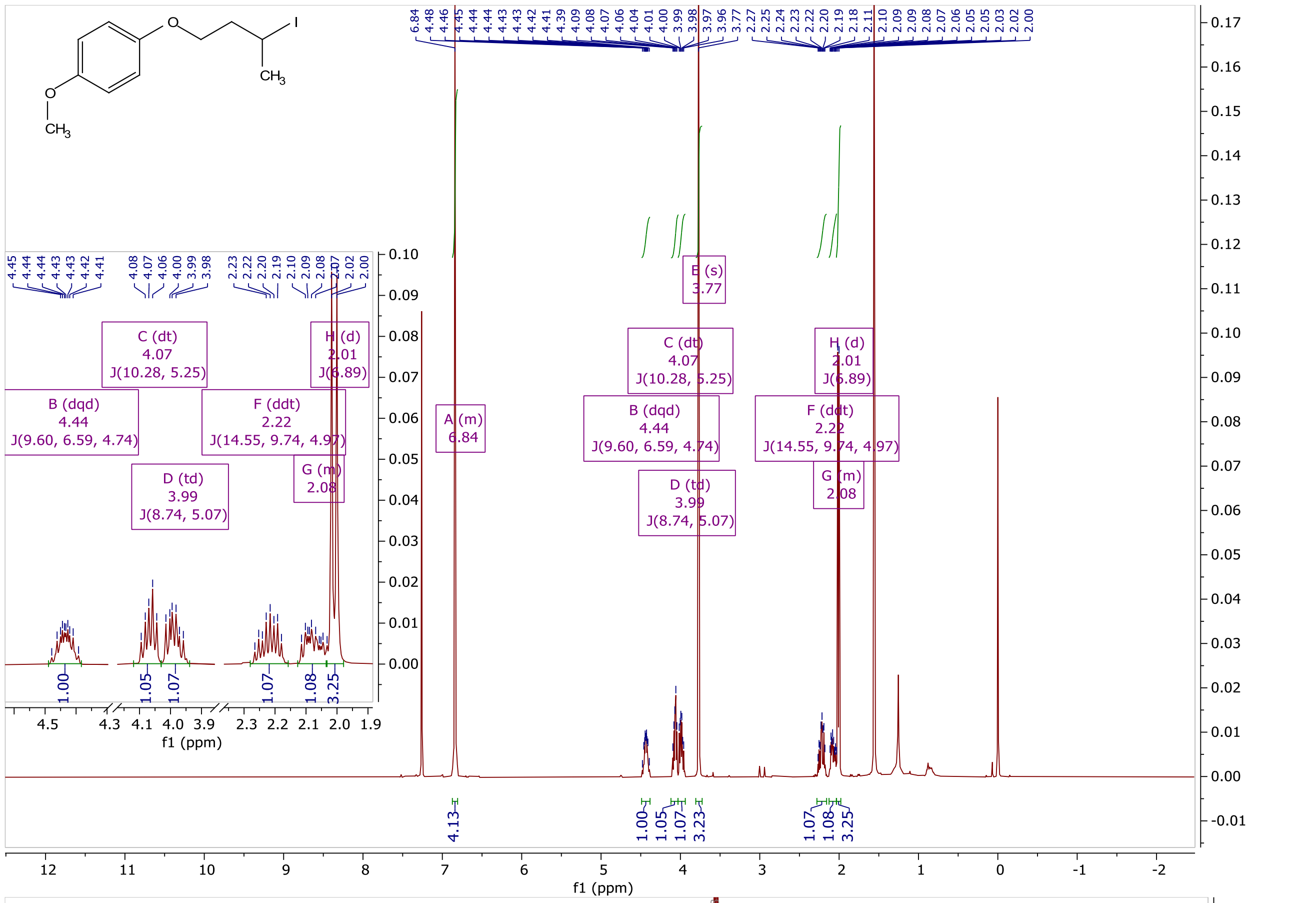

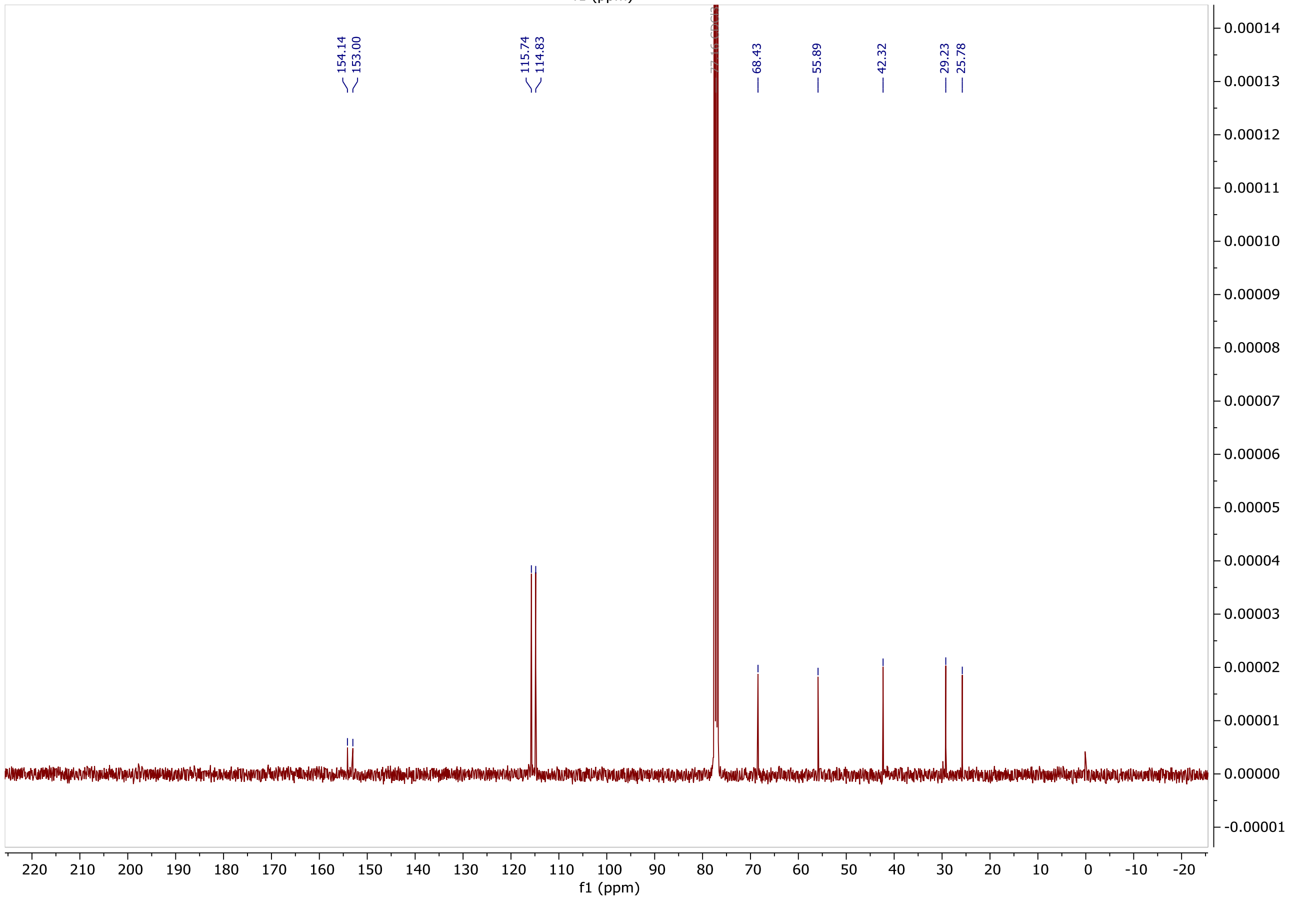

***N*-(4-(3-iodobutyl)phenyl)acetamide (S17).**

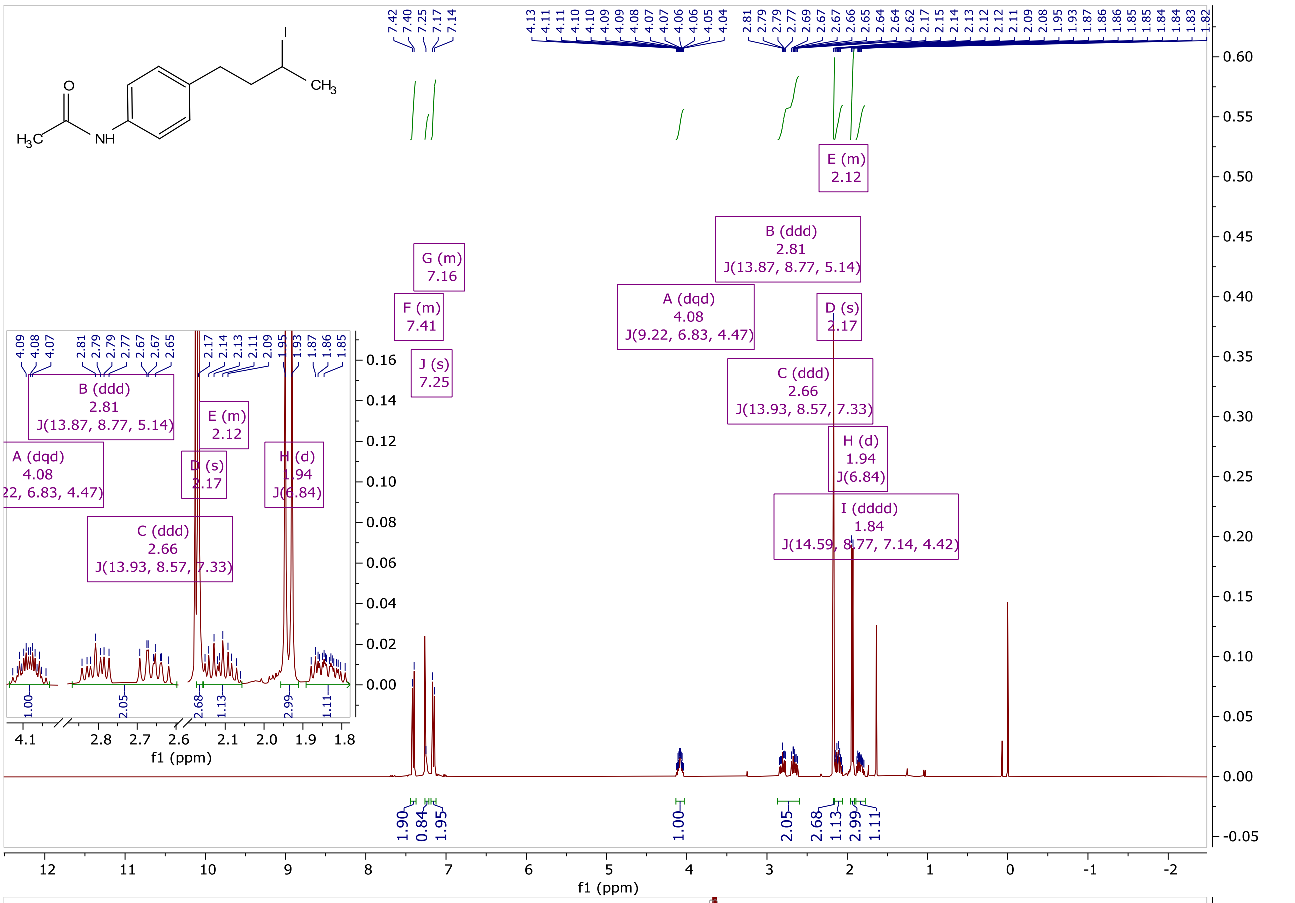

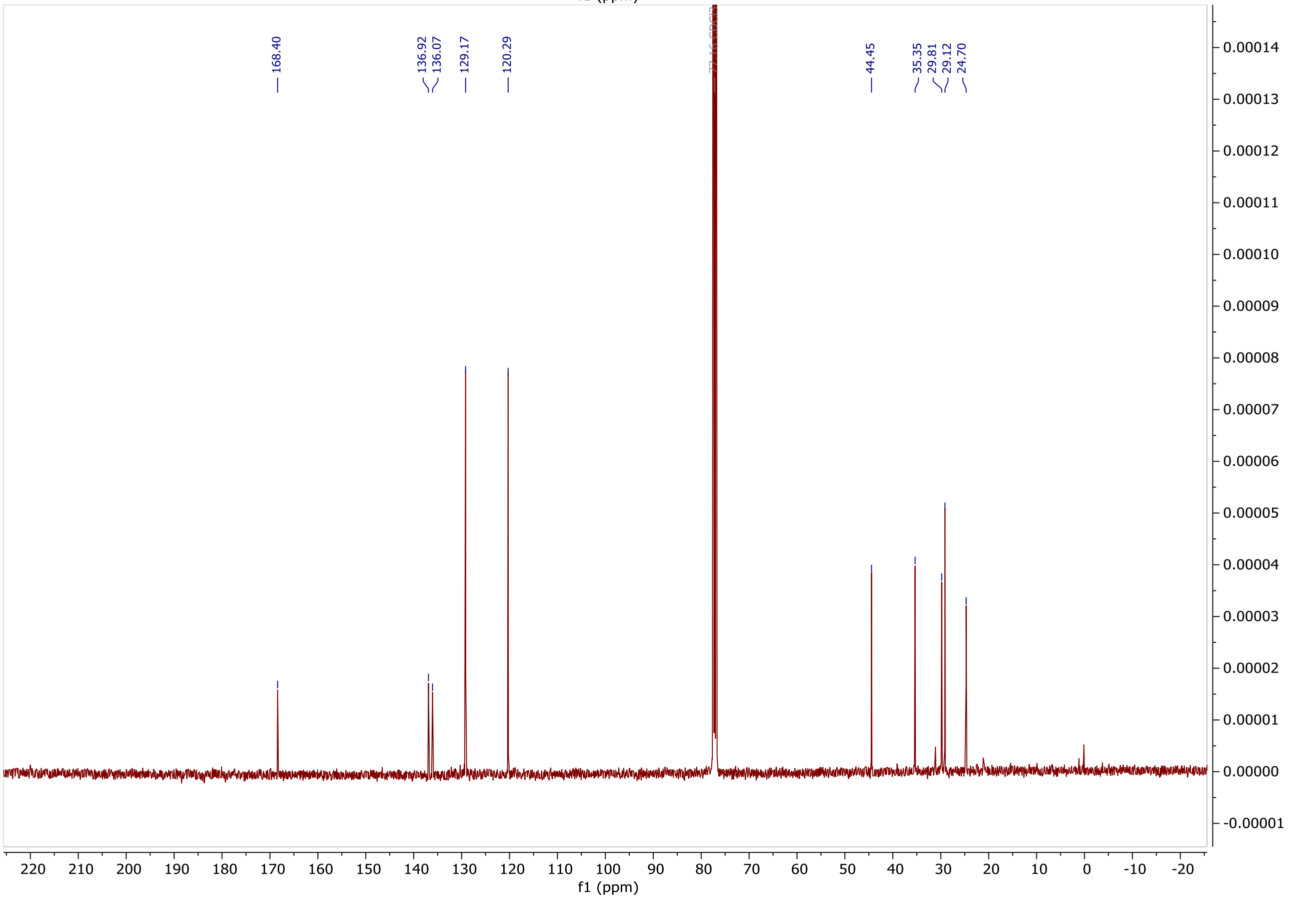

***tert*-Butyl (4-(3-iodobutyl)phenyl)carbamate (S20).**

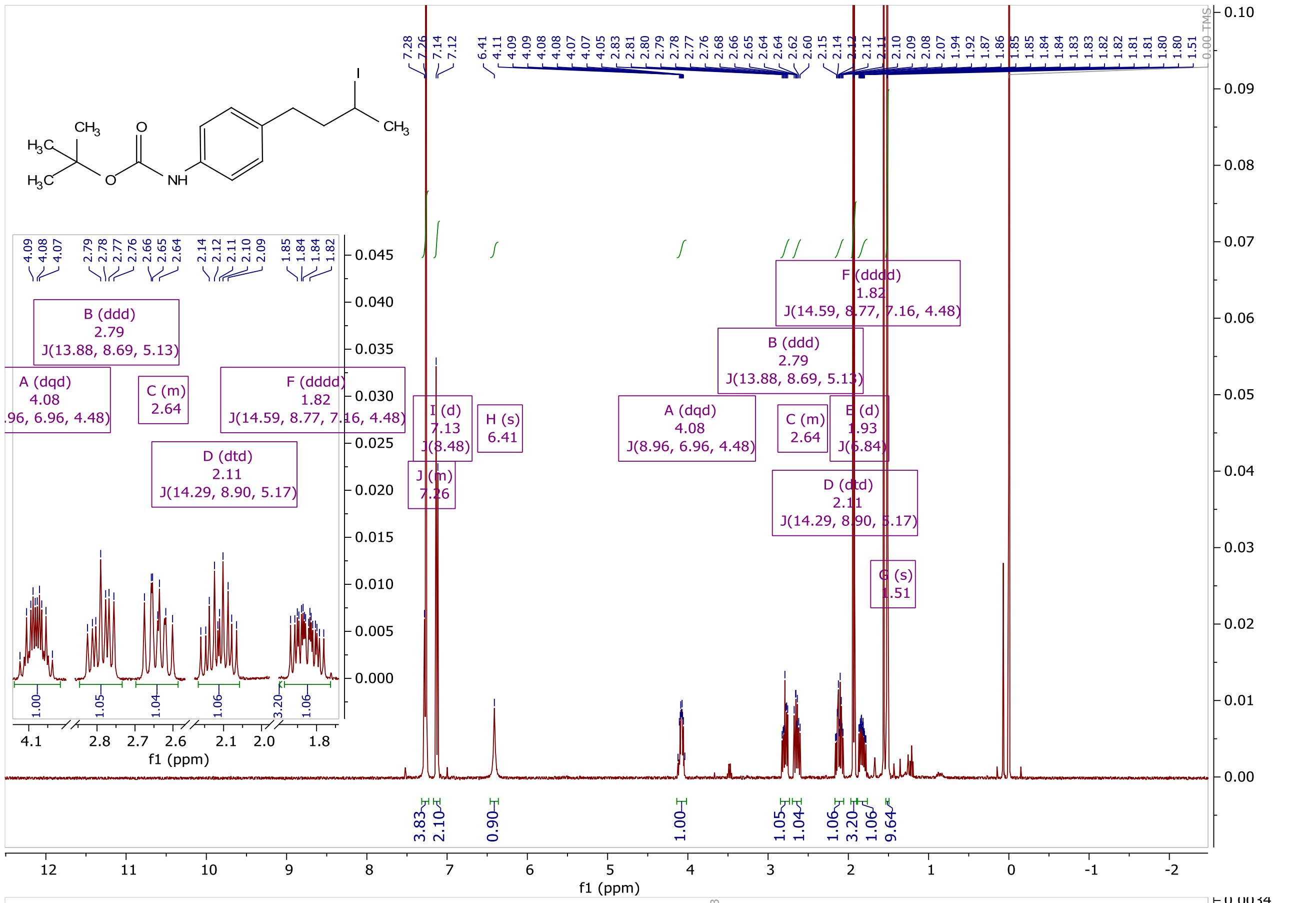

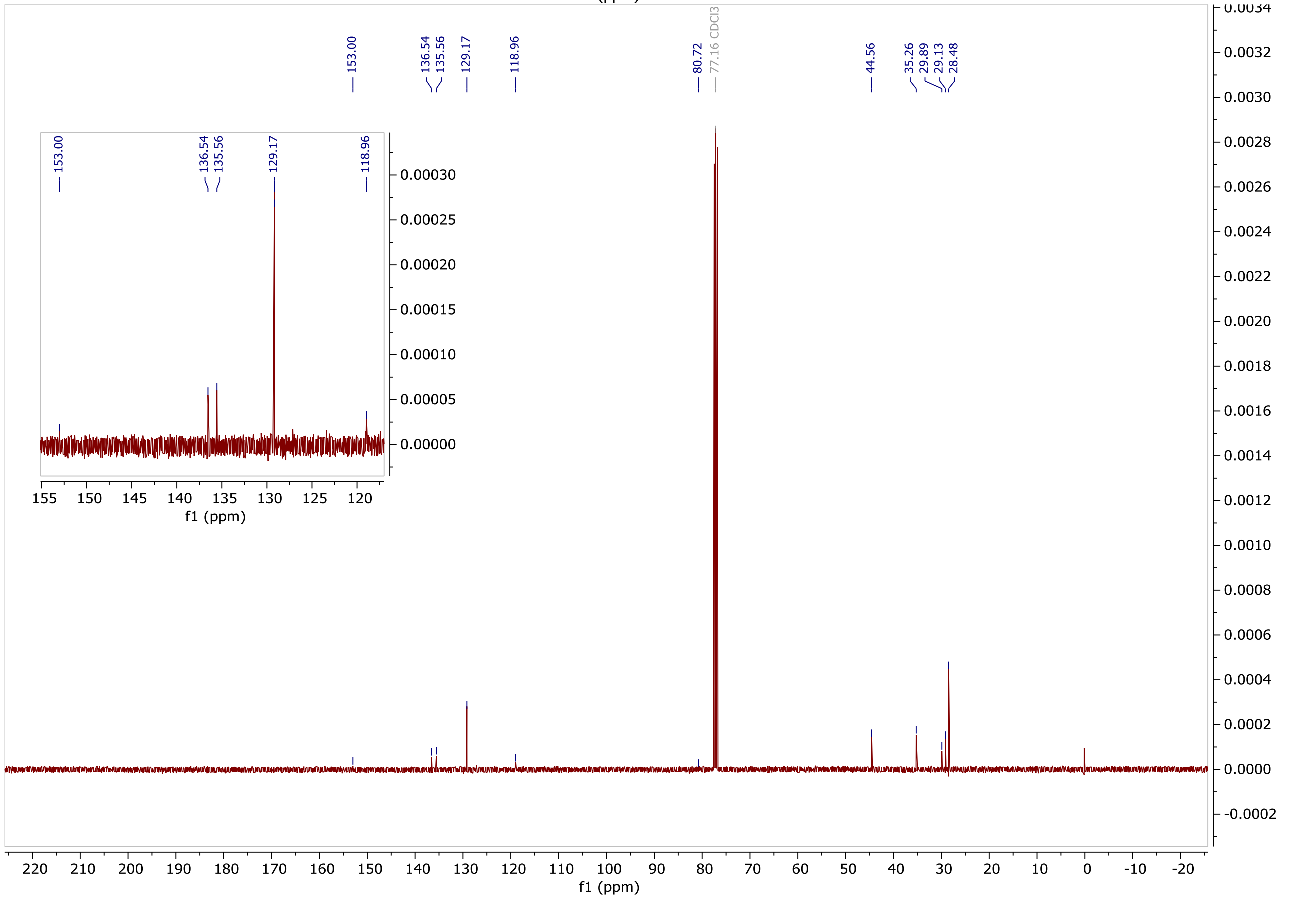

3-(3-Iodobutyl)-1-methyl-1*H*-indole (S22).

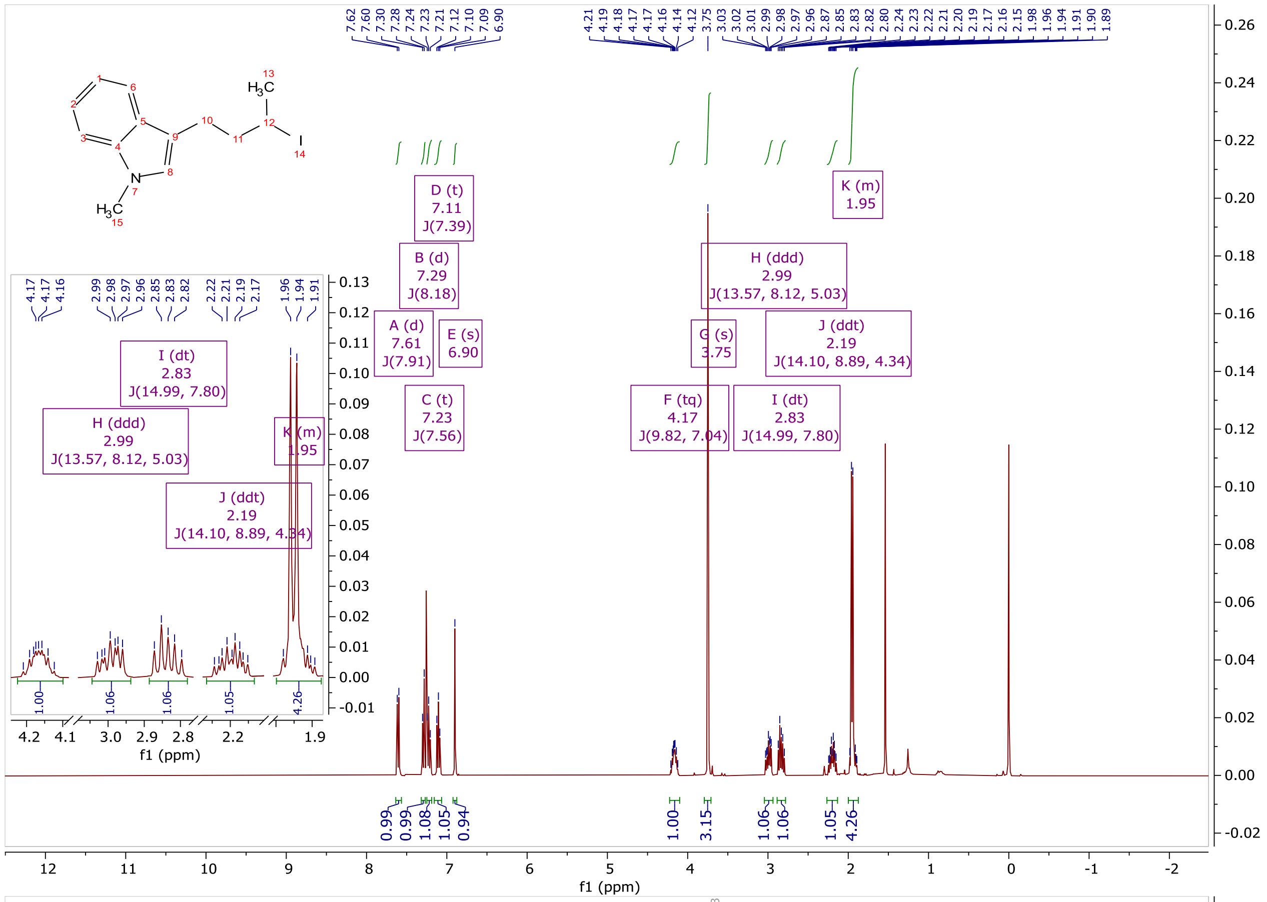

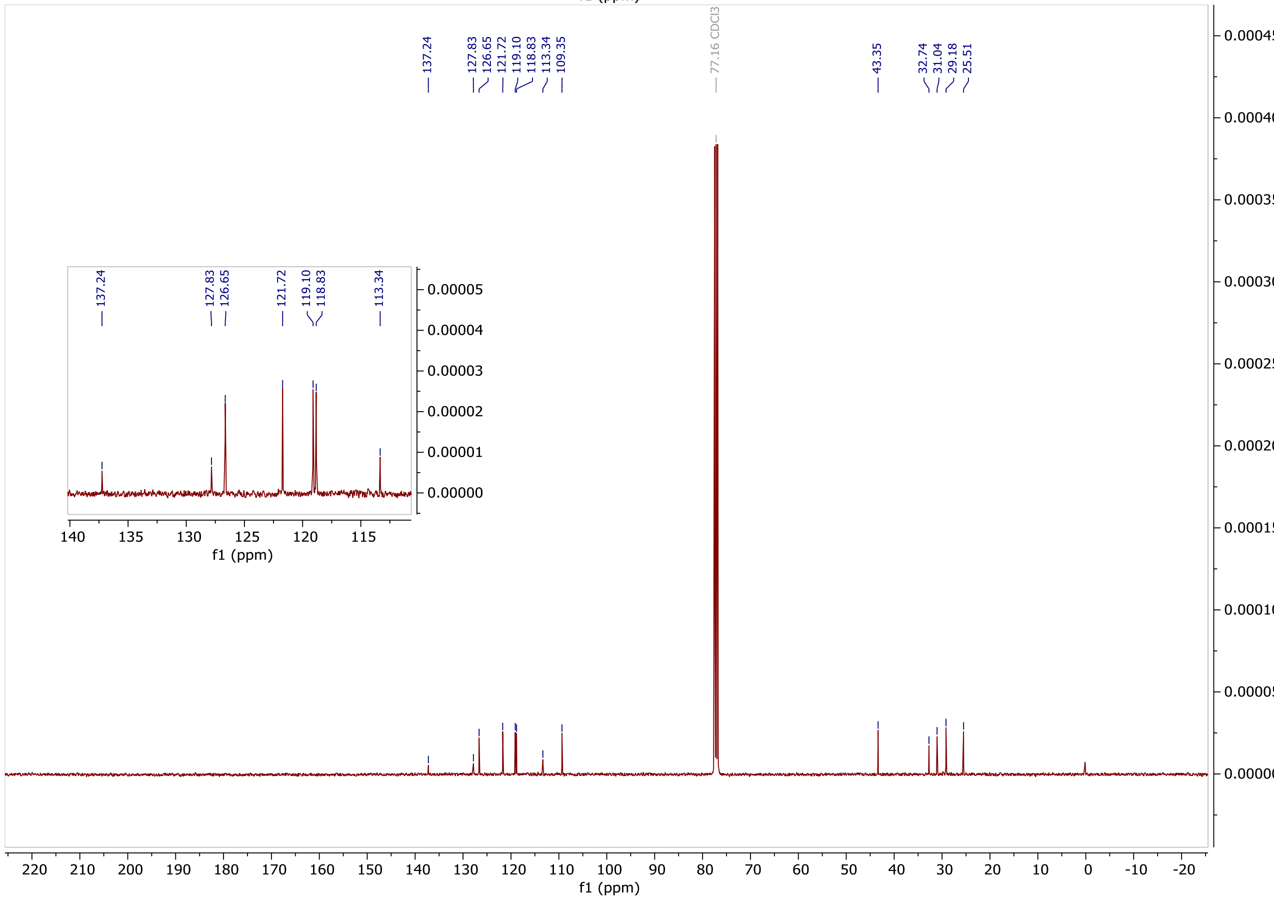

***tert*-Butyl(3-iodobutoxy)dimethylsilane (S26).**

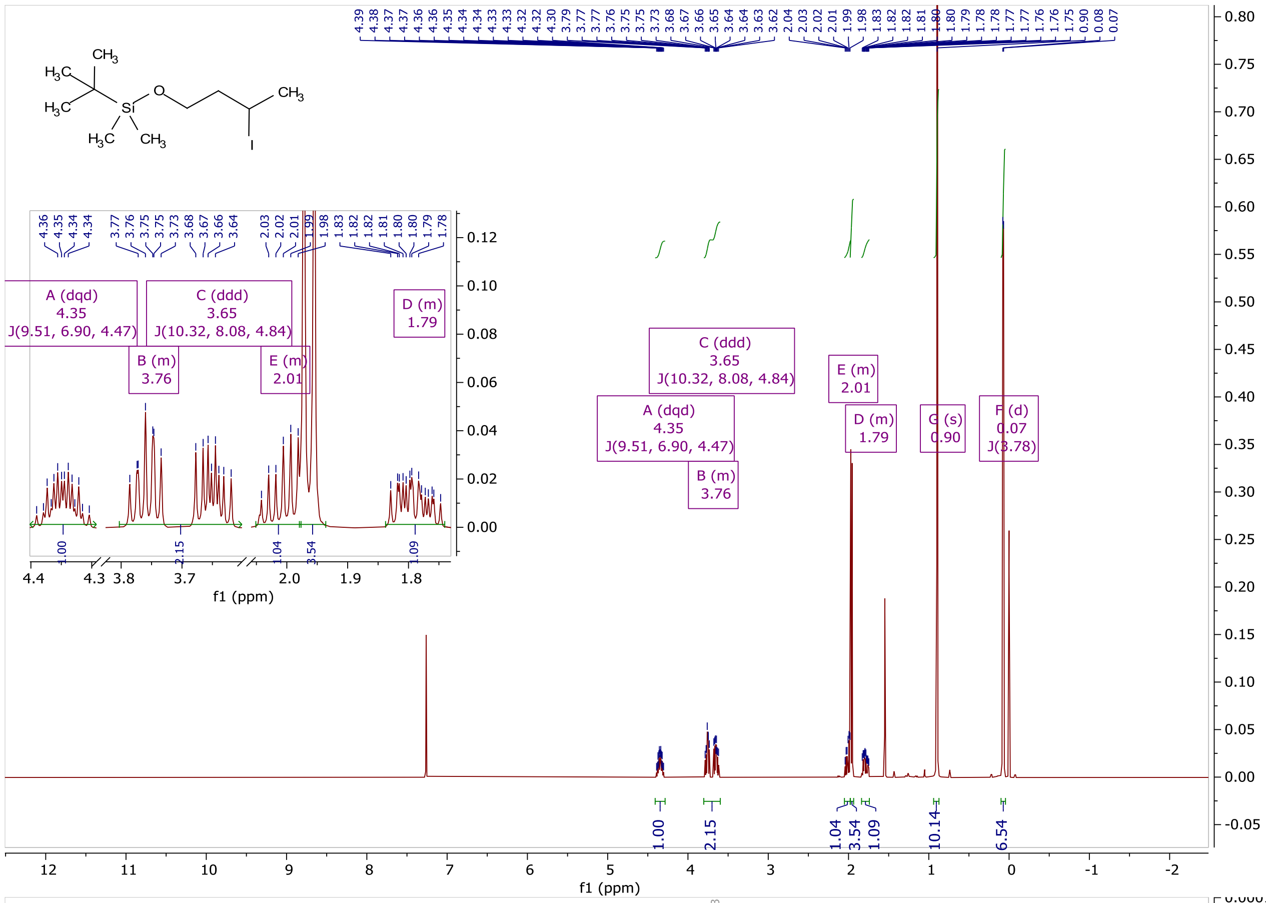

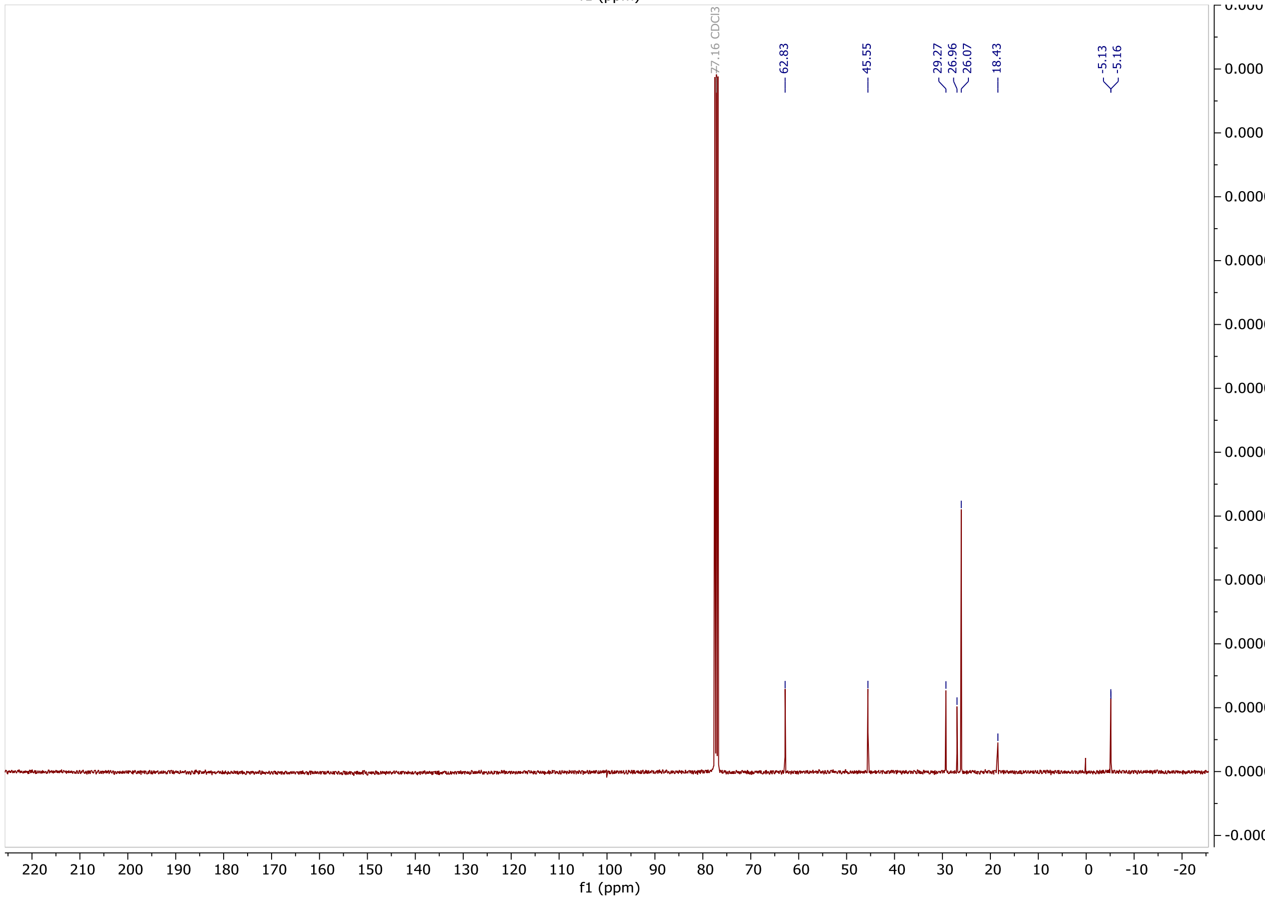

3-Iodobutan-1-ol (S27).

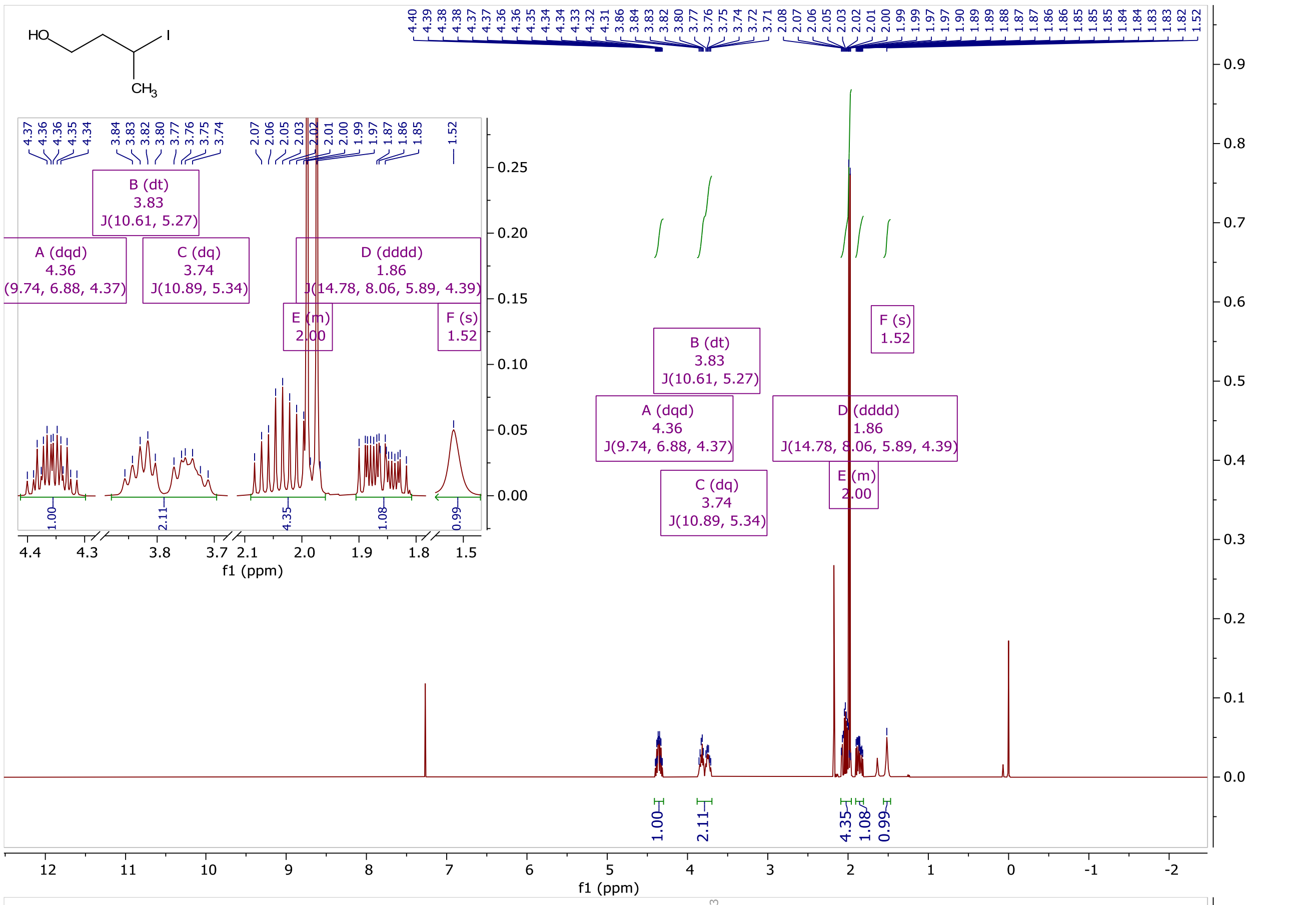

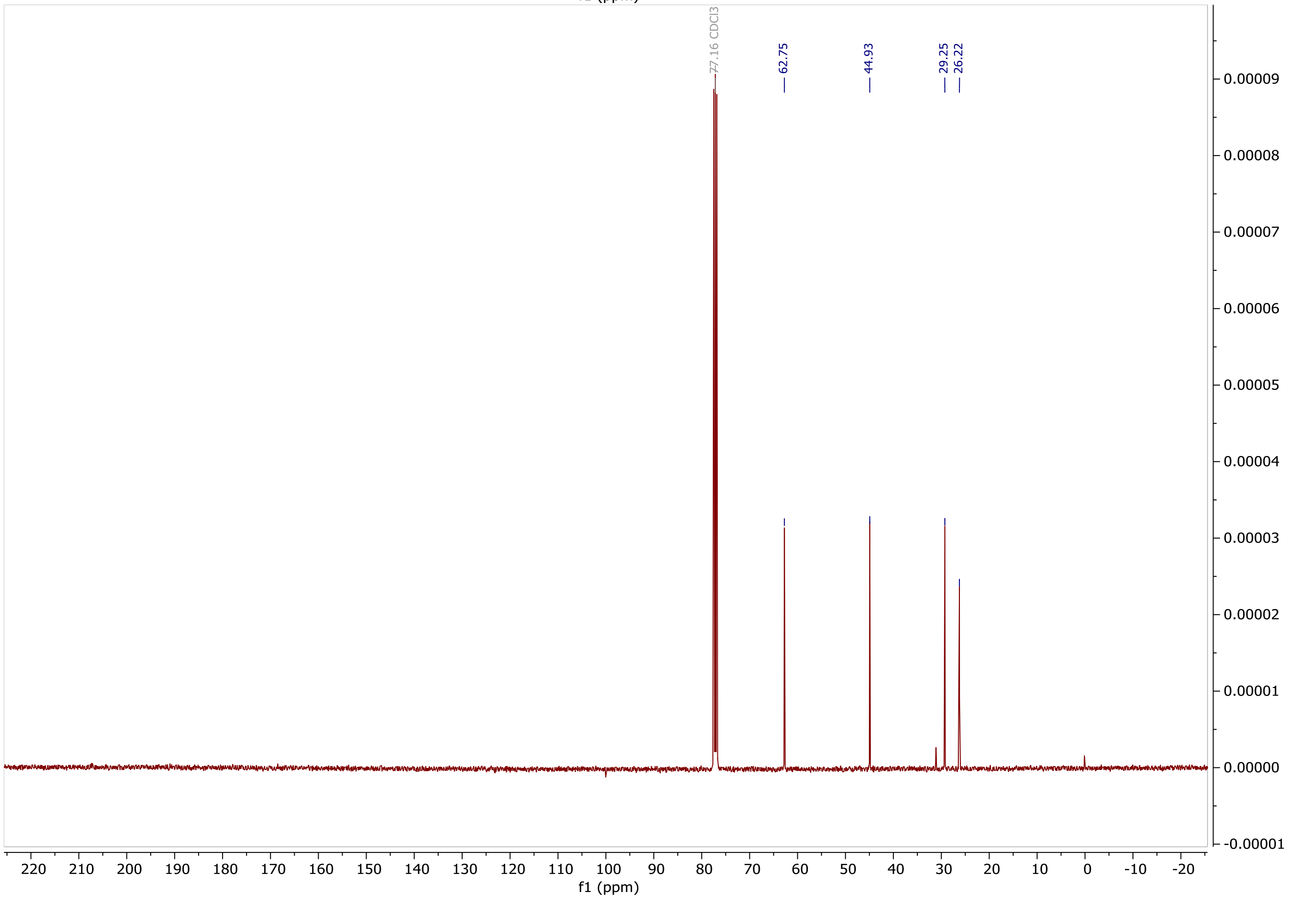

3-Iodobutyl benzoate (S28).

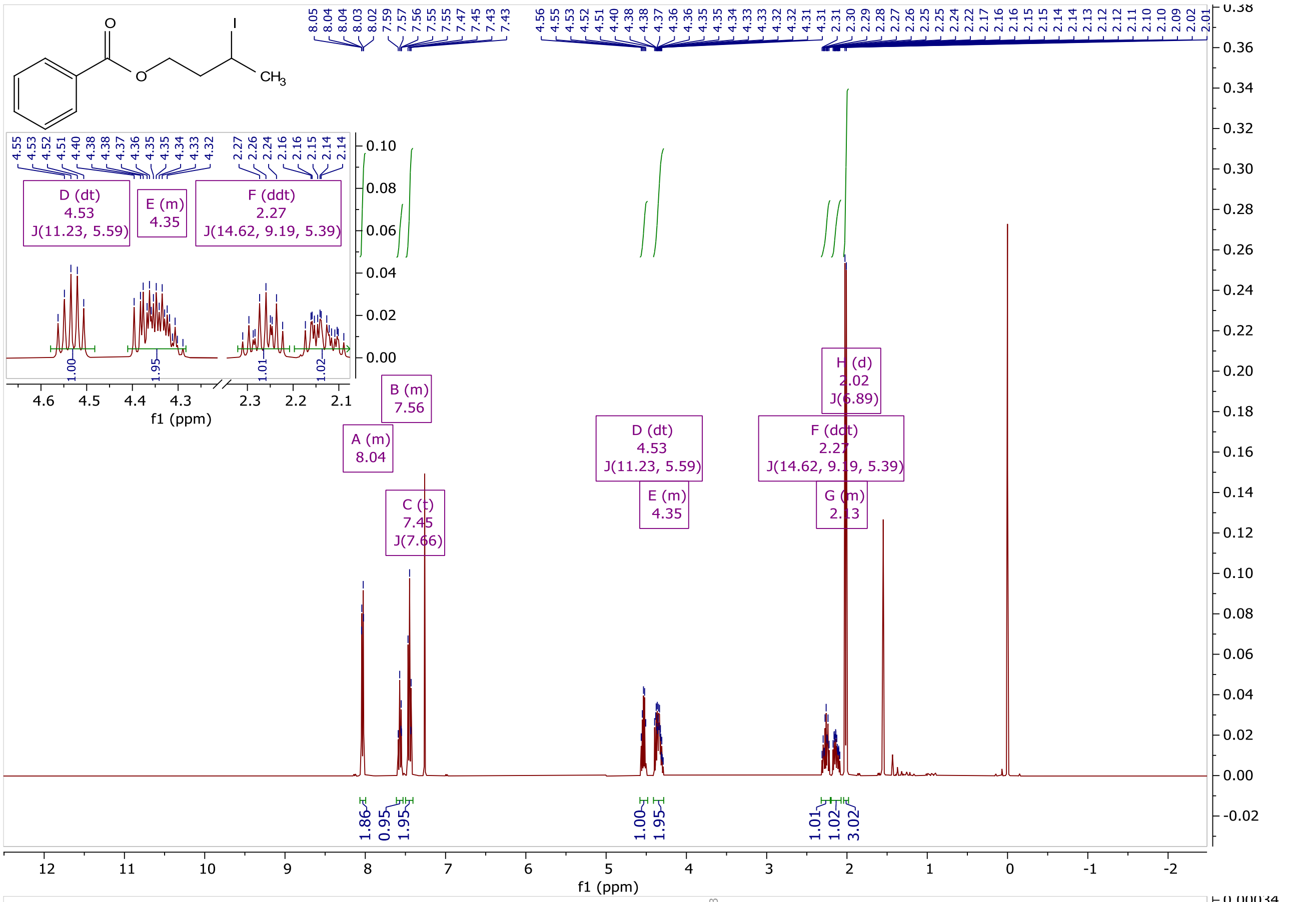

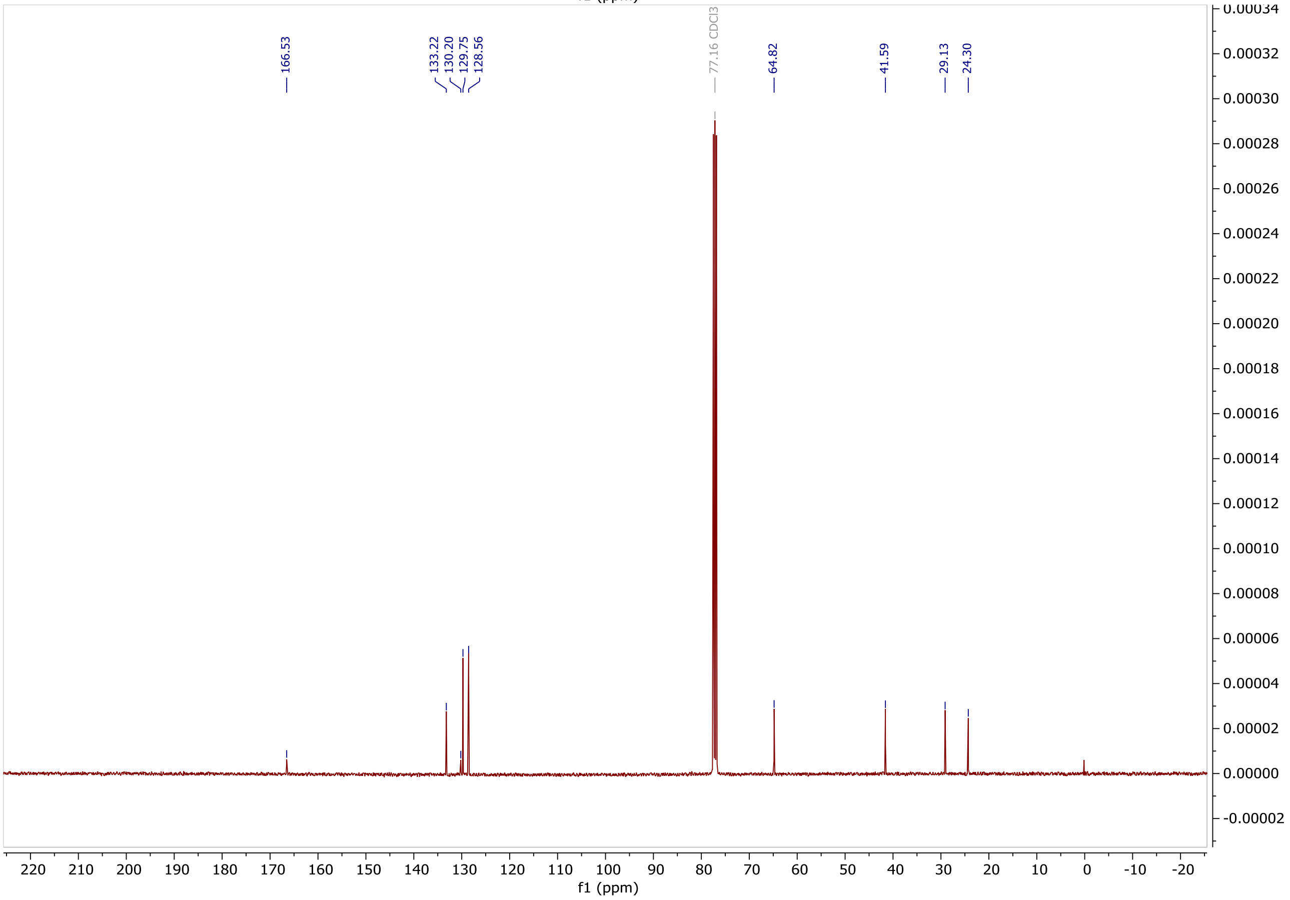

(3-Iodo-3-methylbutyl)benzene (S30).

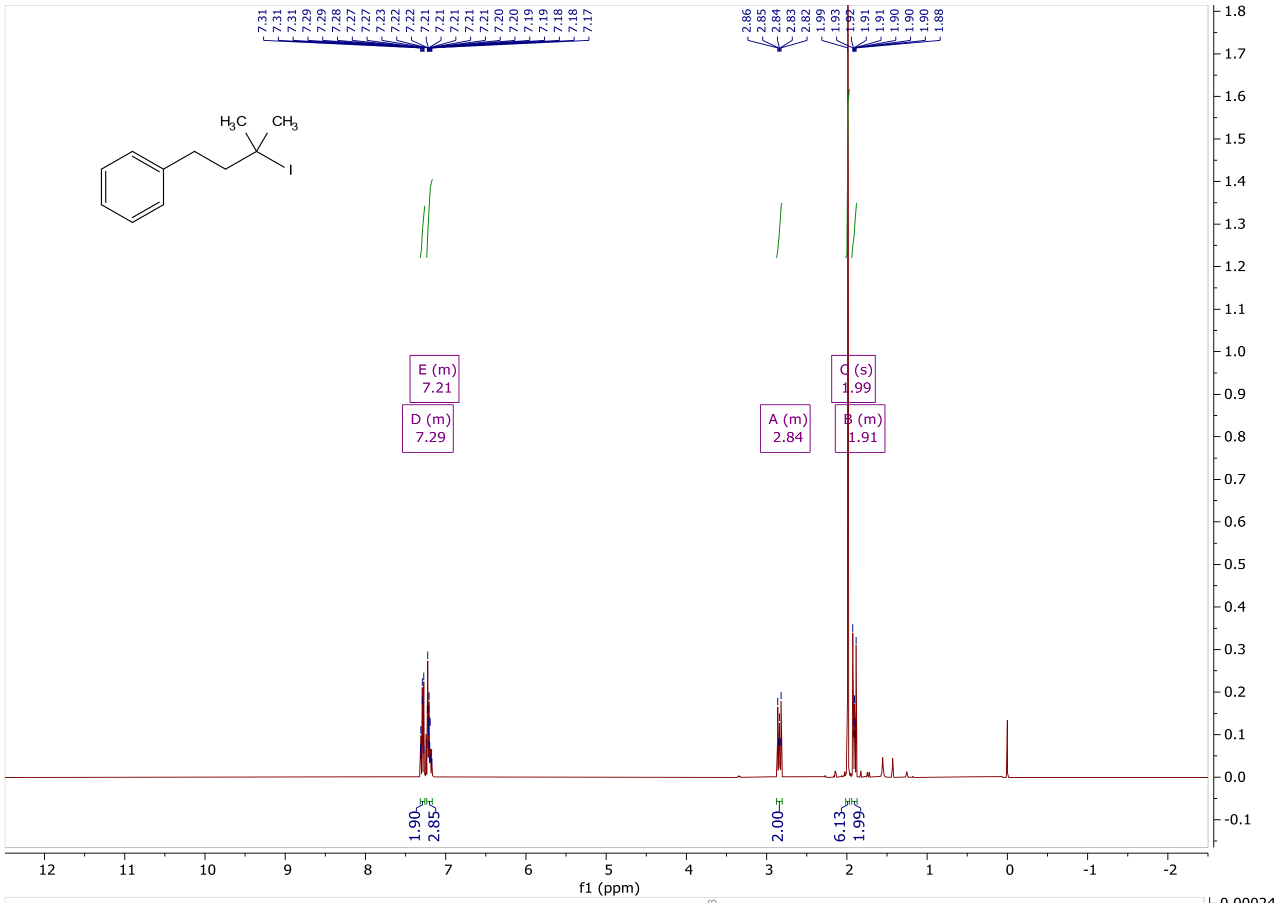

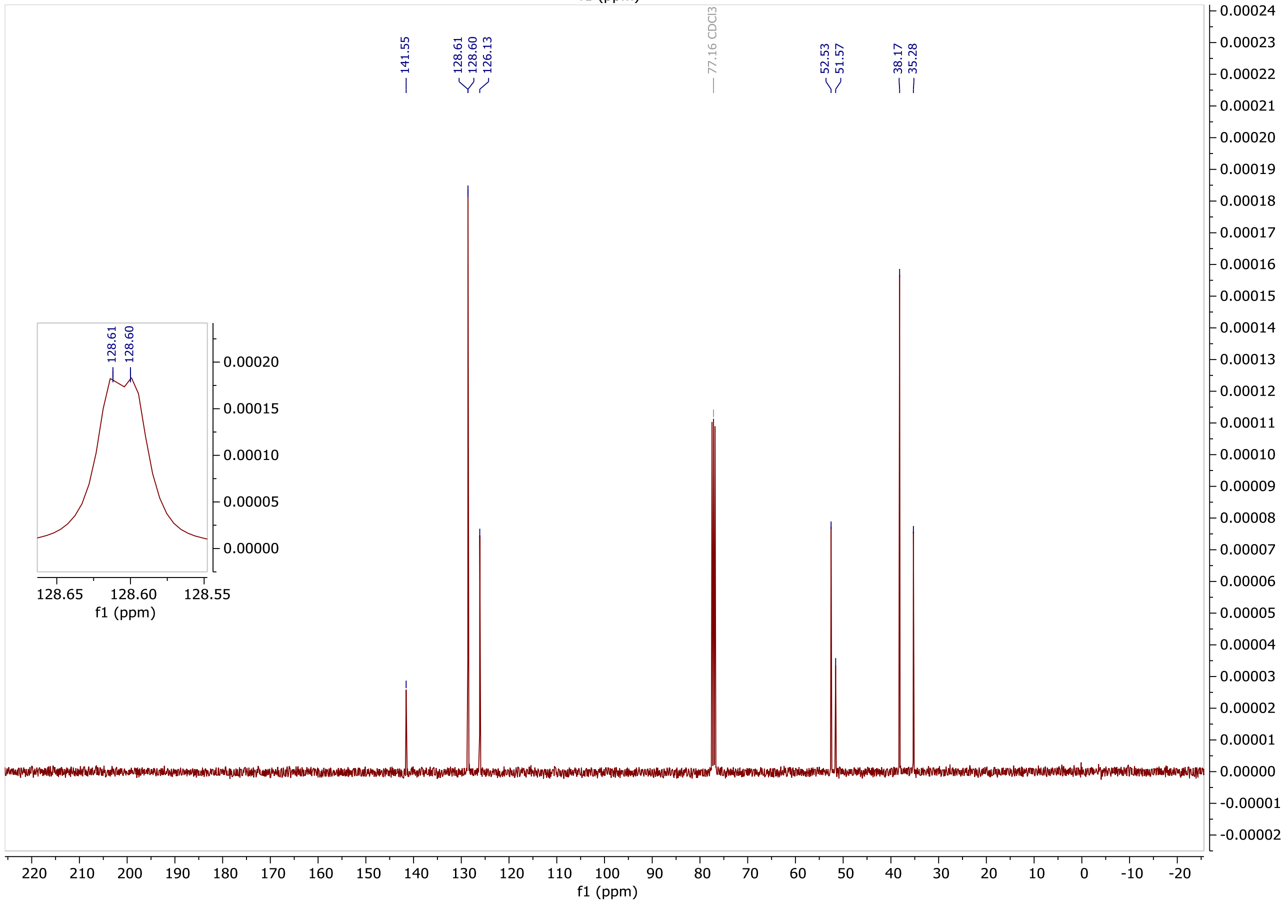

Supplement: Supplementary file 1 [file molecules-30-02584-s001.zip › molecules-3625973-supplementary.pdf]
